# Outcomes Associated With Intracranial Aneurysm Treatments Reported as Safe, Effective, or Durable: A Systematic Review and Meta-Analysis

**DOI:** 10.1001/jamanetworkopen.2023.31798

**Published:** 2023-09-01

**Authors:** Victor Volovici, Iris S. Verploegh, Djaina Satoer, Noëlle J. M. C. Vrancken Peeters, Yasmin Sadigh, Mervyn D. I. Vergouwen, Joost W. Schouten, Gavin Bruggeman, Dana Pisica, Gizem Yildirim, Ayca Cozar, Femke Muller, Ana-Maria Zidaru, Kelsey Gori, Nefeli Tzourmpaki, Esther Schnell, Mbaye Thioub, Kimberly Kicielinski, Pieter-Jan van Doormaal, Nikolay Velinov, Mahjouba Boutarbouch, Michael T. Lawton, Giuseppe Lanzino, Sepideh Amin-Hanjani, Ruben Dammers, Torstein R. Meling

**Affiliations:** 1 Department of Neurosurgery, Erasmus MC Stroke Center, Erasmus MC University Medical Centre, Rotterdam, the Netherlands; 2 Centre for Medical Decision Science, Department of Public Health, Erasmus MC University Medical Centre, Rotterdam, the Netherlands; 3 Department of Neurology and Neurosurgery, UMC Utrecht Brain Center, University Medical Centre Utrecht, Utrecht University, Utrecht, the Netherlands; 4 Department of Neurosurgery, CHNU Fann, University Cheikh Anta Diop, Dakar, Senegal; 5 Department of Neurosurgery, Medical University of South Carolina, Charleston; 6 Department of Interventional Radiology, Erasmus MC Stroke Center, Erasmus MC University Medical Centre, Rotterdam, the Netherlands; 7 Department of Neurosurgery, University Hospital Pirogov, Medical University of Sofia, Sofia, Bulgaria; 8 Department of Neurosurgery, Hopital des Specialites, University Mohammed V, Rabat, Morrocco; 9 Department of Neurosurgery, Barrow Neurological Institute, Phoenix, Arizona; 10 Department of Neurosurgery and Interventional Neuroradiology, Mayo Clinic, Rochester, Minnesota; 11 Department of Neurosurgery, Case Western Reserve, Cleveland, Ohio; 12 Department of Neurosurgery, Rigshospitalet, Copenhagen, Denmark

## Abstract

**Importance:**

Testing new medical devices or procedures in terms of safety, effectiveness, and durability should follow the strictest methodological rigor before implementation.

**Objectives:**

To review and analyze studies investigating devices and procedures used in intracranial aneurysm (IA) treatment for methods and completeness of reporting and to compare the results of studies with positive, uncertain, and negative conclusions.

**Data Sources:**

Embase, MEDLINE, Web of Science, and The Cochrane Central Register of Clinical Trials were searched for studies on IA treatment published between January 1, 1995, and the October 1, 2022. Grey literature was retrieved from Google Scholar.

**Study Selection:**

All studies making any kind of claims of safety, effectiveness, or durability in the field of IA treatment were included.

**Data Extraction and Synthesis:**

Using a predefined data dictionary and analysis plan, variables ranging from patient and aneurysm characteristics to the results of treatment were extracted, as were details pertaining to study methods and completeness of reporting. Extraction was performed by 10 independent reviewers. A blinded academic neuro-linguist without involvement in IA research evaluated the conclusion of each study as either positive, uncertain, or negative. The study followed Preferring Reporting Items for Systematic Reviews and Meta-Analyses guidelines.

**Main Outcomes and Measures:**

The incidence of domain-specific outcomes between studies with positive, uncertain, or negative conclusions regarding safety, effectiveness, or durability were compared. The number of studies that provided a definition of safety, effectiveness, or durability and the incidence of incomplete reporting of domain-specific outcomes were evaluated.

**Results:**

Overall, 12 954 studies were screened, and 1356 studies were included, comprising a total of 410 993 treated patients. There was no difference in the proportion of patients with poor outcome or in-hospital mortality between studies claiming a technique was safe, uncertain, or not safe. Similarly, there was no difference in the proportion of IAs completely occluded at last follow-up between studies claiming a technique was effective, uncertain, or noneffective. Less than 2% of studies provided any definition of safety, effectiveness, or durability, and only 1 of the 1356 studies provided a threshold under which the technique would be considered unsafe. Incomplete reporting was found in 546 reports (40%).

**Conclusions and Relevance:**

In this systematic review and meta-analysis of IA treatment literature, studies claiming safety, effectiveness, or durability of IA treatment had methodological flaws and incomplete reporting of relevant outcomes supporting these claims.

## Key Points

**Question:**

Is there a difference in disease-specific outcomes between studies on intracranial aneurysm (IA) treatment with a positive, uncertain, or negative conclusion regarding safety, effectiveness, or durability, and how are safety, effectiveness, and durability defined?

**Findings:**

In this systematic review and meta-analysis of 1356 studies with 410 993 patients, disease-specific outcomes did not differ between studies with a positive, uncertain, or negative conclusion regarding safety, effectiveness, or durability. Less than 2% of studies reported a definition of safety, effectiveness, or durability.

**Meaning:**

Studies investigating the safety, effectiveness, and durability of IA treatments should follow the highest methodological rigor; the findings of this study suggest that best methodological practices should be improved field-wide.

## Teaser

This systematic review and meta-analysis examines studies investigating devices and procedures used in intracranial aneurysm treatment for methods and completeness of reporting and compares the results of studies with positive, uncertain, and negative conclusions.

## Introduction

In recent decades, the practice of medicine has witnessed an unprecedented rise in the number of medical devices being developed for various specialties. The history of medicine, however, is laden with examples in which the mechanistic logic^1^ that underpins the development of a device does not translate to better outcomes for patients and might, instead, be harmful. A MEDLINE search reveals that “safe and effective” is a mantra used by many reports, with an excess of 150 000 hits boasting these claims in the abstract alone. Even if devices are granted authorization, the evidence may often be of questionable quality or even lacking.^2,3,4^

Aneurysmal subarachnoid hemorrhage (aSAH) is a severe type of hemorrhagic stroke caused by the rupture of an intracranial aneurysm (IA) that affects 6.1 per 100 000 people per year,^5^ of whom more than 30% die within 3 months after ictus.^6^ A vast majority of survivors experience severe cognitive and functional disabilities and less than one-third returns to work.^7,8^ One of the mainstays of treatment is occlusion of the aneurysm, either in an unruptured state to prevent aSAH and increase life-years with good quality of life or after rupture to prevent rebleeding and increase the likelihood of a good outcome.^7^ Complete aneurysmal exclusion from circulation is the main objective of treatment, provided that the risk of treatment complications is lower than the risk of (re)rupture. In terms of endovascular treatment, numerous devices and modifications have been developed and tested in the past 2 decades, several of which have gained US Food and Drug Administration (FDA) approval. This increase of treatment modalities has led to an exponential growth of the number of scientific reports evaluating the safety, effectiveness, and durability of these techniques.

Given the ongoing discussion within the stroke community regarding the best treatment of IAs and proper patient selection, and the paucity of substantial, properly generalizable randomized evidence, we aimed to critically evaluate published claims of safety, effectiveness, and durability of IA treatments. Unsubstantiated claims of safety and effectiveness in biomedical science constitute a potential public health hazard and may influence health policy negatively, leading to suboptimal treatments for patients. We hypothesized that there was a difference in the incidence of disease-specific outcomes between studies with a positive, uncertain, and negative conclusion regarding claims of safety, effectiveness, and durability.

## Methods

The study protocol was registered with PROSPERO, registration number CRD42020169592. The analysis plan was predefined and updated twice (eAppendix 8 in Supplement 1). The study followed the Preferred Reporting Items for Systematic Reviews and Meta-Analyses (PRISMA) reporting guidelines in all aspects except data synthesis, as our objective was not to pool data but to compare the distributions of the reported outcomes.

### Literature Search

We defined the search strategy with the help of a medical information specialist. Using a thrice-refined search algorithm (eAppendix 1 in Supplement 1), studies were retrieved from Embase, Ovid MEDLINE, Web of Science, and The Cochrane Central Register of Clinical Trials. Grey literature was retrieved from Google Scholar. The search was conducted for all records published between January 1, 1995, and the October 1, 2022.

### Inclusion and Exclusion Criteria, Selection Process, and Data Extraction

Studies included had to contain more than 25 patients, as this constitutes in the authors’ opinion a minimum case series, and had to deal with the endovascular or microsurgical treatment of IAs by any device, means, or technique, either individually or combined. We excluded (1) articles that only described a minor modification of an already existing approach or technique, such as a surgical approach; (2) studies dealing with mycotic, traumatic, or purely dissecting aneurysms; and (3) articles solely dealing with aneurysms that recur after treatment. All articles had to mention either safety, effectiveness, or durability in one form or another in the conclusion.

We enrolled a team of 10 master’s degree students with specific interest in interventional neuroradiology, neurology, or neurosurgery and provided formal systematic review training and methods of study design for safety, effectiveness, and durability claims, upon which they were first split into 3 teams and later into 5 teams for independent sifting of studies, extraction of data, and assessment of the outcome parallel to the academic linguist. The process is detailed in eAppendix 8 in Supplement 1.

A professional, academic neuro-linguist, blinded for the type of study (endovascular or microsurgical), with expertise in regulatory assessment of devices and medical products but no extensive knowledge of the field of IA or any involvement in IA research (D.S.), assessed the wording of the conclusions of the included studies. A parallel assessment was conducted by the MSc students who extracted the data. Lexical fields were built for the 3 domains safe, effective, and durable. Every study or study group was labeled positive, uncertain, or negative for each of the 3 domains.

### Definition of the Outcome

A positive conclusion meant the technique was described by the investigators to be safe, effective, or durable in the conclusion without any other comments; uncertain meant that wording was chosen to indicate that a technique might be safe, effective, or durable but only under certain circumstances or with potential caveats; and negative meant that a technique was described as being unsafe, ineffective, or not durable. The neuro-linguist also assessed whether the definition in the abstract was more positive, more negative, or the same as the conclusion in the text. A random sample of 10% of the conclusions were discussed with the first author (V.V.). For studies dealing with unruptured aneurysms, an extra assessment was performed by the second or third author together with the first author. We calculated the concordance rate of these assessments.

### Variables and Data Extraction

For every study and study group (when different therapeutic modalities were compared within one study), we extracted data regarding the context and type of patients included (number of patients and aneurysms, number of centers, aneurysm location, rupture status of aneurysms). We defined (eAppendix 8 in Supplement 1) and extracted domain-specific outcomes. Safety outcomes were in-hospital mortality; poor functional outcome at discharge, defined as either modified Rankin Scale (mRS) 3 to 6 or Glasgow Outcome Score (GOS) 1 to 3; incidence of total complications; and incidence of thromboembolic complications, which were defined as complications leading to ischemia or requiring treatment. For effectiveness, we extracted the rate of complete and adequate occlusion, classified according to the Raymond-Roy Occlusion Classification Scale (RROCS), at discharge and final follow-up.^9^ Any other variation of adequate occlusion, ie, near complete, near total, almost near total, as well as RROCS grade 2 or equivalent were considered adequate. The durability outcome was the proportion of aneurysms with complete occlusion at last follow-up. The follow-up time and proportion of patients available for follow-up were also extracted. Definitions of safety, effectiveness, and durability were also extracted. We recorded whether our predefined domain-specific outcomes were reported at all.

During data extraction, we noticed that some of the studies used various strategies to increase or decrease reported percentages. For example, some reported the proportion of patients with good functional outcome of all living patients at discharge (instead of all included patients). Sometimes the numbers in the various tables did not add up but no explanation was given in the text for the discrepancies. Finally, in some cases, established clinical scales, such as the RROCS, were adjusted to give the appearance of better results. In these cases, we flagged the study under improper reporting.

### Statistical Analysis

The rationale and approved analyses are detailed in eAppendix 8 in Supplement 1. In the main analysis, we compared the proportion of domain-specific outcomes of safety, effectiveness, or durability between studies with a positive, uncertain, or negative conclusion using the Kruskal-Wallis test or the Mann-Whitney *U* test when 1 group was small and only 2 groups were compared. When the Kruskal-Wallis test indicated a statistically significant result, we used Dunn post hoc test to pinpoint which comparisons were statistically significant (positive vs uncertain, uncertain vs negative, positive vs negative). For the rates of complete aneurysmal occlusion at follow-up, we tested whether the 3 groups (positive, uncertain, and negative conclusions) had comparable follow-up periods. When this was not the case, we performed sensitivity analyses by selecting a subset of studies with a follow-up period longer than 9 months, which maximized the number of studies included but kept the distribution of follow-up time between groups comparable.

Second, we summarized the published definitions of safe, effective, and durable together with the characteristics of studies using them. Third, we determined the proportion of studies not reporting our predefined domain-specific outcomes. Fourth, we studied which variables were associated with a higher likelihood of claiming safety, effectiveness, and durability using logistic regression models. Despite the initial analysis plan in terms of the regression analysis, when examining the data, we decided that a simple logistic regression model would be sufficient and provide the best interpretability.

Finally, we performed a sensitivity analysis by reporting the results of intervention groups involving unruptured aneurysms only. These conclusions were independently assessed by 2 of us (I.S.V. or Y.S.) as well as the first author (V.V.). We also extracted for this group whether independent neurological or radiological assessment of patients was performed and the incidence of major complications (stroke, ischemic or hemorrhagic). Statistical significance was set at α = .05, and tests were 2-tailed. R version 4.2.1 (R Project for Statistical Computing) was used for all analyses.

## Results

**Figure 1.**
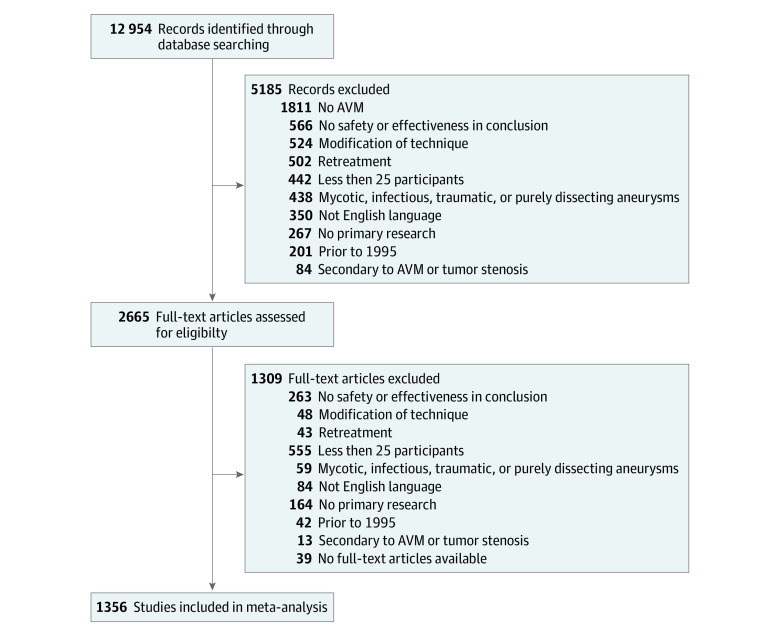
Flowchart of the Included Studies AVM indicates arteriovenous malformations.

We included 1356 studies comprising 410 993 patients, of whom 261 675 underwent endovascular aneurysm treatment and 149 300 microsurgical aneurysm treatment (Figure 1 and eAppendix 9 in Supplement 1). Of the included studies, 1055 were single-group studies with 129 882 patients, and 301 were multigroup studies with 281 111 patients. Of the multigroup studies, 287 were 2-group studies, 11 were 3-group studies, and 3 were 4-group studies.

### Baseline Characteristics

Of all 1674 intervention groups included in this analysis, 1330 (79%) were endovascular interventions, 343 (20%) were microsurgical interventions, and 1 was a combined endovascular and microsurgical approach. Of the endovascular groups, in 554 (42%) the intervention was simple coiling, in 342 (26%) the intervention was stent- or balloon-assisted coiling, in 266 (16%) the intervention was a flow diverter, and in 61 (3%) a WovenEndoBridge device or another intrasaccular device. The rest of the endovascular interventions were classified as other (mainly combined endovascular interventions). Microsurgical interventions included different clip reconstruction modalities, with or without bypass flow replacement.

The 1674 intervention group included a median (IQR) of 63 (37-126) patients harboring 67 (38-130) aneurysms per group. Most studies were carried out in the United States (311 studies [23%]), China (199 studies [15%]), and South Korea (109 studies [8%]). The comparative studies were carried out mostly in the United States (81 studies [26%]), Northwestern Europe (52 studies [16%]), and China (47 studies [15%]). The single-group studies included 172 (16%) prospective observational studies, with the rest being retrospective observational ones. The comparative studies included 35 (12%) prospective observational studies and 9 (6%) randomized clinical trials (RCTs). Three reports included RCT subgroup analyses; 2 reports, periprocedural outcomes; 2 reports, long-term follow-up; and 1 report, safety assessments.

The concordance rate between the linguist and the other extractors was 87%, which was considered very good. In the sensitivity analysis for unruptured aneurysms only the concordance rate between the linguist and the clinicians was 92% (eAppendix 4 in Supplement 1 and eTable in Supplement 2).

### Main Analysis: Safety

Of the 1060 intervention groups mentioning safety in the conclusion, 821 (77%) were deemed safe, 235 (22%) were categorized as uncertain, and 4 (0.4%) were categorized as not safe. There was no difference in the incidence of mortality at discharge between studies with a positive, uncertain, or negative conclusion (median [range]: positive, 0.7% [0%-50.9%]; uncertain, 1.6% [0%-34.4%]; negative, 2.2% [0%-18.0%], *P* = .46) (Table 1 and eAppendix 7 in Supplement 1). Similarly, there was no difference in poor functional outcome at discharge between studies with a positive, uncertain, or negative conclusion (Table 1).

**Table 1.** Main Analysis

Outcome	Median (IQR) [range]			*P* value
	Positive	Uncertain	Negative	
**Safety**				
Intervention groups, No.	821	235	4	NA
Total complications, %	10.5 (6.0-16.0) [0-100]	13.5 (10-19.6) [0-78.1]	21.8 (15.6-33.4) [9.3-45]	<.001
Poor outcome at discharge, %	7.2 (2.4-17.0) [0-86.7]	9.1 (2.5-26.8) [0-100]	1.8^a^	.13
Thromboembolic complication, %	4.8 (2.4-8.1) [0-74.1]	6.0 (3.2-9.1) [0-35.9]	17.5^a^	.02
In-hospital mortality, %	0.7 (0.0-3.0) [0-50.9]	1.6 (0-4.6) [0-34.4]	2.2 (1.4-6.5) [0.35-18]	.46
**Effectiveness**				
Intervention groups	826	195	4	NA
Complete occlusion at discharge, %	61.4 (41.8-80.9) [0.0-100]	62.0 (42.8-76.9) [0.5-100]	51.8 (49.4-54.3) [46.9-56.7]	.82
Complete occlusion at final FU, %	78.8 (68.4-88.0) [3.0-100]	75.0 (61.8-87.2) [3.5-98.8]	61.9 (59.9-63.8) [58.0-65.7]	.08
Adequate occlusion at discharge, %	23.1 (13.4-35.8) [0.0-96.2]	24.0 (12.5-34.0) [0.0-94.0]	37.7 (37.6-37.7) [37.5-37.8]	.32
Adequate occlusion at final FU, %	13.6 (7.6-25.0) [0-89.1]	11.3 (6.9-22.6) [0-69.0]	23^a^	.59
**Durability**				
Intervention groups, No.	94	64	6	NA
Complete occlusion at final FU, %	83.1 (72.7-90.1) [42.0-100]	76.9 (64.7-89.9) [35-100]	70.3 (64.2-72.3) [58-74.2]	.09
FU time, mo	21.3 (12.0-38.8) [5.9-132]	12.4 (9.2-23.9) [6.0-101]	20.5 (15.2-25.5) [10-30]	.04

Abbreviations: FU, follow-up; NA, not applicable.

^a^ Only 1 study was considered, so there is no IQR or range.

A definition of safety was available in 27 reports (2%) in the methods section (Table 2).^10,11,12,13,14,15,16,17,18,19,20,21,22,23,24,25,26,27,28,29,30,31,32,33,34,35,36^ Most (25 of 27 [93%]) detailed the outcomes used to claim safety and the follow-up time but not an incidence threshold used to support the safety claim. Of the 27 reports, 5 (19%) included safety definitions that were available in a published protocol. Two studies (7%) included an incidence threshold, under which the intervention would not be considered safe, either under 15% or under 25% of included patients after a predefined follow-up. The outcome to which the threshold applied was defined as “procedural complications, mortality, or morbidity,”^16,36^ but it was unclear which of the 3 should demonstrate an incidence below the threshold for safety to be claimed.

**Table 2.** Definitions of Safety Available in 27 Studies

Definitions	Study type	Devices	Rupture status	Predefined in protocol	Follow-up moments (No. studies)	Incidence of outcomes deemed safe, (No. studies)
Rate of procedural complications, mortality, and morbidity (7 studies)^10,11,12,13,14,15,16^	3 RO, 3 PO, 1 RCT	3 coil, 1 WEB, 1 FD, 1 clip vs coil, 1 SAC vs SAC	1 aSAH, 6 both	0	At discharge (1), 12 mo (2), 3-6 mo (1)	<25% (1)
Neurological death or major (ipsilateral) stroke (6 studies)^17,18,19,20,21,22^	1 RO, 5 PO	2 FD, 4 SAC	2 elective, 4 both	2 studies	6 mo (1), 12 mo (4)	<15% (1)
Neurological death or major (ipsilateral) stroke or deterioration of the clinical condition (mRS 3-6, NIHSS 4 points) (14 studies)^23,24,25,26,27,28,29,30,31,32,33,34,35,36^	3 RO, 10 PO, 1 RCT	2 WEB, 6 FD, 3 SAC, 2 SAC vs coil, 1 coil vs coil	1 aSAH, 4 elective, 9 both	3 studies	1 mo (1), 3 mo (1), 6 mo (2), 12 mo (6)	NA

Abbreviations: aSAH, aneurysmal subarachnoid hemorrhage; FD, flow diverter; mRS, modified Rankin Scale; NIHSS, National Institutes of Health Stroke Scale; PO, prospective observational; RCT, randomized clinical trial; RO, retrospective observational; SAC, stent-assisted coiling; WEB, WovenEndoBridge.

### Main Analysis: Effectiveness

Of the 1025 intervention groups evaluated for effectiveness, 826 (81%) were deemed effective, 195 (19%) were categorized as uncertain, and 4 (0.4%) as not effective. There was no difference between the incidence of complete occlusion at discharge between studies with positive, uncertain, and negative conclusions (median [range]: positive, 61.4% [0%-100%]; uncertain, 62.0% [0.5%-100%]; negative, 51.8% [46.9%-56.7%]; *P* = .82). Similarly, there was no difference between the incidence of complete occlusion at final follow-up between studies with positive, uncertain, and negative conclusions (median [range]: positive, 78.8% [3.0%-100%]; uncertain, 75.0% [3.5%-98.8%]; negative, 61.9% [58.0%-65.7%]; *P* = .09). Results were similar when we restricted follow-up time between 9 months and 5 years, thereby keeping groups comparable in terms of follow-up period (eAppendix 2 in Supplement 1).

A definition of effectiveness was available in 2 studies (0.1%) (eAppendix 6 in Supplement 1). There were no thresholds available under which an intervention would be considered ineffective. Twenty-five other reports included definition of effectiveness at the aneurysm level, meaning when an aneurysm would be considered effectively treated, but these were excluded as we were only interested in group-level definitions.

### Main Analysis: Durability

Of the 164 intervention groups evaluated for durability, 94 (58%) were considered durable, 64 (39%) were categorized as uncertain and 6 (2%) were not durable. The median number of aneurysms with complete occlusion at final follow-up did not differ between studies with positive, uncertain, or negative conclusions (median [range]: positive, 83.1% [42.0%-100%]; uncertain, 76.9% [35.0%-100%]; negative, 70.3% [58.0%-74.2%]; *P* = .10) (Table 1). The studies with positive conclusions had a higher median (range) proportion of patients available for follow-up (positive, 84.0% [15.4%-100%]; uncertain, 69.9% [33.0%-100%]; negative, 77.3% [73.5%-96.9%]; *P* = .02). Results were similar when we restricted follow-up time to 9 months to 5 years, thereby keeping groups comparable in terms of follow-up period (eAppendix 2 in Supplement 1). No studies reported a definition of durability.

### Subgroup Analysis

When analyzing only studies including patients with unruptured aneurysms, there was a statistically significant difference in terms of median (range) in-hospital mortality between studies with a positive and those with an uncertain conclusion, but not between studies with a positive and those with a negative conclusion or between those with an uncertain conclusion and those with a negative conclusion (positive, 0% [0%-5.6%]; uncertain, 0.2% [0%-20.1%]; negative, 1.0% [0.4%-1.7%], *P* = .009). There was no difference in terms of median (range) poor outcome at discharge between studies with a positive, uncertain, or negative conclusion (positive, 2.0% [0%-27.5%], uncertain: 2.5% [0%-100%], negative: 1.8%, *P* = .79) (eAppendix 2 in Supplement 1). There were no differences in terms of effectiveness or durability outcomes between studies with a positive, negative, or uncertain conclusion (eAppendix 2 in Supplement 1).

The sensitivity analysis revealed that the incidence of major complications ranged between 0% and 28%. There was no difference between studies with a positive, negative, or uncertain conclusion (median [range]: positive, 3.8% [0%-21.0%]; uncertain, 7.2% [0%-21.5%]; negative, 24.1% [20.0%-28.0%]; *P* = .50) Of the intervention groups, 45 (21%) had an incidence rate of major complications greater than 10%. Thirty-two intervention groups (14%) had independent neurological assessment, and 16 (7%) had independent neurological assessment of patients (eAppendix 4 in Supplement 1).

### Risk of Bias

**Figure 2.**
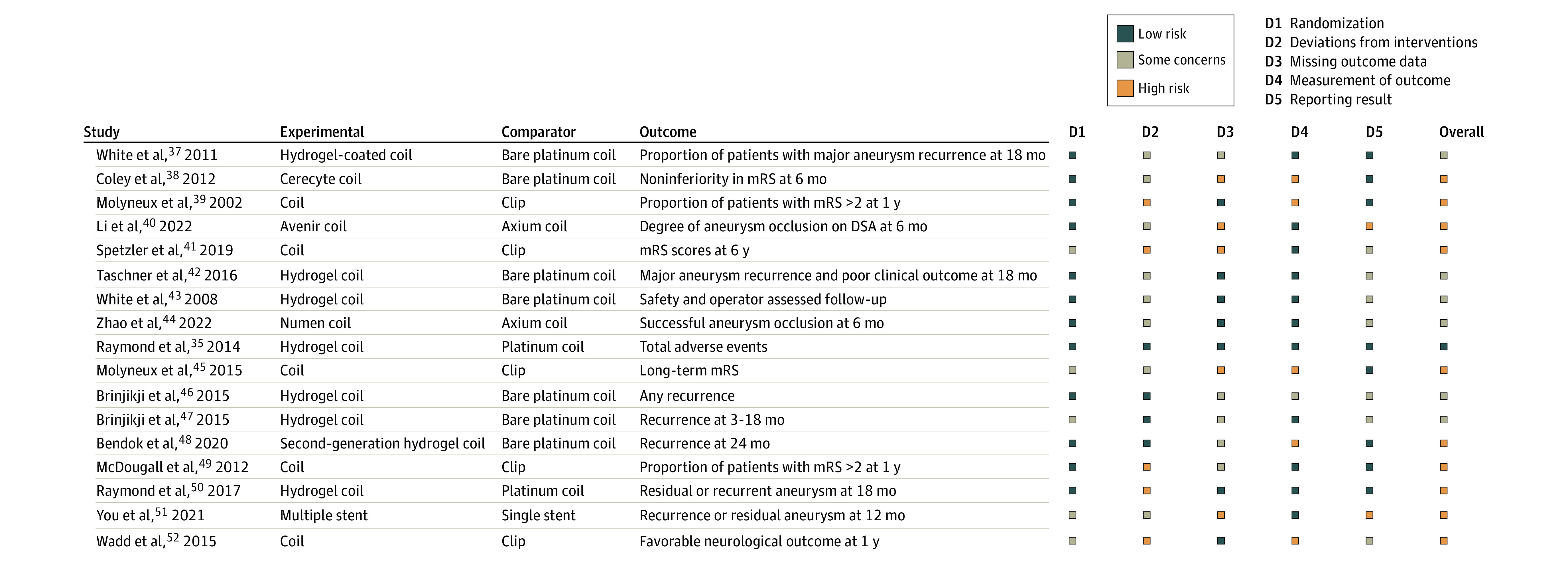
Risk of Bias Analysis of Intracranial Aneurysm Treatment Randomized Clinical Trials and Related Publications DSA indicates digital subtraction angiography; mRS, modified Rankin Scale.

Ten of the 17 RCT reports (59%) showed a high risk of bias (Figure 2).^35,37,38,39,40,41,42,43,44,45,46,47,48,49,50,51,52^ We found improper reporting in 546 of the included studies (40%) (eAppendix 3 in Supplement 1). In 68 studies (5%), the conclusion in the abstract was more positive than the conclusion in the text.

### Results of Regression Analyses

Regression analysis indicated that studies on stent-assisted coiling and with a lower incidence of total complications were associated with a higher likelihood of claiming safety in the conclusion (stent-assisted coiling: adjusted odds ratio [aOR], 1.9 [95% CI, 1.1-3.8]; complication incidence: β = −0.027; SE, 0.01). Similarly, studies on stent-assisted coiling (aOR, 2.0; 95% CI, 1.1-3.9]) and with a higher incidence of complete occlusion at last follow-up (β= 0.016; SE, 0.007) were associated with a higher likelihood of claiming effectiveness in the conclusion (eAppendix 5 in Supplement 1).

## Discussion

In this systematic review and meta-analysis of IA treatment, we found that approximately 80% of the included papers had a positive conclusion on either safety or effectiveness. Most of these papers were single-group and retrospective in nature. In terms of safety, there were no differences in the proportions of patients with poor outcomes between studies with a positive, uncertain, or negative conclusion. When limiting the results to studies on unruptured aneurysms, only the rate of in-hospital mortality was different between the studies with a positive and uncertain conclusion regarding safety. In studies claiming a technique was safe to use in unruptured aneurysms, the maximum proportion of patients with in-hospital mortality was 5%; poor functional outcome, 27.5%; and major complications, 28%. There was no difference in the proportion of aneurysms with a complete occlusion at last follow-up between studies with a positive, uncertain, or negative conclusion neither in terms of effectiveness nor in terms of durability. More than half of all articles had improper reporting. All but 1 RCT showed high or concerning risk of bias in essential domains.

Proving safety and effectiveness of a new technique is important prior to the widespread implementation of such a technique in clinical practice, and its prerequisite is common sense: a new technique should be compared with the current standard of care. Ideally, this should be done in a multicenter RCT, with predefined time points, with blinded, uniform assessment of clinically and radiologically relevant outcomes, with predefined analyses and predefined “safety” and “effectiveness” outcomes. These desiderates are currently almost entirely absent in published studies. If the results of such a study fall within the predefined noninferiority margin compared with current practice, a technique may be adopted in clinical practice. Comparing a new technique with current practice in a prospective manner should be considered the bare minimum for any safety or effectiveness claim to be allowed in a report. Of course, safety may also be claimed when the incidence of complications is low, but without a control group one cannot infer whether the technique should be implemented in practice.

Single-group studies lack a critical benchmark, in that the results obtained cannot be reflected against the results of current standard clinical practice, which makes any claim of safety, effectiveness, or durability weak or unjustifiable. Investigators may be reluctant to label techniques as ineffective or unsafe, as evidenced by the very low number of negative conclusions. Only 0.002% of studies included any kind of incidence threshold for safety, effectiveness, or durability in their definition.

New medical devices must undergo evaluation and approval by regulatory agencies, such as the FDA. This can be done either by providing proof of safety and effectiveness from clinical trials or by the 510(k) clearance process, in which a new device is expected to fare as well as an already existing and approved device. Although this process accelerates innovation, there is evidence to suggest that the latter process is less robust and may lead to more recalls. In addition, the scientific literature backing this process may be methodologically less sound and sometimes even lacking.^4,53,54,55^ A thorough, critical evaluation of the evidence used to back the FDA approval of these devices is necessary. In our view, the current literature reflects a failure of the peer-review process. In a field with a very high level of technological advance, and with techniques that all carry a certain morbidity and mortality risk, reviewers and editors should be critical and hesitant to accept manuscripts with poor methods and reporting and make sure that conclusions are supported by study results.

### Limitations

This study has limitations, including the missing data in the studies; the different definitions of, for example, thrombo-embolic complications or ischemia; the differences in case-mix; and the fact that in many studies results were not reported stratified for patients with aSAH and those with unruptured aneurysms. Another limitation was the fact that the second assessor of the conclusions was not always the same, which may have affected the uniformity of the assessment. We attempted to mitigate the effects of potentially unreliable assessment through training of the assessor and through the assessor’s experience and knowledge.

## Conclusions

In this study, we found that studies claiming safety, effectiveness or durability of IA treatment often had methodological flaws and incomplete reporting of relevant outcomes supporting these claims. The consequences for patients with IA can hereby form a potential public health hazard. The field of IA treatment can be improved by applying proper methods and reporting and performing comparative studies in which new technologies are compared with current best clinical practice.

## Notes

Supplement 1. **eAppendix 1.** Search Strategy **eAppendix 2.** Main Analysis: Sensitivity Analyses and Stratified Results for Effect Modifiers **eAppendix 3.** Frequency of Essential Domain-Specific Outcomes Not Reported in the Included Studies **eAppendix 4.** Sensitivity Analysis Including Only Studies With More Than 100 Patients **eAppendix 5.** Regression Results **eAppendix 6.** Correlations and Cross-Tables **eAppendix 7.** Supplementary Figures **eAppendix 8.** Statistical Analysis Plan and Data Dictionary **eAppendix 9.** Characteristics of Included Studies
eReferences.

Supplement 2. **eTable.** Sensitivity Analysis: Studies or Study Arms Reporting Results of Unruptured Aneurysms Only

Supplement 3.
Data Sharing Statement
