# Supplementary material for: Outcomes Associated With Intracranial Aneurysm Treatments Reported as Safe, Effective, or Durable: A Systematic Review and Meta-Analysis
Source: JAMA Netw Open. 2023 Sep 1;6(9):e2331798. doi: 10.1001/jamanetworkopen.2023.31798 (PMC10474558; doi:10.1001/jamanetworkopen.2023.31798)
Supplement: Supplement 1. — eAppendix 1. Search Strategy eAppendix 2. Main Analysis: Sensitivity Analyses and Stratified Results for Effect Modifiers eAppendix 3. Frequency of Essential Domain-Specific Outcomes Not Reported in the Included Studies eAppendix 4. Sensitivity Analysis Including Only Studies With More Than 100 Patients eAppendix 5. Regression Results eAppendix 6. Correlations and Cross-Tables eAppendix 7. Supplementary Figures eAppendix 8. Statistical Analysis Plan and Data Dictionary eAppendix 9. Characteristics of Included Studies eReferences. [file jamanetwopen-e2331798-s001.pdf]

Supplemental Online Content

Volovici V, Verploegh IS, Satoer D, et al. Outcomes associated with intracranial aneurysm treatments reported as safe, effective, or durable: a systematic review and meta-analysis. *JAMA Netw Open*. 2023;6(9):e2331798. doi:10.1001/jamanetworkopen.2023.31798

eAppendix 1. Search Strategy

eAppendix 2. Main Analysis: Sensitivity Analyses and Stratified Results for Effect Modifiers

eAppendix 3. Frequency of Essential Domain-Specific Outcomes Not Reported in the Included Studies

eAppendix 4. Sensitivity Analysis Including Only Studies With More Than 100 Patients

eAppendix 5. Regression Results

eAppendix 6. Correlations and Cross-Tables

eAppendix 7. Supplementary Figures

eAppendix 8. Statistical Analysis Plan and Data Dictionary

eAppendix 9. Characteristics of Included Studies

eReferences.

This supplemental material has been provided by the authors to give readers additional information about their work.

eAppendix 1. Search Strategy

Embase

('intracranial aneurysm'/exp OR (((intracranial* OR cranial* OR cerebral* OR intracerebral* OR brain*) NEAR/3 (aneurysm*))):ab,ti,kw) AND ('clip'/de OR 'aneurysm clip'/de OR 'blood vessel clip'/de OR 'clipping'/de OR 'coil embolization'/de OR 'endovascular aneurysm repair'/de OR 'flow diverter'/de OR 'intracranial stent'/de OR 'vascular plug'/de OR 'embolization device'/de OR 'neurovascular embolization device'/de OR 'embolization coil'/de OR 'artificial embolization'/de OR (clip OR clips OR clipping OR coil OR coils OR coiling OR (endovascular* NEAR/3 (repair* OR therap* OR treat* OR catheter* OR intervention* OR emboli*)) OR stent* OR (flow NEAR/3 (diverter* OR diversion*)) OR web-device* OR (woven NEAR/3 endobridge) OR (vascular* NEAR/3 plug*) OR ((embolization OR embolisation) NEAR/3 device*)):ab,ti,kw) NOT ('case report'/de OR case-report*:ti) AND (((20 OR 21 OR 22 OR 23 OR 24 OR 25 OR 26 OR 27 OR 28 OR 29 OR 30 OR 31 OR 32 OR 33 OR 34 OR 35 OR 36 OR 37 OR 38 OR 39 OR 40 OR 41 OR 42 OR 43 OR 44 OR 45 OR 46 OR 47 OR 48 OR 49 OR 50 OR 51 OR 52 OR 53 OR 54 OR 55 OR 56 OR 57 OR 58 OR 59 OR 60 OR 61 OR 62 OR 63 OR 64 OR 65 OR 66 OR 67 OR 68 OR 69 OR 70 OR 71 OR 72 OR 73 OR 74 OR 75 OR 76 OR 77 OR 78 OR 79 OR 80 OR 81 OR 82 OR 83 OR 84 OR 85 OR 86 OR 87 OR 88 OR 89 OR 90 OR 91 OR 92 OR 93 OR 94 OR 95 OR 96 OR 97 OR 98 OR 99 OR 00? OR 01? OR 02? OR 03? OR 04? OR 05? OR 06? OR 07? OR 08? OR 09? OR 10? OR 11? OR 12? OR 13? OR 14? OR 15? OR 16? OR 17? OR 18? OR 19? OR 20? OR 21? OR 22? OR 23? OR 24? OR 25? OR 26? OR 27? OR 28? OR 29? OR 30? OR 31? OR 32? OR 33? OR 34? OR 35? OR 36? OR 37? OR 38? OR 39? OR 40? OR 41? OR 42? OR 43? OR 44? OR 45? OR 46? OR 47? OR 48? OR 49? OR 50? OR 51? OR 52? OR 53? OR 54? OR 55? OR 56? OR 57? OR 58? OR 59? OR 60? OR 61? OR 62? OR 63? OR 64? OR 65? OR 66? OR 67? OR 68? OR 69? OR 70? OR 71? OR 72? OR 73? OR 74? OR 75? OR 76? OR 77? OR 78? OR 79? OR 80? OR 81? OR 82? OR 83? OR 84? OR 85? OR 86? OR 87? OR 88? OR 89? OR 90? OR 91? OR 92? OR 93? OR 94? OR 95? OR 96? OR 97? OR 98? OR 99? OR 10?? OR 11?? OR 12?? OR 13?? OR 14?? OR 15?? OR 16?? OR 17?? OR 18?? OR 19?? OR 20?? OR 21?? OR 22?? OR 23?? OR 24?? OR 25?? OR 26?? OR 27?? OR 28?? OR 29?? OR 30?? OR 31?? OR 32?? OR 33?? OR 34?? OR 35?? OR 36?? OR 37?? OR 38?? OR 39?? OR 40?? OR 41?? OR 42?? OR 43?? OR 44?? OR 45?? OR 46?? OR 47?? OR 48?? OR 49?? OR 50?? OR 51?? OR 52?? OR 53?? OR 54?? OR 55?? OR 56?? OR 57?? OR 58?? OR 59?? OR 60?? OR 61?? OR 62?? OR 63?? OR 64?? OR 65?? OR 66?? OR 67?? OR 68?? OR 69?? OR 70?? OR 71?? OR 72?? OR 73?? OR 74?? OR 75?? OR 76?? OR 77?? OR 78?? OR 79?? OR 80?? OR 81?? OR 82?? OR 83?? OR 84?? OR 85?? OR 86?? OR 87?? OR 88?? OR 89?? OR 90?? OR 91?? OR 92?? OR 93?? OR 94?? OR 95?? OR 96?? OR 97?? OR 98?? OR 99??) NEXT/3 (patients OR patient OR subjects OR individuals OR cases OR persons OR men OR women OR males OR females OR participant* OR people OR children OR adolescent* OR boys OR girls OR teens OR teenagers OR infants OR newborns OR elderly OR survivor* OR specimen* OR sample* OR episode* OR isolate* OR pediatric OR paediatric OR aneurysm*)) OR ((twenty* OR thirty* OR forty* OR fifty* OR sixty* OR seventy* OR eighty* OR ninety* OR hundred OR thousand OR million) NEXT/5 (patients OR patient OR subjects OR individuals OR cases OR persons OR men OR women OR participants OR people OR children OR adolescent* OR boys OR girls OR teens OR teenagers OR infants OR newborns OR elderly OR survivor* OR specimen* OR sample* OR episode* OR isolate* OR pediatric OR paediatric OR aneurysm*)) OR ((n OR included OR recruited OR randomized OR randomized OR assigned) NEXT/2 (20 OR 21 OR 22 OR 23 OR 24 OR 25 OR 26 OR 27 OR 28 OR 29 OR 30 OR 31 OR 32 OR 33 OR 34 OR 35 OR 36 OR 37 OR 38 OR 39 OR 40 OR 41 OR 42 OR 43 OR 44 OR 45 OR 46 OR 47 OR 48 OR 49 OR 50 OR 51 OR 52 OR 53 OR 54 OR 55 OR 56 OR 57 OR 58 OR 59 OR 60 OR 61 OR 62 OR 63 OR 64 OR 65 OR 66 OR 67 OR 68 OR 69 OR 70 OR 71 OR 72 OR 73 OR 74 OR 75 OR 76 OR 77 OR 78 OR 79 OR

© 2023 Volovici V et al. *JAMA Network Open.*

80 OR 81 OR 82 OR 83 OR 84 OR 85 OR 86 OR 87 OR 88 OR 89 OR 90 OR 91 OR 92 OR 93 OR 94 OR 95 OR 96 OR 97 OR 98 OR 99 OR 00? OR 01? OR 02? OR 03? OR 04? OR 05? OR 06? OR 07? OR 08? OR 09? OR 10? OR 11? OR 12? OR 13? OR 14? OR 15? OR 16? OR 17? OR 18? OR 19? OR 20? OR 21? OR 22? OR 23? OR 24? OR 25? OR 26? OR 27? OR 28? OR 29? OR 30? OR 31? OR 32? OR 33? OR 34? OR 35? OR 36? OR 37? OR 38? OR 39? OR 40? OR 41? OR 42? OR 43? OR 44? OR 45? OR 46? OR 47? OR 48? OR 49? OR 50? OR 51? OR 52? OR 53? OR 54? OR 55? OR 56? OR 57? OR 58? OR 59? OR 60? OR 61? OR 62? OR 63? OR 64? OR 65? OR 66? OR 67? OR 68? OR 69? OR 70? OR 71? OR 72? OR 73? OR 74? OR 75? OR 76? OR 77? OR 78? OR 79? OR 80? OR 81? OR 82? OR 83? OR 84? OR 85? OR 86? OR 87? OR 88? OR 89? OR 90? OR 91? OR 92? OR 93? OR 94? OR 95? OR 96? OR 97? OR 98? OR 99? OR 10?? OR 11?? OR 12?? OR 13?? OR 14?? OR 15?? OR 16?? OR 17?? OR 18?? OR 19?? OR 20?? OR 21?? OR 22?? OR 23?? OR 24?? OR 25?? OR 26?? OR 27?? OR 28?? OR 29?? OR 30?? OR 31?? OR 32?? OR 33?? OR 34?? OR 35?? OR 36?? OR 37?? OR 38?? OR 39?? OR 40?? OR 41?? OR 42?? OR 43?? OR 44?? OR 45?? OR 46?? OR 47?? OR 48?? OR 49?? OR 50?? OR 51?? OR 52?? OR 53?? OR 54?? OR 55?? OR 56?? OR 57?? OR 58?? OR 59?? OR 60?? OR 61?? OR 62?? OR 63?? OR 64?? OR 65?? OR 66?? OR 67?? OR 68?? OR 69?? OR 70?? OR 71?? OR 72?? OR 73?? OR 74?? OR 75?? OR 76?? OR 77?? OR 78?? OR 79?? OR 80?? OR 81?? OR 82?? OR 83?? OR 84?? OR 85?? OR 86?? OR 87?? OR 88?? OR 89?? OR 90?? OR 91?? OR 92?? OR 93?? OR 94?? OR 95?? OR 96?? OR 97?? OR 98?? OR 99??))):ab,ti,kw NOT ([Conference Abstract]/lim) AND [English]/lim

Medline

(intracranial aneurysm/ OR (((intracranial* OR cranial* OR cerebral* OR intracerebral* OR brain*) ADJ3 (aneurysm*))).ab,ti,kf.) AND (clip/ OR aneurysm clip/ OR blood vessel clip/ OR clipping/ OR coil embolization/ OR endovascular aneurysm repair/ OR flow diverter/ OR intracranial stent/ OR vascular plug/ OR embolization device/ OR neurovascular embolization device/ OR embolization coil/ OR artificial embolization/ OR (clip OR clips OR clipping OR coil OR coils OR coiling OR (endovascular* ADJ3 (repair* OR therap* OR treat* OR catheter* OR intervention* OR emboli*)) OR stent* OR (flow ADJ3 (diverter* OR diversion*)) OR web-device* OR (woven ADJ3 endobridge) OR (vascular* ADJ3 plug*) OR ((embolization OR embolisation) ADJ3 device*)).ab,ti,kf.) NOT (Case Reports/ OR case-report*.ti.) AND (((2# OR 3# OR 4# OR 5# OR 6# OR 7# OR 8# OR 9# OR 0## OR 1## OR 2## OR 3## OR 4## OR 5## OR 6## OR 7## OR 8## OR 9## OR 1### OR 2### OR 3### OR 4### OR 5### OR 6### OR 7### OR 8### OR 9###) ADJ3 (patients OR patient OR subjects OR individuals OR cases OR persons OR men OR women OR participant* OR people OR children OR adolescent* OR boys OR girls OR teens OR teenagers OR infants OR newborns OR elderly OR survivor* OR specimen* OR sample* OR episode* OR isolate* OR pediatric OR paediatric OR aneurysm*)) OR ((twenty* OR thirty* OR forty* OR fifty* OR sixty* OR seventy* OR eighty* OR ninety* OR hundred* OR thousand*) ADJ6 (patients OR patient OR subjects OR individuals OR cases OR persons OR men OR women OR participant* OR people OR children OR adolescent* OR boys OR girls OR teens OR teenagers OR infants OR newborns OR elderly OR survivor* OR specimen* OR sample* OR episode* OR isolate* OR pediatric OR paediatric OR aneurysm*)) OR ((n OR included OR recruited OR randomized OR randomized OR assigned) ADJ2 (2# OR 3# OR 4# OR 5# OR 6# OR 7# OR 8# OR 9# OR 0## OR 1## OR 2## OR 3## OR 4## OR 5## OR 6## OR 7## OR 8## OR 9## OR 1### OR 2### OR 3### OR 4### OR 5### OR 6### OR 7### OR 8### OR 9###))).ab,ti,kf.

Cochrane CENTRAL registry of trials

((((intracranial* OR cranial* OR cerebral* OR intracerebral* OR brain*) NEAR/3 (aneurysm*))):ab,ti,kw) AND ((clip OR clips OR clipping OR coil OR coils OR coiling OR (endovascular* NEAR/3 (repair* OR therap* OR treat* OR catheter* OR intervention* OR emboli*)) OR stent* OR (flow NEAR/3 (diverter* OR diversion*)) OR web-device* OR (woven NEAR/3

endobridge) OR (vascular* NEAR/3 plug*) OR ((embolization OR embolisation) NEAR/3 device*)):ab,ti,kw) NOT "conference abstract":pt

eAppendix 2. Main Analysis: Sensitivity Analyses and Stratified Results for Effect Modifiers

Sensitivity analysis for safety outcomes, only studies including unruptured aneurysms

IQR= interquartile range, FU= follow-up, NA = not available

Safety conclusion	Positive (Safe)	Uncertain	Negative (Not safe)	P – value (Kruskal-Wallis or Mann-Whitney-U, where applicable)
Number of intervention arms	N = 167	N = 54	N = 2	
	Median (IQR) [range]	Median (IQR) [range]	Median (IQR) [range]	
Total complications (%)	8.3 (5.4 - 15.0) [0.0 - 74.1]	11.5 (9.7 - 17.0) [0.0 - 49.4]	21,8	**0.011**
Poor outcome at discharge (%)	2.1 (0.0 - 4.1) [0.0 - 27.5]	2.5 (1.2 - 3.7) [0.0 - 100]	1,8	0.79
Thromboembolic complications (%)	3.8 (1.0 - 6.8) [0.0 - 74.1]	7.0 (3.2 - 8.7) [0.0 - 22.2]	17,5	**0.012**
In-hospital mortality (%)	0.0 (0.0 - 0.5) [0.0 - 5.6]	0.2 (0.0 - 1.7) [0.0 - 20.0]	1.0 (0.7 - 1.4) [0.4 - 1.7]	**0.009**

Dunn's post-hoc test for in-hospital mortality (p-values)		
Safe vs doubt	Not-safe vs doubt	Not-safe vs safe
**0.019**	0.37	0.21

Sensitivity analysis for effectiveness and durability outcomes with comparable follow-up time

Effectiveness conclusion	Positive (Effective)	Uncertain	Negative (Not effective)	P – value (Kruskal-Wallis or Mann-Whitney-U, where applicable)
Number of intervention arms	N = 167	N = 54	N = 2	
	Median (IQR) [range]	Median (IQR) [range]	Median (IQR) [range]	
Complete occlusion at final FU (%)	81.0 (69.6 - 89.6) [3.0 - 100]	73.6 (64.3 - 81.4) [14.4 - 94.2]	65,7	0.087
Adequate occlusion at final FU (%)	13.0 (7.2 - 24.7) [0 - 89.1]	13.6 (8.5 - 22.2) [0 - 58.5]	NA	0.97

Durability conclusion	Positive (Durable)	Uncertain	Negative (Not durable)	P – value (Kruskal-Wallis or Mann-Whitney-U, where applicable)
Number of intervention arms	N = 167	N = 54	N = 2	
	Median (IQR) [range]	Median (IQR) [range]	Median (IQR) [range]	
Complete occlusion at final FU (%)	82.7 (73.1 - 90.3) [46.0 - 100]	78.0 (72.4 - 88.8) [39.2 - 100]	70,3	0.52
Adequate occlusion at final FU (%)	12.0 (6.4 - 16.0) [0 - 31.0]	13.0 (5.7 - 18.0) [0 - 36.0]	11,6	0.89

© 2023 Volovici V et al. *JAMA Network Open.*

For both studies on effectiveness as well as for studies on durability, the follow-up time was between 9 and 60 months in these studies and was comparable between the 3 groups.

Analyses stratified for rupture status

Studies including only patients with aneurysmal subarachnoid haemorrhage

Safety Conclusion	Positive (Safe)	Uncertain	Negative (Not safe)	P- value (Kruskal-Wallis or Mann-Whitney-U test)
**Number of intervention arms**	**N = 115**	**N = 38**	**N = 1**	
	**Median (IQR) [range]**	**Median (IQR) [range]**	**Median (IQR) [range]**	
**Total complications (%)**	11.5 (6.6 - 17.7) [0.0 - 100.0]	15.6 (10.6 - 22.3) [0.0 - 53.3]	45	**0.028**
**Poor outcome at discharge (%)**	18.2 (12.1 - 30.5) [0.0 - 86.7]	32.5 (23.2 - 46.7) [4.8 - 73.0]	NA	0.42
**Thromboemboli c complication (%)**	4.3 (2.5 - 8.2) [0.0 - 58.0]	6.5 (3.0 - 8.0) [0.0 - 24.1]	NA	0.53
**In-hospital mortality (%)**	4.3 (1.0 - 9.5) [0.0 - 50.9]	4.9 (0.3 - 11.3) [0.0 - 34.4]	18	0.76
**Studies evaluating effectiveness**				
**Effectiveness Conclusion**	**Positive (Effective)**	**Uncertain**	**Negative (Not effective)**	
**Number of intervention arms**	**N = 134**	**N = 22**	**N = 0**	

© 2023 Volovici V et al. *JAMA Network Open.*

	**Median (IQR) [range]**	**Median (IQR) [range]**	**Median (IQR) [range]**	
**Complete occlusion at discharge (%)**	64.0 (43.9 - 80.6) [0.0 - 100]	66.8 (48.1 - 75.7) [13.3 - 100]	NA	0.97
**Complete occlusion at final FU (%)**	81.8 (69.2 - 91.7) [33.0 - 100]	64.2 (58.5 - 68.7) [21.5 - 95.9]	NA	**0.043**
**Adequate occlusion at discharge (%)**	24.1 (14.4 - 33.8) [0.0 - 60.1]	25.6 (15.8 - 30.7) [14.0 - 64.4]	NA	0.81
**Adequate occlusion at final FU (%)**	12.7 (6.1 - 25.8) [0 - 40]	31.4 (29.3 - 39.4) [28 - 58.5]	NA	**0.017**
**Studies evaluating durability**				
**Durability Conclusion**	**Positive (Durable)**	**Uncertain**	**Negative (Not durable)**	
**Number of intervention arms**	**N = 10**	**N = 7**	**N = 0**	
	**Median (IQR) [range]**	**Median (IQR) [range]**	**Median (IQR) [range]**	
**Complete occlusion at final FU (%)**	NA	50.2 (42.6 - 57.8) [35.0 - 65.4]	NA	NA
**FU time (months)**	7.5 (6.8 - 36.1) [6.1 - 64.7]	11.6 (7.0 - 12.0) [7.0 - 29.0]	NA	1

© 2023 Volovici V et al. *JAMA Network Open.*

Studies only including patients with unruptured aneurysms

Safety Conclusion	Positive (Safe)	Uncertain	Negative (Not safe)	P- value (Kruskal-Wallis or Mann-Whitney-U test)
**Number of intervention arms**	**N = 167**	**N = 54**	**N = 2**	
	**Median (IQR) [range]**	**Median (IQR) [range]**	**Median (IQR) [range]**	
**Total complications (%)**	8.3 (5.4 - 15.0) [0.0 - 74.1]	11.5 (9.7 - 17.0) [0.0 - 49.4]	21,8	**0.011**
**Poor outcome at discharge (%)**	2.1 (0.0 - 4.1) [0.0 - 27.5]	2.5 (1.2 - 3.7) [0.0 - 100]	1,8	0.79
**Thromboembolic complication (%)**	3.8 (1.0 - 6.8) [0.0 - 74.1]	7.0 (3.2 - 8.7) [0.0 - 22.2]	17,5	**0.012**
**In-hospital mortality (%)**	0.0 (0.0 - 0.5) [0.0 - 5.6]	0.2 (0.0 - 1.7) [0.0 - 20.0]	1.0 (0.7 - 1.4) [0.4 - 1.7]	**0.0085**
**Studies evaluating effectiveness**				
**Effectiveness Conclusion**	**Positive (Effective)**	**Uncertain**	**Negative (Not effective)**	
**Number of intervention arms**	**N = 155**	**N = 37**	**N = 0**	
	**Median (IQR) [range]**	**Median (IQR) [range]**	**Median (IQR) [range]**	
**Complete occlusion at discharge (%)**	62.6 (39.9 - 82.2) [0 - 100]	61.0 (45.6 - 74.1) [0.5 - 100]	NA	0.43

© 2023 Volovici V et al. *JAMA Network Open.*

**Complete occlusion at final FU (%)**	80 (72.2 - 88.3) [36.8 - 100]	75.7 (62.4 - 85.2) [3.5 - 94.0]	NA	0.25
**Adequate occlusion at discharge (%)**	19.4 (10.4 - 32.6) [0 - 83]	21.0 (14.1 - 27.5) [5.7 - 40.9]	NA	0.96
**Adequate occlusion at final FU (%)**	12.0 (7.6 - 17.3) [0 - 89]	10.9 (5.6 - 27.6) [0 - 69.0]	NA	1
**Studies evaluating durability**				
**Durability Conclusion**	**Positive (Durable)**	**Uncertain**	**Negative (Not durable)**	
**Number of intervention arms**	**N = 20**	**N = 15**	**N = 0**	
	**Median (IQR) [range]**	**Median (IQR) [range]**	**Median (IQR) [range]**	
**Complete occlusion at final FU (%)**	86.2 (76.0 - 90.0) [68.6 - 98.4]	78.0 (76.0 - 87.3) [72.7 - 97.9]	NA	0.53
**FU time (months)**	36.0 (19.5 - 46.2) [10.9 - 132]	12.0 (6.4 - 15.4) [6 - 101]	NA	**0.0077**

© 2023 Volovici V et al. *JAMA Network Open.*

Studies including a mix of both patients with a subarachnoid haemorrhage and with unruptured aneurysms

**Safety Conclusion**	**Positive (Safe)**	**Uncertain**	**Negative (Not safe)**	**P- value (Kruskal-Wallis or Mann-Whitney-U test)**
**Number of intervention arms**	**N = 524**	**N = 140**	**N = 1**	
	**Median (IQR) [range]**	**Median (IQR) [range]**	**Median (IQR) [range]**	
**Total complications (%)**	11.0 (6.2 - 16.0) [0.0 - 80.8]	14.0 (10 - 19.8) [0 - 78.1]	9,3	**< 0.0001**
**Poor outcome at discharge (%)**	7.1 (2.8 - 15.6) [0.0 - 55.2]	14.7 (4.7 - 26.8) [0.0 - 100]	NA	**0.01**
**Thromboembolic complication (%)**	5.0 (2.7 - 8.5) [0.0 - 33.0]	5.7 (3.4 - 9.8) [0 - 35.9]	NA	0.2
**In-hospital mortality (%)**	1.0 (0.0 - 2.8) [0.0 - 25.3]	1.8 (0.0 - 4.4) [0.0 - 26.4]	2.7	**0.008**
**Studies evaluating effectiveness**				
**Effectiveness Conclusion**	**Positive (Effective)**	**Uncertain**	**Negative (Not effective)**	
**Number of intervention arms**	**N = 522**	**N = 131**	**N = 4**	
	**Median (IQR) [range]**	**Median (IQR) [range]**	**Median (IQR) [range]**	
**Complete occlusion at discharge (%)**	59.4 (41.5 - 79.9) [0.0 - 100]	61.8 (38.4 - 78.8) [11.5 - 100]	51.8 (49.4 - 54.3) [46.9 - 56.7]	0.78

© 2023 Volovici V et al. *JAMA Network Open.*

**Complete occlusion at final FU (%)**	78.0 (66.2 - 87.0) [3.0 - 100]	76.3 (62.3 - 88.6) [14.4 - 98.8]	61.9 (59.9 - 63.8) [58.0 - 65.7]	0.29
**Adequate occlusion at discharge (%)**	24.1 (13.3 - 36.8) [0.0 - 96.2]	24.0 (9.5 - 34.7) [0.0 - 94.0]	37.7 (35.6 - 37.7) [37.5 - 37.8]	0.39
**Adequate occlusion at final FU (%)**	14.7 (7.9 - 26.0) [0 - 89.1]	11.2 (7.0 - 19.2) [0 - 48.5]	23	0.2
**Studies evaluating durability**				
**Durability Conclusion**	**Positive (Durable)**	**Uncertain**	**Negative (Not durable)**	
**Number of intervention arms**	**N = 59**	**N = 41**	**N = 6**	
	**Median (IQR) [range]**	**Median (IQR) [range]**	**Median (IQR) [range]**	
**Complete occlusion at final FU (%)**	81.4 (71.9 - 90.4) [42.0 - 100]	76.7 (57.0 - 89.9) [39.2 - 100]	70.3 (64.2 - 72.3) [58.0 - 74.2]	0.16
**FU time (months)**	18.7 (11.9 - 36.3) [5.9 - 98]	13.6 (10.6 - 24.5) [6.0 - 68.4]	20.5 (15.2 - 25.5) [10 - 30]	0.54

© 2023 Volovici V et al. *JAMA Network Open.*

Analyses stratified for treatment modality

Endovascular treatment

Safety Conclusion	Positive (Safe)	Uncertain	Negative (Not safe)	P- value (Kruskal-Wallis or Mann-Whitney-U test)
**Number of intervention arms**	**N = 686**	**N = 188**	**N = 4**	
	**Median (IQR) [range]**	**Median (IQR) [range]**	**Median (IQR) [range]**	
**Total complications (%)**	10.2 (5.9 - 15.1) [0 - 74.6]	12.6 (10.0 - 17.9) [0 - 78.1]	21.8 (15.6 - 33.4) [9.3 - 45]	**< 0.0001**
**Poor outcome at discharge (%)**	6.8 (2.4 - 15.3) [0 - 67.0]	5.4 (2.1 - 22.7) [0 - 73.0]	1,8	0.79
**Thromboemboli c complication (%)**	4.8 (2.6 - 8.2) [0 - 74.1]	6.0 (3.5 - 9.1) [0 - 29.6]	17.5	**0.03**
**In-hospital mortality (%)**	0.7 (0 - 2.8) [0 - 29]	1.6 (0 - 4.1) [0 - 34.4]	2.2 (1.4 - 6.5) [0.4 - 18]	**0.005**
**Studies evaluating effectiveness**				
**Effectiveness Conclusion**	**Positive (Effective)**	**Uncertain**	**Negative (Not effective)**	
**Number of intervention arms**	**N = 717**	**N = 159**	**N = 4**	
	**Median (IQR) [range]**	**Median (IQR) [range]**	**Median (IQR) [range]**	

© 2023 Volovici V et al. *JAMA Network Open.*

**Complete occlusion at discharge (%)**	58.1 (38.1 - 75.0) [0 - 100]	59.1 (38.0 - 73.7) [0.5 - 96.7]	51.8 (49.4 - 54.3) [46.9 - 56.7]	0.9
**Complete occlusion at final FU (%)**	77.8 (67.0 - 85.8) [3.0 - 100]	74.1 (61.0 - 83.9) [3.5 - 98.8]	61.9 (59.9 - 63.8) [58 - 65.7]	0.06
**Adequate occlusion at discharge (%)**	24.9 (14.6 - 36.6) [0 - 96.2]	25.0 (15.0 - 34.3) [1.6 - 94.0]	37.7 (37.6 - 37.7) [37.5 - 37.8]	0.39
**Adequate occlusion at final FU (%)**	14.0 (7.9 - 25.1) [0 - 89.1]	13.0 (7.7 - 26.9) [0 - 69]	23	0.91
**Studies evaluating durability**				
**Durability Conclusion**	**Positive (Durable)**	**Uncertain**	**Negative (Not durable)**	
**Number of intervention arms**	**N = 69**	**N = 57**	**N = 6**	
	**Median (IQR) [range]**	**Median (IQR) [range]**	**Median (IQR) [range]**	
**Complete occlusion at final FU (%)**	81.0 (71.4 - 87.7) [42.0 - 100]	76.7 (64.0 - 84.5) [35 - 100]	70.3 (64.2 - 72.3) [58.0 - 74.2]	0.2
**FU time (months)**	18.3 (12.0 - 29.2) [5.9 - 63.0]	12.4 (9.2 - 23.9) [6 - 101]	20.5 (15.2 - 25.5) [10 - 30]	0.3

© 2023 Volovici V et al. *JAMA Network Open.*

Microsurgical treatment

Safety Conclusion	Positive (Safe)	Uncertain	Negative (Not safe)	P- value (Kruskal-Wallis or Mann-Whitney-U test)
**Number of intervention arms**	**N = 122**	**N = 46**	**N = 0**	
	**Median (IQR) [range]**	**Median (IQR) [range]**	**Median (IQR) [range]**	
**Total complications (%)**	14.4 (7.7 - 20.6) [0 - 100]	19.0 (12.8 - 26.1) [0 - 53.3]	NA	**0.009**
**Poor outcome at discharge (%)**	12.2 (2.9 - 26.0) [0 - 86.7]	24.7 (16.0 - 46.7) [0 - 100]	NA	**0.014**
**Thromboembolic complication (%)**	2.2 (0 - 6.3) [0 - 58]	4.0 (0 - 8.7) [0 - 35.9]	NA	0.22
**In-hospital mortality (%)**	1.1 (0 - 5.0) [0 - 50.9]	1.4 (0 - 7.8) [0 - 27]	NA	0.48
**Studies evaluating effectiveness**				
**Effectiveness Conclusion**	**Positive (Effective)**	**Uncertain**	**Negative (Not effective)**	
**Number of intervention arms**	**N = 109**	**N = 36**	**N = 0**	
	**Median (IQR) [range]**	**Median (IQR) [range]**	**Median (IQR) [range]**	
**Complete occlusion at discharge (%)**	92.2 (87.1 - 99.1) [13 - 100]	93.0 (87.7 - 98.5) [58.8 - 100]	NA	0.95

© 2023 Volovici V et al. *JAMA Network Open.*

**Complete occlusion at final FU (%)**	93.6 (88.6 - 100) [33.3 - 100]	94.1 (92.7 - 96.4) [86.7 - 98.4]	NA	0.93
**Adequate occlusion at discharge (%)**	7.8 (2.5 - 13.1) [0 - 36.4]	5.4 (3.2 - 9.3) [0 - 27]	NA	0.75
**Adequate occlusion at final FU (%)**	6.8 (1.5 - 9.4) [0 - 47.7]	4.1 (0 - 4.3) [0 - 11.7]	NA	0.32
**Studies evaluating durability**				
**Durability Conclusion**	**Positive (Durable)**	**Uncertain**	**Negative (Not durable)**	
**Number of intervention arms**	**N = 25**	**N = 7**	**N = 0**	
	**Median (IQR) [range]**	**Median (IQR) [range]**	**Median (IQR) [range]**	
**Complete occlusion at final FU (%)**	90.5 (89.2 - 98.1) [88.5 - 100]	96.0 (95.0 - 96.9) [94.0 - 97.9]	NA	0.73
**FU time (months)**	49.2 (37.5 - 63.0) [6 - 132]	14.6 (10.9 - 30.0) [7.4 - 68.4]	NA	0.32

© 2023 Volovici V et al. *JAMA Network Open.*

eAppendix 3. Frequency of Essential Domain-Specific Outcomes Not Reported in the Included Studies

Safety

Safety conclusion	**Positive (Safe)**	**Uncertain**	**Negative (Not Safe)**
Number of intervention arms	**N = 821**	**N = 235**	**N = 4**
	% of studies not reporting outcome	% of studies not reporting outcome	% of studies not reporting outcome
Mortality at discharge	28%	25%	0%
Mortality at final FU	41%	51%	0%
Poor outcome discharge	66%	68%	75%
Poor outcome at final FU	56%	69%	50%
Total complications	24%	29%	25%
Thromboembolic complications	34%	40%	75%

Effectiveness

Effectiveness conclusion	**Positive (Effective)**	**Uncertain**	**Negative (Not Effective)**
Number of intervention arms	**N = 826**	**N = 195**	**N = 4**
	% of studies not reporting outcome	% of studies not reporting outcome	% of studies not reporting outcome
Complete occlusion at discharge (%)	41%	41%	50%
Complete occlusion at final FU (%)	42%	53%	50%
Adequate occlusion at discharge (%)	51%	50%	50%
Adequate occlusion at final FU (%)	56%	64%	75%

Durability

Durability conclusion	**Positive (Durable)**	**Uncertain**	**Negative (Not Durable)**
Number of intervention arms	**N = 94**	**N = 64**	**N = 6**
	% of studies not reporting outcome	% of studies not reporting outcome	% of studies not reporting outcome
Complete occlusion at final FU (%)	36%	55%	50%

© 2023 Volovici V et al. *JAMA Network Open.*

Adequate occlusion at final FU (%)	49%	47%	67%
Attrition rate	27%	22%	33%
FU time	27%	22%	33%

© 2023 Volovici V et al. *JAMA Network Open.*

eAppendix 4. Sensitivity Analysis Including Only Studies With More Than 100 Patients

Main analysis

Safety Conclusion	Positive (Safe)	Uncertain	Negative (Not safe)	P- value (Kruskal-Wallis or Mann-Whitney-U test)
Number of intervention arms	N = 255	N = 86	N = 4	
	Median (IQR) [range]	Median (IQR) [range]	Median (IQR) [range]	
Total complications (%)	9.3 (6.2 - 15.7) [0.0 - 43.9]	11.8 (9.9 - 6.1) [2.3 - 49.4]	21.8 (15.6 - 33.4) [9.3 - 45]	**0.002**
Poor outcome at discharge (%)	8.1 (2.8 - 19.0) [0 - 86.7]	13.8 (2.2 - 26.0) [0 - 100]	1.8	0.63
Thromboembolic complication (%)	4.6 (2.8 - 6.7) [0 - 58.0]	4.9 (3.7 - 6.9) [0 - 16.5]	17.5	0.56
In-hospital mortality (%)	1.0 (0.0 - 3.0) [0.0 - 50.9]	1.6 (0.3 - 3.9) [0 - 29.4]	2.2 (1.4 - 6.5) [0.4 - 18.0]	0.26
**Studies evaluating effectiveness**				
Effectiveness Conclusion	Positive (Effective)	Uncertain	Negative (Not effective)	P – value (Mann-Whitney-U test)
Number of intervention arms	N = 242	N = 69	N = 0	
	Median (IQR) [range]	Median (IQR) [range]	Median (IQR) [range]	

© 2023 Volovici V et al. *JAMA Network Open.*

**Complete occlusion at discharge (%)**	62.9 (45.9 - 80) [0 - 100]	63.3 (46.0 - 76.0) [15.7 - 98]	NA	0.97
**Complete occlusion at final FU (%)**	78.4 (65.9 - 85.2) [3 - 100]	75.0 (64.8 - 88.5) [14.4 - 97.7]	NA	0.84
**Adequate occlusion at discharge (%)**	22.8 (13.9 - 35.4) [0 - 96.2]	24.7 (17.0 - 34.0) [1.6 - 67.7]	NA	0.94
**Adequate occlusion at final FU (%)**	13.0 (7.8 - 24.8) [0 - 89.1]	14.2 (9.2 - 21.9) [3.9 - 42.1]	NA	0.62
**Studies evaluating durability**				
**Durability Conclusion**	**Positive (Durable)**	**Uncertain**	**Negative (Not durable)**	**P – value (Mann-Whitney-U test)**
**Number of intervention arms**	**N = 37**	**N = 21**	**N = 1**	
	**Median (IQR) [range]**	**Median (IQR) [range]**	**Median (IQR) [range]**	
**Complete occlusion at final FU (%)**	81.5 (70.2 - 90.3) [46.0 - 100]	76.8 (58.5 - 80.6) [39.2 - 94]	70.3	0.12
**FU time (months)**	24.5 (10.1 - 38.2) [5.9 - 132]	12.0 (8.0 - 22.5) [6.5 - 68.4]	10	0.26

Sensitivity analysis for safety outcomes, only studies including unruptured aneurysms

Safety conclusion	Positive (Safe)	Uncertain	Negative (Not safe)	P – value (Kruskal-Wallis or Mann-

© 2023 Volovici V et al. *JAMA Network Open.*

Number of intervention arms	N = 61	N = 21	N = 2	Whitney-U, where applicable)
	Median (IQR) [range]	Median (IQR) [range]	Median (IQR) [range]	
Total complications (%)	6.9 (4.9 - 9.7) [1.0 - 27.0]	10.3 (9.5 - 12.2) [5.0 - 49.4]	21,8	**0.003**
Poor outcome at discharge (%)	1.9 (1.0 - 3.5) [0.0 - 11.1]	2.3 (1.6 - 2.8) [0 - 100]	1,8	0.72
Thromboembolic complications (%)	4.1 (1.5 - 6.2) [0 - 17]	6.3 (4.6 - 7.8) [0 - 16.5]	17,5	0.06
In-hospital mortality (%)	0.0 (0.0 - 0.7) [0.0 - 3.1]	0.5 (0.0 - 1.9) [0.0 - 20.0]	1.0 (0.7 - 1.4) [0.4 - 1.7]	0.07

Sensitivity analysis for durability outcomes with comparable follow-up time

**Studies evaluating durability**				
**Durability Conclusion**	**Positive (Durable)**	**Uncertain**	**Negative (Not durable)**	**P- value (Kruskal-Wallis or Mann-Whitney-U test)**
**Number of intervention arms**	**N = 32**	**N = 17**	**N = 3**	
	**Median (IQR) [range]**	**Median (IQR) [range]**	**Median (IQR) [range]**	
**Complete occlusion at final FU (%)**	82.7 (73.1 - 90.3) [46.0 - 100]	78.0 (72.4 - 88.8) [39.2 - 100]	70.3	0.54
**FU time (months)**	24.5 (16.8 - 36) [12 - 60]	20 (12 - 29) [9 - 50]	24.0 (17 - 27) [10 - 30]	0.17

© 2023 Volovici V et al. *JAMA Network Open.*

eAppendix 5. Regression Results

Variables associated with a positive safety conclusion

N= 457 studies, multivariable model

Covariate	p-value	aOR (9% CI) or Beta and standard error
Intervention = Stent- assisted coiling	**0.04**	1.9 (1.1 - 3.8)
Total complications (%)	**0.01**	Beta: -0.027, S.E.: 0.01
Mortality final FU	0.25	1.0 (0.9 - 1.0)
New technique	0.24	1.6 (0.8 - 3.6)

Variables associated with a positive effectiveness conclusion

N=573 studies, multivariable model

Covariate	p-value	aOR (9% CI) or Beta and standard error
Stent-assisted coiling	**0.008**	2.3 (1.3 - 4.3)
Flow diverter	0.052	1.7 (1.0 - 2.9)
Complete occlusion at final FU	**0.02**	Beta: 0.013, Standard Error: 0.006

The regression analyses were not run for durability as the dependent variable as the level of missing data did not allow is, making the models laden with sparse data bias.

© 2023 Volovici V et al. *JAMA Network Open.*

eAppendix 6. Correlations and Cross-Tables

Association between Conflict of Interest and the assessed device

		Intervention						
		Coil	Clip	FD	SAC	Endo other	Micro other	Total
Conflict of Interest	Yes	60 (10.8%)	22 (6.7%)	88 (33.1%)	66 (21.5%)	50 (24.9%)	3 (17.6%)	289
	None	205 (36.9%)	180 (54.9%)	124 (46.6%)	170 (55.4%)	91 (45.3%)	8 (47.1%)	778
	Not reported	290 (52.2%)	126 (38.4%)	54 (20.3%)	71 (23.1%)	60 (29.9%)	6 (35.3%)	607
		555 (33.2%)	328 (19.5%)	266 (15.9%)	307 (18.3%)	201 (12.0%)	17 (1.0%)	1674

P < 0.0001, chi-square test

Studies reporting a new technique for their center and asking for informed consent

		New technique		
		No	Yes	Total
Informed consent	Not reported	930 (73.6%)	172 (68.8%)	1102
	Yes	334 (26.2%)	79 (31.2%)	413
		1264 (83.3%)	251 (16.7%)	1515

P = 0.13

Conflict of Interest in relation to safety assessment

		Conflict of Interest			
		Yes	None	Not mentioned	Total
Conclusion	Safe	173 (81.2%)	388 (78.7%)	260 (73.4%)	821
	Uncertain	39 (18.3%)	104 (21.1%)	92 (26.0%)	235
	Not-safe	1 (0.5%)	1 (0.02%)	2 (0.6%)	4
		213 (20.1%)	493 (46.5%)	354 (33.4%)	1060

P = 0.20

Conflict of Interest in relation to efficacy assessment

		Conflict of Interest			
		Yes	None	Not mentioned	
Conclusion	Effective	173 (85.2%)	391 (81.3%)	262 (76.8%)	826
	Uncertain	30 (14.8%)	88 (18.3%)	77 (22.6%)	195
	Not-effective	0 (0%)	2 (0.4%)	2 (0.6%)	4
		203 (19.8%)	481 (46.9%)	341 (33.3%)	1025

P = 0.16

**Group**	**Outcome**	**Rho**	**p-value**
Endovascular / aSAH	Complete occlusion final FU	0,41	**0.0001**
Microsurgical / aSAH	Complete occlusion final FU	0,47	0.24

© 2023 Volovici V et al. *JAMA Network Open.*

Endovascular / Elective	Complete occlusion final FU	0,3	**0.0004**
Microsurgical / Elective	Complete occlusion final FU	-0,09	0.7
Endovascular / Both	Complete occlusion final FU	0,14	**0.003**
Microsurgical / Both	Complete occlusion final FU	-0,2	0.23

Correlations between the year the study was published and the proportion of complete occlusion at final follow-up, stratified for ruptured status and treatment modality.

Group	Outcome	Rho	p-value
Endo / SAH	Total complications	0,055	0.52
Micro / SAH	Total complications	0,017	0.92
Endo / Elective	Total complications	-0,06	0.4
Micro / Elective	Total complications	-0,17	0.21
Endo / Both	Total complications	-0,05	0.19
Micro / Both	Total complications	-0,07	0.54

Correlations between the year the study was published and the incidence of total complications, stratified for ruptured status and treatment modality.

Group	Outcome	Rho	p-value
Endo / SAH	In-hospital mortality	-0,02	0.77
Micro / SAH	In-hospital mortality	0,06	0.65
Endo / Elective	In-hospital mortality	-0,198	**0.009**
Micro / Elective	In-hospital mortality	-0,29	**0.02**
Endo / Both	In-hospital mortality	-0,17	**0.0001**
Micro / Both	In-hospital mortality	-0,16	0.16

Correlations between the year the study was published and the incidence of in-hospital mortality, stratified for ruptured status and treatment modality.

Table. Definitions of effectiveness (n=2)

First author, year	Study design	Rupture status	Intervention	FU period (months)	Predefined in protocol	Definition of effectiveness
Arthur, 2019	PO	Both aSAH and elective	WEB	12	Yes	The proportion of patients with complete

© 2023 Volovici V et al. *JAMA Network Open.*

						aneurysm occlusion without re-treatment, recurrent subarachnoid hemorrhage, or significant parent artery stenosis (defined as >50% stenosis) at 1 year after treatment.
McDougall, 2022	PO	Elective	FD	12	Yes	The proportion of subjects with complete occlusion of the target aneurysm and ≤50% stenosis of the parent artery at the target intracranial aneurysm at 12 months after treatment as assessed by angiography, and without re-treatment of the target intracranial aneurysm within 1 year post-FRED placement. Any re-treatment was considered an endpoint failure for effectiveness.

eAppendix 7. Supplementary Figures

The relationship between the conclusions of the study on safety and the incidence of in-hospital mortality and poor functional outcome. The absolute incidence of in-hospital mortality/functional outcome as well as their confidence intervals, calculated with the Clopper-Pearson method, are presented for each study, stratified for treatment modality. Red lines indicate a study with a negative conclusion (not safe), green lines a study with a positive conclusion (safe), blue lines indicate studies with an uncertain conclusion. It would be expected that the lower half would include preponderantly green studies and the upper half progressively more blue and red studies. In IA scientific literature, this is not the case.

© 2023 Volovici V et al. *JAMA Network Open.*

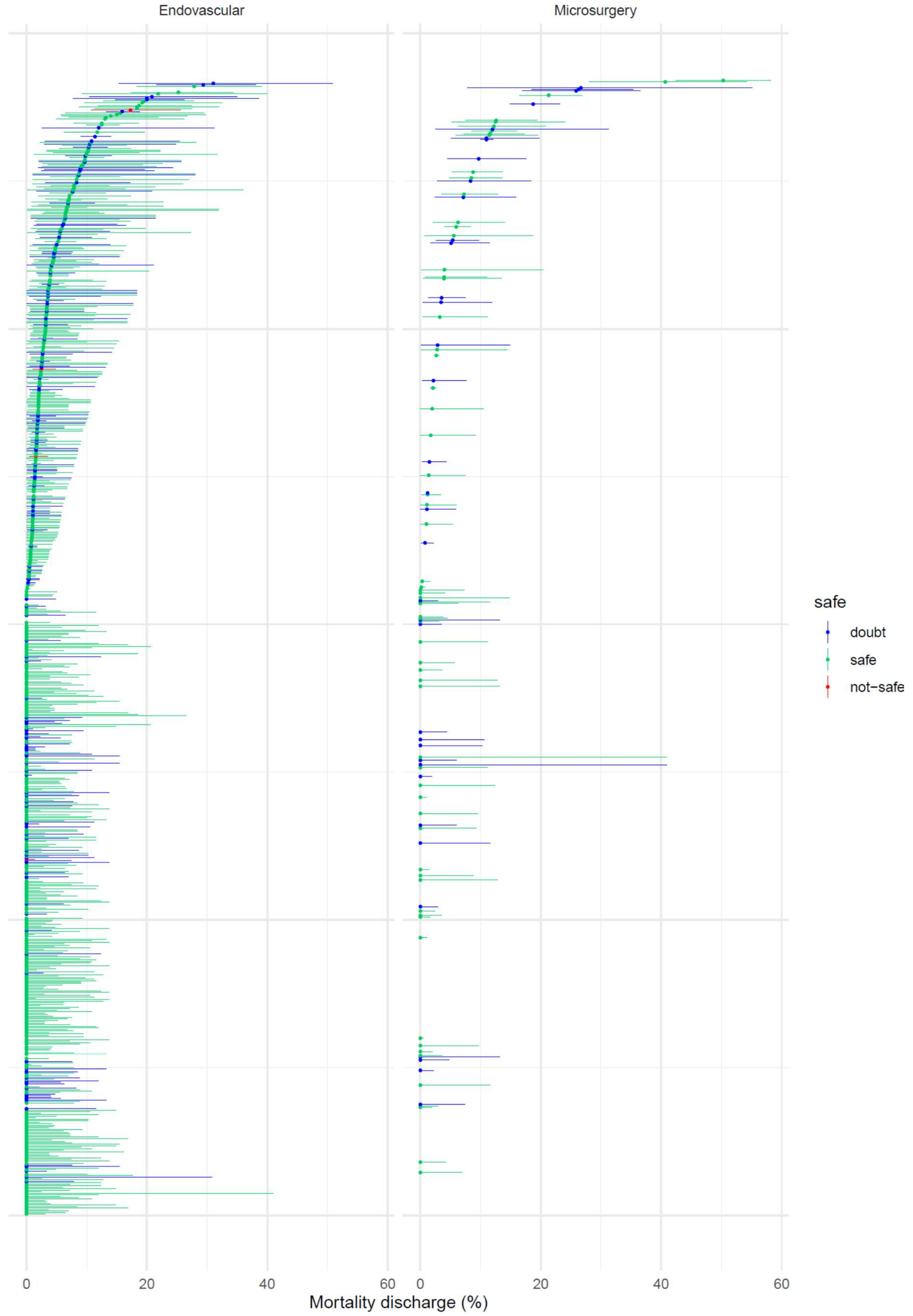

© 2023 Volovici V et al. *JAMA Network Open.*

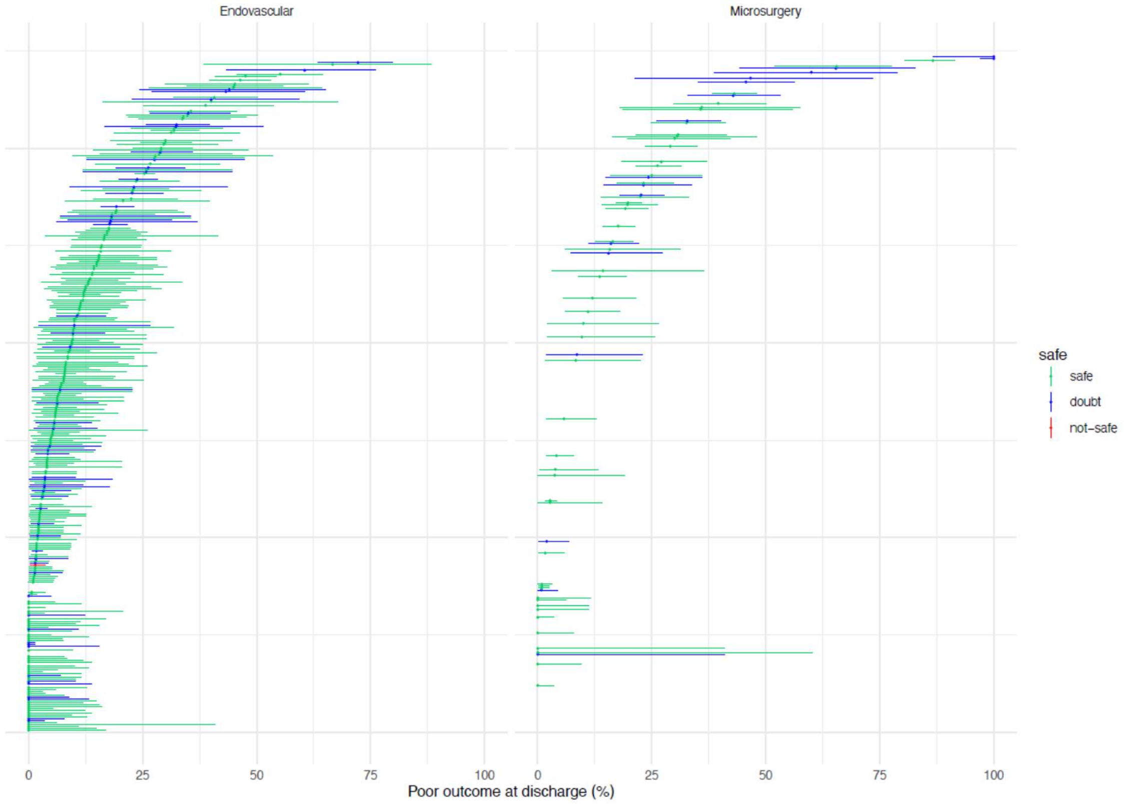

© 2023 Volovici V et al. *JAMA Network Open.*

eAppendix 8. Statistical Analysis Plan and Data Dictionary

Statistical analysis plan and data dictionary

Claims of safety, effectiveness, and durability in intracranial aneurysm research

Victor Volovici, MD, PhD; Iris S. C. Verploegh, MD; Torstein R. Meling, MD, PhD

Study Coordinators: Victor Volovici, MD, PhD; Torstein R. Meling, MD, PhD

Responsible Methodologist: Victor Volovici, MD, PhD

Table of Contents

1. Version History
2. Included variables and data dictionary. Definitions of domains and domain-specific outcomes. Definitions of effect modifiers
3. Domain-specific outcomes
4. Regression and correlation analyses
5. Sensitivity analyses

1. Version History

July 1st, 2020: First version

October 30th, 2021: First update

- Added regression analyses
- Added sensitivity analyses

Otober 1st, 2022: Final update

- Added correlation analyses

2. Included variables and data dictionary. Definitions

2.1. Study characteristics and design

Number of patients;

Number of aneurysms;

Country;

© 2023 Volovici V et al. *JAMA Network Open.*

Number of centres;

Number of countries;

Year of publication;

Year start inclusion of patients;

Year end inclusion of patients;

Study design: Retrospective Observational; Prospective Observational; Randomized Controlled Trial (RCT) - Any subgroup analysis report of an RCT, as well as any periprocedural outcomes or long-term follow-up will be considered in the category RCT due to the paucity of such available evidence in IA treatment;

Confounder adjustment- Any statistical technique used to account for confounding in the data and leading to adjusted effect estimates being reported in the article;

Anterior circulation – Percentage of aneurysms located in the anterior circulation of the total sample;

Informed consent – This variable will record informed consent was obtained from the patients included in the study;

New technique – A study or intervention arm is defined as describing a new technique when the intervention was applied for the first time in the centre(s) participating in the study, and previous scientific evidence supporting the use of the technique was scarce (no studies supporting effectiveness available before the inclusion of the first patient);

Conflict of interest – This variable will be recorded as "present" when there was a conflict of interest belonging to one of the authors to one of the devices researched in the paper;

Funding – This variable will be recorded as "present" when any type of funding was obtained to support the study.

Effect modifiers

Rupture status- Whether the study or intervention arm included patients with ruptured or unruptured aneurysms only;

Treatment – Whether patients in the study or arm were treated by endovascular, microsurgical means or a combination of techniques
Device – The device used as the intervention in the study. These are categorized as "coil", "stent-assisted coiling", "balloon-assisted coiling", "flow diverter", "WEB", "clip" or "other", which includes any type of combination.

2.2. Study results

The following variables may be recorded as a number or as "not reported". Missing values are used for cases in which the data reported is not comparable to the other reports on the topic, as defined below.

Procedure successfully finalized – This variable will record the percentage of patients in which the procedure was successfully finalised, not having to be aborted due to technical reasons;

Complications

Total complications – The percentage of total complications, including all of the categories listed below. When the study report appears to include other types of categories of complications that cannot be categorised in the types below, the paper will be discussed in the bimonthly meetings with the senior authors. If the true result cannot be recalculated, the data will be recorded missing;

Thromboembolic complications – The percentage of thromboembolic complications requiring treatment or leading to subclinical or symptomatic ischemia. If the definition used in the paper is different, the paper will be discussed in the bimonthly meetings with the senior authors. If the percentage cannot be recalculated or the definition does not fit, the data will be recorded missing.

Ischemia (symptomatic) – The percentage of patients with new-onset or worsening neurological status due to ischemia as a direct result of the procedure. If definitions differ in the included papers, the results will be discussed in bimonthly meetings with the senior authors. If the results cannot be recalculated to make them comparable to the rest of the studies, or if the definition does not fit, the data will be recorded missing.

Ischemia (subclinical) - The percentage of patients with ischemia on cerebral imaging as a direct result of the procedure, without or new-onset worsening neurological status. If definitions differ in the included papers, the results will be discussed in bimonthly meetings with the senior authors. If the results cannot be recalculated to

make them comparable to the rest of the studies, or if the definition does not fit, the data will be recorded missing.

Technical complications – The percentage of patients undergoing procedures during which a technical complications, such as a failure to deploy a stent, or an inadvertently detached coil, occurred. If definitions differ in the included papers, the results will be discussed in bimonthly meetings with the senior authors. If the results cannot be recalculated to make them comparable to the rest of the studies, or if the definition does not fit, the data will be recorded missing.

Peri-procedural rupture – The percentage patients undergoing procedures during which a rupture of either the aneurysm or a vessel occurred. If definitions differ in the included papers, the results will be discussed in bimonthly meetings with the senior authors. If the results cannot be recalculated to make them comparable to the rest of the studies, or if the definition does not fit, the data will be recorded missing.

Clinical outcomes

In-hospital mortality – The percentage of patients who died during hospitalization after having undergone the intervention. If definitions differ in the included papers, the results will be discussed in bimonthly meetings with the senior authors. If the results cannot be recalculated to make them comparable to the rest of the studies, or if the definition does not fit, the data will be recorded missing.

Mortality at final follow-up – The percentage of patients who were dead at final follow-up having undergone the intervention. If definitions differ in the included papers, the results will be discussed in bimonthly meetings with the senior authors. If the results cannot be recalculated to make them comparable to the rest of the studies, or if the definition does not fit, the data will be recorded missing.

Poor functional outcome at discharge and at final follow-up (2 separate variables) – Poor functional outcome is defined as the modified Rankin Scale (mRS) scores 3-6 and Glasgow Outcome Score (GOS) 1-3. This variable records the percentage of patients with poor functional outcome at discharge. If definitions of functional outcome or scales used differ in the included papers, the results will be discussed in bimonthly meetings with the senior authors. If the results cannot be recalculated to make them comparable to the rest of the studies, or if the definition does not fit, the data will be recorded missing.

Follow-up data

Number of patients with clinical follow-up available at discharge and at final follow-up (2 separate variables) – The total number of patients who had undergone clinical assessment of functional status at discharge and final follow-up. If definitions differ in the included papers, the results will be discussed in bimonthly meetings with the senior authors. If the results cannot be recalculated to make them comparable to the rest of the studies, or if the definition does not fit, the data will be recorded missing.

© 2023 Volovici V et al. *JAMA Network Open.*

Duration of follow-up in months – The duration of total follow-up for a particular study. The follow-up duration concerns the extracted scientific article, not any subsequent papers that may have been published after with longer-term results.

Number of patients with anatomical follow-up available at discharge and at final follow-up (2 separate variables) - The total number of patients who had undergone cranial vascular imaging to determine the degree of aneurysm occlusion at discharge and final follow-up. If definitions differ in the included papers, the results will be discussed in bimonthly meetings with the senior authors. If the results cannot be recalculated to make them comparable to the rest of the studies, or if the definition does not fit, the data will be recorded missing.

Effectiveness outcomes

Complete occlusion at discharge and Complete occlusion at final follow-up – The percentage of patients with an aneurysm that was completely excluded from circulation, as evaluated by cranial vascular imaging, at discharge and final follow-up. If definitions differ in the included papers, the results will be discussed in bimonthly meetings with the senior authors. If the results cannot be recalculated to make them comparable to the rest of the studies, or if the definition does not fit, the data will be recorded missing.

Adequate occlusion at discharge and Adequate occlusion at final follow-up – The percentage of patients with an aneurysm that was incompletely occluded at discharge or final follow-up according to the Raymond-Roy classification. In this category will be included any modification of the Raymond-Roy classification which suggest an “almost” 100% occlusion, e.g. “near total”; “near complete”; “almost complete” etc. When the definitions are unclear or the categories used vague, the results will be discussed in bimonthly meetings with the senior authors. If the results cannot be recalculated to make them comparable to the rest of the studies, or if the definition does not fit, the data will be recorded missing.

Definitions

Definition of safe/effective/durable (3 variables) - We will extract whether a study has a definition of either “safety”, “effectiveness” or “durability” in the methods. We will also extract whether these definition were pre-specified in the protocol and whether the definitions in the protocol and the main text are the same. Only definitions on a group-level will be considered as true definitions. Definitions of effectiveness or durability at the individual aneurysm level will be excluded.

Assessment of the conclusion by the neuro-linguist

A professional, academic neuro-linguist, blinded for the type of study (endovascular or microsurgical), with no extensive knowledge of the field of IA or any involvement in IA research will assess the wording of the conclusions of the included studies. Lexical fields will be built for purpose for the 3 domains “safe”, “effective” and “durable”. Conclusions will be classified for each of the three domains separately. A “positive” conclusion means the technique was described to be either safe, effective or durable in the conclusion without any caveats whatsoever.

“uncertain” means wording was chosen to indicate that a technique might be safe, effective or durable but with certain caveats or only under certain circumstances and

“negative” means a technique was described as being not safe, effective or durable.

3. Domain-specific outcomes

In concordance with the best practice in aSAH research, we defined domain-specific outcomes, for safety, effectiveness and durability separately, which constitute the minimum essential outcomes that should be reported in order to substantiate claims of safety, effectiveness or durability.

Safety

The proportions of:

In-hospital mortality

Poor functional outcome at discharge

Total complications

Thrombo-embolic complications

Effectiveness

The proportions of:

Complete occlusion at discharge and at final follow-up

Adequate occlusion at discharge and at final follow-up will be seen as a secondary outcomes, as the objective of the treatment is the complete exclusion of the aneurysmal sac from circulation

Durability

The proportion of:

Complete occlusion at final follow-up

© 2023 Volovici V et al. *JAMA Network Open.*

The main analysis consists of a comparison of the distribution of incidences of domain-specific outcomes between studies with a positive, uncertain or negative conclusion.

The null hypothesis is that these samples (from studies with positive, uncertain and negative conclusions) all originate from the same distribution. As we do not expect a normal distribution, we will use the Kruskal-Wallis test, or the Mann-Whitney-U test if only 2 groups (positive, uncertain or negative) are available. The significance level is set at a two-tailed alpha of 0.05. If one of the groups has little to no data, we will use the Mann-Whitney-U test to compare the distributions of the other two groups.

When a statistically significant association is detected, a Dunn's post-hoc test will be performed to pinpoint which comparisons are statistically significant (positive versus uncertain, uncertain versus negative, positive versus negative).

Essential considerations

The main analyses will be reported stratified according to the effect modifiers defined in the data dictionary, rupture status and treatment. The main analysis will include the comparisons for safety-specific outcomes for studies reporting unruptured (elective) aneurysms only. Subarachnoid haemorrhage may confound outcomes to such an extent, that only reporting a pooled analysis may not be sufficiently informative.

Similarly, for effectiveness and durability outcomes, complete occlusion at final follow-up is dependent on follow-up time for some endovascular techniques. Therefore, we will apply formal statistical tests to check whether the follow-up period is comparable between studies with a positive, uncertain and negative conclusion. If this is not the case, we will select a sub-set of studies with follow-up times above 9 months that maximizes the number of studies while keeping the distribution of follow-up time comparable between groups. This analysis will be reported as a sensitivity analysis alongside the main analysis.

4. Regression and correlation analyses

We will be interested in assessing which variables are associated with a higher likelihood of claiming safety, effectiveness and durability. We will use hierarchical mixed effects regression models. We will include in the models variables that concern the design of the study, such as the number of patients included, the type of study design, but also the device used, whether a conflict of interest was present, whether a new technique was employed and the reported incidence of domain-specific outcomes. Variables with a p-value under 0.2 in the univariable models will be included included in the multivariable models, and restricted cubic splines will be used when necessary to relax the linearity assumption for variables that are non-linearly associated with the outcomes. This evaluation will be done by visual inspection of plots between covariate and outcome.

© 2023 Volovici V et al. *JAMA Network Open.*

If missing data is prevalent and there is a risk of sparse data bias, we will attempt to include the maximum number of studies that report the most important variables as evaluated by the multivariable models. We will also use the Akaike Information Criterion and R^2 metrics to guide the best choice for the models.

Correlation analyses

We are interested to test the hypothesis that perhaps safety and effectiveness outcomes have changes with time. Therefore, we will perform correlation analyses using Spearman's correlation between the year of publication and the incidence of mortality, poor functional outcome and the proportion of complete occlusion. These analyses will also be stratified for the pre-defined effect modifiers.

Conflict of interest and informed consent

We will formally test the hypothesis that having a conflict of interest is associated with the likelihood of reporting a technique as safe or effective. We will report frequencies of studies with conflict of interest stratified for the device used.
We will also calculate frequencies of studies reporting on a new technique in particular centre(s) and not reporting that informed consent was acquired from included patients.

5. Sensitivity analyses

The main analyses will include a sensitivity analysis for studies including unruptured aneurysms (elective cases) only for safety outcomes. Furthermore, for effectiveness and durability outcomes, a sensitivity analysis will be reported if the follow-up time is not comparable between groups.

We will also report the results of the main analysis for studies including more than 100 patients, as the number of patients might be a surrogate marker for better-quality studies.

eAppendix 9. Characteristics of Included Studies

Article	***Journal***	***Intervention***	***Number of patiënts***	***Number of centers***	***Coordinating country***	***Study Design***	***Study period***	***Duration of FU (months)***	***Funding***	***Conflict of Interest***	***Safety claim***	***Effectiveness claim***	***Durability claim***
Aguilar-Salinas, 2019[1]	Cureus Open Access	**SAC**	**120**	**1**	**USA**	**RO**	**2007-2014**		**None**	**None**	**Safe**	**Effective**	**Durable**
Pierot, 2017[2]	AJNR Am J Neuroradiol	WEB	55	10	Multi-country	**PO**	**2014-2015**	**12**	**Yes**	**Yes**	**Safe**	**Effective**	**No mention**
Pierot, 2016[3]	J Neurosurg	WEB	**51**	10	Multi-country	**PO**	**2011-2013**	**6**	**Yes**	**Yes**	**Safe**	**Effective**	**Durable**
Chalouhi, 2015[4]	J Neurosurg	PED	**100**	**1**	**USA**	**RO**	**2011-2013**	**7.3**	**NR**	**Yes**	**Safe**	**Effective**	**No mention**
Briganti, 2016[5]	Acta Neurochir	FD	20	**1**	**Italy**	**RO**	**2013-2015**		**None**	**Yes**	**Safe**	**Effective**	**No mention**
Kraus, 2019[6]	NeuroIntervent Surg	FD	42	**3**	**Germany**	**RO**	**2015-2017**	**1**	**None**	**Yes**	**Safe**	**Effective**	**No mention**
Xue, 2019[7]	**Clin Neuroradiol**	SAC	23	**1**	**China**	**RO**	**2010-2017**	**58.3**	**Yes**	**None**	**Safe**	**Effective**	**Durable**
Dellaretti, 2018[8]	World Neurosurg	Clip	320	**1**	**Brazil**	RO	**2008-2016**		**None**	**None**	**Safe**	**Effective**	**No mention**
Lawson, 2018[9]	J Neurosurg	WEB	**109**	**14**	**UK**	RO	**2012-2014**	**3**	**Yes**	Yes	**Safe**	**No mention**	**No mention**
Griessenauer, 2017[10]	Neurosurgery	PED	**117**	**5**	**USA**	RO	**2009-2015**		**NR**	Yes	**Safe**	**Effective**	**No mention**
Pierot, 2019[11]	J Neurointervent Surg	FD	103	**13**	**France**	**PO**	**2014-2016**	**12**	**Yes**	**Yes**	**Safe**	**Effective**	**No mention**
Phillips, 2012[12]	Am J Neuroradiol	PED	**32**	**3**	**Australia**	**PO**	**2009-2011**	**22**	**NR**	**Yes**	**Safe**	**Effective**	**No mention**
Martínez-Galdámez, 2019[13]	J Neurointervent Surg	FD	41		**Multi**	RO	**2018-2018**		None	None	**Safe**	**No mention**	**No mention**

© 2023 Volovici V et al. *JAMA Network Open.*

Deshaies, 2007[14]	J Neurosurg	Coil	64	1	USA	PO	2003-2004		None	None	Safe	Effective	No mention
Taschner, 2007[15]	Neuroradiology	Coil	52	1	France	PO	2005-2007		NR	None	Safe	Effective	No mention
Möhlenbruch, 2015[16]	Am J Neuroradiol	FD	29	1	Germany	PO	2013-2014	3	NR	None	Safe	Effective	No mention
Clarençon, 2017[17]	Clin Neurorad	FD	7	1	France	PO	2010-2015	19	None	Yes	Safe	Effective	No mention
Guerreiro-Simoes, 2019[18]	J Neuroradiol	Coil	132	1	France	PO	2013-2017		NR	Yes	Safe	Effective	No mention
Fiorella, 2017[19]	J Neurointervent Surg	WEB	150	31	Multi	PO	2014-2016	1	Yes	Yes	Safe	No mention	No mention
Luecking, 2017[20]	Am J Neuroradiol	FD	50	1	Germany	PO	2014-2015		NR	NS	Safe	Doubt	Doubt
Fargen, 2015[21]	J Neurointervent Surg	Coil	99	13	USA	PO	2010-2012	5.2	NR	Yes	Safe	Effective	No mention
Ng, 2002[22]	Stroke	Coil	144	1	Australia	RO	1992-1998	24	NR	NS	Safe	Effective	Doubt
Gizewski, 2008[23]	Am J Neuroradiol	Coil	108	1	Germany	RO	1997-2005	6	NR	NS	Safe	Effective	No mention
Mukonoweshuro, 2003[24]	Neuroradiology	Coil	23	1	UK	RO	1996-2001	12	NR	NS	Safe	Effective	No mention
Cho, 2014[25]	Neuroradiology	Coil	59	1	South Korea	PO	2006-2012		Yes	None	Safe	Effective	No mention
Gonzalez, 2010[26]	Neurosurgery	Coil	196	1	USA	RO	1993-2010	16.2	NR	None	Safe	Effective	No mention
Oishi, 2013[27]	J Neurointerv Surg	Coil	25	1	Japan	RO	2003-2011	24.4	NR	None	Safe	Effective	No mention
Zhou, 2014[28]	Am J Neuroradiol	FD	28	1	China	PO	2010-2012	19	Yes	Yes	Safe	Effective	No mention
Guzzardi, 2018[29]	Radiol Med	FD	49	1	Italy	RO	2009-2014	33	NR	None	Safe	Effective	No mention
Gobble, 2012[30]	J Vasc Surg	Bypass	36	1	USA	RO	1990-2010	53	None	None	Safe	Effective	No mention

© 2023 Volovici V et al. *JAMA Network Open.*

**Van Rooij, 2018[31]**	Am J Neuroradiol	WEB	40	**1**	Netherlands	RO	**2016-2017**	**3**	NR	Yes	**Safe**	**Effective**	**No mention**
Sun, 2011[32]	Intervent Neuroradiol	Coil	28	**1**	China	RO	**2007-2010**	**14.8**	NR	NS	**Safe**	**Effective**	**No mention**
Gentric, 2013[33]	American Journal of Neuroradiology	Coil	107	10	France	PO	**2008-2010**	**18**	**Yes**	Yes	**Doubt**	**Effective**	**No mention**
Ozpeynirci, 2019[34]	Acta Neurochir	WEB	45	**1**	Germany	RO	**2013-2018**	**3**	NR	**None**	**Doubt**	**Effective**	**No mention**
Pujari, 2019[35]	World Neurosurg	PED	27	**1**	USA	RO	**2011-2017**		NR	**None**	**Safe**	**Effective**	**No mention**
Hanel, 2019[36]	J Neurointerv Surg	PED	141	23	USA	PO	**2014-2015**	**12**	**Yes**	Yes	**Safe**	**Effective**	**No mention**
Teramoto, 2019[37]	World Neurosurg	SAC	116	**1**	Japan	RO	**2007-2017**		NR	None	**No mention**	**Effective**	**No mention**
Enriquez-Marulanda, 2019[38]	World Neurosurg	PED	57	3	USA	RO	**2013-2017**	**10**	NR	None	**No mention**	**Effective**	**No mention**
Pujari, 2019[39]	J Neurointerv Surg	PED	10	**1**	USA	RO	**2011-2017**		None	None	**Doubt**	**Effective**	**No mention**
Haffaf, 2019[40]	J Neurointerv Surg	FD	19	**1**	France	RO	**2015-2016**	**8**	None	Yes	**Safe**	**Effective**	**No mention**
Wallace,2019[41]	World Neurosurg	PED	35	4	USA	RO	**2012-2018**	**14**	NR	None	**Safe**	**Effective**	**No mention**
Balaji, 2019[42]	World Neurosurg	Clip	52	**1**	Japan	RO	**2014-2018**		NR	None	**Safe**	**Effective**	**No mention**
Mooney,2019[43]	J Neurosurg	Clip	46	**1**	USA	RO	**2003-2007**		NR	None	**No mention**	**No mention**	**No mention**
**Roy,2019[44]**	World Neurosurg	Clip	91	**1**	USA	RO	**2002-2019**		NR	None	**Safe**	**Effective**	**No mention**
**Ni,2019[45]**	World Neurosurg	Clip	92	**1**	China	RO	**2007-2017**	**59.4**	NR	None	**Doubt**	**Effective**	**No mention**

© 2023 Volovici V et al. *JAMA Network Open.*

Zidan, 2016[46]	J neurad	Coil	88	**1**	France	RO	**2013-2015**	**1**	**None**	**Yes**	**Safe**	**Effective**	**No mention**
Zhu, 2013[47]	Eur Radiol	SAC	42	3	China	PO	**2005-2011**	**43.5**	Yes	None	**Safe**	**Effective**	**No mention**
Zang, 2015[48]	Clin Neurol Neurosurg	SAC	35	**2**	China	RO	**2007-2013**		NR	**NS**	**No mention**	**No mention**	**Not durable**
Wallace,2019[49]	World Neurosurg	BAC + SAC	135	1	USA	**PO**	**2014-2017**		NR	**Yes**	**Safe**	**No mention**	**No mention**
Aguilar-Perez 2014[50]	AJNR Am J Neuroradiol	WEB	28	1	Germany	**RO**			**Yes**	**Yes**	**Safe**	**Effective**	**No mention**
Zanaty, 2015[51]	Neurosurgery	PED	41	3	USA	**RO**	**2009-2014**	**9.86**	**None**	**None**	**Doubt**	**Doubt**	**No mention**
Zamar, 2018[52]	World Neurosurg	PED	109	**4**	USA	RO	**2011-2015**		**None**	**None**	**Doubt**	**No mention**	**No mention**
Yue, 2011[53]	Interv Neuroradiol	Coil	74	**1**	China	RO	**2008-2011**	**14.1**	**NR**	**NS**	**Safe**	**No mention**	**No mention**
Ten Brinck, 2019[54]	Neurosurgery	**SAC**	27	1	Netherlands	RO	**2015-2016**	**7.4**	**NR**	**Yes**	**No mention**	**Effective**	**No mention**
Yan, 2006[55]	Neuroradiology	Coil	26	1	Germany	RO	**2002-2003**	**6**	**NR**	**NS**	**No mention**	**No mention**	**No mention**
Xu, 2018[56]	World Neurosurg	**Clip**	20	1	China	RO	**2015-2017**	**18**	**NR**	**None**	**No mention**	**No mention**	**No mention**
Sheen, 2019[57]	Clinical Neurology and Neurosurgery	**Clip**	68	1	South Korea	**RO**	**2008-2014**	**6**	**None**	**None**	**No mention**	**No mention**	**No mention**
Brasiliense, 2019[58]	J Neurosurg	PED	205	9	USA	**RO**	**2014-2016**	**6**	**None**	**None**	**Doubt**	**Doubt**	**No mention**
Xiaoxi, 2017[59]	World Neurosurg	Coil	29	**1**	China	**PO**	**2014-2016**		**Yes**	**None**	**Safe**	**Effective**	**No mention**
Williams, 2014[60]	Interv Neuroradiol	Coil	82	**2**	**UK**	**RO**	**2005-2008**	**12**	**NR**	**NS**	**No mention**	**No mention**	**No mention**
Weber, 2005[61]	AJNR Am J Neuroradiol	**LE**	22	**2**	Germany	**PO**	**2001-2003**		**NR**	**NS**	**Doubt**	**No mention**	**No mention**

© 2023 Volovici V et al. *JAMA Network Open.*

Wanke, 2005[62]	Zentralbl Neurochir	**SAC**	25	**1**	Germany	RO	**2001-2004**		**NR**	**Yes**	**No mention**	**No mention**	**No mention**
Wang, 2017[63]	PLoS One	**SAC**	38	**1**	China	RO	**2014-2016**	**14.1**	**Yes**	**None**	**No mention**	**Doubt**	**No mention**
Wang, 2015[64]	Br J Neurosurg	**Clip**	30	**1**	China	RO	**1996-2014**	**28**	**Yes**	**None**	**No mention**	**No mention**	**No mention**
Wang, 2015[65]	Br J Neurosurg	**Clip**	52	**1**	China	RO	**2009-2014**	**6**	**Yes**	**None**	**No mention**	**No mention**	**No mention**
Wakhloo, 2015[66]	AJNR Am J Neuroradiol	**FD**	165	**24**	**Multi-county**	**PO**	**2010-2013**	**15.4**	**Yes**	**Yes**	**Safe**	**No mention**	**No mention**
Wakhloo, 2008[67]	Stroke	**SAC**	28	**1**	USA	**RO**	**1996-2007**		**NR**	**NS**	**No mention**	**No mention**	**No mention**
Wakhloo, 2007[68]	AJNR Am J Neuroradiol	Coil	**69**	**1**	USA	**PO**	**2002-2005**	**12**	**NR**	**None**	**No mention**	**No mention**	**No mention**
Wajnberg, 2009[69]	Surg Neurol	**SAC**	24	**1**	**Brazil**	RO	**2005-2008**	**7**	**NR**	**NS**	**No mention**	**No mention**	**No mention**
Volker, 2018[70]	Neurointervention	PED	89	**3**	Germany	RO	**2011-2017**		**None**	**Yes**	**Doubt**	**No mention**	**No mention**
Velasco González, 2018[71]	Neuroradiology	BAC + SAC	80	**5**	Germany	RO	**2009-2012**		**NR**	**NS**	**No mention**	**No mention**	**No mention**
Van Rooij, 2007[72]	AJNR Am J Neuroradiol	Coil	44	**1**	Netherlands	RO	**1995-2005**	**6**	**NR**	**NS**	**Doubt**	**Doubt**	**No mention**
Van Rooij, 2006[73]	AJNR Am J Neuroradiol	Coil	149	**1**	Netherlands	RO	**1995-2005**		**NR**	**NS**	**No mention**	**No mention**	**No mention**
Van Rooij, 2006[74]	AJNR Am J Neuroradiol	Coil	48	**1**	Netherlands	RO	**1995-2005**		**None**	**None**	**No mention**	**No mention**	**No mention**
Tureli, 2016[75]	Acta Neurochir (Wien)	**SAC**	47	**4**	Turkey	RO	**2013-2015**		**None**	**None**	**Doubt**	**Effective**	**No mention**
Thornton, 2000[76]	Surg Neurol	Coil	66	**1**	**USA**	RO	**1994-1999**	**22.3**	**Yes**	**Yes**	**No mention**	**No mention**	**No mention**
Hagen,2019[77]	AJNR Am J Neuroradiol	Coil	150	**1**	Germany	RO	**2008-2017**	**6**	**None**	**Yes**	**Safe**	**No mention**	**No mention**
Goertz,2019[78]	J Clinical Neuroscience	**SAC**	37	**1**	Germany	RO	**2014-2018**	**40.6**	**Yes**	**NS**	**Safe**	**Effective**	**No mention**

© 2023 Volovici V et al. *JAMA Network Open.*

Wang, 2017[79]	World Neurosurg	**Bypass**	32	**2**	China	RO	**2010-2016**	**56.4**	**NR**	**None**	**No mention**	**No mention**	**No mention**
Van Dijk, 2011[80]	Acta Neurochir (Wien)	**Clip**	105	**1**	Netherlands	RO	**2001-2006**	**3**	**NR**	**NS**	**No mention**	**No mention**	**No mention**
Thines, 2012[81]	Can J Neurol Sci	**Clip**	85	**1**	**France**	RO	**2000-2005**	**11.3**	**Yes**	**Yes**	**Safe**	**Effective**	**No mention**
Taschner, 2017[82]	AJNR Am J Neuroradiol	**FD**	53	**8**	**Multi-country**	RO	**2010-2015**	**28.1**	**None**	**None**	**No mention**	**No mention**	**No mention**
Tähtinen, 2013[83]	Neuroradiology	**SAC**	55	**3**	**Finland**	RO	**2003-2011**		**Yes**	**Yes**	**No mention**	**No mention**	**No mention**
De Leacy,2019[84]	J Neurointervent Surg	Coil	115	**10**	**USA**	RO	**2012-2017**	**7**	**NR**	**Yes**	**No mention**	**No mention**	**No mention**
Bhogal,2018[85]	World Neurosurg	**FD**	29	**1**	Germany	RO	**2009-2018**		**None**	**None**	**No mention**	**Doubt**	**No mention**
Schob, 2019[86]	J Neurointervent Surg	**FD**	25	**1**	Germany	**PO**	**2018-2018**	**23**	**None**	**None**	**Safe**	**Effective**	**No mention**
Tähtinen, 2012[87]	Neurosurgery	**FD**	24	**2**	**Finland**	RO	**2009-2010**		**NR**	**NS**	**No mention**	**No mention**	**No mention**
Symon, 1992[88]	Acta Neurochir (Wien)	**Clip**	63	**1**	**UK**	RO			**NR**	**None**	**No mention**	**No mention**	**No mention**
Suzuki, 2017[89]	Neurol Med Chir (Tokyo)	Coil	35	**1**	**Japan**	RO	**2011-2014**		**NR**	**NS**	**Safe**	**Effective**	**Niet durable**
Suh, 2008[90]	J Korean Neurosurg Soc	**Clip**	21	**2**	**South Korea**	RO	**2002-2006**		**Yes**	**Yes**	**Safe**	**No mention**	**No mention**
Sturiale, 2013[91]	AJNR Am J Neuroradiol	**Coil**	20	**1**	**USA**	RO	**2999-2012**		**None**	**Yes**	**No mention**	**No mention**	**No mention**
Starke, 2013[92]	J Neurointerv Surg	**Endo**	91	**2**	**USA**	RO	**2004-2011**		**NR**	**NS**	**Safe**	**Effective**	**No mention**
Souza, 2007[93]	Arq Neuropsiquiatr	**Endo**	152	**1**	**Brazil**	RO	**2002-2005**		**NR**	**NS**	**Safe**	**Effective**	**No mention**
Sorteberg, 2008[94]	Br J Neurosurg	**Clip**	109	**1**	**Norway**	RO	**1998-2003**		**NR**	**None**	**No mention**	**No mention**	**No mention**
Mine, 2018[95]	J Neurointervent Surg	WEB	48	**3**	**Belgium**	RO	**2010-2015**	**39**	**NR**	**NS**	**Safe**	**Effective**	**Durable**
Sluzewski, 2003[96]	AJNR Am J Neuroradiol	Coil	29	**1**	Netherlands	RO	**1994-2000**	**50**	**Yes**	**Yes**	**No mention**	**Not effective**	**No mention**

© 2023 Volovici V et al. *JAMA Network Open.*

Simon, 2014[97]	World Neurosurg	**LE**	113	**25**	**USA**	**PO**	**2008-2010**	**6**	**None**	**NS**	**No mention**	**No mention**	**Durable**
Shimizu, 2017[98]	J Clin Neurosci	**Occlusion**	28	**1**	**Japan**	RO	**2002-2015**	**65**	**None**	**Yes**	**Safe**	**Effective**	**Durable**
Shimizu, 2016[99]	AJNR Am J Neuroradiol	**SAC**	**377**	**1**	**Japan**	RO	**2006-2012**	**32**	**NR**	**None**	**No mention**	**No mention**	**No mention**
Sato, 2018[100]	World Neurosurg	**Endo + bypass**	**44**	**2**	**Japan**	RO	**2007-2012**	**35.6**	**NR**	**None**	**No mention**	**No mention**	**Durable**
Santillan, 2012[101]	Neurosurgery	SAC	77	**1**	**USA**	**RO**	**2002-2010**	**54.2**	**NR**	**None**	**No mention**	**No mention**	**No mention**
Rodríguez-Harná, 2017[102]	Neurosurgery	Clip	62	**1**	**USA**	**RO**	**1997-2013**		**Yes**	**None**	**Safe**	**No mention**	**No mention**
Raymond, 1997[103]	Neurosurgery	Coil	75	**1**	**Canada**	**RO**	**1992-1995**	**6**	**NR**	**NS**	**No mention**	**No mention**	**No mention**
Ravindran, 2018[104]	World Neurosurg	FD	46	**3**	**Multi**	**RO**	**2014-2017**		**NR**	**None**	**No mention**	**No mention**	**No mention**
Bender,2018[105]	J Neurointervent Surg	PED	69	**1**	**USA**	**RO**		**9.6**	**NR**	**Yes**	**Safe**	**No mention**	**No mention**
Cohen,2018[106]	J Neurointervent Surg	FD	47	**1**	**Israel**	**RO**	**2011-2016**	**3**	**None**	**None**	**No mention**	**Doubt**	**Doubt**
Raslan, 2011[107]	Neurosurgery	SAC	44	**1**	**USA**	**RO**	**2003-2009**		**NR**	**None**	**No mention**	**No mention**	**Doubt**
Raco, 2008[108]	J Neurosurg	Clip	104	**1**	**Italy**	**RO**	**1974-2004**	**42**	**NR**	**Ns**	**No mention**	**No mention**	**No mention**
Pumar, 2013[109]	J Neurointerv Surg	FD	20	**1**	**Spain**	**RO**	**2008-2011**		**NR**	**None**	**No mention**	**No mention**	**No mention**
Pierot, 2015[110]	AJNR Am J Neuroradiol	WEB	26	**10**	**Multi**	**RO**		**13.8**	**NR**	**Yes**	**No mention**	**No mention**	**Doubt**
Pierot, 2013[111]	Neurosurgery	WEB	33	**5**	**Multi**	**RO**	**2010-2012**		**NR**	**None**	**No mention**	**No mention**	**No mention**
Pierot, 2012[112]	AJNR Am J Neuroradiol	WEB	20	**3**	**Multi**	**PO**	**2010-2012**		NR	**Yes**	**No mention**	**No mention**	**No mention**
Pierot, 2010[113]	AJNR Am J Neuroradiol	**Endo**	**622**	**27**	**Multi**	**PO**	**2005-2006**		Yes	**NS**	**No mention**	**Effective**	**No mention**
Pierot, 2010[114]	AJNR Am J Neuroradiol	**Coil**	**773**	**20**	**France**	**PO**	**2006-2008**		Yes	**NS**	**No mention**	**No mention**	**No mention**
Pierot, 2006[115]	AJNR Am J Neuroradiol	Coil	261	**4**	**France**	**PO**	**2004-2004**		Yes	**NS**	**No mention**	**Doubt**	**No mention**

© 2023 Volovici V et al. *JAMA Network Open.*

Cay,2018[116]	Interventional Neuroradiology	SAC	48	**1**	**Turkey**	**RO**	**2015-2017**	**7.9**	None	None	**No mention**	**Doubt**	**No mention**
Dodier,2018[117]	World Neurosurg	**PED**	**40**	**1**	**Austria**	RO	**2010-2015**		**None**	**None**	**No mention**	**No mention**	**No mention**
Liang,2018[118]	World Neurosurg	**PED**	**35**	**1**	China	RO	**2015-2016**		**Yes**	**None**	**No mention**	**Doubt**	**No mention**
Ho,2018[119]	eNeurologicalSci	**SAC**	**29**	**1**	**Switserland**	RO	**2003-2016**	**42**	**None**	**None**	**Doubt**	**Doubt**	**No mention**
Zhou, 2019[120]	World Neurosurg	**Endo**	**30**	**3**	China	RO	**2014-2018**	**5.07**	**NR**	**None**	**No mention**	**Effective**	**No mention**
Zhou, 2014[121]	J Stroke Cerebrovasc Dis	**SAC**	**70**	**1**	China	RO	**2003-2012**	**33**	**NR**	**None**	**Safe**	**Effective**	**No mention**
Zhou, 2012[122]	Acta Neurochir (Wien)	**Endo**	**29**	**1**	China	RO	**2007-2011**	**22.5**	**Yes**	**None**	**Safe**	**Effective**	**No mention**
Zheng, 2017[123]	Acta Neurochir (Wien)	**SAC**	**50**	**2**	China	RO	**2007-2014**	**36.7**	**None**	**None**	**Safe**	**Effective**	**No mention**
Zheng, 2016[124]	World Neurosurg	**SAC**	**480**	**1**	China	RO	**2007-2014**	**44.8**	**NR**	**None**	**Safe**	**Effective**	**No mention**
Zhao, 2017[125]	Int J Neurosci	**Endo**	**67**	**1**	China	RO	**2011-2015**	**8.24**	**NR**	**None**	**No mention**	**Effective**	**No mention**
Zhang, 2018[126]	World Neurosurg	**PED**	**55**	**1**	China	RO	**2015-2016**		**Yes**	**NS**	**No mention**	**Doubtful**	**No mention**
Zhang, 2017[127]	World Neurosurg	**Endo**	**93**	**4**	China	RO	**2010-2014**	**13**	**Yes**	**NS**	**Safe**	**Effective**	**No mention**
Zhang, 2017[128]	Exp Ther Med	**Coil**	**27**	**1**	China	RO	**2013-2015**		**Yes**	**NS**	**No mention**	**Effective**	**No mention**
Zhang, 2017[129]	World Neurosurg	**Endo**	**30**	**2**	China	RO	**2007-2014**	**25**	**None**	**NS**	**Safe**	**Doubt**	**No mention**
Zhang, 2014[130]	J Clin Neurosci	**SAC**	**23**	**1**	China	RO	**2003-2012**		**Yes**	**None**	**No mention**	**Effective**	**No mention**
Zenteno, 2006[131]	Surg Neurol	**SAC**	**36**	**4**	**Multi**	**PO**	**2003-2005**		**NR**	**NS**	**No mention**	**No mention**	**No mention**
Yu, 2015[132]	Interv Neuroradiol	**SAC**	**35**	**1**	China	**PO**	**2007-2011**		**None**	**None**	**Safe**	**Effective**	**No mention**
Yu, 2004[133]	AJNR Am J Neuroradiol	**Coil**	**97**	**1**	China	RO	**1995-2001**		**NR**	**NS**	**Safe**	**Effective**	**No mention**
Youn, 2010[134]	J Korean Neurosurg Soc	**BAC + SAC**	**34**	**1**	**South Korea**				**NR**	**NS**	**Safe**	**Effective**	**No mention**

© 2023 Volovici V et al. *JAMA Network Open.*

Yavuz, 2014[135]	AJNR Am J Neuroradiol	PED	21	1	Turkey				NR	Yes	Safe	Effective	No mention
Yavuz, 2008[136]	J Neurosurg	SAC	37	1	Turkey				None	None	No mention	Effective	No mention
Yang, 2010[137]	J Clin Neurosci	SAC	84	1	China	RO	2008-2009		NR	None	Safe	Effective	No mention
Yakovlev, 2015[138]	Zh Vopr Neirokhir Im N N Burdenko	FD	210	1	Russia	RO	2009-2014		NR	NS	No mention	Effective	No mention
Ya, 2007[139]	Neuroradiol J	Coil	31	1	China	RO	2005-2006		NR	NS	Safe	Effective	No mention
Xu, 2018[140]	World Neurosurg	SAC	44	1	China	RO	2013-2016	14.18	Yes	None	Safe	Effective	No mention
Xu, 2017[141]	World Neurosurg	Clip	204	1	China	RO	1995-2008	62	Yes	NS	No mention	Effective	No mention
Xu, 2017[142]	J Neurointerv Surg	Coil	40	1	China	RO	2007-2014	50	NR	None	No mention	Effective	No mention
Xu, 2015[143]	Cell Biochem Biophys	Coil	58	1	China	RO	2010-2012		None	None	Safe	Effective	No mention
Xu, 2009[144]	Neuroradiol J	BAC + SAC	55	1	China	RO	2002-2005		NR	NS	Safe	Effective	No mention
Wu, 2019[145]	J Stroke Cerebrovasc Dis	Coil	32	1	China	RO	2014-2017	9.3	Yes	None	Safe	Effective	No mention
Wong, 2007[146]	Surg Neurol	Coil	42	1	China	RO			NR	NS	Safe	No mention	No mention
Wolynski, 2007[147]	Clinical Neuroradiology	Coil	116	1	Switserland	RO	1993-2003	46.8	NR	NS	Safe	Effective	No mention
Willinsky, 2009[148]	AJNR Am J Neuroradiol	Endo	377	1	Canada	RO	1994-2008		NR	NS	No mention	Effective	Durable
Weber, 2007[149]	Neuroradiology	SAC	30	4	Germany	RO	2003-2005		NR	Yes	Safe	Effective	No mention
Wanke, 2002[150]	AJNR Am J Neuroradiol	Coil	39	1	Germany	RO	1997-2000		NR	NS	Safe	Effective	No mention
Wang, 2019[151]	J Neurointerv Surg	SAC	23	1	China	RO	2015-2017	16	Yes	None	Safe	No mention	No mention
Wang, 2018[152]	J Neurointerv Surg	SAC	229	1	USA	RO	2009-2016		NR	Yes	Safe	Effective	No mention
Wang, 2017[153]	AJNR Am J Neuroradiol	SAC	22	1	China	RO	2014-2016	16.1	Yes	NS	Safe	Effective	No mention

© 2023 Volovici V et al. *JAMA Network Open.*

Wang, 2016[154]	World Neurosurg	**Endo**	**47**	**1**	China	RO	**2012-2014**	**22.8**	**Yes**	**NS**	**Safe**	**No mention**	**No mention**
Wang, 2013[155]	J Neurointerv Surg	**Endo**	**126**	**1**	China	RO	**2009-2011**	**16.6**	**Yes**	**None**	**No mention**	**Effective**	**No mention**
Wang, 2013[156]	Acta Neurochir (Wien)	**Endo**	**56**	**1**	China	RO	**2009-2010**	**18.45**	**Yes**	**None**	**Safe**	**Effective**	**No mention**
Wan, 2014[157]	Med Sci Monit	**Coil**	**86**	**1**	China	RO	**2004-2012**		**Yes**	**None**	**Safe**	**Effective**	**No mention**
Wallace, 2019[158]	World Neurosurg	**PED**	**20**	**1**	**USA**	RO			**NR**	**Yes**	**Safe**	**Effective**	**No mention**
Waldenberger, 2008[159]	Surg Neurol	**Endo**	**29**	**1**	**Austria**	RO	**1997-2006**	**23**	**NR**	**NS**	**Safe**	**Effective**	**No mention**
Waldau, 2012[160]	J Neurointerv Surg	**Coil**	**141**	**3**	**USA**	RO			**NR**	**None**	**Safe**	**No mention**	**No mention**
Wakhloo, 2012[161]	AJNR Am J Neuroradiol	**SAC**	**147**	**2**	**USA**	**PO**	**2007-2010**	**11.8**	**Yes**	**Yes**	**Safe**	**Effective**	**Durable**
Voigt, 2017[162]	Front Neurol	**SAC**	**39**	**1**	**Germany**	RO	**2015-2017**		**Yes**	**None**	**No mention**	**Effective**	**No mention**
Veznedaroglu, 2008[163]	Neurosurgery	**Coil**	**81**	**1**	**USA**	**PO**	**2004-2006**		**NR**	**Yes**	**Safe**	**Effective**	**No mention**
Vendrell, 2011[164]	AJNR Am J Neuroradiol.	**SAC**	**49**	**1**	**France**	**PO**	**2003-2009**	**6**	**NR**	**NS**	**Safe**	**Effective**	**Durable**
Velioglu, 2015[165]	Turk Neurosurg	**Endo**	**49**	**1**	**Turkey**	RO	**1995-2011**	**12**	**NR**	**NS**	**Safe**	**Effective**	**No mention**
Velioglu, 2012[166]	Neuroradiology	**SAC**	**76**	**1**	**Turkey**	RO	**2008-2011**		**NR**	**None**	**No mention**	**Effective**	**No mention**
Van Rooij, 2018[167]	Interv Neuroradiol	**WEB**	**51**	**1**	Netherlands	RO	**2015-2017**	**3**	**Yes**	**Yes**	**Safe**	**Effective**	**No mention**
Van Rooij, 2017[168]	AJNR Am J Neuroradiol	**WEB**	**100**	**1**	Netherlands	**PO**	**2015-2017**		**Yes**	**None**	**Safe**	**Effective**	**No mention**
Van Rooij, 2016[169]	AJNR Am J Neuroradiol	**WEB**	**32**	**1**	Netherlands	**PO**	**2015-2015**		**NR**	**Yes**	**Safe**	**Effective**	**No mention**
Van Rooij, 2006[170]	AJNR Am J Neuroradiol	**Endo**	**22**	**1**	Netherlands	RO	**1995-2005**		**NR**	**NS**	**Safe**	**Effective**	**No mention**
Van Doormaal, 2010[171]	Neurosurgery	Clip + bypass	**33**	**1**	Netherlands	**RO**	**1994-2008**		NR	Yes	Safe	No mention	No mention
Ulfert, 2018[172]	Clin Neuroradiol	WEB	21	1	Germany	PO	2013-2015		NR	Yes	Safe	No mention	No mention

© 2023 Volovici V et al. *JAMA Network Open.*

Ulfert, 2018[173]	J Neurointerv Surg	SAC	36	3	Germany	RO	2016-2017	6.10	None	Yes	Safe	Effective	No mention
Tsai, 2019[174]	J Neurointerv Surg	SAC	58	1	USA	RO	2018-2018		None	Yes	Safe	No mention	No mention
Topcuoglu, 2016[175]	World Neurosurg	FD	28	1	Turkey	RO	2010-2015	10.30	None	None	No mention	No mention	No mention
Tomatis, 2019[176]	World Neurosurg	Clip	221	2	Italy	RO	2009-2015	3.00	None	None	Safe	No mention	No mention
Tjahjadi, 2016[177]	Neurosurgery	Clip	96	2	Finland	RO	2004-2014		Yes	None	Safe	No mention	No mention
Tevah, 2011[178]	J Neuroradiol	LE	38	1	Chile	RO	2003-2007	89.20	NR	None	Safe	Effective	No mention
Teping, 2019[179]	World Neurosurg	Clip	45	2	Germany	RO	2015-2018	9.60	NR	None	Safe	Effective	No mention
Teleb, 2014[180]	J Neurointerv Surg	Endo	100	1	USA	RO	2005-2011		NR	None	Safe	No mention	No mention
Taqi, 2018[181]	Interv Neurol	Coil	81	8	USA	PO	2013-2015		Yes	Yes	Safe	Effective	No mention
Tanweer, 2014[182]	AJNR Am J Neuroradiol	PED	41	1	USA	RO		24.60	Yes	Yes	Doubt	Effective	No mention
Tan, 2011[183]	J Neurosurg	SAC	34	1	China	RO	2005-2009	26.70	Yes	None	Safe	No mention	Durable
Takayasu, 2000[184]	J Neurosurg	Clip	347	1	Japan	RO	1996-1998	12.00	NR	NS	Safe	No mention	No mention
Szikora, 2013[185]	AJNR Am J Neuroradiol	FD	27	1	Hungary	RO	2006-2011		Yes	Yes	No mention	Effective	No mention
Suh, 2010[186]	AJNR Am J Neuroradiol	BAC + SAC	20	2	South Korea	RO	2005-2008	12.30	NR	NS	Safe	No mention	No mention
Stiefel, 2010[187]	J Neurointerv Surg	Endo	77	1	USA	PO	2000-2008		NR	None	Safe	No mention	No mention
Stetler, 2015[188]	J Neurointerv Surg	Coil	85	1	USA	RO	2005-2012		NR	None	Safe	Effective	No mention
Stapleton, 2015[189]	Neurosurgery	Clip	49	1	USA	RO	2000-2013	25.30	NR	None	No mention	Effective	No mention

© 2023 Volovici V et al. *JAMA Network Open.*

Standhardt, 2008[190]	Stroke	Coil	173	1	Austria	RO	1992-2004		None	None	Safe	No mention	No mention
Spiotta, 2018[191]	AJNR Am J Neuroradiol	SAC	34	10	USA	PO		12.00	Yes	Yes	Safe	Effective	No mention
Spiotta, 2017[192]	Neurosurgery	SAC	34	10	USA	PO		6.00	Yes	Yes	Safe	No mention	No mention
Spiotta, 2017[193]	World Neurosurg	Coil	59	2	USA	RO	2016-2016		Yes	Yes	Safe	Effective	No mention
Sonobe, 2008[194]	Surg Neurol	Coil	247	1	Japan	RO	1997-2005		NR	NS	Safe	No mention	No mention
Solander, 1999[195]	J Neurosurg	Coil	38	1	USA	RO	1990-1997	15.00	NR	NS	No mention	No mention	No mention
Sokolowski, 2019[196]	J Clin Neurosci	Coil	33	1	USA	RO			Yes	None	No mention	Effective	No mention
Soeda, 2004[197]	Neurol Med Chir (Tokyo)	Coil	92	1	Japan	RO	2016-2001	12.00	NR	NS	No mention	Effective	No mention
Sirakov, 2019[198]	Clin Neuroradiol	FD	72	1	Bulgaria	RO	2015-2018		NR	None	Safe	Effective	No mention
Sirakov, 2018[199]	J Neurointerv Surg	WEB	29	1	Bulgaria	RO	2017-2017		NR	None	Safe	No mention	No mention
Sheehan, 2015[200]	Interv Neurol	Coil	20	1	Ireland	RO	2006-2011	16.00	None	None	No mention	Effective	No mention
Shankar, 2017[201]	J Neurointerv Surg	SAC	100	10	Canada	RO	2013-2015	7.52	NR	Yes	Safe	Effective	No mention
Sedat, 2018[202]	Neuroradiol	SAC	156	1	France	RO	2008-2012		None	None	Safe	Effective	Durable
Sedat, 2009[203]	Neuroradiology	SAC	42	2	France	RO	2003-2007	42.00	NR	None	No mention	Effective	Doubt
Sedat, 2002[204]	Stroke	Coil	52	1	France	RO	1993-2000		NR	NS	No mention	Effective	No mention
Sattur, 2019[205]	World Neurosurg	Endo	33	1	USA	RO	2007-2019		NR	Yes	Safe	Effective	No mention
Saraf, 2012[206]	J Neurosurg Pediatr	Endo	23	1	India	RO	1998-2010	36.00	NR	None	Safe	Effective	Durable

© 2023 Volovici V et al. *JAMA Network Open.*

Santillan, 2019[207]	Interv Neuroradiol	SAC	25	1	USA	RO	2015-2018	15.80	None	None	Safe	Effective	No mention
Santillan, 2018[208]	Interv Neuroradiol	SAC	35	1	USA	RO	2015-2017	10.50	None	None	Safe	No mention	No mention
Sandalcioglu, 2005[209]	Zentralbl Neurochir	Endo	28	1	Germany	RO	1997-2002	9.00	NR	NS	No mention	Effective	No mention
Samaniego, 2018[210]	Interv Neuroradiol	SAC	30	7	USA	RO			NR	Yes	Doubt	Effective	No mention
Adeeb, 2017[211]	World Neurosurg	PED	50	3	USA	RO	2009-2016		NR	None	No mention	No mention	No mention
Ghani, 2016[212]	Malays J Med Sci	Clip	30	1	Malaysia	RO	2013-2014	6.00	None	None	No mention	No mention	No mention
Abla, 2016[213]	J Neurosurg	Bypass	35	1	USA	PO	1997-2014	16.30	NR	Yes	No mention	Effective	No mention
Aydin, 2012[214]	Acta Neurochir	Clip	192	1	Turkey	PO	1997-2008	87.60	None	None	Safe	Effective	No mention
Gory, 2014[215]	Acta Neurochir	Coil	390	34	Multi-country	PO	2006-2007		NR	None	Safe	Doubt	No mention
Choi, 2012[216]	J Cerebrovasc Endovasc Neurosurg	Clip	125	1	South Korea	RO	2007-2010	22.50	NR	NS	Safe	No mention	No mention
Jung, 2011[217]	J Korean Neurosurg Soc.	Clip	48	1	South Korea	RO	1999-2010		NR	NS	Doubt	Doubt	No mention
Adeeb, 2017[218]	AJNR Am J Neuroradiol	PED	218	3	USA	RO	2009-2016		NR	Yes	No mention	Effective	No mention
Cai, 2005[219]	J Neurosurg	Coil	63	1	France	RO	1998-2003	13.00	NR	NS	No mention	No mention	No mention
Andic, 2017[220]	J Neurointervent Surg	Endo	53	1	Turkey	RO	2011-2016	16.20	NR	None	Doubt	Effective	No mention
Akpek, 2005[221]	AJNR Am J Neuroradiol	SAC	32	1	USA	RO	2002-2003		Yes	NS	No mention	Doubt	No mention
Aletich, 2000[222]	J Neurosurg	Coil	72	1	USA	RO	1994-1999		NR	NS	No mention	No mention	No mention
Benes, 2010[223]	Cent Eur Neurosurg	Coil	131	1	UK	PO	1996-2005	10.40	NR	None	Doubt	No mention	No mention

© 2023 Volovici V et al. *JAMA Network Open.*

Elewa, 2018[224]	Egypt J Neurol Psychiatr Neurosurg	Coil	31	1	Egypt	RO	2011-2016		None	None	Doubt	No mention	No mention
Chalouhi, 2013[225]	Clin Neurol Neurosurg	Coil	72	1	USA	RO	2009-2011		NR	NS	Safe	Effective	No mention
Liu, 2016[226]	Turk Neurosurg	Coil	45	1	China	RO	2008-2011	17.00	NR	NS	Safe	No mention	No mention
Arustamyan, 2015[227]	Zh Vopr Neirokhir Im N N Burdenko	SAC	37	1	Russia	RO	2004-2014	20.00	NR	NS	No mention	Doubt	No mention
Bracard, 2010[228]	J Neurosurg	Coil	140		France	RO	1992-2001	51.60	NR	None	No mention	No mention	No mention
Bavinzski, 1999[229]	J Neurosurg	Coil	45	1	Austria	RO	1992-1998	27.40	NR	NS	Safe	Effective	Doubt
Bergui, 2004[230]	Neuroradiology	Coil	45	1	Italy	CS	1984-2001	6.00	NR	NS	No mention	No mention	No mention
Bhogal, 2018[231]	Neurointervention	FD	25	1	Germany	RO	2015-2017	8.10	NR	Yes	No mention	Doubt	No mention
Gory, 2015[232]	Am J Neuroradiol	WEB	40	4	Multi-country	RO	2012-2014		NR	NS	Safe	Doubt	No mention
Bhogal, 2017[233]	J NeuroIntervent Surg	FD	26	1	Germany	RO	2009-2016		NR	Yes	Doubt	Effective	No mention
Bhogal, 2017[234]	J NeuroIntervent Surg	FD	56	1	Germany	RO	2009-2016		NR	Yes	No mention	Effective	No mention
Cho, 2013[235]	Am J Neuroradiol	SAC	32	1	South Korea	PO	2008-2012	15.70	Yes	NS	No mention	Effective	No mention
Gory, 2013[236]	Neuroradiology	SAC	64	7	Multi-country	PO	2009-2010		NR	None	Doubt	Effective	No mention
Chiu, 2015[237]	Am J Neuroradiol	PED	98	3	Australia	RO	2009-2011	30.00	NR	NS	Doubt	Effective	Durable
Chae, 2010[238]	Korean J Radiol	Coil	30	1	Korea	RO	2005-2008	13.30	NR	NS	No mention	Doubt	No mention
Lin, 2016[239]	Neurosurgery	PED	28	10	USA	RO	2011-2013	10.70	NR	None	Safe	Effective	No mention
Caroff, 2015[240]	Am J Neuroradiol	WEB	90	10	Multi-country	RO	2013-2014	3.80	NR	NS	Doubt	Doubt	No mention

© 2023 Volovici V et al. *JAMA Network Open.*

Johnson, 2013[241]	J Neurosurg	SAC	91	1	USA	CS	2002-2012	22.92	NR	Yes	Doubt	No mention	No mention
Kühn, 2016[242]	J Neurointervent Surg	SAC	41	1	USA	RO	2006-2012	6.00	NR	None	No mention	Doubt	No mention
Geyik, 2013[243]	Am J Neuroradiol	SAC	468	1	Turkey	RO	2004-2010		NR	NS	Safe	Effective	Durable
Byrne, 2001[244]	Ann R Coll Surg Engl	Coil	317	1	UK	RO	1992-1997		NR	NS	Doubt	Doubt	No mention
Kang, 2007[245]	Neurosurgery	Coil	76	5	South Korea	PO	2005-2006		Yes	NS	Doubt	Doubt	Durable
Bonadio, 2017[246]	Arquivos Brasileiros de Neurocirurgia Brazilian Neurosurgery	Coil	31	1	Brazil	RO	2005-2015		NR	NS	Doubt	Doubt	No mention
Das, 2017[247]	World Neurosurgery	Clip	85	1	India	RO	2012-2015	11.60	NR	None	Doubt	Doubt	No mention
Giacomini, 2015[248]	Intervent Neuroradiol	FD	77	1	Brazil	RO	2010-2013	6.00	None	None	Safe	Effective	No mention
Pahl, 2016[249]	Arq Neuropsiquiatr	Clip	43	1	Brazil	RO	2009-2014		NR	NS	No mention	No mention	No mention
Oishi, 2009[250]	Neurol med Chir (Tokyo)	Coil	112	1	Japan	RO	2001-2007	17.00	NR	NS	Doubt	Doubt	No mention
Chung, 2014[251]	J Neurosurg	SAC	72	4	South Korea	RO	2007-2012	18.80	NR	None	No mention	No mention	No mention
Cho, 2014[252]	Interventional Neuroradiology	SAC	55	2	South Korea	RO	2012-2013	6.00	Yes	None	No mention	No mention	No mention
Fargen, 2014[253]	J Neurointervent Surg	Coil	99	13	USA	PO	2010-2012		None	Yes	Safe	Effective	No mention
Choulakian, 2010[254]	J Neurointervent Surg	Endo	113	1	USA	RO	2001-2010		None	Yes	Doubt	Doubt	No mention
Geyik, 2008[255]	Neuroradiology	Coil	78	1	Turkey	PO			NR	None	Safe	No mention	No mention

© 2023 Volovici V et al. *JAMA Network Open.*

Lylyk, 2005[256]	J Neurosurg	SAC	50	1	Argentina	PO	2002-2004		NR	NS	Safe	No mention	No mention
Durst, 2014[257]	ajnr	Coil	65	1	USA	RO	2001-2009		Yes	Yes	Safe	No mention	No mention
Niemann, 2004[258]	Am J Neuroradiol	Coil	133	1	USA	PO	2000-2002		NR	NS	Safe	Doubt	No mention
Klisch, 2010[259]	Neuroradiology	Coil	45	1	Germany	RO	2008-2009		NR	Yes	No mention	No mention	No mention
Gu, 2013[260]	J Clin Neurosci	SAC	54	1	China	RO	2005-2010	14.00	NR	None	Safe	Effective	No mention
Guglielmi, 2009[261]	J Neurosurg	Coil	318	1	Italy	CS			NR	None	Doubt	Doubt	No mention
Ilyas, 2018[262]	Clin Neurol Neurosurg	Coil	32	1	USA	RO	2016-2017		NR	NS	Safe	Doubt	No mention
Calvacante, 2013[263]	Elsevier Masson	Clip	28	1	France	RO	1990-2011	6.00	NR	None	No mention	No mention	No mention
Alghamdi, 2016[264]	Neuroradiology	SAC	40	3	Belgium	RO	2013-2014		NR	Yes	Safe	Effective	Doubt
O'Kelly, 2013[265]	Am J Neuroradiol	PED	97	7	Canada	RO	2008-2010		NR	NS	No mention	No mention	No mention
Mocco, 2009[266]	J Neurosurg	Coil	141	10	USA	CS			Yes	Yes	Doubt	No mention	No mention
Colby, 2018[267]	J Neurosurg	FD	494	1	USA	RO	2011-2016	11,60	NR	Yes	Doubt	Doubt	No mention
Invergo, 2012[268]	J Neuroimaging	Coil	56	1	USA	RO	2004-2009		NR	None	Doubt	Effective	No mention
Galal, 2013[269]	British Journal of Neurosurgery	SAC	43	1	USA	RO	2005-2010	16,00	NR	None	Doubt	Effective	No mention
Cho, 2016[270]	Clin Neuroradiol	Coil	172	1	South Korea	PO	2010-2013		NR	None	Safe	Effective	No mention
Lan, 2006[271]	Surg Neurol	Clip	100	1	China	RO	2000-2005		Yes	NS	No mention	Effective	No mention
Lubicz, 2004[272]	Am J Neuroradiol	Coil	68	1	France	CS	1996-2002	20.00	NR	NS	No mention	Effective	No mention

Grunwald, 2007[273]	Neuroradiology	Coil	220	1	Germany	RO	2000-2003	1.00	NR	NS	Safe	Effective	Not durable
Muto,2017[274]	Radiol Med	SAC	40	3	Italy	RO	2010-2014		None	None	Doubt	Effective	No mention
Kaya, 2016[275]	Turk Neurosurg	FD	96	1	Turkey	RO	2010-2013		NR	NS	Safe	Effective	No mention
Kocer, 2014[276]	J Neurosurg	FD	33	1	Turkey	RO	2012-2013	3.60	NR	Yes	No mention	No mention	No mention
Moritz, 2012[277]	J Neuroradiol	Coil	32	1	France	PO	2006-2006	16.90	NR	None	No mention	Not effective	Not durable
Leonardi, 2011[278]	Intervent Neuroradiol	FD	25	1	Italy	RO	2007-2009		NR	NS	No mention	No mention	No mention
de Sousa, 2005[279]	Surg Neurol	Clip	30	1	Brazil	RO	1986-1997		NR	NS	Safe	Doubt	No mention
Akurkar, 2012[280]	Surg Neurol	SAC	42	1	India	RO	2008-2012	12.00	NR	NS	Safe	Effective	No mention
Ferrel, 2012[281]	World neurosrugery	SAC	57	1	USA	RO	2009-2012	12.10	NR	Yes	Doubt	Doubt	No mention
Fiorella, 2005[282]	Neurosurgery	SAC	64	1	USA	RO	2002-2004		NR	Yes	Doubt	Doubt	No mention
Maimon, 2012[283]	Acta Neurochir	FD	28	1	Israel	RO	2008-2010		NR	None	Safe	Effective	No mention
McDougall, 1996[284]	J Neurosurg	Coil	33	1	USA	CS	1991-1995	15.00	NR	NS	No mention	No mention	No mention
Lubicz, 2010[285]	Stroke	FD	29	3	Belgium	PO	2009-2010	6.00	NR	NS	Doubt	Doubt	No mention
Bae, 2019[286]	World neurosurgery	Coil	98	1	Korea	RO	2008-2012	25.50	NR	NS	Safe	Effective	No mention
Bartolini, 2015[287]	Intervent Neuroradiology	Coil	33	1	France	RO	2013-2014		Yes	None	Safe	Effective	No mention
Atallah, 2019[288]	Neurosurgery	PED	437	1	USA	RO	2011-2016	19.80	NR	NS	No mention	Doubt	No mention
Aydin, 2015[289]	Am J Neuroradiol	SAC	80	3	Turkey	RO	2012-2014	7.20	NR	NS	Safe	Effective	Durable

© 2023 Volovici V et al. *JAMA Network Open.*

Aydin, 2018[290]	Am J Neuroradiol	SAC	40		Turkey	RO	2012-2018	24.80	NR	NS	Doubt	Effective	Durable
Ban, 2018[291]	Neuroradiology	Coil	51	1	South Korea	RO	2003-2015	48.50	None	None	Safe	Effective	No mention
Bartolini, 2014[292]	Am J Neuroradiol	SAC	97	1	France	RO	2006-2013		NR	NS	No mention	Effective	Durable
Bechan, 2016[293]	AJNR AM J Neuroradiol	Coil	146	1	Netherlands	PO	1995-2015	35.00	NR	NS	Safe	Effective	No mention
Geyik, 2007[294]	Interventional Neuroradiology	Coil	35	1	Turkey	CS			NR	None	No mention	Effective	Doubt
Deng, 2012[295]	Neurosurg	SAC	41	1	China	RO	2003-2010		NR	None	No mention	No mention	No mention
Çinar, 2013[296]	Diagn Interv Radiol	PED	45	1	Turkey	RO	2009-2010		NR	None	Safe	Effective	No mention
Martínez-Galdámez, 2015[297]	J Neurointervent Surg	PED	30	1	Spain	CS	2014-2014		NR	Yes	No mention	No mention	No mention
Lopes, 2014[298]	Neurosurgery	SAC	410	1	USA	RO	2002-2012		NR	Yes	No mention	No mention	Durable
Colby, 2016[299]	J Neurointervent Surg	PED	42	1	USA	CS	2015-2015		NR	None	Doubt	Doubt	No mention
Lijima, 2005[300]	Radiology	Coil	142	1	France	RO	1998-2002		NR	None	Safe	Doubt	No mention
Linzey, 2017[301]	J Neurointervent Surg	SAC	122	1	USA	RO	2005-2012		NR	None	Safe	Effective	No mention
Molyneux, 2004[302]	Am J Neuroradiol	LE	97	20	Mutli-country	PO		12.00	NR	NS	Safe	Effective	Durable
Hokari, 2013[303]	Neurosurg Rev	Clip	166	1	Japan	RO	1991-2008	7.90	NR	None	Doubt	Effective	Doubt
Vora, 2010[304]	J Neuroimaging	Coil	26	1	USA	RO	1999-2008		NR	NS	Doubt	Doubt	No mention
Colby, 2013[305]	J Neurointervent Surg	PED	34	1	USA	RO	2011-2011		None	None	Safe	Doubt	No mention
Lubicz, 2014[306]	AJNR Am J Neuroradiol	WEB	45	12	Belgium	RO	2010-2012		Yes	NS	Safe	Effective	Doubt

© 2023 Volovici V et al. *JAMA Network Open.*

Abdulrauf, 2017[307]	J neurosurg	Clip	30	1	USA	PO		1.00	NR	None	No mention	Doubt	No mention
Lee, 2016[308]	J. Cerebrovasc. Endovasc Neurosurg.	Coil	150	1	South Korea	RO	2011-2015		NR	None	Safe	No mention	No mention
Yang, 2008[309]	Neurol Res	SAC	36	1	China	RO		11.30	NR	NS	No mention	No mention	No mention
Fischer, 2012[310]	Neuroradiology	PED	88	1	Germany	CS	2009-2011	10.00	NR	Yes	Safe	Doubt	No mention
Xu, 2011[311]	Chin Med J	SAC	40	1	China	CS	2008-2010	3.00	NR	NS	No mention	No mention	No mention
Feng, 2017[312]	World Neurosurg	Coil	264	1	China	RO	2009-2014	47.10	Yes	Yes	Safe	Doubt	Doubt
Becske, 2017[313]	J Neurosurg	PED	107	10	Mutli-country	PO	2008-2009	34.79	Yes	NS	Safe	Effective	Durable
Debrun, 2000[314]	Surg Neurol	Coil	208	1	USA	RO			NR	NS	Doubt	No mention	No mention
Behme, 2015[315]	Am J Neuroradiol	WEB	52	2	Germany	RO	2012-2014		NR	NS	Safe	Doubt	No mention
Machi, 2015[316]	Am J Neuroradiol	SAC	29	1	France	RO	2012-2013	10.00	NR	NS	No mention	No mention	No mention
Lozier, 2004[317]	Neuorsurgery	Clip	98	1	USA		1987-1999	88.80	None	None	Doubt	No mention	No mention
Oishi, 2012[318]	Am J Neuroradiol	Coil	457	1	Japan	RO	2001-2009	34.70	NR	NS	Safe	Effective	No mention
Raymond, 2001[319]	Radiology	WEB	25	1	Canada	RO	2000-2001	7.28	NR	NS	No mention	Doubt	No mention
Wang, 2011[320]	Neurol India	SAC	46	1	China	RO	2009-2010	9.10	None	None	Safe	Effective	No mention
Behme, 2015[321]	J Neurointervent Surg	SAC	32	2	Mutli-country	RO	2012-2012		NR	None	Safe	Effective	No mention
Asiltürk, 2017[322]	Neurol Neurochir Pol	Clip	90	1	Turkey	RO	2011-2013	52.30	None	None	**Safe**	No mention	**Doubt**
Lubicz, 2007[323]	J Neuroradiol	Coil	144	1	Belgium	PO	2004-2006		NR	NS	**Doubt**	No mention	No mention

© 2023 Volovici V et al. *JAMA Network Open.*

Gupta, 2011[324]	Neurol India	Clip	165	1	India	RO	1999-2005	31.80	None	None	**Safe**	**Effective**	**Durable**
Kulcsár, 2013[325]	Neuroradiology	SAC	113	1	Switzerland	RO	2001-2010		Yes	Yes	**Safe**	**Effective**	**Durable**
Bender, 2019[326]	Neurosurgery	PED	55	1	USA	RO	2011-2017		Yes	NS	**Safe**	**Effective**	No mention
Benitez, 2004[327]	Boston Scientific	SAC	48	1	USA	RO			NR	Yes	No mention	**Doubt**	No mention
Bohnstedt, 2013[328]	World neurosurg	Clip	122	1	USA	PO	1977-2008	6.00	NR	None	No mention	No mention	No mention
Pinsker, 2002[329]	Acta Neurochir suppl	Clip	41	1	Germany	RO	1994-2000	1.00	NR	NS	**Safe**	**Doubt**	No mention
Su, 2018[330]	Neurol Res	SAC	218	1	China	RO	2014-2016	8.80	Yes	None	**Safe**	**Effective**	**Doubt**
Limbucci, 2016[331]	J Neurointervent Surg	SAC	52	1	Italy	RO	2010-2013	28.00	NR	None	No mention	**Doubt**	**Doubt**
Sawada, 2000[332]	Interv. Neuroradiol	Coil	26	1	Japan	RO	1995-1999	3.00	NR	NS	**Doubt**	**Effective**	No mention
Tomasello, 2016[333]	Neurol Res	PED	47	4	Spain	RO	2010-2013	12.00	NR	None	**Doubt**	**Effective**	**Doubt**
Lv, 2016[334]	Ann Indian Acad Neurol	Coil	89	1	China	RO	2009-2011	22.00	Yes	None	**Safe**	**Doubt**	No mention
Mangiafico, 2002[335]	Interv Neuroradiol	Coil	37	1	Italy	RO	1999-2002		NR	NS	No mention	No mention	No mention
Khan, 2005[336]	Acta Neurochir suppl	Clip	75	1	Switzerland	RO	1993-2003	3.00	NR	NS	**Doubt**	**Doubt**	No mention
Mejdoubi, 2006[337]	Neuroradiology	Endo	222	1	France	PO	1998-2002	26.00	NR	None	No mention	No mention	No mention
Panigrahi, 2016[338]	Arch Neurosci	Clip	170	1	India	RO	2010-2013		NR	NS	**Doubt**	No mention	No mention
Terada, 2005[339]	Acta Neurochir suppl	Coil	76	1	Japan	CS	1999-2004	38.40	NR	NS	No mention	No mention	No mention

© 2023 Volovici V et al. *JAMA Network Open.*

Berenstein, 2006[340]	Am J Neuroradiol	Coil	100	1	USA	RO	2002-2005	5.30	NR	NS	**Safe**	No mention	No mention
Berge, 2012[341]	Am J Neuroradiol	FD	65	6	France	RO	2008-2009	12.00	NR	NS	No mention	**Effective**	No mention
Krisht, 2006[342]	Neurosurgery	Clip	116	1	USA	RO	1998-2003	35.00	NR	NS	**Safe**	**Effective**	**Durable**
Tateshima, 2000[343]	Neurosurgery	Coil	73	1	USA	RO	1990-1999	31.30	NR	NS	**Safe**	**Effective**	**Durable**
Wang, 2015[344]	Acta Radiol	Coil	88	1	China	RO	1998-2009		Yes	NS	No mention	**Not effective**	No mention
Bulters, 2011[345]	Acta Neurochir	Clip	200	1	UK	RO	2001-2004	60.00	NR	None	**Doubt**	**Effective**	No mention
Holmin, 2008[346]	Stroke	Coil	413	1	France	PO	1993-2005	54.00	Yes	NS	**Safe**	**Effective**	**Durable**
Tähtinen, 2009[347]	Radiology	SAC	61	1	Finland	RO	2003-2007		NR	NS	No mention	**Effective**	No mention
Cekirge, 2006[348]	Interventional Neuroradiology	LE	94	1	Turkey	RO	1999-2003	12.00	NR	NS	**Safe**	**Effective**	**Durable**
Fiorella, 2010[349]	J NeuroIntervent Surg	SAC	284	1	USA	PO	2002-2006		Yes	Yes	No mention	**Doubt**	No mention
Hara, 2016[350]	Acta Neurochir Suppl	Bypass	26	1	Japan	CS	2006-2013	15.30	NR	None	**Doubt**	**Doubt**	No mention
Heller, 2013[351]	Neurosurgery	SAC	76	1	USA	PO			Yes	None	**Doubt**	**Doubt**	No mention
Jödicke, 2018[352]	J Neurol Surg A Cent Eur Neurosurg	Clip	100	1	Germany	RO	2007-2013	6.00	NR	NS	**Safe**	**Effective**	**Doubt**
Mocco, 2011[353]	Neurosrugery	SAC	213	10	USA	RO			None	Yes	**Doubt**	**Effective**	No mention
Kremer, 2002[354]	Neuroradiology	Coil	79	1	Germany	RO	1993-1997	41.00	NR	NS	**Safe**	No mention	**Doubt**
Miyazawa 2002[355]	Clin Neurol Neurosurg	Clip	25	1	Japan	CS			NR	NS	**Doubt**	**Effective**	No mention

© 2023 Volovici V et al. *JAMA Network Open.*

Arustamyan, 2016[356]	Zh Vopr Neirokhir Im N N Burdenko	Coil	50	1	Russia	RO	2002-2014	42.70	NR	None	No mention	No mention	No mention
Biondi, 2007[357]	Neurosurgery	SAC	42	1	USA	RO	2002-2005	10.00	NR	NS	**Safe**	**Effective**	No mention
Birchall, 2001[358]	Br J Neurosurg	Coil	35	1	Australia	RO	1992-1998	42.70	NR	NS	**Safe**	**Effective**	No mention
Brasiliense, 2016[359]	Neurosurgery	Clip	32	1	USA	RO	2008-2014	1.00	NR	Yes	**Safe**	**Effective**	No mention
Horiuchi, 2011[360]	Neurological Research	Clip	333	1	Japan	RO	1988-2009		NR	NS	No mention	No mention	No mention
O'Hare, 2010[361]	AJNR Am J Neuroradiol	Coil	328	1	Ireland	RO	2003-2008		NR	NS	**Doubt**	**Doubt**	No mention
Miyachi, 2003[362]	Interv Neuroradiol	Coil	497	18	Japan	RO			NR	NS	**Doubt**	**Doubt**	No mention
Breu, 2016[363]	Radiol Res Pract	FD	28	1	Germany	RO	2009-2013		NR	None	**Safe**	**Effective**	No mention
Xu, 2014[364]	Kuwait Med J	Clip	146	1	China	RO	2003-2008	91.20	NR	NS	No mention	No mention	No mention
Hirsch, 2007[365]	J Vasc Interv Radiol	Coil	291	37	Multi-country	RO	2004-2006	6.54	NR	Yes	No mention	**Doubt**	**Doubt**
McLaughlin, 2017[366]	Journal of Medical Imaging and Radiation Oncology	SAC	152	1	Australia	RO	2012-2014	14.72	NR	None	No mention	No mention	No mention
Mont'alverne, 2005[367]	Interventional Neuroradiology	SAC	42	1	France	RO	1993-2003	9.20	NR	NS	No mention	No mention	No mention
Zeeshan, 2018[368]	Neurol India	Clip + bypass	73	1	USA	RO	2005-2018	32.30	None	None	**Safe**	**Effective**	No mention
Alhothi, 2010[369]	Neurol Neurochir Pol	SAC	26	1	China	RO	2004-2007		NR	None	**Safe**	**Effective**	No mention
Dabus, 2017[370]	J Neurointerv Surg	Coil	80	6	USA	CS			Yes	Yes	**Safe**	**Effective**	No mention
Kim, 2000[371]	Interv. Neuroradiol	Coil	141	1	USA	RO			NR	NS	No mention	No mention	No mention

Castaño, 2017[372]	Interv Neuroradiol	SAC	45	1	Spain	RO	2007-2017	6.00	None	None	**Safe**	**Effective**	No mention
Mortimer, 2014[373]	AJNR Am J Neuroradiol	Coil	295	1	UK	RO	1996-2012	6.00	Yes	Yes	**Safe**	**Effective**	No mention
Bruneau, 2015[374]	Neurosurgery	Clip	183	4	Belgium	RO	2001-2012	121.30	None	None	**Safe**	**Effective**	No mention
Clajus, 2017[375]	J NeuroIntervent Surg	WEB	108	1	Germany	PO	2010-2015	13.40	NR	Yes	**Safe**	**Effective**	No mention
Feng, 2016[376]	J NeuroIntervent Surg	SAC	97	1	China	RO	2014-2014	7.80	Yes	None	**Safe**	**Effective**	No mention
Oishi, 2013[377]	Neurol med Chir	Coil	31	1	Japan	RO	2002-2011	36.50	NR	None	**Safe**	**Effective**	No mention
Losif, 2018[378]	J NeuroIntervent Surg	SAC	90	2	France	PO		18.00	NR	None	**Safe**	**Effective**	No mention
Martínez-Galdámez, 2019[379]	Neuroradiology	SAC	84	8	Mutli-country	RO	2015-2017		NR	Yes	**Safe**	**Effective**	No mention
Kim, 2019[380]	World Neurosurg	Clip	36	1	South Korea	RO	1993-2017	76.80	Yes	Yes	**Safe**	**Effective**	**Durable**
Da Ros, 2019[381]	World Neurosurg	WEB	33	3	Italy	RO	2014-2017	14.00	None	None	**Doubt**	**Effective**	No mention
Briganti, 2017[382]	J NeuroIntervent Surg	FD	40	6	Italy	RO	2013-2015	12.00	NR	Yes	**Safe**	**Effective**	No mention
Li, 2018[383]	Turk Neurosurg	Coil	33	1	China	CS	2010-2015	6.00	NR	NS	**Safe**	**Effective**	No mention
Jindal, 2016[384]	J. Vasc. Interv. Neurol.	Coil	50	1	USA	RO	2011-2015		NR	NS	**Safe**	**Effective**	No mention
Malisch, 1997[385]	J Neurosurg	Coil	100	1	USA	PO	1990-1994	42.00	NR	NS	**Safe**	**Effective**	No mention
Jin, 2009[386]	J Korean Neurosurg Soc.	Coil	65	1	South Korea	RO	1997-2007		NR	NS	**Safe**	**Effective**	No mention
Fiorella, 2019[387]	J NeuroIntervent Surg	SAC	153	21	USA	PO		12.00	Yes	Yes	**Safe**	**Effective**	No mention
Mihalea, 2019[388]	J NeuroIntervent Surg	WEB	25	1	France	PO	2017-2018		None	None	**Safe**	**Effective**	No mention

© 2023 Volovici V et al. *JAMA Network Open.*

Cheung, 2018[389]	J NeuroIntervent Surg	WEB	98	3	Australia	RO	2012-2015	18.00	NR	None	**Safe**	**Effective**	**Durable**
Li, 2015[390]	Int J Clin Exp Med	SAC	38	1	China	RO	2009-2014		Yes	None	No mention	**Effective**	No mention
Peluso, 2008[391]	J Neurol Neurosurg Psychiatry	Coil	154	1	Netherlands	PO	1995-2006	53.00	NR	None	**Safe**	**Effective**	No mention
Fargen, 2012[392]	Neurosurgery	SAC	229	9	USA	RO	2007-2009	22.80	Yes	Yes	**Safe**	**Effective**	**Durable**
Chalouhi, 2014[393]	AJNR Am J Neuroradiol	Coil	324	1	USA	RO	2004-2011	26.30	NR	NS	**Safe**	**Effective**	No mention
Nussbaum, 2019[394]	J Neurosurg	Clip	716	1	USA	RO	1997-2015	102.00	NR	None	**Safe**	**Effective**	No mention
Li, 2015[395]	PLos One	SAC	116	1	China	RO	2010-2014	6.00	None	None	No mention	**Effective**	No mention
De Vries, 2013[396]	Stroke	FD	37	1	Netherlands	PO	2010-2012	6.00	NR	None	**Safe**	**Effective**	No mention
Dima, 2012[397]	J med life	Coil	116	1	Romania	RO	2005-2011		NR	NS	**Safe**	**Effective**	**Durable**
Briganti, 2017[398]	Neurosurg Focus	FD	60	1	Italy	RO	2008-2015		NR	Yes	**Safe**	**Effective**	**Durable**
Hai, 2009[399]	J Clin Neurosci	Coil	37	1	China	RO	2004-2007	15.90	NR	NS	**Safe**	**Effective**	**Durable**
Drescher, 2017[400]	Am J Neuroradiol	FD	50	4	Germany	RO	2013-2016	12.00	NR	NS	No mention	**Effective**	**Durable**
Hwang, 2011[401]	Am J Neuroradiol	Coil	96	1	South Korea	RO	2003-2010		Yes	NS	**Safe**	**Doubt**	No mention
Lu, 2012[402]	Eur J Radiol	SAC	46	1	China	RO	2002-2009	46.70	NR	None	**Safe**	**Effective**	No mention
Ma, 2019[403]	Neurosurg Rev	Endo	53	1	China	RO	2011-2016	42.00	NR	None	**Safe**	**Effective**	No mention
Briganti, 2015[404]	The Neurordiology Journal	Coil	29	1	Italy	RO	2004-2005	12.00	NR	NS	**Safe**	No mention	**Durable**

© 2023 Volovici V et al. *JAMA Network Open.*

Brown, 2017[405]	J Neurosurg	Clip	616	1	USA	RO	1990-2010		NR	None	**Doubt**	**Effective**	**Doubt**
Jankowitz, 2019[406]	J Neurointervent Surg	SAC	30		USA	PO	2015-2017	12.00	Yes	None	**Safe**	**Doubt**	No mention
Chen, 2019[407]	J Neurointervent Surg	Endo	49	2	USA	RO	2016-2018		None	Yes	**Safe**	**Effective**	No mention
Ciccio, 2019[408]	J Neurointervent Surg	SAC	55		Mutli-country	PO	2015-2019		None	None	No mention	**Effective**	No mention
Imamura, 2019[409]	Wolrd Neurosurg	Coil	122	26	Japan	PO		12.00	Yes	None	**Safe**	No mention	**Doubt**
D'Urso, 2012[410]	Am J Neuroradiol	Coil	118	1	USA	RO	1999-2010	37.00	NR	NS	**Safe**	**Effective**	No mention
Park, 2003[411]	Neurosurg	Coil	70	1	USA	RO	1993-2002	14.40	NR	NS	**Safe**	**Effective**	No mention
Lylyk, 2009[412]	Neurosurgery	PED	53	1	Argentina	PO	2006-2008	12.00	NR	Yes	**Safe**	**Effective**	**Durable**
Liao, 2018[413]	J Neuroradiol	Coil	92	1	France	RO	1992-2013	54.00	None	None	**Safe**	**Effective**	No mention
Chen, 2013[414]	J clin Neurosci	Coil	47	1	China	RO	2008-2011	18.60	NR	NS	**Safe**	**Effective**	No mention
Hwang, 2012[415]	EWHA Med J	Coil	33	1	South Korea	RO	2005-2010		NR	NS	**Safe**	**Doubt**	No mention
Hong, 2011[416]	J clin Neurosci	Coil	51	1	China	RO	2000-2009	54.20	NR	NS	**Safe**	No mention	No mention
Friedman, 2003[417]	AJNR Am J Neuroradiol	Coil	83	1	USA	RO	1991-2000	19.10	NR	NS	**Safe**	**Effective**	**Doubt**
Djurdjevic, 2019[418]	J Neurointervent Surg	SAC	101	1	Mutli-country	RO	2013-2018		None	None	**Safe**	**Effective**	No mention
Gao, 2012[419]	J clin Neurosci	Coil	102	1	China	RO	2000-2008	56.50	NR	NS	**Safe**	**Effective**	No mention
Park, 2011[420]	AJNR Am J Neuroradiol	Coil	120	10	Korea	PO	2008-2009		Yes	NS	**Safe**	**Doubt**	**Doubt**
Piano, 2013[421]	J Neurosurg	FD	101	1	Italy	RO	2008-2011	3.00	NR	NS	**Safe**	**Effective**	No mention

Brinkjikji, 2016[422]	J Neurosurg	SAC	31	2	Mutli-country	RO	2009-2014	18.30	NR	Yes	**Safe**	**Effective**	No mention
Buyukkaya, 2014[423]	Inventional neuroradiology	FD	32	1	Turkey	RO	2009-2013	17.00	NR	NS	**Safe**	**Effective**	No mention
Liu, 2018[424]	Med Sci Monit	SAC	71	2	China	RO	2015-2018	6.00	Yes	NS	**Safe**	**Effective**	No mention
Martínez-Galdámez, 2017[425]	J Neurointervent Surg	PED	50	7	Mutli-country	PO	2015-2015		Yes	Yes	**Safe**	**Effective**	No mention
Jin, 2013[426]	Neuroradiology	Coil	100	1	South Korea	RO	2003-2008	29.50	NR	None	**Safe**	**Effective**	**Not durable**
Colby, 2017[427]	J Neurointervent Surg	PED	41	1	USA	RO	2012-2016		NR	Yes	**Safe**	**Effective**	No mention
Mericle, 2006[428]	Neurosurg	Coil	31	1	USA	CS		10.00	Yes	NS	**Safe**	**Effective**	No mention
Mine, 2014[429]	J Neuroradiol	SAC	164	2	Belgium	RO	2004-2012		NR	Yes	**Safe**	**Effective**	No mention
Luo, 2012[430]	J Chin Med Assoc	SAC	59	1	Taiwan	RO	2005-2010	28.00	Yes	NS	**Safe**	**Effective**	**Doubt**
Ma, 2018[431]	J Neurointerv Surg	SAC	57	1	China	RO	2013-2016	9.00	Yes	None	**Safe**	**Effective**	No mention
Lee, 2013[432]	Clin Radiol	SAC	261	1	South Korea	RO	2008-2011		NR	NS	**Doubt**	**Effective**	No mention
Huang, 2013[433]	J clin Neurosci	SAC	27	1	China	RO	2008-2010	12.60	Yes	None	**Safe**	**Effective**	No mention
Delgado Almandoz, 2017[434]	Neuroradiology	PED	140	2	Mutli-country	RO	2011-2015		None	Yes	**Safe**	**Effective**	**Durable**
Katsaridis, 2006[435]	Am J Neuroradiol	SAC	50	1	Greece	RO	2003-2005		NR	NS	**Safe**	**Effective**	No mention
Kawabata, 2017[436]	Neuroradiol J	Coil	25	1	Japan	RO	2012-2016		None	None	**Safe**	No mention	**Not durable**
Caragliano, 2019[437]	journal of neuroradiology	SAC	113	7	Italy	PO	2016-2017	6.00	None	None	**Safe**	**Effective**	No mention

© 2023 Volovici V et al. *JAMA Network Open.*

Cekirge, 2011[438]	J neurosurg	Coil	800	1	Turkey	RO	2001-2008	6.00	None	Yes	**Safe**	**Effective**	No mention
Choi, 2019[439]	J neurointervent surg	SAC	184	1	South Korea	RO	2008-2016		None	None	**Safe**	**Effective**	No mention
Choi, 2019[440]	J neurointervent surg	Coil	250	1	South Korea	RO	2004-2016		None	None	**Safe**	**Effective**	No mention
Chung, 2015[441]	J neurosurg	SAC	31	1	South Korea	RO	2008-2013		Yes	None	**Safe**	**Effective**	No mention
Chung, 2013[442]	clinical neurology and neurosurgery	Coil	72	1	UK	RO	2008-2010		NR	None	**Doubt**	**Doubt**	No mention
Clajus, 2013[443]	Interventional Neuroradiology	SAC	102	1	Germany	RO	2008-2010	13.60	NR	NS	**Safe**	**Effective**	No mention
Cottier, 2001[444]	AJNR Am J Neuroradiol	Coil	44	5	France	RO	1994-1999		NR	NS	**Safe**	**Effective**	No mention
Daglioglu, 2019[445]	Turk Neurosurg	FD	146	2	Turkey	RO		7.02	NR	NS	**Safe**	**Doubt**	No mention
Daou, 2015[446]	Neurosurgery	PED	33	1	USA	RO	2011-2014	11.40	NR	None	**Safe**	**Effective**	No mention
Fischer, 2015[447]	AJNR	FD	121	1	Germany	RO	2012-2014		Yes	Yes	**Safe**	**Effective**	No mention
Foa, 2018[448]	Interventional Neuroradiology	FD	246	4	Argentina	RO	2009-2017	12.00	None	None	**Safe**	**Effective**	No mention
Elias, 2003[449]	British Journal of Neurosurgery	Coil	30	1	UK	RO	1990-1998	32.60	NR	NS	Safe	Effective	Durable
Luo, 2007[450]	J Clin Neurosci	Coil	25	1	Taiwan	RO	1998-2005	13.00	Yes	NS	Safe	Effective	No mention
Malatesta, 2013[451]	Radiol Med	FD	28	1	Italy	PO	2009-2012		NR	None	Safe	Effective	No mention
Maldonado, 2011[452]	Am J Neuroradiol	SAC	68	1	France	RO	2003-2007		NR	NS	Safe	Effective	No mention
Goddard, 2002[453]	J Neurol Neurosurg Psychiatry	Coil	62	1	UK	RO	1994-2000		NR	NS	Safe	No mention	No mention

© 2023 Volovici V et al. *JAMA Network Open.*

Johnson, 2013[454]	J Neurointervent Surg	SAC	64	1	USA	RO	2003-2012	26.40	Yes	Yes	Safe	Effective	Doubt
Jia, 2012[455]	Intervent Neuroradiol	SAC	69	1	China	RO	2009-2011	33.00	Yes	None	Safe	Effective	No mention
Hur, 2015[456]	J Korean Neurosurg Soc.	Coil	134	1	South Korea	RO	2003-2013	49.70	NR	NS	Safe	Effective	No mention
Hwang, 2013[457]	J Clin Neurosci	SAC	116	1	South Korea	RO	2008-2010	6.00	None	None	Safe	Effective	No mention
Knap, 2017[458]	Pol J Radiol	Coil	60	1	Poland	PO	2010-2012		None	NS	Safe	Effective	No mention
Morais, 2017[459]	Neuroradiology	FD	39	2	Mutli-country	RO	2015-2016		NR	Yes	Safe	Effective	No mention
Herbreteau, 2016[460]	Am J Neuroradiol	WEB	39	1	France	PO	2012-2015	1.00	NR	NS	Safe	Effective	No mention
Kosty, 2019[461]	BR j Neurosurg	Clip	336	1	USA	RO	2005-2016	15.00	NR	None	Safe	Effective	Durable
Peker, 2017[462]	Turk Neurosurg	FD	30	1	Turkey	RO	2011-2016	9.30	NR	NS	Safe	Effective	No mention
Fiorella, 2016[463]	J Neurointervent Surg	SAC	31	6	Mutli-country	PO	2012-2013	6.00	Yes	Yes	Safe	Effective	Doubt
Deshmukh, 2006[464]	Neurosurgery	Wrap	63	1	USA	RO	1994-2003	44.10	NR	NS	Safe	Doubt	No mention
Doerfler, 2006[465]	Am J Neuroradiol	Coil	36	1	Germany	CS		6.00	NR	NS	Safe	Effective	No mention
Kim, 2010[466]	AJNR Am J Neuroradiol	Coil	69	1	Korea	RO	2000-2008	23.00	NR	NS	Safe	Doubt	No mention
Nossek, 2015[467]	J Neurosurg	PED	27	1	USA	RO	2011-2014	11.00	NR	None	Safe	Effective	No mention
Korkmazer, 2019[468]	Acta Neurochir	FD	133	1	Turkey	RO	2008-2013	12.00	NR	Yes	Doubt	Effective	Durable
Luo, 2018[469]	J Chin Med Assoc	Coil	42	1	Taiwan	RO	2010-2015	21.00	NR	None	Safe	Effective	No mention
Nanda, 2011[470]	Neurosurgery	Clip	80	1	USA	RO	1994-2009	27.38	None	None	Safe	Effective	No mention

© 2023 Volovici V et al. *JAMA Network Open.*

Nelson, 2011[471]	Am J Neuroradiol	PED	31	1	USA	CS		5.91	Yes	NS	Safe	Effective	No mention
Gonzalez, 2004[472]	Am J Neuroradiol	Coil	217	1	USA	RO	1991-2000		NR	NS	Safe	Effective	No mention
Gonzalez, 2008[473]	Stroke	Coil	181	1	USA	RO	1991-2005	9.70	NR	NS	Safe	Effective	No mention
Fanning, 2007[474]	Interventional Neuroradiology	Coil	94	1	Ireland	PO	2003-2004		NR	None	Safe	No mention	No mention
David, 1999[475]	J neurosurg	Clip	102	1	USA	RO	1988-1997		NR	NS	No mention	Doubt	Durable
Lee, 2011[476]	AJNR Am J Neuroradiol	Coil	74	1	South Korea	RO	2003-2010	14.70	NR	NS	Safe	Effective	No mention
Kwon, 2014[477]	Acta neurochir	Coil	2035	22	South Korea	CS	2007-2009		Yes	None	No mention	Effective	No mention
Link, 2018[478]	Interventional Neuroradiology	SAC	93	1	USA	RO	2002-2017		None	None	Safe	Effective	No mention
Kwon, 2005[479]	AJNR Am J Neuroradiol	Coil	25	1	South Korea	RO	2001-2004	7.90	NR	NS	Safe	Effective	No mention
Pandey, 2007[480]	Neurosurgery	Coil	275	1	USA	RO	1995-2005	22.50	NR	NS	No mention	Effective	No mention
Vallé, 2005[481]	Neuroradiology	Coil	62	5	France	RO	2002-2003		NR	NS	Safe	Doubt	No mention
Peluso, 2007[482]	Neuroradiology	Coil	33	1	Netherlands	RO	1995-2007	44.50	NR	None	Safe	Effective	No mention
Pardo, 2008[483]	Neuroradiol J	SAC	32	1	Spain	RO	2004-2005		NR	None	Safe	Effective	No mention
Pagiola, 2019[484]	J Neurointervent Surg	FD	30	1	Mutli-country	RO	2014-2018		None	None	Safe	Effective	No mention
Mohammadian, 2013[485]	Cerebrovasc dis	Coil	40	1	Iran	RO	2008-2012	6.00	NR	None	Doubt	Effective	Doubt
Darwish, 2003[486]	ANZ J Surg	Coil	40	1	New Zealand	RO	1997-2001		NR	NS	Safe	No mention	No mention
Gallas, 2008[487]	Am J Neuroradiol	Coil	321	5	France	PO	1998-2005		NR	NS	**Not safe**	Doubt	No mention

© 2023 Volovici V et al. *JAMA Network Open.*

Jeon, 2014[488]	Acta Neurochirg	SAC	25	1	South Korea	RO	2008-2012	30.00	NR	None	Safe	Doubt	Durable
Kim, 2016[489]	J Neurointervent Surg	Coil	33	1	South Korea	RO	2005-2013		NR	None	Safe	Effective	No mention
Kim, 2011[490]	Neurosurgery	Coil	70	1	South Korea	RO	2000-2009	25.00	Yes	None	Safe	Effective	No mention
Gunnarsson, 2007[491]	Can J Neurol Sci	Coil	28	1	Canada	PO	1992-2004	3.30	None	None	No mention	Effective	No mention
Mitra, 2007[492]	AJNR Am J Neuroradiol	Coil	77	1	UK	RO	2002-2004		NR	NS	Safe	No mention	No mention
Melake, 2010[493]	J Clin Neurosci	Coil	203		Japan	RO	2006-2007	9.30	NR	NS	Safe	Effective	No mention
Linfante, 2005[494]	Stroke	Coil	52	1	USA	PO	2002-2004	12.00	Yes	NS	Safe	No mention	No mention
Matouk, 2012[495]	Am J Neuroradiol	Occlusion	28	1	USA	RO	1992-2009	41.86	NR	NS	Doubt	Doubt	No mention
Cheung, 2018[496]	J Neurointervent Surg	SAC	59	1	Australia	RO	2012-2014	6.00	NR	None	Safe	Effective	Durable
Drazin, 2017[497]	J Neurointervent Surg	Coil	50	11	Mutli-country	RO	2003-2009	3.00	Yes	Yes	Safe	Effective	No mention
Oh, 2013[498]	J. Cerebrovasc. Endovasc Neurosurg.	Coil	28	1	South Korea	RO	2008-2012		NR	NS	Safe	No mention	No mention
Griessenauer, 2016[499]	J Neurosurg	PED	52	4	USA	RO	2009-2015	11.50	NR	NS	Safe	Effective	No mention
Griessenauer, 2017[500]	Neurosurgery	FD	127	2	USA	RO	2010-2015		None	None	Safe	Effective	No mention
Murayama, 1999[501]	J Neurosurg	Coil	115	1	USA	RO	1991-1998	16.30	NR	NS	Safe	Doubt	No mention
Müller, 2017[502]	Am J Neuroradiol	SAC	33	1	Germany	RO	2010-2015		NR	NS	Safe	Effective	No mention
Gupta, 2006[503]	Neuroradiol J	Coil	78	1	India	RO	1986-2004		NR	NS	Safe	Effective	No mention

© 2023 Volovici V et al. *JAMA Network Open.*

Lubicz, 2017[504]	Neuroradiology	SAC	50	1	Belgium	RO	2004-2015	50.20	None	None	Safe	Effective	Durable
Hallout, 2015[505]	World Neurosurg	Clip	251	1	France	RO	2002-2012	83.50	NR	None	Safe	Doubt	No mention
Chalouhi, 2013[506]	J Neurosurg	Clip	76	1	USA	RO	2001-2011	21.00	NR	Yes	Safe	Effective	Doubt
Chen, 2017[507]	Eur Rev Med Pharmacol Sci	SAC	57	1	China	RO	2012-2015		NR	None	Safe	Effective	No mention
Lv, 2009[508]	Neuroradiology	SAC	25	1	China	RO	1998-2005	23.50	NR	None	Safe	Effective	No mention
Lubicz, 2009[509]	Neuroradiology	SAC	32	1	Belgium	RO	2004-2008		NR	Yes	Safe	Effective	Doubt
Liu, 2019[510]	World Neurosurg	SAC	26	1	China	RO	2015-2017	12.40	Yes	Yes	Safe	Effective	No mention
Aghakhani, 2008[511]	Neurosurgery	Clip	325	2	France	RO	1996-2006		NR	NS	Safe	Effective	No mention
Grossberg, 2017[512]	J Neurointervent Surg	SAC	85	4	USA	RO			Yes	Yes	Safe	Effective	No mention
Kadkhodayan, 2015[513]	Neurosurgery	Endo	292	1	USA	RO	1996-2013		None	None	Safe	Effective	No mention
Ko, 2017[514]	J Korean Neurosurg Soc.	SAC	68	1	South Korea	RO	2009-2015	17.50	NR	NS	Safe	Effective	No mention
Kocur, 2016[515]	Neuroradiol J	Coil	28	1	Poland	RO	2008-2014	23.70	None	None	Doubt	Effective	No mention
Lukić, 2011[516]	Jap J Rad	Coil	108	1	Serbia	CS	2007-2009		NR	NS	No mention	Effective	No mention
Moscato,2013[517]	Neuroradiol J	Coil	182	1	Italy	RO	2000-2011		NR	NS	Doubt	Effective	No mention
Griffin, 2019[518]	World Neurosurg	PED	174	1	USA	RO	2014-2018	11.00	NR	NS	Safe	No mention	No mention
Gory, 2017[519]	Neurosurgery	WEB	40	1	Mutli-country	RO	2012-2014	11.90	NR	NS	Safe	No mention	No mention
Ota, 2018[520]	World Neurosurg	Clip	159	1	Japan	RO		6.00	NR	None	No mention	Effective	No mention

© 2023 Volovici V et al. *JAMA Network Open.*

Meyers, 2019[521]	Stroke	FD	180	26	USA	PO	2012-2015	12.00	Yes	NS	Safe	Effective	No mention
D'Agostino, 2009[522]	Surg Neurol	Coil	86	1	USA	RO	2005-2006		NR	NS	Doubt	Doubt	No mention
Chalouhi, 2013[523]	Stroke	SAC	508	1	USA	RO	2006-2011		NR	NS	Safe	Effective	No mention
Martínez-Galdámez, 2015[524]	J Neurointervent Surg	PED	25	1	Mutli-country	RO		6.00	NR	None	Safe	Effective	No mention
Lylyk, 2002[525]	J Neurosurg	SAC	123	1	Argentinia	RO	1996-2001	6.70	None	None	Safe	No mention	No mention
Castro, 2008[526]	Interventional Neuroradiology	Coil	47		Spain	PO	2005-2007	6.00	None	None	No mention	No mention	No mention
Gao, 2018[527]	World neurosurg	Coil	75	1	China	RO	2013-2015		NR	None	Safe	Effective	No mention
Ghinda, 2015[528]	Interventional Neuroradiology	SAC	62	1	Canada	RO	2003-2012	53.00	None	None	No mention	No mention	No mention
Guan, 2016[529]	Journal of stroke and cerbrovascular disease	Endo	147	1	China	RO	2011-2014	12.00	NR	NS	Safe	Effective	No mention
Henkes, 2005[530]	J neurosurg	Coil	314	1	Germany	RO	1992-2005		NR	NS	Safe	Effective	No mention
Jeon, 2016[531]	AJNR Am J Neuroradiol	BAC + SAC	62	2	South Korea	RO	2007-2014		NR	NS	Safe	Effective	No mention
Henkes, 2004[532]	Neurosurgery	Coil	1579	1	Germany	RO	1992-2003		NR	Yes	Safe	Effective	No mention
Liu, 2018[533]	World Neurosurg	Bypass	35	1	Mutli-country	CS	2006-2016	40.60	Yes	Yes	Doubt	Doubt	No mention
Li, 2016[534]	J Korean Neurosurg Soc.	SAC	31	1	China	RO	2010-2014		NR	NS	Doubt	Doubt	No mention
Jeong, 2015[535]	J. cerebrovasc. endovasc. neurosurg.	SAC	50	1	South Korea	RO	2011-2014	12,00	Yes	None	Safe	Effective	No mention
Petr, 2016[536]	Am J Neuroradiol	FD	310	1	Mutli-country	PO	2009-2015	23.40	Yes	Yes	Safe	Effective	No mention

© 2023 Volovici V et al. *JAMA Network Open.*

Iosif, 2014[537]	J Neurointervent Surg	Coil	59	1	France	RO	2002-2011	6.00	NR	None	Safe	Effective	No mention
Kähärä, 1999[538]	Acta Neurol Scand	Coil	44	1	Finland	RO	1997-1997	26.00	NR	NS	Safe	Effective	No mention
Kühn, 2019[539]	J Clin Neurosci	PED	260	1	USA	RO	2011-2017		Yes	Yes	Safe	Effective	No mention
Fu, 2018[540]	Int J Clin Exp Med	SAC	116	1	South Korea	RO	2008-2010	6.00	NR	None	Safe	Effective	No mention
Chalouhi, 2012[541]	ajnr	Endo	87	1	USA	RO	2006-2011		NR	NS	Safe	Effective	No mention
Debrun, 1998[542]	Neurosurgery	Coil	144	1	USA	RO	1994-1997		NR	NS	Safe	No mention	No mention
Liu, 2017[543]	Medicine	Coil	82	1	China	RO	2004-2015	28.00	NR	NS	Safe	Effective	No mention
Deng, 2011[544]	Neurology India	Endo	38	1	China	RO	2008-2010	12.10	None	None	Safe	Effective	No mention
Dinc, 2013[545]	Interventional radiology	Coil	481	1	Turkey	RO	1998-2011		NR	None	Safe	Effective	No mention
Duvuru, 2018[546]	Asian Journal of Neurosurgery	Clip	224	1	Japan	RO	2014-2016		None	None	Safe	No mention	No mention
Fang, 2010[547]	Annals of Vascular Surgery	Coil	19	1	China	RO	2004-2007	13.30	NR	NS	No mention	Effective	No mention
Finitsis, 2010[548]	Interventional Neuroradiology	Endo	280	1	France	RO	1992-2001	37.08	NR	NS	No mention	Effective	No mention
Koźba-Gosztyła, 2016[549]	Adv Clin Exp Med	Clip	104	1	Poland	RO	2005-2013	78.00	NR	None	Safe	Effective	No mention
Grasso, 2015[550]	Surg Neurol Intl	Clip	53	1	Italy	RO	2008-2014		Yes	None	No mention	Effective	No mention
Ji, 2016[551]	Neurol Neurochir Pol	SAC	108	1	China	RO	2008-2012	25.80	Yes	None	Doubt	Doubt	No mention
Gao, 2018[552]	Acta Med Mediterr	SAC	68	1	China	RO	2008-2014		NR	Yes	Safe	Effective	Durable
Liu, 2015[553]	J Neuroradiol	SAC	59	1	China	RO	2009-2013	26.90	Yes	None	Safe	Effective	No mention

Ishida, 2016[554]	Surg Neurol Intl	Clip	150	1	Japan	RO	2010-2015		Yes	None	Safe	No mention	No mention
Kim, 2013[555]	J Cerebrovasc Endovasc Neurosurg	SAC	39	1	South Korea	CS	2009-2011		NR	NS	No mention	Effective	No mention
Mordasini, 2005[556]	Am J Neuroradiol	Coil	46	1	Switserland	RO	1993-2003	39.60	NR	NS	Safe	Effective	No mention
Kocur, 2016[557]	Neurol Neurochir Pol	SAC	34	1	Poland	CS	2008-2014	14.60	None	None	Safe	Effective	Durable
Lempert, 2000[558]	Stroke	Coil	112	1	USA	RO	1991-1998	9.50	NR	NS	Safe	Effective	No mention
Gupta, 2007[559]	J Int Med Sci Acad	Coil	27	1	India	RO		12.00	NR	NS	Safe	Effective	No mention
Lee, 2009[560]	Intervent Neuroradiol	Coil	142	1	South Korea	RO	2005-2006	8.20	NR	NS	No mention	Effective	No mention
Jeon, 2014[561]	AM J Neuroradiol	Coil	167	1	South Korea	RO	2003-2012	35.80	Yes	NS	Safe	Effective	No mention
Juszkat, 2006[562]	Pol J Radiol	Coil	129	1	Poland	RO	2005-2005		NR	NS	Doubt	Effective	No mention
Higashida, 2006[563]	Intervent Neuroradiol	Coil	112	23	USA	RO	2000-2002		Yes	NS	Safe	Effective	No mention
Killer-Oberpfalzer, 2018[564]	Am J Neuroradiol	FD	531	15	Multi-country	RO	2012-2015	6.60	NR	NS	Safe	Effective	No mention
Matano, 2016[565]	World Neurosurg	Clip	127	1	Japan	RO	2012-2014		NR	None	Doubt	Effective	No mention
Kuether, 1998[566]	Neurosurgery	Coil	74	1	USA	RO	1992-1996	26.40	NR	NS	Safe	Effective	No mention
Lubicz, 2017[567]	Neuroradiology	SAC	49	1	Belgium	RO	2007-2015		NR	NS	Safe	Effective	Durable
Iosif, 2017[568]	Am J Neuroradiol	FD	58		Mutli-country	RO	2010-2014	6.00	NR	NS	Doubt	Effective	No mention
Khaldi, 2012[569]	J. vasc. Interv. Radiol. Neurol.	Coil	57	5	USA	RO	2010-2010		NR	NS	Safe	Doubt	No mention

© 2023 Volovici V et al. *JAMA Network Open.*

Gao, 2010[570]	Neurol India	SAC	71	2	China	RO	2003-2007	37.10	NR	None	Safe	Effective	No mention
Karanam, 2012[571]	J Clin Diagn Res	Coil	25	1	India	RO	2003-2008	24.00	None	None	Safe	Effective	No mention
Kaku, 1999[572]	Intervent Neuroradiol	Coil	28	1	Japan	RO	1994-1998	24.80	NR	NS	Safe	No mention	No mention
Liang, 2010[573]	Neurol Res	SAC	107	1	China	RO	2003-2006	47.30	NR	NS	Safe	Effective	No mention
Negoro, 1998[574]	Intervent Neuroradiol	Coil	109	1	Japan	RO	1982-1998		NR	NS	Safe	Effective	No mention
Mu, 2008[575]	Chin Med J	Coil	42	1	China	RO	2004-2006	8.60	NR	NS	Safe	Effective	No mention
Gory, 2014[576]	Neurosurgery	SAC	64	7	Mutli-country	PO	2009-2010	6.00	NR	Yes	Safe	Effective	No mention
Murayama, 1999[577]	Intervent Neuroradiol	Coil	115	1	USA	RO	1991-1998	16.30	NR	NS	Safe	Effective	No mention
Moret, 1996[578]	Neuroradiology	Coil	36	1	France	CS	1992-1994		NR	NS	No mention	Effective	No mention
Nakai, 2006[579]	Intervent Neuroradiol	Coil	71	1	Japan	CS	1997-2004		NR	NS	No mention	Effective	No mention
Juszkat, 2007[580]	Intervent Neuroradiol	SAC	28	1	Poland	CS	2004-2006		NR	NS	Safe	Effective	No mention
Kim, 2012[581]	Neurointervention	Coil	126		South Korea	PO	2008-2009		NR	NS	Safe	Effective	No mention
Lubicz, 2015[582]	Am J Neuroradiol	FD	58	2	Belgium	RO	2009-2014	22.00	NR	NS	Safe	Effective	Durable
Heller, 2014[583]	J Neurosurg	SAC	104	1	USA	RO	2006-2011		Yes	Yes	Safe	Doubt	No mention
Gory, 2017[584]	Intervent Neuroradiol	Coil	163	7	France	RO	2011-2014	12.50	Yes	Yes	Safe	Effective	No mention
Li, 2009[585]	Radiology	SAC	31	1	China	PO	2005-2008	27.00	Yes	NS	No mention	Effective	No mention
Nagatani, 1998[586]	Neurol Med-Chir	Clip	64	1	Japan	RO	1996-1996		NR	NS	Safe	Effective	No mention

© 2023 Volovici V et al. *JAMA Network Open.*

Panagiotopoulos, 2011[587]	Am J Neuroradiol	Coil	30	1	Germany	RO	1999-2008	19.00	NR	NS	Safe	Effective	No mention
Piano, 2019[588]	J Neurosurg	FD	162	30	Italy	PO	2013-2014	12.00	NR	NS	Safe	Effective	No mention
Moon, 2014[589]	Neurol Res	PED	29	1	USA	RO	2011-2012		None	None	Safe	Effective	No mention
Matsumaru, 1999[590]	Intervent Neuroradiol	Coil	42	1	Japan	RO	1997-2008		None	None	Safe	Doubt	No mention
Liang, 2019[591]	Neurointervention	PED	48	1	USA	RO	2012-2017	12.06	NR	NS	Safe	Effective	No mention
Qi, 2008[592]	Chin Med J	Clip	170	1	China	RO	1995-2007	32.00	NR	NS	No mention	Effective	No mention
Qin, 2017[593]	Medicine	SAC	37	1	China	CS	2012-2016	10.00	None	None	Safe	Effective	No mention
Krisht, 2007[594]	Neurosurgery	Clip	51	1	USA	PO	1998-2006	33.00	NR	NS	Safe	No mention	Durable
Sorimachi, 2012[595]	Neurol Res	Coil	138	3	Japan	RO	1998-2010		NR	NS	Safe	Effective	No mention
Turek, 2016[596]	Neurol Neurochir Pol	Coil	190	1	Poland	RO	2006-2013		Yes	None	Safe	Effective	No mention
Tang, 2011[597]	Acta Neurochir Suppl	Clip	120	1	China	RO	2000-2008		Yes	None	No mention	Effective	No mention
Sonobe, 2001[598]	Intervent Neuroradiol	Coil	93	1	Japan	RO	1997-2000		NR	NS	Safe	No mention	No mention
Rathore, 2012[599]	Neurol India	Occlusion	27	1	India	RO	2001-2010	28.80	None	None	Safe	Effective	No mention
Sluzewski, 2003[600]	Radiology	Coil	160	1	Netherlands	RO	1995-2000	36.00	NR	NS	Safe	Effective	No mention
Jafar, 2002[601]	Neurosurgery	Bypass	29	1	USA	RO	1990-1999	62.00	NR	NS	Safe	Effective	No mention
Kaku, 2003[602]	Intervent Neuroradiol	Coil	175	1	Japan	RO	1994-2001	24.80	NR	NS	Safe	No mention	No mention
Kallmes, 2016[603]	Intervent Neuroradiol	PED	191	28	Mutli-country	PO		6.20	Yes	None	Safe	Effective	No mention

© 2023 Volovici V et al. *JAMA Network Open.*

Watanabe, 2014[604]	Surg Neurol Intl	Coil	51	1	Japan	RO	2006-2010	3.00	NR	NS	Doubt	Effective	No mention
Yaltirik Bilgin, 2017[605]	Turk Neurosurg	FD	61	1	Turkey	RO			NR	NS	Safe	Effective	No mention
Huang, 2010[606]	Neurol India	Coil	41	1	China	RO	1998-2008	27.50	Yes	None	Safe	Effective	No mention
Hayakawa, 2000[607]	J Neurosurg	Coil	173	1	USA	RO	1990-1998		NR	NS	No mention	Effective	No mention
Larson, 1995[608]	Neurosurgery	Coil	60	1	USA	RO	1977-1992	76.00	NR	NS	Doubt	Effective	No mention
Liu, 2016[609]	Neurol India	SAC	218	1	China	RO	2011-2014	29.10	None	None	No mention	Effective	No mention
Liu, 2016[610]	Neurol India	Coil	38	1	China	RO	2011-2014	26.60	Yes	None	No mention	Effective	No mention
Gunia, 2017[611]	Georgian Med News	Coil	412	1	Georgia	RO	2011-2016		NR	NS	Doubt	Effective	No mention
Lubicz, 2015[612]	Am J Neuroradiol	FD	58	2	Belgium	RO	2009-2014	22.00	NR	NS	Safe	Effective	Durable
Vendrell, 2009[613]	Radiology	Coil	153	1	France	RO	1999-2006		NR	NS	Safe	No mention	Doubt
Pereira-Filho, 2014[614]	Neuropschycolog y	Clip	40	1	Brazil	PO	2005-2009	36.00	NR	NS	Safe	Effective	Durable
Yu, 2012[615]	Radiology	PED	143	11	Hong Kong	PO	2008-2011		Yes	Yes	Safe	Effective	No mention
Lv, 2009[616]	Neuroradiology	SAC	51	1	China	RO	2005-2007	14.50	NR	NS	Safe	Effective	No mention
Adeeb, 2018[617]	Neurosurgery	SAC	74	3	Mutli-country	RO	2007-2015	11.00	None	None	Safe	Effective	No mention
Iosif, 2015[618]	J Neurosurg	FD	38	1	France	PO	2012-2013	3.00	NR	None	Safe	Effective	No mention
Wilson, 2015[619]	Medicine	Coil	80	1	Australia	RO	1995-2003	67.20	None	None	No mention	Effective	Durable
Jabbour, 2013[620]	Neurosurgery	PED	109	1	USA	RO	2010-2012		None	Yes	Safe	Effective	No mention

© 2023 Volovici V et al. *JAMA Network Open.*

Wang, 2017[621]	Int J Clin Exp Med	Coil	56	1	China	PO	2011-2014	49.00	Yes	None	Doubt	Effective	No mention
Zhu, 2017[622]	Biomed Res	SAC	51	1	China	RO	2004-2014		NR	NS	Safe	Doubt	Durable
Matsukawa, 2018[623]	J Neurosurg	Clip	702	1	Japan	RO	2012-2015	12.00	NR	None	Safe	Effective	Durable
Vallee, 2003[624]	Radiology	Coil	53	1	France	RO		24.00	NR	NS	Safe	Effective	No mention
Abud, 2010[625]	Arq Neuropsiquiatr	Endo	106	1	Brazil	PO	2005-2008	22.00	NR	NS	Safe	No mention	No mention
Murias Quintana, 2016[626]	Intervent Neuroradiol	Coil	50	1	Mutli-country	RO	2008-2012	1.00	None	None	Safe	Effective	No mention
Mori, 2017[627]	J Neurosurg	Clip	260	2	Japan	RO	2005-2015	12.00	NR	None	Safe	Effective	Durable
Adeeb, 2017[628]	Am J Neuroradiol	PED	329	3	USA	RO	2009-2016		None	None	No mention	No mention	No mention
Zaidat, 2019[629]	Front Neurol	Coil	148	12	USA	PO	2013-2014	5.90	Yes	Yes	Safe	Effective	Durable
Lowe, 2018[630]	Neurosurgery	Coil	158	1	USA	RO			Yes	Yes	Safe	No mention	No mention
Labeyrie, 2015[631]	AJNR Am J Neuroradiol	Occlusion	56	1	France	RO	2004-2013	45.00	NR	NS	Safe	Effective	No mention
Juszkat, 2016[632]	Intervent Neuroradiol	Coil	38	1	Poland	RO	2004-2014	27.70	None	None	Doubt	Effective	No mention
Iosif, 2018[633]	J Neurosurg	Endo	128	1	France	PO	2012-2013	6.00	NR	None	Safe	Effective	No mention
Debrun, 1998[634]	Neurol Med Chir	Coil	139	1	USA	CS	1994-1997		NR	NS	Safe	Effective	No mention
Jo, 2015[635]	Neurosurg Rev	Clip	610	1	South Korea	RO	2008-2012		NR	NS	Safe	Effective	No mention
Akgul, 2020[636]	Neurosurg	FD	43	1	Turkey	RO			NR	NS	Safe	Effective	No mention
Baek,2018[637]	Acta Neurochir	Coil	52	4	Korea	RO	2003-2014	31.60	NR	None	Safe	Effective	No mention

© 2023 Volovici V et al. *JAMA Network Open.*

Duckwiler, 1999[638]	Intervent Neuroradiol	Coil	510	1	USA	RO	1990-1998		NR	NS	Safe	No mention	No mention
Miyazawa, 1999[639]	Neurol Med -Chir	Clip	68	1	Japan	RO	1986-1997		NR	NS	No mention	No mention	No mention
Lubicz, 2006[640]	Neurocrit Care	Coil	25	1	Belgium	PO	2004-2005		NR	NS	Doubt	Effective	No mention
Bradac, 2007[641]	Neurosurg Rev	Coil	533	1	Italy	RO	1994-2005		NR	NS	Doubt	Doubt	No mention
Wallace, 2019[642]	Intervent Neuroradiol	Coil	219	1	USA	RO	2012-2018		None	Yes	Doubt	Doubt	No mention
Spiotta, 2013[643]	J Neurointervent Surg	Coil	48	1	USA	RO	2011-2012		NR	None	Doubt	Effective	No mention
Nishi, 2019[644]	Neurol Med Chir	Occlusion	274		Japan	RO	2010-2014	1.00	Yes	None	Not safe	Effective	No mention
Takemoto, 2014[645]	J Neurointervent Surg	SAC	31	1	USA	RO	1995-2012		NR	None	Doubt	Effective	No mention
Gunnarson, 2009[646]	AJNR Am J Neuroradiol	Coil	187	1	Canada	RO	2002-2005		NR	NS	Doubt	Doubt	Doubt
Van Rooij, 2009[647]	Am J Neuroradiol	Coil	187	1	Netherlands	RO	1995-2008	6.00	NR	NS	Doubt	Effective	No mention
Shi, 2013[648]	Acta Neurochir	Clip	41	1	China	RO	2001-2011		Yes	NS	No mention	Effective	No mention
Pierot, 2008[649]	Stroke	Endo	649	27	Mutli-country	PO	2005-2006		Yes	None	Doubt	Effective	No mention
Norbäck, 2005[650]	Neuroradiology	Coil	239	1	Sweden	RO	1996-2000		Yes	None	No mention	No mention	No mention
Moon, 2015[651]	J Neurointerv Surg	Coil	99	1	USA	PO	2004-2012		None	None	Safe	Effective	No mention
Mehta, 2011[652]	Neuroradiol J	Coil	34	1	India	RO	2000-2001	6.00	NR	NS	No mention	Effective	No mention
Lum, 2012[653]	J Neurointervent Surg	Coil	34	1	Canada	RO	2003-2008		None	None	Doubt	Effective	Durable
Lim, 2013[654]	Neurosurgery	SAC	34	9	South Korea	PO	2006-2012	32.00	NR	NS	No mention	Effective	No mention

© 2023 Volovici V et al. *JAMA Network Open.*

Kang, 2018[655]	J Neurosurg	Clip	198	1	USA	PO	1997-2016		NR	None	No mention	Effective	Durable
Johansson, 2004[656]	Neuroradiology	Coil	62	1	Sweden	RO	1996-2000	6.00	Yes	NS	Doubt	Effective	No mention
Jia, 2018[657]	Acta Neurochir (Wien)	Coil	34	1	South Korea	RO	2016-2017	6.00	None	None	Safe	Doubt	No mention
Jankowitz, 2011[658]	J Neuroimaging	Endo	32	1	USA	RO	2003-2007	6.00	Yes	NS	No mention	Doubt	Durable
Arthur, 2019[659]	J Neurointervent Surg	WEB	150	27	Mutli-country	PO		12.00	Yes	Yes	Safe	Effective	No mention
Atasoy, 2019[660]	AM J Neuroradiol	PED	41	1	Mutli-country	RO	2016-2018	12.00	Yes	NS	Doubt	Doubt	No mention
Iskandar, 2011[661]	Intervent Neuroradiol	Endo	107	1	Denmark	RO	1999-2009	9.00	NR	NS	No mention	Doubt	Doubt
Byrne, 1995[662]	Br J Neurosurg	Coil	50	1	UK	RO	1992-1993	12.00	NR	NS	No mention	No mention	No mention
Daglioglu, 2020[663]	Turk Neurosurg	PED	146	2	Turkey	RO		7.02	NR	NS	Safe	Effective	No mention
Im, 2009[664]	Am J Neuroradiol	Coil	370	2	South Korea	RO	2002-2006	21.50	NR	NS	Doubt	Doubt	No mention
Hwang, 2011[665]	Neuroradiology	Coil	38	1	South Korea	RO	2005-2009	14.80	NR	None	Safe	Effective	No mention
Hirota, 2007[666]	Neuroradiol J	Endo	59	1	France	RO	1999-2005		NR	None	No mention	Doubt	No mention
Gallas, 2009[667]	Am J Neuroradiol	Coil	929	5	France	PO	1998-2003		NR	NS	Safe	Effective	Durable
Goertz, 2019[668]	Clin Neurorad	SAC	98	3	Germany	RO	2013-2018	16.00	None	Yes	Safe	Effective	No mention
Almatter, 2020[669]	Clin Neurorad	FD	45	1	Mutli-country	RO	2010-2018	12.00	NR	None	Doubt	Doubt	No mention
Almatter, 2020[670]	CVIR Endovasc	FD	74	1	Mutli-country	RO	2016-2019		None	Yes	Safe	Effective	No mention
Hagen, 2021[671]	Cardiovasc Intervent Radiol	Endo	118	2	Germany	RO	2008-2017		None	Yes	Safe	Effective	No mention

© 2023 Volovici V et al. *JAMA Network Open.*

Guimaraens, 2020[672]	J Neurointervent Surg	FD	150	3	Spain	PO	2015-2018	18.99	None	None	Safe	Effective	No mention
Griffin, 2020[673]	Neurosurg Rev	FD	29	1	USA	RO	2012-2019	17.40	NR	NS	No mention	Effective	No mention
Gallas, 2005[674]	Am J Neuroradiol	Coil	650	1	France	RO	1998-2003	36.00	NR	NS	Doubt	Effective	No mention
Andrade-Barazarte, 2015[675]	Neurosurgery	Clip	30	1	Finland	RO	1957-2012	3.00	NR	None	Doubt	Doubt	No mention
Arslan, 2021[676]	Neuroradiology	SAC	112	2	Germany	RO	2015-2019		None	None	Safe	Effective	No mention
Aguilar-Perez, 2021[677]	Open Neurosurg (Hagerstown)	FD	530	1	Germany	RO	2011-2019		None	None	Safe	Effective	Durable
Aydin, 2020[678]	Neurosurgery	SAC	30	1	Turkey	RO			NR	None	Safe	Effective	Durable
Baek, 2020[679]	J Neurosurg	SAC	51	1	South Korea	RO	2018-2018	7.40	Yes	None	Doubt	Doubt	No mention
Bhogal, 2019[680]	Clin Neuroradiol	FD	30	1	Germany	RO	2009-2017		NR	Yes	Safe	Effective	No mention
Kwon, 2021[681]	J. Korean Neurosurg Soc	SAC	123	1	South Korea	RO	2018-2019	12.40	NR	None	Safe	Effective	No mention
Kim, 2008[682]	Neuroradiology	Coil	37	4	South Korea	RO	1999-2006	27.00	NR	None	Doubt	Doubt	No mention
Patzig, 2017[683]	Cardiovasc Intervent Radiol	FD	25	1	Germany	RO	2011-2013		NR	None	No mention	No mention	No mention
Lee, 2020[684]	J. Cerebrovasc. Endovasc. neurosurg.	Coil	39	1	South Korea	RO	2007-2019		NR	None	No mention	No mention	No mention
Bradač, 2005[685]	Neuroradiology	Coil	61	1	Italy	RO	1994-2004	50.40	NR	NS	No mention	Doubt	No mention
Daniel, 2020[686]	BMC Neurol	Coil	49	1	Mutli-country	RO	2016-2018	6.00	Yes	Yes	Safe	Effective	No mention
Maurer, 2019[687]	Am J neuroradiol	WEB	117	2	Germany	RO	2017-2019		Yes	NS	Safe	Doubt	No mention

© 2023 Volovici V et al. *JAMA Network Open.*

Binh, 2020[688]	Heliyon	FD	130	2	Mutli-country	RO	2012-2017		None	None	Safe	Effective	No mention
Wang, 2019[689]	Neuroradiology	Coil	42	1	China	RO	2011-2017	24.30	Yes	None	Safe	Effective	No mention
Weir, 2003[690]	AJNR Am J Neuroradiol	Coil	27	1	USA	RO	1995-2001		NR	NS	No mention	Doubt	No mention
Teo, 2015[691]	Br J Neurosurg	Clip	47	1	UK	RO	2012-2013		NR	None	No mention	Doubt	No mention
Strauss, 2016[692]	Acta Neurochir	FD	60	1	Israel	RO	2008-2013	15.00	None	None	No mention	Effective	No mention
Shankar, 2016[693]	J Neurointervent Surg	FD	92	8	Canada	RO	2009-2013	12.00	NR	Yes	No mention	No mention	No mention
Rodríguez-Hernández, 2013[694]	Neurosurgery	Clip	543	1	USA	RO	1997-2010		NR	NS	No mention	No mention	No mention
Kim, 2018[695]	J. cerebrovasc. endovasc. neurosurg.	Coil	143	2	South Korea	RO	2012-2013	39.50	NR	None	No mention	Doubt	No mention
Choi, 2019[696]	Yeungnam univ. j. med.	Coil	67	1	South Korea	RO	2004-2015	29.00	Yes	None	No mention	Effective	No mention
Ioannidis, 2010[697]	J Neurosurg	Coil	94		UK	CS	1995-2006	23.00	NR	None	Doubt	Doubt	No mention
Zhou, 2020[698]	Front Neurol	SAC	42	1	China	RO	2016-2018	24.40	NR	None	Safe	Effective	No mention
Zhou, 2020[699]	World Neurosurg	Endo	185	1	China	RO	2006-2016		Yes	None	Doubt	Doubt	No mention
Jiang, 2020[700]	J Coll Phys Surg Pak	Clip	38	1	China	RO	2013-2018	12.00	Yes	None	Safe	Effective	No mention
Zheng, 2019[701]	World Neurosurg	Clip	217	1	China	RO	2013-2017		Yes	NS	No mention	Doubt	No mention
Gudelj, 2020[702]	J Belg Soc Radiol	SAC	33	1	Belgium	RO	2016-2018		NR	None	Doubt	Doubt	No mention
Cui, 2015[703]	Eur Rev Med Pharmacol Sci	SAC	65	1	China	RO			NR	None	Safe	Effective	No mention

© 2023 Volovici V et al. *JAMA Network Open.*

Zhao, 2006[704]	J Clin Neurosci	Clip	60	1	China	RO	1994-2002		NR	NS	Doubt	Doubt	No mention
Linfante, 2020[705]	Cureus	Coil	45	3	Mutli-country	RO	2008-2016	3.00	None	Yes	Doubt	Doubt	Doubt
Zhang, 2020[706]	Stroke Vasc Neurol	Endo	27	1	China	RO	2009-2018		Yes	None	Doubt	Effective	No mention
Zhang, 2020[707]	J Intervent Med	Coil	390	1	China	RO	2004-2015	30.00	NR	None	No mention	Doubt	Durable
Zaidat, 2020[708]	Stroke	SAC	182	25	USA	PO	2015-2016	12.00	Yes	Yes	Doubt	Effective	No mention
Yue, 2020[709]	BMC Neurol	Coil	199	1	China	RO	2012-2019	6.00	Yes	None	Safe	Doubt	No mention
Youssef, 2020[710]	J Neurointerv Surg	WEB	48	3	USA	RO	2014-2020	1.00	Yes	Yes	Safe	Effective	No mention
Yeomans, 2020[711]	Neuroradiol J	PED	31	1	UK	PO	2017-2019		None	None	Doubt	Doubt	No mention
Yan, 2020[712]	Intervent Neuroradiol	SAC	57	1	China	RO	2014-2018	13.60	Yes	None	Safe	Effective	No mention
Yan, 2020[713]	J Clin Neurosci	SAC	46	1	China	RO	2015-2018		Yes	None	No mention	Doubt	No mention
Caragliano, 2020[714]	J Neuroradiol	SAC	113	7	Italy	RO	2016-2017	6.00	None	None	Safe	Effective	No mention
Xue, 2019[715]	World Neurosurg	SAC	26	1	China	RO	2010-2018	22.20	Yes	NS	Safe	Effective	Durable
Wongsuriyanan, 2020[716]	World Neurosurg	Clip	25	1	Thailand	RO	2016-2018	3.00	None	None	Safe	Effective	No mention
Winkler, 2019[717]	Oper Neurosurg	Clip	146		USA	RO	1998-2015		NR	None	Safe	Effective	No mention
Weinberg, 2020[7198]	World Neurosurg	Coil	35	1	USA	RO	2019-2019		NR	Yes	Safe	Effective	No mention
Waqas, 2020[719]	Interv	FD	32	1	USA	RO	2016-2019		None	Yes	Safe	Effective	No mention
Wang, 2020[720]	Clin Neurol Neurosurg	SAC	101	4	China	RO	2017-2017	15.90	Yes	None	Safe	Effective	No mention

© 2023 Volovici V et al. *JAMA Network Open.*

Wang, 2020[721]	Intervent Neuroradiol	Endo	277	1	China	RO	2009-2013		None	None	Safe	Effective	No mention
Van Rooij, 2000[722]	Neurosurgery	BAC	29	1	Netherlands	RO	1993-1999	21.00	NR	NS	Safe	Effective	No mention
Van Rooij, 2008[723]	Neuroradiology	Coil	46	1	Netherlands	RO	1995-2007	24.10	NR	None	Safe	Effective	No mention
Sato, 2020[724]	Intervent Neuroradiol	Coil	46	1	Japan	RO	2008-2019		None	None	No mention	Doubt	No mention
Vargas, 2010[725]	Biomedica (Bogota)	Endo	473	12	Colombia	RO	1996-2008		None	None	Doubt	Doubt	No mention
Ulutas, 2021[726]	J Neurosurg	Clip	54	1	Turkey	PO	2016-2018	12.10	NR	None	Safe	Effective	No mention
Trivelato, 2020[727]	Neurosurgery	PED	151	7	Brasil	PO	2017-2018		NR	None	Safe	Effective	No mention
Tjahjadi, 2016[728]	World Neurosurg	Clip	31	1	Finland	RO	1998-2004	6.20	NR	None	No mention	No mention	No mention
Tian, 2020[729]	Front Neurol	Coil	504	4	China	RO	2014-2015	13.60	Yes	None	No mention	Effective	No mention
Thavara, 2020[730]	Asian J Neurosurg	Clip	27	1	Japan	RO	2017-2019		None	None	No mention	No mention	No mention
Taschner, 2020[731]	Neurointerv Surg	PED	96	12	Germany	PO	2014-2018	14.80	Yes	Yes	Safe	Effective	No mention
Srinivasan, 2020[732]	J Neurosurg	SAC	54	13	USA	PO			NR	Yes	Safe	Effective	No mention
Bhogal, 2019[733]	Clin Neuroradiol	FD	69	1	Germany	RO	2009-2018	18.00	None	Yes	Doubt	Doubt	No mention
Song, 2020[734]	Korean J Radiol	SAC	50	1	South Korea	PO	2016-2018	6.00	Yes	Yes	Safe	Effective	No mention
Sirakov, 2020[735]	Neurosurgery	SAC	118	1	Bulgaria	RO	2017-2018	6.00	NR	None	Safe	Doubt	No mention
Sharma, 2020[736]	Asian J Neurosurg	Clip	37	1	Japan	RO	2015-2019		None	None	Safe	Doubt	No mention
Shanno, 2001[737]	Neurosurgery	Coil	42	1	USA	RO	1995-1999		NR	NS	No mention	No mention	No mention

© 2023 Volovici V et al. *JAMA Network Open.*

Schob, 2020[738]	Front Neurol	FD	32	1	Germany	PO	2019-2019		None	Yes	Safe	Effective	No mention
Satow, 2020[739]	Neurol Med -Chir	Coil	6619		Japan	RO	2010-2014		Yes	Yes	No mention	Doubt	No mention
Sato, 2020[740]	World Neurosurg	SAC	38	1	Japan	RO	2015-2019	22.90	NR	None	No mention	Effective	No mention
Sai Kiran, 2020[741]	World Neurosurg	Clip	157	1	India	RO	2013-2016	9.60	NR	NS	Doubt	Doubt	No mention
Russo, 2020[742]	J Neuroradiol	SAC	61	8	Italy	RO	2017-2018	3.00	None	NS	No mention	Effective	No mention
Ross, 2008[743]	Neurosurgery	Endo	27	1	France	PO	1991-1998		NR	NS	Safe	Effective	No mention
Ross, 2006[744]	Surg Neurol	BAC	56	1	USA	PO	2002-2003		NR	NS	Doubt	Doubt	No mention
Rodríguez-Hernández, 2013[745]	World Neurosurg	Clip	140	1	USA	RO			NR	None	No mention	Doubt	No mention
Rice, 2020[746]	J Neurointervent Surg	PED	204	21	Mutli-country	PO			Yes	Yes	Safe	Effective	No mention
Renowden, 2009[747]	Clin Neurol Neurosurg	Coil	711	1	UK	RO	1996-2005	17.00	NR	NS	Doubt	Effective	No mention
Pop, 2020[748]	Intervent Neuroradiol	BAC	120	1	France	RO	2016-2018		None	None	No mention	Effective	No mention
Poncyljusz, 2020[749]	J Clin Med	SAC	162	4	Poland	RO	2013-2019	3.00	None	Yes	Safe	Effective	Doubt
Poncyljusz, 2020[750]	J Clin Med	SAC	30	1	Poland	RO	2020-2020		None	None	Safe	Effective	No mention
Pierot, 2020[751]	J Neuroradiol	FD	28	2	France	RO	2019-2020	1.00	None	None	Safe	Effective	No mention
Pierot, 2020[752]	J Neurointervent Surg	Coil	1135	16	France	PO	2013-2015		Yes	None	No mention	Doubt	No mention
Petrov, 2020[753]	Intervent Neuroradiol	FD	29	1	Mongolia	PO		6.74	Yes	Yes	Doubt	Doubt	No mention

© 2023 Volovici V et al. *JAMA Network Open.*

Peng, 2019[754]	Clin Neurorad	Endo	271	2	China	RO	2011-2016	60.00	Yes	None	No mention	Effective	Doubt
Pang, 2020[755]	Int J Clin Exp Med	Endo	128	1	China	RO	2013-2018		Yes	None	No mention	Doubt	No mention
Pasqualin, 2016[756]	Acta Neurochir Suppl	Clip	66	2	Italy	RO	1990-2014	3.00	NR	None	No mention	No mention	No mention
Van Lanen, 2020[757]	World Neurosurg	Clip	190	1	Netherlands	RO	2004-2015	12.00	NR	None	Doubt	Doubt	No mention
Tamrakar, 2011[758]	J Nepal Med Assoc	Coil	250	1	China	RO	2007-2009		NR	NS	No mention	Effective	No mention
Otani, 2020[759]	World Neurosurg	Clip	38	1	Japan		2009-2018	49.20	NR	None	No mention	No mention	Durable
Orru, 2020[760]	J Neurointerv Surg	FD	25	2	Canada	PO	2019-2019	3.00	None	Yes	Safe	Effective	No mention
Oğuz, 2019[761]	Turk J Med Sci	PED	49	2	Turkije	RO	2015-2017	12.00	NR	None	Safe	Effective	No mention
Nussbaum, 2020[762]	Oper Neurosurg	Clip	300	1	USA	RO	1997-2008		NR	NS	No mention	Doubt	No mention
Nurminen, 2020[763]	Clin Neurol Neurosurg	FD	62	1	Finland	RO	2014-2019		NR	NS	No mention	Doubt	No mention
Nanda, 2014[764]	World Neurosurg	Clip	62	1	USA	RO	1992-2009	21.78	NR	None	Doubt	Doubt	No mention
Nanda, 2014[765]	J La State Med Soc	Clip	125	1	USA	RO	1992-2012	34.00	NR	NS	Doubt	Doubt	No mention
Munich, 2020[766]	World Neurosurg	Clip	375	1	USA	RO	2010-2018	26.50	NR	Yes	Safe	Effective	No mention
Aydin, 2021[767]	J Neurosurg	SAC	102	4	Mutli-country	RO		20.00	NR	None	Safe	Effective	Durable
Moubark, 2020[768]	Egypt J Radiol Nucl Med	FD	47	2	Egypt	RO	2014-2016	20.00	None	None	Doubt	Effective	No mention
Michelozzi, 2019[769]	J Neurosurg	FD	29	1	France	RO	2010-2017		NR	NS	Safe	Effective	No mention
McAvoy, 2020[770]	Intervent Neuroradiol	Coil	105	1	USA	RO	2015-2018		None	Yes	Safe	Effective	No mention

© 2023 Volovici V et al. *JAMA Network Open.*

Maus, 2020[771]	Clin Neurorad	FD	42	2	Germany	PO	2019-2020	3.81	NR	None	Safe	Effective	No mention
Mascitelli, 2020[772]	J Neurointerv Surg	SAC	29	5	USA	RO	2016-2020		None	Yes	Doubt	Effective	No mention
Lv, 2011[773]	Neuroradiol J	Endo	63	1	China	RO	1998-2005	17.00	NR	NS	No mention	Doubt	Doubt
Lv, 2012[774]	Eur J Radiol	SAC	46	1	China	RO	2009-2010		NR	None	Doubt	Effective	No mention
Lv, 2020[775]	Neuroradiol J	PED	80	1	China	RO	2015-2019		Yes	None	Safe	Doubt	No mention
Lv, 2019[776]	J Neurorestoratology	SAC	104	1	China	RO	2017-2019		Yes	None	Safe	Effective	No mention
Kim, 2019[777]	J. Cerebrovasc. endovasc. neurosurg.	Coil	35	1	South Korea	RO	2006-2017	17.20	NR	NS	No mention	Effective	No mention
Ko, 2015[778]	J Korean Neurosurg Soc	Coil	30	1	South Korea	RO	2003-2013	32.70	Yes	NS	No mention	Effective	No mention
Baek, 2020[779]	J Clin Neurosci	Coil	38	1	South Korea	RO	2005-2016		None	None	No mention	No mention	No mention
Luzzi, 2020[780]	World Neurosurg	Clip	82	2	Italy	RO	2000-2019		NR	None	Doubt	No mention	Durable
Luo, 2020[781]	Ther Adv Neurol Disord	PED	1171	14	China	RO	2012-2019		Yes	None	Safe	Effective	No mention
Lukic, 2019[782]	Serb J Exp Clin Res	Coil	681	1	Serbia	PO	2010-2016	6.00	NR	NS	Safe	Effective	No mention
Luecking, 2020[783]	Clin Neuroradiol	SAC	81	1	Germany	RO	2014	7.20	Yes	None	Safe	Effective	No mention
Luecking, 2020[784]	Intervent Neuroradiol	FD	78	1	Germany			36.90	None	None	Safe	Effective	No mention
Lopes, 2018[785]	Neurosurgery	PED	91	17	Mutli-country	RO	2008-2013	21.10	Yes	Yes	No mention	No mention	No mention
Leonardi, 2008[786]	Intervent Neuroradiol	Coil	39	1	Italy	RO	2005-2007		NR	NS	No mention	Doubt	No mention

© 2023 Volovici V et al. *JAMA Network Open.*

Le Feuvre, 2008[787]	S Afr Med J	Endo	75	1	South Africa	RO	2002-2003		NR	NS	No mention	No mention	Durable
Labeyrie, 2017[788]	World Neurosurg	WEB	36	3	Mutli-country	RO	2012-2015		NR	Yes	No mention	Doubt	No mention
Kühn, 2016[789]	Intervent Neuroradiol	FD	56	3	USA	RO	2011-2017		None	Yes	Safe	Effective	No mention
Kim, 2019[790]	J Korean Neurosurg Soc	SAC	31	1	South Korea	RO	2018-2018	6.00	Yes	None	Safe	Effective	No mention
Lafuente, 2003[791]	J Neurol Neurosurg Psychiatry	Clip	245	1	UK	RO	1990-1999	12.00	NR	None	No mention	Doubt	No mention
Kaya, 2020[792]	Clin Imaging	WEB	42	1	Turkey	RO	2014-2018	7.00	NR	None	Safe	Effective	No mention
Kannan, 2019[793]	Asian J Neurosurg	Clip	347	1	Japan	RO	2016-2018		None	None	Safe	Doubt	No mention
İnci, 2020[794]	World Neurosurg	Clip	70	1	Turkey	RO	1997-2007	105.20	NR	None	Doubt	Effective	Durable
Igüs, 2020[795]	Med J Bakirkoy	FD	41	1	Turkey	RO	2010-2014		None	None	Doubt	Effective	No mention
Guglielmi, 2008[796]	Intervent Neuroradiol	Coil	113	1	USA				NR	NS	Doubt	Effective	No mention
Griessenauer, 2020[797]	Am J Neuroradiol	FD	84	8	Mutli-country	RO	2012-2019	27.00	Yes	Yes	Safe	Effective	No mention
Gory, 2019[798]	Stroke	FD	398	17	France	PO	2012-2014	12.00	Yes	Yes	Doubt	Effective	No mention
Goel, 2014[799]	Asian J Neurosurg	Coil	304	1	India	RO	2006-2011		NR	NS	No mention	Effective	No mention
Gariel, 2020[800]	J Neuroradiol	FD	101	22	France	PO	2012-2014	12.00	Yes	Yes	Safe	Effective	No mention
Fuse, 2000[801]	Intervent Neuroradiol	Coil	203	1	France	RO	1993-1998		NR	NS	Doubt	Doubt	No mention
Fujimoto, 2020[802]	AJNR Am J Neuroradiol	WEB	47	1	Japan	RO	2009-2016	15.40	NR	NS	Safe	Effective	Doubt

© 2023 Volovici V et al. *JAMA Network Open.*

Foreman, 2020[803]	World Neurosurg	FD	50	6	USA	RO	2009-2018	24.30	NR	Yes	Safe	Effective	No mention
Enomoto, 2020[804]	World Neurosurg	Endo	31	1	Japan	RO	2004-2018	12.00	NR	None	Doubt	Doubt	No mention
Dinc, 2021[805]	Neuroradiology	FD	136	10	Turkey	PO	2013-2016		None	Yes	Safe	Effective	Doubt
Decharin, 2020[806]	Asian J Neurosurg	Coil	102	1	Thailand	RO	2002-2018		None	None	No mention	Doubt	No mention
Chiu, 2018[807]	J Neurosurg	Clip	25	13	Mutli-country	PO	2013-2015		NR	Yes	No mention	Effective	No mention
De Beule, 2020[808]	Intervent Neuroradiol	FD	108	1	Belgium	RO	2014-2019		None	None	Safe	Effective	No mention
Chitale, 2012[809]	Neurosurgery	PED	36	1	USA	RO	2010-2011	2.99	NR	NS	No mention	Effective	No mention
Chen, 2020[810]	Front Neurol	SAC	25	1	China	RO	2018-2019		Yes	None	Doubt	Doubt	No mention
Chalouhi, 2014[811]	J Clin Neurosci	Clip	138	1	USA	RO	2004-2011	42.50	NR	None	Safe	Effective	Durable
Ban, 2017[812]	Oper Neurosurg	Bypass	62	1	South Korea	RO	2000-2014	46.50	NR	None	No mention	Doubt	No mention
Beuing, 2020[813]	Sci Rep	SAC	32	1	Germany	RO	2015-2018		None	None	Safe	Effective	No mention
Birknes, 2006[814]	Neurosurgery	Coil	123	1	USA	RO	2000-2003	6.00	NR	NS	No mention	No mention	No mention
Borota, 2021[815]	Interv	Endo	26	1	Sweden	RO	2012-2019		None	None	No mention	Doubt	No mention
Bracard, 2002[816]	AJNR Am J Neuroradiol	Coil	80	1	France	RO	1992-1998	12.00	NR	NS	No mention	Effective	No mention
Brilstra, 2002[817]	J Neurosurg	Bypass	77	1	Netherlands	RO	1990-2000	2.50	NR	NS	Safe	Effective	Durable
Brzegowy, 2019[818]	Wideochir Inne Tech Maloinwazyjne	Endo	111	1	Poland	RO	2012-2016	1.00	NR	None	Safe	Effective	No mention

© 2023 Volovici V et al. *JAMA Network Open.*

Chen, 2020[819]	Chin Neurosurg J	Endo	74	1	China	RO	2011-2020	3.00	Yes	None	No mention	Doubt	No mention
Cherian, 2020[820]	Neurosurgery	PED	39	16	USA	RO	2012-2018		NR	None	Safe	Effective	No mention
Trivelato, 2019[821]	Stroke	PED	146	7	Brazil	PO	2016-2018		NR	NS	Safe	Effective	No mention
Kallmes,2015[822]	Am J Neuroradiol	PED	793	17	Multi-country	RO	2008-2013	19.3	NR	NS	Doubt	No mention	No mention
Kobayashi, 2000[823]	Intervent Neuroradiol	Coil	35	1	Japan	RO			NR	NS	No mention	No mention	No mention
Fargen, 2013[824]	Neurosurgery	SAC	45	7	USA	RO		7.8	None	Yes	**Safe**	**Safe**	No mention
Garbossa, 2012[825]	Geriatr Gerontol Int	Coil	378	1	Italy	RO	1994-2009	57.6	None	None	Doubt	Doubt	No mention
Jung, 2019[826]	World Neurosurg	Coil	145	1	South Korea	RO	2017-2018		Yes	NS	**Safe**	No mention	No mention
Limbucci, 2020[827]	Neurosurgery	SAC	52	1	Italy	RO	2010-2013	26	None	NS	Doubt	No mention	No mention
Ibrahim Ali, 2016[828]	Front Neurol	Coil	30	2	Egypt	PO	2013-2014	1	NR	None	**Safe**	No mention	No mention
Gu, 2012[829]	Acad Radiol	Coil	96	1	China	RO	2003-2010		NR	NS	No mention	No mention	No mention
Hauck, 2008[830]	J Neurosurg	Clip	62	1	USA	RO	1998-2006	12	NR	None	No mention	No mention	No mention
Brinjikji, 2016[831]	Journal of Neurosurgery	PED	711	17	USA	RO	2008-2013	21	Yes	NS	Doubt	No mention	No mention
Ohnishi, 1998[832]	Interv Neuroradiol	Clip	92	1	Japan	RO	1980-1998		NR	NS	Doubt	Doubt	No mention
Oishi, 2020[833]	J Clin Neurosci	SAC	46	1	Japan	RO	2015-2020	27.4	Yes	None			
Burkhardt, 2020[834]	World Neurosurg	Clip	85	1	USA	RO	1997-2016		None	None	**Safe**	No mention	No mention
Yuan, 2020[835]	Clin Neurol Neurosurg	Endo	75	1	China	RO	2014-2018	16.9	None	None	No mention	**Effective**	No mention

© 2023 Volovici V et al. *JAMA Network Open.*

Klompenhouwer, 2011[836]	Am J Neuroradiol	Clip vs coil	**173 vs 230**	1	The Netherlands	RO	2000-2008	33.9	NR	None	Safe -No mention	Effective – No mention	No mention - No mention
Iwamuro, 2007[837]	Intervent Neuroradiol	Clip vs coil	78 vs 54	1	Japan	RO	2000 **-2005**	3	NR	NS	**No mention - Safe**	**No mention - Effective**	No mention - No mention
Jiang, 2014[838]	Cardiovasc Intervent Radiol	Coil vs coil	**56 vs 68**	1	China	RO	2009 **-2012**		NR	None	**Safe - No mention**	**Effective – No mention**	No mention - No mention
Jang, 2011[839]	The Korean Neurosurgical Society	Clip vs coil	**56 vs 25**	1	South Korea	RO	1997-**2009**	12	NR	NS	No mention - No mention	Effective – Effective	No mention - No mention
Jamróz, 2019[840]	Pol J Radiol	Clip vs Endo	**32 vs 33**	1	Poland	RO	2008-2014		NR	None	**Safe - Safe**	Effective – Effective	No mention - No mention
Jahshan, 2013[841]	Clin Neurosurgery	SAC vs coil	225 **vs 264**	1	USA	RO	2005-2009	18.2	NR	NS	No mention - No mention	Effective – No mention	No mention - No mention
Jin, 2009[842]	J Korean Neurosurg	Clip vs coil	28 vs 49	1	South Korea	RO	1999-2007	6	NR	NS	**Safe - Safe**	No mention - No mention	No mention - No mention
Johnston, 2000[843]	Ann Neurol	Clip vs coil	68 **vs** 62	1	USA	RO		46.8	Yes	NS	No mention - Safe	No mention - No mention	No mention - No mention
Ishii, 2008[844]	Neurosurgery	BAC + SAC **vs** BAC + SAC **vs** Coil	255 **vs** 79 vs 15	1	USA	RO	2002**-2006**		NR	NS	No mention - No mention - No mention	No mention - Effective – Effective	No mention - No mention -No mention
Kabbasch, 2019[845]	J Neurointervent Surg	WEB vs SAC	66 vs 66	3	Germany	RO	**2011 - 2018**	6	NR	NS	**Safe - Safe**	Doubt - Doubt	No mention - No mention
Kadkhodayan, 2012[846]	J Neurointervent Surg	SAC vs SAC	50 vs 56	1	USA	PO	2003-**2009**		NR	NS	**Safe - Safe**	Effective - Effective	No mention - No mention
Kaesmacher, 2016[847]	J Neurointervent Surg	Coil vs coil		1	Germany	RO	2010-2015		NR	NS	No mention - Safe	No mention – Effective	No mention - No mention
Kato, 2001[848]	Acta Neurochi	Clip vs coil	84 vs **14**	1	Japan	RO	1998-1999		NR	NS	No mention - No mention	Effective – Effective	No mention - No mention
Katsaridis, 2006[849]	Am J Neuroradiol	Coil vs coil	187 vs **120**	1	Greece	RO	2001-2005		NR	NS	Doubt - Doubt	No mention - No mention	No mention - No mention
Kawabata, 2011[850]	J Neurointervent Surg	Clip vs coil	77 vs 25	1	Japan	RO	1999-2010		NR	NS	No mention - Doubt	No mention - No mention	No mention - No mention

© 2023 Volovici V et al. *JAMA Network Open.*

Kawabe, 2006[851]	Clin Neurol Neurosurg	Clip vs coil	51 vs 38	1	Japan	RO	1998-2003	37.3	NR	NS	No mention - No mention	No mention - No mention	No mention - Doubt
Khandelwal, 2005[852]	Minimally Invasive Neurosurg	Clip vs coil	126 vs 324	1	Japan	RO	1997-2003		NR	NS	No mention - Doubt	No mention - No mention	No mention - No mention
Kim, 2008[853]	Am J Neuroradiol	Clip vs coil	35 vs 37	1	South Korea	RO	1999-2006	31 vs 27	NR	NS			
Kim, 2020[854]	J. cerebrovasc. endovasc. neurosurg.	Clip vs coil	187 vs **434**	1	South Korea	RO	2000-2015	6	NR	NS	Doubt - Doubt	No mention - No mention	No mention - No mention
Izar, 2011[855]	PLoS ONE	SAC vs SAC	42 vs **42**	1	USA	RO	2001 - 2010		NR	NS	**Safe - Safe**	Effective – Effective	No mention - No mention
Kim, 2018[856]	J. cerebrovasc. Endovasc neurosurg	BAC + SAC vs coil	103 vs **45**	1	South Korea	RO	2012 - 2013	39.5	NR	None	No mention - No mention	Doubt - Doubt	No mention - No mention
Kim, 2017[857]	J Korean Neurosurg Soc	SAC vs coil	37 vs **61**	1	South Korea	RO	2005 - 2016	37.1 vs 31.5	NR	NS	Safe – No mention	Effective – No mention	No mention - No mention
Kim, 2010[858]	Acta Neurochir	SAC vs SAC	37 vs **37**	1	South Korea	RO			Yes	NS	No mention - No mention	Effective - Effective	No mention - No mention
Kim, 2014[859]	Neurosurgery	SAC vs PED vs clip vs bypass	41 vs 24 vs 24 vs **20**	1	South Korea	RO	2007 - 2012		NR	None	No mention - No mention No mention - No mention	No mention - No mention No mention - No mention	No mention - No mention No mention - No mention
Chalouhi, 2013[860]	Clinical Neurology and Neurosurgery	Clip vs coil vs SAC	5 vs **32 vs 3**	1	USA	RO	2004 - 2009	15 vs 8	NR	NS	No mention - Doubt	No mention - Doubt	No mention - No mention
Chalouhi, 2013[861]	Stroke	BAC + SAC vs PED	40 vs **120**	1	USA	RO	2004 - 2012		NR	NS	**Safe - Safe**	No mention - No mention	No mention - No mention
Jee, 2019[862]	World Neurosurgery	Clip vs coil	178 vs **137**	1	South Korea	RO	2001 - 2015	58.8 vs 49.2	NR	None	**Safe - Safe**	No mention - No mention	Durable – No mention
Kato, 2020[863]	World Neurosurgery	SAC vs SAC vs SAC	103 vs 105 vs **127**	1	Japan	RO	2010 - 2019	77.9 vs 49.2 vs 14.9	NR	None	No mention - No mention No mention	No mention - No mention No mention	No mention - No mention No mention
Kiselev, 2018[864]	Clin Neurol Neurosurg	FD vs occlusion	40 vs **40**	2	Russia	PO	2015 - 2017		NR	NS	No mention - Doubt	No mention - No mention	No mention - No mention

© 2023 Volovici V et al. *JAMA Network Open.*

Brinjikji, 2011[865]	Stroke	Clip vs coil	29886 vs **34054**	1	USA	RO	2001 - 2008		NR	NS	No mention - Safe	No mention - No mention	No mention - No mention
Brinjikji, 2015[866]	American Journal of Neuroradiology	Coil vs coil	144 vs **144**	24	Multi-country	RCT		17	Yes	NS	No mention - No mention	No mention - No mention	No mention - No mention
Nagashima, 2004[867]	Journal of Clinical Neuroscienc	Clip vs coil	76 vs **41**		Japan	RO	1998 - 2001		NR	NS	No mention - No mention	No mention - Effective	No mention - No mention
Choi, 2020[868]	Acta Neurochirurgica	Coil vs FD	47 vs **39**	1	South Korea	RO	2009 - 2018	33.3	Yes	None	Doubt - Doubt	Doubt - Doubt	No mention - No mention
Brinjikji, 2015[869]	American Journal of Neuroradiology	Coil vs coil	232 vs **237**	24	Multi-country	RCT		12	Yes	NS	Doubt – No mention	No mention - No mention	No mention - No mention
Chun, 2013[870]	Clin Neurol Neurosurg	Coil vs coil vs coil	21 vs **7 vs 28**	1	South Korea	RO	2003 – 2010		NR	NS	No mention - No mention	No mention - No mention	Doubt - No mention
Aoki, 2016[871]	Surg Neurol Int	Clip vs coil	28 vs **23**	1	Japan	RO	1990 - 2013	121.6 vs 61.4	Yes	None	Safe – No mention	No mention - No mention	No mention - No mention
Penning, 2020[872]	World Neurosurgery	WEB vs coil	52 vs **236**	3	Germany	RO	2010 – 2019	24	NR	NS			
Natarajan, 2008[873]	Am J Neuroradiol	Clip vs coil	107 vs **85**	1	USA	RCT	2005 – 2006		NR	NS	No mention - No mention	No mention - No mention	No mention - No mention
Navrátil, 2020[874]	Brain Sci	Clip vs coil	127 vs **51**	1	Czech Republic	RCT	2008 – 2017	3	NR	NS	No mention - No mention	No mention - No mention	No mention - No mention
Nishido, 2014[875]	Am J Neuroradiol	SAC vs coil	323 vs **1492**	1	France	RCT	2003 – 2012	24	NR	NS	Not safe-No mention	No mention - No mention	No mention - No mention
Ocal, 2019[876]	Am J Neuroradiol	FD vs FD + stent	35 vs **33**	1	Turkey	RO	2013 - 2017	12	NR	NS			
Oh, 2015[877]	Clin Neurol Neurosurg	Clip vs SAC	31 vs **154**	1	South Korea	RO	2008 - 2012	2	NR	NS	**Safe - Safe**	Effective - Effective	No mention - No mention
Orlicky, 2015[878]	Br J Neurosurg	Clip vs coil	37 vs **21**	1	Czech Republic	RO	1998 - 2010	1	NR	NS	No mention - No mention	No mention - No mention	No mention - No mention
Pan, 2013[879]	Neurosurgery	SAC vs BAC	45 vs **28**	2	Multi-country	RO	2008 - 2012		NR	NS	No mention - No mention	No mention - Doubt	No mention - No mention

© 2023 Volovici V et al. *JAMA Network Open.*

Park, 2012[880]	Neurosurgery	Clip vs coil	286 vs **185**	1	South Korea	RO	2008 - 2011	12.53	None	None	**Safe - Doubt**	No mention - No mention	No mention - No mention
Peschillo, 2017[881]	Oper Neurosurg	FD vs FD	26 vs **18**	2	Multi-country	RO	2010 - 2015	12 vs 6.12	None	None	No mention - No mention	No mention - No mention	No mention - No mention
Pierot, 2009[882]	Radiology	Coil vs endo	325 vs **222**	27	France	RO	2005 - 2006		None	None			
Pierot, 2011[883]	Radiology	Coil vs endo	608 vs **160**	20	France	RO	2006 - 2008		NR	Yes	**Safe - Safe**	Effective - Effective	No mention - No mention
Popiela, 2017[884]	Neurol Neurochir	Coil vs coil	32 vs **44**	2	Poland	RO	2012 - 2015		Yes	None	No mention - No mention	No mention - No mention	No mention - No mention
Proust, 2003[885]	J Neurosurg	Clip vs coil	186 vs **37**	1	France	RO	1990 - 2000		None	None	Doubt - Doubt	No mention - No mention	No mention - No mention
Raymond, 2017[886]	AJNR Am J Neuroradiol	Coil vs coil	222 vs **225**	25	Multi-country	RCT	2007 - 2014	18	Yes	NS	**Safe - Safe**	No mention - No mention	No mention - No mention
Ren, 2018[887]	World Neurosurg	Clip vs SAC	33 vs **50**	1	China	RO	2010 – 2016	12	NR	None	Not desribed- **Safe**	No mention - Effective	No mention - No mention
Richling, 2000[888]	Oper Tech Neurosurg	Clip vs coil	379 vs **337**	1	Austria	RO	1998 - 1999		NR	NS	No mention - No mention	No mention - No mention	No mention - No mention
Lanzino, 2006[889]	J Neurosurg	Clip vs coil	41 vs **47**	1	USA	RO	2002 – 2008		NR	NS	Doubt - Doubt	Doubt - Doubt	No mention - No mention
Linfante, 2009[890]	Am J Neuroradiol	Coil vs coil	63 vs **65**	1	USA	RO	2005 - 2008	12	NR	NS	Safe -Safe	No mention - No mention	No mention - No mention
Koh, 2013[891]	Singapore Med J	Clip vs coil	33 vs **23**	1	Singapore	RO	2005 – 2009	6	NR	NS	No mention - Doubt	Effective - Doubt	**Durable - Durable**
Kunz, 2013[892]	J Neurol	Clip vs coil	363 vs **200**	1	Germany	RO	2000 – 2010	12.6 vs 14.1	NR	NS	Doubt - Doubt	No mention - No mention	No mention - No mention
Lad, 2013[893]	Neurosurgery	Clip vs coil	1878 vs **2626**		UK	RO	2000 – 2009	28.83 vs 21.97	NR	NS	No mention - No mention	No mention - No mention	No mention - No mention
Lawson, 2013[894]	World Neurosurg	Clip vs coil	6611 vs **7439**		USA	RO	2002 - 2008		NR	Yes	Safe -Safe	No mention - No mention	No mention - No mention
Lee, 2014[895]	Acta Neurochir	Coil vs BAC + SAC	401 vs **221**		South Korea	RO	2008 - 2011		NR	Yes	No mention - No mention	Effective - Effective	Durable - Durable

© 2023 Volovici V et al. *JAMA Network Open.*

Liu, 2014[896]	J Clin Neurosci	SAC vs coil	56 vs **235**	1	China	RO	2008 – 2012	13.8	NR	NS	Doubt – No mention	No mention - No mention	Doubt – No mention
Lehto, 2015[897]	World Neurosurg	Clip vs coil	7 vs **22**	1	USA	RO	2008 - 2015	58.6	NR	None	Doubt - Doubt	Doubt - Doubt	No mention - No mention
Lan, 2017[898]	World Neurosurg	Clip vs clip	195 vs **123**	1	China	RO	2004 – 2015	6	NR	NS	Safe -Safe	Effective - Effective	No mention - No mention
Li, 2017[899]	World Neurosurg	Clip vs BAC + SAC	85 vs **77**	1	China	RO	2002-2010	12	NR	Yes	No mention - No mention	No mention - No mention	No mention - No mention
Koyanagi, 2019[900]	J Neurosurg	Clip vs coil	382 vs **184**	3	Japan	RO	2009 – 2016		NR	None	No mention - No mention	No mention - No mention	No mention - No mention
Kumar, 2019[901]	World Neurosurg	Clip vs BAC + SAC	4 vs **36**	1	Japan	RO	2014 - 2018	10.5 vs 13.3	NR	NS	Safe -Safe	No mention - No mention	No mention - No mention
Kwinta, 2019[902]	World Neurosurg	Clip vs SAC	72 vs **67**	1	Poland	RO	2007 - 2017	12	NR	None	Safe -Safe	**Effective - Effective**	No mention - No mention
Li, 2019[903]	Front Neurol	SAC vs SAC	42 vs **45**	1	China	RO	2016 - 2017	9.1 vs 11.82	Yes	None	No mention - No mention	No mention - No mention	No mention - No mention
Chalouhi, 2012[904]	J Am Heart Assoc	Clip vs BAC + SAC	60 vs **31**	1	USA	RO	2004 – 2011		NR	None	Doubt - Safe	**Doubt - Effective**	No mention - No mention
Chalouhi, 2013[905]	AJNR Am J Neuroradiol.	SAC vs BAC	69 vs **32**	1	USA	RO	2009 – 2010		NR	None	Safe -Safe	Effective - Effective	No mention - No mention
Ghorbani, 2020[906]	Interdiscip Neurosurg	Clip vs coil	42 vs **38**	1	Iran	RO	2011 – 2016		NR	None	Safe -Safe	Effective - Effective	No mention - No mention
Krylov, 2012[907]	Neurosci Behav Physiol	Clip vs coil	49 vs **18**	1	Russia	RO	1992 – 2010		NR	NS	Safe -Doubt	No mention - No mention	No mention - No mention
La Pira, 2016[908]	Acta Neurochir	Clip vs endo	7 vs **22**	1	USA	RO	2008 - 2015	58.6 vs 56.4	NR	Yes	Safe -Doubt	Effective - Doubt	No mention - No mention
Lim, 2020[909]	J Neurointervent Surg	SAC vs SAC	168 vs **245**	1	South Korea	PO	2012 - 2017	15.5 vs 24.9	NR	None	No mention - No mention	No mention - No mention	No mention - No mention
Liu, 2017[910]	Med Sci Monit	SAC vs coil	113 vs **166**	1	China	RO	2004 - 2015	28	NR	NS	Safe -Safe	No mention - No mention	Durable -Doubt
Lodi, 2011[911]	J Vasc interv radiol neurol	SAC vs SAC	37 vs **50**	1	UK	RO	2003 - 2010		NR	NS	Safe -Doubt	No mention - No mention	No mention - No mention

© 2023 Volovici V et al. *JAMA Network Open.*

Lot, 1998[912]	Neurol Med Chir	Clip vs coil	102 vs **293**	1	Japan	PO	1992 - 1996		NR	NS	Safe -Safe	Effective - Effective	No mention - No mention
Liu, 2020[913]	Medecine	Clip vs coil	112 vs **40**	1	China	RO	2014 - 2018		None	None	No mention - No mention	No mention - No mention	No mention - No mention
Aboukais, 2014[914]	Clin Neurol Neurosurg	Clip vs SAC	23 **vs 29**	1	France	RO	2000 - 2011	55.2	NR	NS	No mention - No mention	No mention - No mention	No mention - No mention
Aboukais, 2014[915]	Neurochirurgie	Clip vs coil	110 **vs 99**	1	France	RO	2009 - 2009		NR	None	No mention - No mention	No mention - No mention	No mention - No mention
Aboukais, 2015[916]	Neurochirurgie	Clip vs coil	19 **vs 9**	1	France	RO	2002 - 2012	40.8	NR	None	No mention - No mention	No mention - No mention	No mention - No mention
Adeeb, 2017[917]	World Neurosurg	PED vs SAC	106 **vs 62**	2	USA	RO	2007 - 2016		NR	None	No mention - No mention	Effective - Effective	No mention - No mention
Ahmed, 2013[918]	Egypt J Radiol	Clip vs coil	15 **vs 15**	1	Egypt	PO	2010 - 2011		NR	None	Doubt - Safe	No mention - No mention	No mention - No mention
Bendok, 2020[919]	Neurosurgery	Coil vs coil	297 **vs 303**	1	USA	RCT			Yes	NS	No mention - No mention	No mention - No mention	No mention - No mention
Bendszus, 2007[920]	Stroke	Coil vs coil	54 **vs 55**	1	Germany	PO	2002 - 2004		NR	NS	Safe - Safe	No mention - No mention	No mention - No mention
Alawi, 2014[921]	J Neurosurg Pediatr	Clip vs coil	20 **vs 920**	1	USA	RO	1998 - 2009		NR	None	No mention - No mention	Doubt - Doubt	No mention - No mention
Allen, 2018[922]	Neurocrit Care	Clip vs coil	66 **vs 194**	2	USA	RO	2010 - 2015		NR	None	No mention - No mention	No mention - No mention	No mention - No mention
AlMatter, 2018[923]	J Neuroradiol	Clip vs endo	135 vs **452**	2	Germany	RO	2007 - 2016	111 vs 32	None	None	Safe - Safe	No mention - No mention	No mention - No mention
Barker, 2004[924]	Neurosurgery	Clip vs coil	3498 **vs 421**	1	USA	RO	1996 - 2000		None	NS	Safe - Safe	Effective - Effective	No mention - No mention
Bekelis, 2015[925]	J Am Heart Assoc	Clip vs coil	1453 **vs 3190**	3	Multi-country	RO	2009 - 2013		Yes	NS	No mention - No mention	No mention - No mention	No mention - No mention
Bhatia, 2011[926]	Acta Neurochir (Wien)	Clip vs coil	157 **vs 68**	1	USA	RO	2002 - 2007	145 vs 6	NR	None	No mention - No mention	No mention - No mention	No mention - No mention
Bose, 2012[927]	J Clin Neurosci	Coil vs coil	136 **vs 277**	2	Australia	RO	1998 - 2009	20.9	NR	NS	No mention - No mention	Effective – No mention	No mention - No mention
Braun, 2005[928]	Neuroradiology	Clip vs coil	18 **vs 19**	1	Germany	RO	1999 - 2003	40 vs 16	NR	NS	No mention - No mention	No mention - Doubt	No mention - No mention

© 2023 Volovici V et al. *JAMA Network Open.*

Brilstra, 2004[929]	Cerebrovasc Dis	Clip vs coil	32 **vs 19**	3	The Netherlands	RO	1997 - 2000	32 vs 3	NR	NS	No mention - No mention	No mention - No mention	No mention - No mention
Brinjikji, 2011[930]	AJNR Am J Neuroradiol	Clip vs coil	29918 **vs 34125**	1	USA	RO	2001 - 2008		NR	NS	Doubt - No mention	No mention - No mention	No mention - No mention
Britz, 2004[931]	Stroke	Clip	3527	1	USA	RO	1987 **-2001**	120	NR	NS	Doubt	No mention	No mention
Cantore, 2008[932]	Neurosurgery	Clip vs clip + bypass	99 **vs 31**	1	Italy	RO	1990 - 2004	8.6	NR	NS	No mention - No mention	No mention - No mention	No mention - No mention
Catapano, 2021[933]	Acta Neurochirurgica	Clip vs coil	58 **vs 15**	1	USA	RO	2003 - 2007	10	NR	None	Safe - Safe	Effective- No mention	No mention - No mention
Bründl, 2016[934]	World Neurosurgery	Clip vs endo	10 **vs 10**	1	Germany	RO	2013 - 2015	10 vs 1.5	None	None	No mention - No mention	No mention - No mention	No mention - No mention
Birski, 2014[935]	Polish Journal of Neurology and Neurosurgery	Clip vs coil	114 **vs 48**	2	Poland	RO	2003 - 2008	94	None	None	Doubt -Doubt	No mention - No mention	No mention - No mention
Cai, 2016[936]	World Neurosurgery	SAC vs BAC	65 **vs 32**	1	China	RO	2011 - 2014	45 vs 43	Yes	None	Doubt - Doubt	No mention - No mention	No mention - No mention
Brunken, 2009[937]	Neuroradiologie	Clip vs coil	370 **vs 145**	2	Germany	RO	1990 - 2004	6	None	None	No mention - No mention	No mention - No mention	No mention - No mention
Chalouhi, 2014[938]	Stroke	PED vs SAC	40 **vs 160**	1	USA	RO	2011 - 2013	7 vs 17	None	None	**Safe - Safe**	Effective - Effective	No mention - No mention
Chung, 2014[939]	Acta Neurochir	SAC vs coil vs BAC	127 **vs 45 vs 35**	1	South Korea	RO	2008 - 2013		NR	None	**Safe - Safe**	Effective - Effective	No mention - No mention
Claiborne Johnston, 1999[940]	Neurology	Clip vs coil	2357 **vs 255**	60	USA	RO	1994 - 1997		NR	NS	No mention - Doubt	No mention - No mention	No mention - No mention
Claiborne Johnston, 2001[941]	Stroke	Clip vs coil	1699 **vs 370**	167	USA	RO	1990 - 1998		NR	NS	No mention - Doubt	No mention - No mention	No mention - No mention
Coley, 2012[942]	AJNR	Coil vs coil	249 **vs 251**	23	Multi-country	RCT	2005 - 2005		Yes	NS	Doubt - Doubt	No mention - No mention	No mention - No mention
Consoli, 2016[943]	J NeuroIntervent Surg	BAC vs SAC	151 **vs 117**	1	Italy	RO	2004 - 2012		NR	None	No mention - Doubt	No mention - Doubt	No mention - No mention
Crobeddu, 2014[944]	Int J Neurosci	Clip vs coil	5 **vs 36**	1	Italy	RO	1994 - 2011		None	None	No mention - No mention	No mention - Effective	No mention - No mention

© 2023 Volovici V et al. *JAMA Network Open.*

Crocker, 2008[945]	J Neurosurg	Clip vs coil	44 **vs 237**	1	UK	RO	2005 - 2007		NR	None	No mention - No mention	No mention - No mention	No mention - No mention
Dabus, 2018[946]	J Neurointervent Surg	BAC vs coil	85 **vs 110**	27	Multi-country	PO	2009 - 2016		Yes	Yes	No mention - No mention	Doubt – No mention	No mention - No mention
Dengler, 2021[947]	J Neurosurg	Clip vs coil	50 **vs 130**	32	Multi-country	RO	2006 - 2016	57.6	Yes	Yes	Doubt - Doubt	No mention - No mention	No mention - No mention
Derrey, 2015[948]	Neurochirurgie	Clip vs coil	29 **vs 42**	1	France	RO	2004 - 2008	6	NR	None	No mention - Doubt	No mention - No mention	No mention - No mention
Di, 2020[949]	J Clin Neurosci	SAC vs SAC	45 **vs 56**	1	China	RO	2013 - 2017	8.26 vs 11.9	NR	None	No mention - Doubt	No mention - Doubt	No mention - No mention
Diaz, 2014[950]	World Neurosurg	Clip vs coil	34 **vs 50**	1	USA	RO	2005 - 2010	4.9 vs 11.9	None	None	No mention - Doubt	No mention - Doubt	No mention - No mention
Dietrich, 2020[951]	Clin Neuroradiol	SAC vs SAC	47 **vs 48**	1	Germany	RO	2012 - 2018	25.2 vs 9.6	None	None	**Safe** - **Safe**	Effective - Effective	No mention - No mention
Dong, 2016[952]	Int J Surg	Clip vs coil vs clip + coil	33 vs 39 **vs 18**	1	China	RO	2005 - 2014		Yes	None	Doubt - Safe	No mention - No mention	No mention - No mention
Dumont, 2010[953]	Stroke	Clip vs coil	199 **vs 214**	52	Multi-country	RO	2005 - 2006		Yes	None	No mention - Doubt	No mention - No mention	No mention - No mention
Durst, 2014[954]	Clin Radiol	SAC vs SAC	53 **vs 77**	1	USA	RO	2002 - 2012	33	NR	NS	No mention - No mention	No mention - No mention	No mention - No mention
Enriquez-Marulanda, 2019[955]	Neuroradiol J	SAC vs PED	17 **vs 21**	1	USA	RO	2008 - 2017		None	Yes	Doubt - Doubt	Effective - Effective	No mention - No mention
Fan, 2016[956]	Clin Neurol Neurosurg	SAC vs coil	63 **vs 159**	1	China	RO	2008 - 2015	25.7 vs 35.6	NR	None	Doubt - Doubt	No mention - Doubt	No mention - No mention
Feng, 2020[957]	Cerebrovasc Dis	Coil vs FD	80 **vs 26**	3	China	RO	2013 - 2018		Yes	None	**Safe** - **Safe**	No mention - No mention	No mention - No mention
Frontera, 2014[958]	J Neurointervent Surg	Clip vs SAC vs coil	36 vs 47 **vs 33**	1	USA	RO	2003 - 2010		None	None	**Safe** - **Safe**	No mention - No mention	No mention - No mention
Gaba, 2006[959]	Stroke	Coil vs coil	50 **vs 57**		USA	RO	2003-2004		Yes	Yes	No mention - No mention	No mention - No mention	Doubt - No mention
Ge, 2016[960]	World Neurosurg	Coil vs coil	92 **vs 98**	1	China	RO	2014-2015	6.3	Yes	NS	Doubt - Doubt	Effective - Effective	No mention - No mention

© 2023 Volovici V et al. *JAMA Network Open.*

Gentric, 2015[961]	Neurosurgery	BAC + SAC vs SAC	51 **vs 46**	10	France	PO	2008-2010		None	Yes	Doubt – No mention	No mention - No mention	No mention - No mention
Gerlach, 2007[962]	J Neurol Neurosurg Psychiatry	Clip vs coil	81 **vs 37**	1	Germany	PO	1999-2005	6	NR	None	Doubt - Doubt	No mention - No mention	No mention - No mention
Sanai, 2006[963]	J Neurosurg	Coil vs BAC	13 **vs 16**	1	USA	RO	1977-2003	73.2 vs 58.8	NR	NS	No mention - No mention	No mention - No mention	No mention - No mention
Sauvigny, 2019[964]	Acta Neurochir	Clip vs coil vs WEB	56 **vs 107 vs 38**	1	Germany	RO	2015-2018	6	None	None	**Safe** - **Safe**	Effective - Effective	No mention - No mention
Schwyzer, 2015[965]	Acta Neurochir Suppl	Clip vs coil	63 **vs 41**	1	Switzerland	RO	2000-2006		NR	NS	No mention - No mention	No mention - No mention	No mention - No mention
Starke, 2015[966]	J Neurointervent Surg	SAC vs coil	120 **vs 60**		USA	RO	2006-2012	18.9 vs 27	NR	NS	**Safe** - **Safe**	Effective - Effective	No mention - No mention
Steklacova, 2016[967]	Acta Neurochirurgica	Clip vs coil	238 **vs 77**	3	Multi-country	RO	2000-2013		NR	None	No mention - No mention	Doubt - Doubt	No mention - No mention
Sweid, 2018[968]	J Clin Neuroscience	PED vs PED	363 **vs 74**	3	USA	RO	2010-2016	12.7	NR	None	**Safe** - **Safe**	No mention - No mention	No mention - No mention
Taheri, 2014[969]	Iran J Neurol	Clip vs coil	21 **vs 27**	8	Iran	PO	2011-2013	12	NR	None	No mention - No mention	No mention - No mention	No mention - No mention
Taschner, 2009[970]	Neuroradiology	Coil vs coil	38 **vs 38**	3	USA	RO	2005-2007	6	NR	None	No mention - No mention	No mention - No mention	No mention - No mention
Tsukahara, 2005[971]	Acta Neurochir Suppl	Clip vs coil	472 **vs 31**	6	Multi-country	RO	1999-2001	3.9	NR	NS	No mention - No mention	No mention - No mention	No mention - No mention
Vakharia, 2020[972]	J Neurointervent Surg	PED vs PED	49 **vs 33**	3	USA	RO	2014-2016	12	None	Yes	**Safe** - **Safe**	Effective - Effective	No mention - No mention
Van Rooij, 2008[973]	Am J Neuroradiol	Coil vs coil	120 **vs 101**		The Netherlands	RO	2006-2007	6	NR	NS	**Safe** - **Safe**	No mention - No mention	No mention - No mention
Vergouwen, 2011[974]	Stroke	Clip vs coil	548 **vs 383**		Canada	RO			Yes	NS	No mention - No mention	No mention - No mention	No mention - No mention
Ye, 2015[975]	Exp Ther Med	SAC vs SAC	43 **vs 47**	2	China	RO	2010-2012	11.4 vs 6.8	Yes	Yes	**Safe** - **Safe**	Effective - Effective	No mention - No mention
Zaidat, 2009[976]	J Neuroimaging	Clip vs coil	118 **vs 98**	4	USA	RO	1999-2005		None	None	No mention - No mention	No mention - No mention	No mention - No mention

© 2023 Volovici V et al. *JAMA Network Open.*

Zaidat, 2019[977]	Front Neurol	Coil vs coil	92 **vs 56**	12	USA	PO	2013-2014	5.9	Yes	None	No mention - No mention	No mention - No mention	No mention - No mention
Zanaty, 2016[978]	World Neurosurg	Clip vs coil	70 **vs 182**	5	USA	RO	2010-2015	1.6 vs 2.3	None	None			
Zhai, 2020[979]	Turk Neurosurg	Clip vs coil	18 **vs 24**	1	China	RO	2013-2017	31.3	Yes	NS	No mention - No mention	Effective - Effective	No mention - No mention
Zhao, 2017[980]	J Neurointervent Surg	SAC vs coil	23 **vs 108**	3	China	RO	2010-2012	12	Yes	None	**Safe - Safe**	Effective - Effective	No mention - No mention
Zhao, 2019[981]	World Neurosurg	Clip vs SAC	65 **vs 46**	1	China	RO	2008-2015	32.4	NR	NS	**Safe - Safe**	Effective - Effective	No mention - No mention
Zuo, 2018[982]	J Neurosurg	Coil vs coil	133 **vs 289**	1	China	RO	2012-2014	36	NR	NS	**Safe - Safe**	Effective - Effective	No mention - No mention
Yu, 2007[983]	Hong Kong Med	Clip vs coil	89 **vs 80**		China	RO	1995-2001	12	NR	None	No mention - No mention	No mention - No mention	No mention - No mention
Sharma, 2013[984]	Neurol India	Clip vs coil	74 **vs 66**		USA	RO	2007-2012	11 vs 7	None	None	No mention - No mention	No mention - No mention	No mention - No mention
Silva, 2019[985]	J Neurosurg	Clip vs coil	30 **vs 23**	2	USA	RO	2011-2017		None	None	**Safe - Safe**	Effective - Effective	No mention - No mention
Song, 2017[986]	Surg Neurol Intl	Clip vs coil	12 **vs 27**	1	South Korea	RO	2002-2013	12	None	None	No mention - No mention	No mention - No mention	No mention - No mention
Spetzler, 2020[987]	J Neurosurg	Clip vs coil	241 **vs 115**		USA	RCT	2003-2007	72	Yes	NS	No mention - No mention	No mention - No mention	No mention - No mention
Starke, 2014[988]	J Clin Neurosci	Clip vs coil	68 **vs 14**		USA	RO	2005-2011	21.4	NR	Yes	No mention - No mention	No mention - No mention	No mention - No mention
Vrsajkov, 2012[989]	Turk J Med Sci	Clip vs coil	52 **vs 30**	1	Serbia	RO	2008-2010		NR	NS			
Vanzin, 2005[990]	J Neuroradiol	Clip vs coil	9 **vs 90**	1	France	RO	1998-2003	58	None	None	No mention - No mention	No mention - No mention	No mention - No mention
Chen, 2019[991]	Exp Ther Med	SAC vs SAC	53 **vs 39**		China	RO	2014-2016	6	None	None	No mention - No mention	Effective - Effective	No mention - No mention
Taweesomboony, 2019[992]	World Neurosurg	Clip vs coil	104 **vs 85**	1	Thailand	RO	2002-2018	6	None	None	No mention - No mention	Effective - Effective	No mention - No mention
Xie, 2021[993]	Clin Neurol Neurosurg	Clip vs coil	46 **vs 32**	1	China	RO	2012-2018	12	None	None	**Safe - Safe**	Effective - Effective	No mention - No mention

© 2023 Volovici V et al. *JAMA Network Open.*

Zaeske, 2021[994]	World Neurosurg	PED vs FD	62 **vs 49**	4	Germany	RO	2011-2019	6	None	None	No mention - No mention	No mention - No mention	No mention - No mention
Wadd, 2015[995]	J Coll Phys Surg	Clip vs coil	70 **vs 70**	1	Pakistan	PO	2010-2013	12	None	None	**Safe** - **Safe**	Effective - Effective	No mention - No mention
Lu, 2019[996]	Exp Ther Med	SAC vs PED	23 **vs 20**	1	China	RO	2016-2018	7.8 vs 7.5	None	NS	**Safe** - **Safe**	Effective – No mention	No mention - No mention
Lubicz, 2008[997]	Neuroradiology	Coil vs BAC	174 **vs 80**		Belgium	CS	2004-2008	6	NR	None	No mention - No mention	No mention - No mention	No mention - No mention
Lusseveld, 2002[998]	J Neurol Neurosurg Psychiatry	Clip vs coil	44 **vs 44**	2	The Netherlands	RO	1983-1999	3.5 vs 4	Yes	NS	**Safe** - **Safe**	Effective - Effective	No mention - No mention
Lv, 2019[999]	J Invest Surg	SAC vs PED	81 **vs 63**		China	RO	2013-2017		NR	None	**Safe** - **Safe**	Effective - Effective	No mention - No mention
Makhambetov, 2019[1000]	Acta Neurochirgica	FD vs Endo +bypass	13 **vs 16**	1	Kazakhstan	RO	2008-2017	33	NR	None	**Safe** - **Safe**	Effective - Effective	No mention - No mention
Manabe, 2003[1001]	Interventional Neuroradiology	Clip vs coil	119 **vs 20**	1	Japan	RO	1997-2001		NR	NS	No mention - No mention	No mention - No mention	No mention - No mention
Manabe, 2004[1002]	Interventional Neuroradiology	Clip vs coil	75 **vs 15**	1	Japan	RO	1996-2002		NR	NS	No mention - No mention	No mention - No mention	No mention - No mention
Maria, 2015[1003]	J Neuroradiol	Coil vs FD	61 **vs 77**	2	France	RO	2006-2013	31.5 vs 13.5	NR	NS	Doubt- Doubt	Effective - Effective	No mention - No mention
Mascitelli, 2019[1004]	Neurosurgery	Clip vs coil	159 **vs 168**	1	USA	PO	2003-2007		Yes	NS	No mention - No mention	No mention - No mention	No mention - No mention
McDonald, 2013[1005]	Stroke	Clip vs coil	1388 **vs 3551**	120	USA	RO	2006-2011		NR	NS	Not desribed-Safe	No mention - No mention	No mention - No mention
McDougall, 2014[1006]	AJNR AM J Neuroradiol	Coil vs coil	311 **vs 315**	43	USA	PO	2007-2009	57.6	Yes	NS	No mention - No mention	No mention - No mention	No mention - No mention
McDougall, 2020[1007]	J Neurointerv Surg	Coil vs coil	311 **vs 315**	47	USA	PO	2007-2012		Yes	NS	No mention - No mention	No mention - No mention	No mention - No mention
McDougall, 2015[1008]	Interventional Neuroradiology	Coil vs coil	311 **vs 315**	43	USA	PO	2007-2009	0.6	Yes	NS	No mention - No mention	No mention - No mention	No mention - No mention
Milburn, 2014[1009]	J Neurointerv Surg	Coil vs coil	18 **vs 40**	1	USA	RO	2010-2012	8,6 vs 11.4	NR	NS	No mention - No mention	Effective - Effective	No mention - No mention

© 2023 Volovici V et al. *JAMA Network Open.*

Mohammad, 2020[1010]	Surg Neurol Int	Clip vs coil	147 **vs 53**	1	Japan	RO	2012-2016		NR	None	**Safe - Safe**	Effective - Effective	Durable – No mention
Mokin, 2020[1011]	J Neurointerv Surg	SAC vs SAC vs SAC	330 **vs 182 vs 158**	6	USA	RO	2009-2018	9.25	NR	Yes	No mention - No mention No mention	No mention - No mention No mention	No mention - No mention No mention
Moon, 2020[1012]	J Cerebrovasc Endovasc Neurosurg	Clip vs coil	33 **vs 37**	1	South Korea	RO	2012-2019	9.25	NR	Yes	No mention - No mention	No mention - No mention	No mention - No mention
Moret, 1997[1013]	Interv Neuroradiol	Coil vs BAC	50 **vs 205**		France	RO	1992-1996		NR	None	Doubt-Doubt	No mention - No mention	No mention - No mention
Mortimer, 2016[1014]	J Neurointerv Surg	Clip vs coil	66 **vs 69**		Australia	PO	2005-2010		NR	None	No mention - No mention	No mention - No mention	No mention - No mention
Murphy, 2005[1015]	Br J Neurosurg	Clip vs coil	73 **vs 74**	1	UK		1998-2003		NR	None	No mention - No mention	No mention - No mention	No mention - No mention
Cho, 2020[1016]	J Cerebrovasc Endovasc Neurosurg	Clip vs coil	11 **vs 44**	1	South Korea	RO	2010-2018	25.6 vs 25.6	NR	None	No mention - No mention	No mention - No mention	No mention - No mention
Miyachi, 1999[1017]	Interv Neuroradiol	Clip vs BAC	43 **vs 63**	1	Japan	RO	1990-1998		NR	None	No mention-Safe	No mention - No mention	No mention - No mention
Monteiro, 2020[1018]	Neurosurgery	SAC vs SAC	57 **vs 64**	1	USA	RO	2014-2019	18.7 **vs 13.6**	NR	Yes	**Safe - Safe**	Doubt-Doubt	Durable - Durable
Moon, 2015[1019]	Neurosurgery	Clip vs coil	91 **vs 39**		USA	RO	2003-2007		NR	None	**Safe - Safe**	No mention - No mention	No mention - No mention
McDonald, 2014[1020]	AJNR AM J Neuroradiol	Clip vs coil	1228 **vs 4001**	125	USA	RO	2006-2011		NR	None	No mention - No mention	No mention - No mention	No mention - No mention
McDougall, 2012[1021]	J Neurosurg	Clip vs coil	238 **vs 233**	1	USA	PO	2003-2007		NR	None	No mention-Safe	No mention - No mention	No mention - No mention
Mascitelli, 2015[1022]	J Neurointerv Surg	Coil vs coil	75 **vs 282**	1	USA	RO	2011-2013		NR	None	Safe – No mention	No mention - No mention	No mention - No mention
Mascitelli, 2013[1023]	J Neurointerv Surg	Coil vs coil	16 **vs 79**	1	USA	RO	2004-2011		NR	None	No mention - No mention	No mention - No mention	No mention - No mention
Molyneux, 2015[1024]	Lancet	Clip vs coil	1070 **vs 1073**	43	UK	PO	1994-2002		Yes	None	**Safe - Safe**	Effective-Effective	No mention-Durable

© 2023 Volovici V et al. *JAMA Network Open.*

Molyneux, 2002[1025]	Lancet	Clip vs coil	1070 **vs 1073**	43	UK	PO	1994-2002	12 vs 12	Yes	Yes	No mention-Safe	No mention - No mention	No mention-Durable
Choi, 2016[1026]	J Korean Neurosurg Soc	Clip vs coil	153 **vs 25**		South Korea	RO	2008-2012	11.7 vs 12.3	NR	NS	No mention - No mention	Doubt – No mention	No mention - No mention
Cho, 2015[1027]	Neurosurgery	SAC vs coil	157 **vs 157**	1	South Korea	RO	2009-2011	24.1 vs 22.9	NR	NS	No mention - No mention	Doubt – No mention	No mention - No mention
Goertz, 2019[1028]	World Neurosurg	Clip vs SAC	130 **vs 260**	1	Germany	RO	2010-2018		NR	None	**Safe - Safe**	No mention - No mention	No mention - No mention
Goertz, 2019[1029]	Am J Neuroradiol	WEB vs WEB	38 **vs 70**	3	Germany	RO	2011-2019		NR	NS	Doubt – No mention	No mention - No mention	No mention - No mention
Gordhan, 2011[1030]	Neurol Res	SAC vs coil	25 **vs 12**	1	USA	RO	2005-2009	16.9 vs 15.9	NR	NS	Safe – No mention	Effective – No mention	No mention - No mention
Griessenauer, 2020[1031]	J Neurointervent Surg	PED vs FD	285 **vs 84**	25	Multi-country	RO	2012-2019		None	NS	No mention - No mention	No mention - No mention	No mention - No mention
Guo, 2011[1032]	Eur J Radiol	Coil vs coil	73 **vs 64**	1	China	RO	2006-2009		NR	NS	No mention - No mention	No mention - No mention	No mention - No mention
Hadjivassiliou, 2001[1033]	Neurology	Clip vs coil	40 **vs 40**	1	UK	PO	1995-1996		NR	NS	No mention-Doubt	No mention - No mention	No mention - No mention
Hammer, 2017[1034]	World Neurosurg	Clip vs coil	390 **vs 271**	2	Germany	PO	1997-2014		NR	None	Doubt – No mention	Doubt – No mention	No mention - No mention
Hammer, 2017[1035]	PLoS One	Clip vs coil	390 **vs 252**	2	Germany	PO	1997-2014		None	NS	No mention - No mention	No mention - No mention	No mention - No mention
Helland, 2006[1036]	Neurosurgery	Clip vs coil	203 **vs 83**	1	Norway	RO	1987-2004	12	NR	NS	No mention-Doubt	No mention - No mention	No mention - No mention
Higashida, 2007[1037]	AJNR Am J Neuroradiol	Clip vs coil	1881 **vs 654**	429	USA	RO	1998-2000		NR	NS	No mention-Safe	No mention - No mention	No mention - No mention
Hoh, 2004[1038]	Neurosurgery	Clip vs coil	413 **vs 79**	1	USA	RO	1995-2003		NR	NS	Safe – No mention	No mention - No mention	No mention - No mention
Hohlrieder, 2002[1039]	Eur J Radiol	Clip vs coil	53 **vs 91**	1	Austria	RO	1997-1998		NR	NS	No mention - No mention	No mention - No mention	No mention - No mention
Horcajadas, 2018[1040]	Neurocirurgia	Clip vs endo	23 **vs 66**	1	Spain	RO	2010-2015	32 v**s** 38	NR	None	No mention - No mention	No mention - No mention	No mention - No mention

© 2023 Volovici V et al. *JAMA Network Open.*

Huang, 2019[1041]	World Neurosurg	Clip vs coil	45 **vs 37**	1	China	RO	2015-2017		NR	NS	**Safe** - **Safe**	Effective - Effective	No mention - No mention
Hui, 2011[1042]	J Neurointervent Surg	Clip vs coil	54 **vs 32**	1	USA	RO	1999-2009		NR	NS	No mention-Doubt	No mention - No mention	No mention - No mention
Hwang, 2014[1043]	J. cerebrovasc. endovasc. neurosurg.	Clip vs coil	40 **vs 30**	1	South Korea	RO	2006-2013		NR	NS	No mention - No mention	No mention - No mention	No mention - No mention
Iihara, 2003[1044]	J Neurosurg	Clip vs coil	34 **vs 76**	1	Japan	RO	1997-2002		NR	NS	Doubt -Doubt	No mention - No mention	No mention - No mention
Ikawa, 2018[1045]	Neurosurg Rev	Clip vs coil	2666 **vs 881**	163	Japan	RO	2000-2013		Yes	None	No mention - No mention	No mention - No mention	No mention - No mention
Ikawa, 2019[1046]	World Neurosurg	Clip vs coil	4996 **vs 3023**		Japan	RO	2010-2015		Yes	NS	No mention - No mention	No mention - No mention	No mention - No mention
Inamasu, 2014[1047]	Geriatr Gerontol Int	Clip vs coil	30 **vs 31**	1	Japan	RO	2007-2011	32 vs 36,2	NR	None	**Safe** - **Safe**	No mention - No mention	No mention - No mention
Park, 2020[1048]	J. cerebrovasc. endovasc. neurosurg.	Clip vs clip	458 **vs 170**		South Korea	RO	2012-2018		NR	None	**Safe** - **Safe**	Effective - Effective	No mention - No mention
Peng, 2019[1049]	Int J Clin Exp Med	Clip vs coil	55 **vs 62**		China	PO	2013-2018	60	NR	NS	No mention-Doubt	No mention - Effective	No mention - No mention
Pierot, 2015[1050]	AJNR Am J Neuroradiol	WEB vs WEB	30 **vs 32**	10	France	PO	2012-2014		NR	NS	**Safe** - **Safe**	No mention - No mention	No mention - No mention
Pilipenko, 2014[1051]	Zh Vopr Neirokhir Im N N Burdenko	Clip vs coil	887 **vs 187**		Russia	RO	2005-2012		NR	NS	No mention - No mention	No mention - No mention	No mention - No mention
Regli, 2002[1052]	Acta Neurochir Suppl	Clip vs coil	34 **vs 1**		Switzerland	PO	1997-2000	12	NR	NS	No mention - No mention	Effective – No mention	No mention - No mention
Roh, 2020[1053]	J Neurosurg	Coil vs SAC	64 **vs 38**	1	South Korea		2011-2017	28.9 **vs** 26.6	NR	None	**Safe** - **Safe**	Effective - Effective	No mention - No mention
Salahuddin, 2019[1054]	J Stroke Cerebrovasc Dis	Clip vs coil	14411 **vs 16659**		USA	PO	2011-2014		None	None	No mention - No mention	No mention - No mention	No mention - No mention
Salem, 2020[1055]	Neurosurgery	PED **vs SAC**	135 **vs 30**		USA	RO	2009-2017	11.5 vs 29.2	NR	None	No mention - No mention	No mention - No mention	No mention - No mention
Binboga, 2021[1056]	Interv Neuroradiol	SAC	12	1	Turkey	RO	2016 - 2020	16	None	None	Safe	Effective	No mention

© 2023 Volovici V et al. *JAMA Network Open.*

Bonafe, 2021[1057]	J NeuroIntervent Surg	FD	420	26	Multi-country	PO	2015 - 2019		Yes	Yes	No mention	Effective	No mention
Boulouis, 2021[1058]	J Neurointerv Surg	FD	55	9	Multi-country	RO	2015 - 2020	13	None	Yes	No mention	Effective	No mention
Brzegowy, 2020[1059]	Pol J Radiol	Endo	40	1	Poland	RO	2013 – 2018	35	NR	None	No mention	Doubt	No mention
Byun, 2021[1060]	J Korean Neurosurg Soc	Clip vs Endo		1	South Korea	RO	2002 - 2018		None	None	No mention - No mention	No mention - No mention	Durable - Durable
Chacón-Quesada, 2022[1061]	Neurosurg Rev	Clip	84	1	Germany	RO	2010-2020	19	Yes	Yes	No mention	No mention	No mention
Chagas Lourenco, 2022[1062]	J NeuroIntervent Surg	SAC	127	3	Brazil	RO	2016-2019	12	None	None	Safe	Effective	No mention
Cherian, 2021[1063]	Neurosurgery	WEB	91	6	USA	RO	2019-2020	5	None	None	Safe	Not effective	No mention
Achey, 2022[1064]	World Neurosurg	FD	36		USA	RO	2019-2020	6	NR	None	No mention	No mention	No mention
Choi, 2022[1065]	Yonsei Med J	FD	33	2	South Korea	RO	2018-2020	14	NR	None	No mention	No mention	No mention
Cler, 2022[1066]	J Neurosurg	PED	330	1	USA	RO	2011-2020		NR	Yes	No mention	No mention	No mention
Cohen, 2021[1067]	J Neurosurg	FD	76	1	Israel	RO	2010-2018		NR	None	No mention	Effective	No mention
Cortez, 2021[1068]	J Neurointervent Surg	WEB	91	8	USA	RO		3	None	Yes	Safe	Effective	No mention
Cui, 2021[1069]	Front Neurol	SAC	130	1	China	RO		27,2	Yes	None	Safe	No mention	No mention
Dakay, 2021[1070]	Brain circ	FD	27	1	USA	RO	***2017***-2020		None	None	No mention	Effective	Durable
Daou, 2021[1071]	Intervent Neuroradiol	SAC vs SAC	77 vs 77	1	USA	RO	2015-2019	6,1 vs 23,8	None	None	Safe - No mention	Effective - No mention	No mention - No mention
De Beule, 2021[1072]	Intervent Neuroradiol	WEB	93	1	Belgium	RO	2014-2019		None	None	Safe	Effective	No mention
De Leacy, 2021[1073]	Front Neurol	Coil	72	5	USA	PO	2016-2018		Yes	Yes	Safe	Effective	No mention
De Vries, 2021[1074]	J Neurointervent Surg	WEB	24	12	Multi-country	PO	2014-2019		None	Yes	Safe	Effective	No mention
Diestro, 2021[1075]	Neurosurgery	FD	54		USA	RO		12	None	Yes	Not safe	No mention	No mention
Dellaretti, 2021[1076]	Interdiscip Neurosurg Adv	Clip	194	1	Brasil	RO	2008 - 2020		None	None	Safe	Effective	No mention

© 2023 Volovici V et al. *JAMA Network Open.*

	Tech Case Manage												
Dietrich, 2021[1077]	Clin Neurorad	SAC	85	1	Germany	RO	2012-2018		None	None	No mention	Effective	No mention
Dutta, 2021[1078]	J. cerebrovasc. endovasc. neurosurg.	Endo	42	1	India	RO	2015-2017	10,6	NR	None	Safe	Effective	No mention
Elian, 2021[1079]	Egypt J Radiol Nucl Med	Endo	30	1	Egypt	PO	2017-2018	3	None	None	Safe	Effective	No mention
Elsheikh, 2021[1080]	Clin Neurorad	FD	18	5	Germany	RO	2013-2020	3	Yes	Yes	Safe	Effective	No mention
Enriquez-Marulanda, 2022[1081]	Neurosurgery	PED	391	1	USA	RO	2013-2019	6,4	None	None	No mention	No mention	No mention
Feng, 2022[1082]	J Chin Med Assoc	FD	125	1	China	RO	2018-2020	14	NR	NS	Safe	Effective	No mention
Fujii, 2022[1083]	Neurol Med -Chir	PED	112	1	Japan	RO			NR	Yes	No mention	No mention	No mention
Gajera, 2022[1084]	Neurointervention	WEB	63	5	Australia	RO	2017-2020	9,1	None	None	Safe	No mention	No mention
Zimmer, 2021[1085]	Am J Neuroradiol	WEB	47	2	Germany	RO	2017-2020	31,9	None	Yes	Safe	Effective	No mention
Zhong, 2021[1086]	Front Neurol	PED	38	8	China	RO	2015-2020		Yes	None	No mention	Effective	No mention
Aihara, 2021[1087]	World Neurosurg	SAC	12	4	Japan	RO	2017-2020	17	NR	None	No mention	Effective	No mention
Alpay, 2022[1088]	J Neuroradiol	WEB	22	2	Finland	RO	2014 - 2020		None	Yes	Safe	Effective	No mention
Aydin, 2022[1089]	Neurosurgery	BAC + SAC	61	4	Turkey	RO	2009 - 2020		None	None	Safe	Effective	No mention
De Beule, 2020[1090]	Intervent Neuroradiol	FD	108	1	Belgium	RO	2014 - 2019		None	None	Safe	Effective	No mention
Deuschl, 2020[1091]	Clin. Pract.	Coil vs Clip	45 vs 27	1	Germany	RO	**1998 - 2013**	6	NR	None	No mention – No mention	No mention - No mention	No mention – Durable
Lu, 2022[1092]	Intervent Neuroradiol	SAC vs SAC		1	China	RO	2014 - 2019		Yes	None	No mention – No mention	No mention – Effective	Durable – Durable
Luzzi, 2021[1093]	Acta Neurochir	Clip	149	3	Italy	RO	1990 - 2018		NR	None	No mention	Effective	Durable

© 2023 Volovici V et al. *JAMA Network Open.*

Lylyk, 2021[1094]	Neurosurgery	PED	835	1	Argentina	PO	2006 - 2019	60	None	NS	Safe	Effective	No mention
MacDonell, 2022[1095]	Brain circ.	Coil vs Coil	42 vs 26	1	USA	RO	2014 - 2018		None	None	No mention – No mention	No mention – No mention	Durable – Durable
Church, 2021[1096]	J Neurosurg	Clip vs Endo	31 vs 53	1	USA	RO	2021 - 2021		NR	Yes	No mention - No mention	No mention - No mention	No mention – No mention
Feigen, 2022[1097]	World Neurosurg	PED vs PED	141 vs 141	2	USA	RO	1990 - 2018		NR	None	Safe - Safe	Effective - Effective	No mention - No mention
Ge, 2022[1098]	Front Neurol	PED	29	1	China	RO	2018-2020	23,5	Yes	None	No mention	Effective	Durable
Girot, 2021[1099]	Am J Neuroradiol	WEB	27	1	France	RO	2010-2020		NR	Yes	Safe	No mention	No mention
Adeeb, 2021[1100]	Intervention Neuroradiologie	WEB	34	1	Japan	RO	2019 - 2020	3,4	None	None	Safe	Effective	No mention
Adeeb, 2022[1101]	Vascular neurology	WEB	572	22	Multi-country	RO	2011 - 2021	9	None	None	Safe	Effective	No mention
Akiyama, 2022[1102]	Neurosurgical review	PED	43	1	Japan	RO	2015 - 2020	22	None	None	Safe	Effective	No mention
Irie, 2022[1103]	Neurol Med Chir	**Coil vs Clip**	**417 vs 792**	38	Japan	RO	2010 - 2013	3	Yes	NS	Safe - Safe	No mention – No mention	No mention – No mention
Jang, 2022[1104]	Clin Neuroradiol	Coil	103	1	South Korea	RO	2002 - 2020		NR	NR	Safe	Effective	No mention
Algin, 2020[1105]	Interventional neuroradiology	WEB	79	1	Turkey	RO	2015 - 2021		None	None	Safe	Effective	Doubt
Jankowitz, 2022[1106]	J Neurointervent Surg	SAC	116	25	USA	PO	2015 - 2017	12	Yes	Yes	Safe	Effective	No mention
Jee, 2021[1107]	World Neurosurg	**FD**	**35**	1	South Korea	RO	2014 - 2019		NR	NR	No mention	Not effective	No mention
Jee, 2022[1108]	Neuroradiology	**FD vs FD**	**31 vs 53**	1	South Korea	PO	2019 - 2020		NR	NR	Safe - Safe	Effective – Effective	No mention - No mention
Jesser, 2021[1109]	Front Neurol	FD	150	150	Multi-country	RO	2014-2020	24	NR	None	Safe	Effective	No mention
Kan, 2022[1110]	J Neurointerv Surg	FD	38	26	Multi-country	PO			NR	Yes	Safe	Effective	No mention
Kim, 2021[1111]	World Neurosurg	Coil	36	1	South Korea	RO	2001 - 2020		Yes	Yes	No mention	Effective	No mention
Kandemirli, 2021[1112]	Clin Neurorad	FD vs FD	24 vs 21	3	Multi-country	RO	2010 - 2020		NR	None	Safe - Safe	Effective - No mention	No mention - Durable

© 2023 Volovici V et al. *JAMA Network Open.*

Kachhara, 2021[1113]	J Neurosci Rural Pract	Clip	35	3	India	RO		6	None	None	No mention	No mention	No mention
Kidani, 2022[1114]	Acta Neurochir (Wien)	Coil	98	6	Japan	RO	2000 - 2018		NR	None	Safe	Effective	No mention
Jiang, 2022[1115]	Clin Neurol Neurosurg	SAC vs SAC	89 vs 145	1	China	RO	2014 - 2018	30	Yes	None	Safe - No mention	No mention - No mention	Durable - No mention
Jin, 2022[1116]	Intervent Neuroradiol	Coil vs SAC	62 vs 28	1	China	RO	2014 - 2019		None	None	Safe – No mention	Effective - No mention	No mention - No mention
Kang, 2022[1117]	J Neurointervent Surg	PED vs PED	587 vs 431 vs 153		China	RO	2014 - 2019		Yes	None	No mention - No mention - Doubt	No mention - No mention - No mention	No mention - No mention - No mention
Becske, 2017[1118]	Neurosurgery	PED	107	10	USA	PO	2008 - 2009	60	Yes	NS	Safe	Effective	No mention
Bender, 2019[1119]	World Neurosurg	FD	57		USA	PO	2011 - 2018	19	NR	Yes	Safe	Effective	No mention
Briganti, 2014[1120]	Eur J Radiol	FD	35	1	Italy	RO	2008 - 2012		NR	None	Safe	Effective	No mention
Ahn, 2014[1121]	AJNR Am J Neuroradiol	Coil	43	1	South Korea	PO	2003 - 2013		None	None	Safe	Effective	Doubt
Al-Kasab, 2019[1122]	Interventional neurology	PED	36	1	USA	PO	2015 - 2017		None	None	Safe	Effective	No mention
Alanen, 2018[1123]	Acta neurochirurgica	Coil	491	1	Austria	RO	2000 - 2014		None	None	Safe	No mention	No mention
Andaluz, 2008[1124]	Skull base	Clip	75	1	USA	RO	2000 - 2004	41	None	None	Safe	No mention	No mention
Chen, 2018[1125]	World Neurosurg	Clip	26	1	China	RO	2014 - 2017	22	Yes	Yes	Safe	No mention	No mention
Cavalcanti, 2017[1126]	World Neurosurg	Clip vs Clip	70 vs 47	1	Brasil	RO	2013 - 2016	13.3 ***vs 13***	NR	None	Safe - Safe	Effective - Effective	No mention - No mention
Chalouhi, 2013[1127]	Neurosurgery	Clip vs Clip	40 vs 47	1	USA	RO	20014 - 2010	12	None	None	Safe -Doubt	Effective - Effective	No mention - No mention
No mention Chalouhi, 2013[1128]	AJNR Am J Neuroradiol	SAC	37	1	USA	RO	2005 - 2011	11.3	Yes	Yes	Safe	Effective	No mention
Cheng, 2006[1129]	Minimally Invasive Neurosurg	Clip	40	1	Taiwan	RO	1999 - 2004		NR	NS	No mention	Effective	No mention
Chhabra, 2005[1130]	Surg Neurol	Clip	28	1	India	RO	1999 - 2003		NR	NS	No mention	Effective	No mention

© 2023 Volovici V et al. *JAMA Network Open.*

Cho, 2014[1131]	Neuroradiology	Coil	48	1	South Korea	RO	2002 - 2013		Yes	None	Safe	Effective	No mention
Cho, 2012[1132]	Neurosurgery	Coil	36	1	South Korea	RO	2009 - 2011		Yes	None	Safe	No mention	No mention
Hanel, 2022[1133]	J Neurointerv Surg	PED	141	23	USA	PO	2014 - 2015	36	Yes	Yes	Safe	Effective	No mention
Kabbasch, 2016[1134]	J. vasc. interv. radiol. neurol.	PED vs PED	19 vs 18		Germany	RO	2011 - 2013		Yes	Yes	Safe - Safe	Effective - Effective	No mention - No mention
Jin, 2022[1135]	Intervent Neuroradiol	Coil vs SAC	62 vs 28	1	China	RO	2014 - 2019	17.5	None	None	Safe - Safe	Effective - Effective	No mention - No mention
Jeon, 2003[1136]	J Korean Med Sci	Clip	27	1	South Korea	RO	1999 - 2001	60	NR	NS	Safe	Effective	No mention
Batista, 2002[1137]	Interv Neuroradiol	Coil	102	1	France	PO	1993 - 1997	24	NR	NS	Safe	Effective	Doubt
Bae, 2021[1138]	Am J Neuroradiol	FD	64	1	South Korea	RO	2014 - 2019	6	NR	NS	No mention	Effective	No mention
**Ba, 2021[1139]**	Am J Trans Res	Clip vs Coil	30 vs 38	1	China	RO	2017 - 2020	17.5	NR	None	Doubt - Safe	Effective - Effective	No mention - No mention
Garg, 2022[1140]	J NeuroIntervent Sur	Clip vs Coil	93 vs 255	1	USA	RO	2016 - 2018		None	Yes	Safe - Safe	No mention – No mention	No mention - No mention
Furtado, 2021[1141]	J Neurosci Rural Pract	Clip vs Coil	27 vs 7	1	India	RO	2007 - 2019	24	None	NS	Doubt – No mention	Effective - No mention	No mention – No mention
Goertz, 2021[1142]	Nature	Coil vs SAC	220 vs 64	1	Germany	RO	2010 - 2019	6	Yes	Yes	No mention - Safe	No mention - Effective	No mention - No mention
Gündogmus, 2022[1143]	Intervent Neurorad	PED vs FD	55 vs 83	1	Turkey	PO	2012 - 2019	36	None	None	Safe - Safe	Effective - Effective	No mention - No mention
Alpay, 2021[1144]	Journal of neurointervention al radiology	FD	110	5	Finland	RO	2012 - 2019	8	None	Yes	Not safe	No mention	Doubt
Koltz, 2014[1145]	Journal of clinical neuroscience	Coil	93	1	USA	RO	2010 - 2010	16	None	None	Safe	Effective	No mention
Kang, 2009[1146]	Journal of neurosurgery	Coil	88	1	South Korea	RO	1999 - 2008	25	None	None	Safe	Effective	No mention
Lin, 2013[1147]	Surg Neurol Int	PED	41	1	USA	PO	2011 - 2012		NR	None	Safe	No mention	No mention
Li, 2022[1148]	J Neurol Surg Part A Cent Eur Neurosurg	SAC vs SAC vs SAC	277 vs 138 vs 93	1	China	RO	2009 - 2013		NR	None	Safe – Not safe – No mention	Effective - Not effective – No mention	No mention – No mention - No mention

© 2023 Volovici V et al. *JAMA Network Open.*

Durst, 2014[1149]	J neurosurg	Coil	100 vs 52 vs	1	USA	RO	2006 - 2012	20	NR	Yes	Safe	Effective	No mention
Kunert, 2021[1150]	Sci Rep	FD vs FD vs Coil	52 vs 33 vs 19		Poland	RO	2009 - 2016	61	NR	None	Safe - Safe - Safe	Effective - Effective - Effective	No mention - No mention - No mention
Lebeaupin, 2021[1151]	Journal of clinical medicine	SAC	64	1	France	RO	2011 - 2018	79	None	None	Safe	Effective	No mention
Lee, 2021[1152]	The neuroradiology Journal	FD vs BAC	14 vs 12	1	Canada	RO	2008 - 2018	27.1 vs 17.3	None	None	Safe - Safe	Effective - Effective	Durable - Durable
Saal-Zapata, 2022[1153]	Surg Neurol Int	Endo	33	1	Peru	RO	2017 - 2019	11.1	None	None	Safe	Effective	Doubt
Kim, 2014[1154]	Interventional neuroradiology	Coil	53	1	South Korea	PO	2002 - 2013		None	None	Safe	Effective	No mention
Kim, 2021[1155]	J. Korean Neurosurgery	Clip vs Clip	21 vs 43	1	South Korea	RO	2005 - 2016	41.7 vs 55.6	None	None	Doubt – Doubt	Doubt – Doubt	No mention - No mention
Turhon, 2022[1156]	Clinical Neurology	PED	104	14	China	RO	2014 - 2019		Yes	None	Safe	Effective	No mention
Vollherbst, 2022[1157]	J. neurointervention al surgery	FD	60	10	Multi-country	RO	2020 - 2020	4.9	None	None	Safe	No mention	No mention
Wang, 2022[1158]	Stroke vascular neurology	PED vs PED	637 vs 685		Multi-country	RO	2014 - 2019	8.7	Yes	None	Doubt - Safe	Doubt - Effective	No mention - No mention
Vieira, 2022[1159]	Acta neurochirurgica	Clip	300	1	Brazil	RO	2014 - 2021	16.5	None	None	Safe	No mention	No mention
Wan, 2022[1160]	Interventional neuroradiol	Coil	101	1	South Korea	RO	2010 - 2019		Yes	None	No mention - No mention	Effective - No mention	No mention - No mention
Uche, 2021[1161]	Asian J Neurosurgery	Coil vs Clip	11 vs 99	1	Nigeria	RO	2010 - ***2016***		None	None	No mention - No mention	Doubt – Questionabel	No mention - No mention
Mori, 2011[1162]	Minim Invas Neurosurg	Clip	100	1	Japan	PO		***3***	NR	None	Safe	No mention	No mention
McDougall 2022[1163]	J Neurosurg	FD	145	25	USA	PO	2013 - 2016	12	Yes	Yes	Safe	Effective	No mention
Martinez-Galdamez, 2019[1164]	J NeuroIntervent surg	PED	50	7	Spain	PO	2015 - 2015	12	Yes	Yes	Safe	Effective	No mention
Martinez-Galdamez, 2021[1165]	J NeuroIntervent surg	FD	57	19	Spain	RO	2020 ***-2021***		None	Yes	Safe	No mention	No mention
Mascitelli, 2021[1166]	J Neurosurg	Endo vs Clip	55 vs 32	3	USA	PO	2017- ***2019***	12	Yes	Yes	Safe - Safe	Doubt - Effective	No mention - Durable

© 2023 Volovici V et al. *JAMA Network Open.*

McEachern, 2022[1167]	Interventional neuroradiol	SAC	196	6	Canada		2013 - 2019	30	None	None	Safe	Effective	No mention
Mitchell, 2005[1168]	Surg Neurol	Clip	47	1	UK	PO	1993 - 2002	12	NR	NS	No mention	No mention	No mention
Mizunari, 2011[1169]	J Nippon Med Sch	Clip	41	1	Japan		1998 - 2009		NR	NS	No mention	No mention	No mention
Mohlenbruch, 2017[1170]	AJNR	FD	42	6	Germany	PO	2015 - 2016	12	Yes	Yes	Safe	Effective	Doubt
Mokin, 2018[1171]	J NeuroIntervent Surg	FD	49	3	USA	RO	2011 - 2017	3	None	Yes	Safe	Effective	No mention
Mori, 2019[1172]	J Neurosurg	Clip	149	3	Japan	RO	2005 - 2016	132	NR	None	No mention	No mention	Durable
Mori, 2018[1173]	Operative Neurosurg	Clip	63	1	Japan	PO	2005 - 2014	62.4	None	None	Safe	Effective	Durable
Mouchtouris, 2022[1174]	J Neurosurg	WEB	110	1	USA	RO	2019 - 2021	6	NR	Yes	Doubt	No mention	No mention
Naamani, 2022[1175]	J Neurosurg	SAC vs WEB	85 vs 63	6	USA	RO	2011 - 2019	18.6 vs 10.8	NR	Yes	Safe - Safe	No mention - Effective	No mention - Durable
Strittmatter, 2022[1176]	J Clin Med	SAC	156	1	Germany	RO	2013 - 2020	12	None	Yes	Safe	No mention	No mention
Salem, 2021[1177]	J NeuroIntervent Surg	FD	87	6	Multi-country	RO	2011 - 2018	15.6	None	None	Safe	Effective	No mention
Starke, 2021[1178]	J NeuroIntervent Surg	Coil	851	66	Multi-country	PO	2016 - 2018	42	Yes	Yes	**Doubt**	No mention	No mention
Li, 2021[1179]	Front Neurol	Endo	77	1	China	RO	2008 - 2019	12	NR	None	Safe	Effective	Durable
Li, 2021[1180]	Front Neurol	**SAC vs Coil**	**162 vs 161**	**10**	**China**	**RCT**	2018 - 2019		NR	Yes	Safe - Safe	Effective - Effective	No mention - No mention
Li, 2022[1181]	J Clin Neurosci	FD	110	1	**China**	**RO**	**2014 - 2019**	**12**	Yes	None	Safe	Effective	No mention
Xenofontos, 2022[1182]	The Neuroradiology Journal	Coil		**1**	**UK**	**RO**	2008 **- 2019**	19.7	None	None	Safe	Effective	No mention
Wu, 2021[1183]	Clin Neuroradiology	SAC **Vs Clip**	**21 vs 18**	**1**	China	**RO**	2013 - 2018	6	NR	None	Safe - Safe	No mention - No mention	No mention - No mention
Yatomi, 2022[1184]	The Neuroradiology Journal	SAC	130	***1***	Japan	***RO***	2016 - 2019	28.9	None	Yes	No mention	No mention	No mention

© 2023 Volovici V et al. *JAMA Network Open.*

Yavuz, 2012[1185]	American Journal of Neuroradiology	Coil	188	***1***	Turkey	***RO***	2006 - 2011	6	NR	NS	Safe	No mention	No mention
Sturiale, 2022[1186]	Neurosurg Rev	Clip vs Coil	**340 vs 71**	**5**	Italy	***RO***	2015 - 2019	23	Yes	None	Safe - Safe	No mention - No mention	No mention - No mention
Styczen, 2021[1187]	Neuroradiol J	Endo	**35**	**9**	Multi-country	***RO***	2003 - 2020	44	NR	NS	Safe	Effective	No mention
Sukun, 2022[1188]	Turk Neurosurg	Clip vs Coil	**78 vs 109**	**1**	Turkey	***RO***	2012 - 2016	12	NR	NS	Safe - Safe	Effective - Effective	No mention - No mention
Li, 2022[1189]	J Clin Neurosci	FD	28	1	China	RO	2017 - 2020	12	Yes	None	Safe	Effective	No mention
Lefevre, 2022[1190]	J Neurointerv Surg	SAC	105	11	France	PO	2016 - 2018	16	Yes	Yes	Safe	Effective	No mention
Lee, 2022[1191]	J Chin Med Assoc	FD	70	2	Taiwan	RO	2018 - 2020	15	Yes	None	Safe	Effective	No mention
Lam, 2021[1192]	J Clin Neurosci	Bypass	40	1	USA	RO	2013 - 2018	14	None	None	No mention	No mention	No mention
Hong, 2021[1193]	BMC Neurol	SAC	63		China	RO	2015 - 2017	24.3	Yes	None	Safe	Effective	No mention
Adeeb, 2022[1194]	Radiology	WEB	683	22	Multi-country	RO	2011 - 2021	9	NR	Yes	No mention	No mention	No mention
Nakamura, 2005[1195]	Surgical neurology	Clip	487	12	Japan	RO	1997 - 2001	12	None	NS	Safe	Effective	No mention
Nanda, 2016[1196]	World neurosurgery	Clip	26	1	USA	RO	1994 - 2015	67	None	None	Safe	Effective	No mention
Ozpeynirci, 2021[1197]	Interventional neuroradiology	**FD vs Coil**	**23 vs 18**	**1**	**Germany**	**RO**	**2013 – 2019**		None	Yes	Safe - Safe	Effective - Effective	No mention - No mention
Pagano, 2021[1198]	J Neurointervent surg	WEB vs WEB		1	France	RO	**2015 - 2019**	12	None	Yes	Safe - Safe	Effective - Effective	No mention - No mention
Peng, 2020[1199]	Clin neuroradiol	Endo	231	2	China	RO	2011 - 2016	60	Yes	None	Safe	Effective	No mention
Rahmanian, 2017[1200]	World neurosurgery	Clip	52	1	Iran	RO	2010 - 2016	6	None	None	Safe	Effective	No mention
Rahmanian, 2018[1201]	World neurosurgery	Clip	26	1	Iran	RO	2010 - 2016	6	None	None	Safe	Effective	No mention
Sharma, 2008[1202]	Clinical neurology and neurosurgery	Clip	177	1	India	RO	1995 - 2007		NR	NS	Safe	Effective	No mention

© 2023 Volovici V et al. *JAMA Network Open.*

Shivhare, 2021[1203]	Turkish neurosurgery	Clip	28	1	India	RO	2011 - 2015	6	NR	NS	No mention	No mention	No mention
Sim, 2022[1204]	J Neurointervent surg	Endo	40	5	South Korea	RO	2004 - 2020	84	Yes	None	No mention	No mention	No mention
Simgen, 2022[1205]	The neuroradiology journal	FD	93	1	Germany	RO	2010 - 2019	101	None	None	Safe	Effective	No mention
Song, 2021[1206]	Neurointervention	Coil	58	1	South Korea	RO	2019 - 2020	17.7	Yes	Yes	Safe	Effective	No mention
Oishi, 2015[1207]	J NeuroIntervent Surg	Coil	375	4	Japan	RO	2001 - 2013	39.1	NR	None	Safe	No mention	No mention
Reisch, 2014[1208]	World Neurosurg	Clip	793			RO			NR	NS	Safe	Effective	No mention
Bhogal, 2022[1209]	Intervent Neuroradiol	FD	60	4	UK	RO	2018 - 2020		Yes	Yes	Safe	Effective	No mention
Yıldırım, 2021[1210]	Intervent Neuroradiol	SAC	30	1	Turkey	RO	2013 - 2019	17.8	None	None	Doubt	Doubt	No mention
Winkler, 2021[1211]	Acta Neurochir	Coil vs Clip	26 vs 16	1	USA	RO	2014 - 2019	12.7 vs 11.8	NR	None	Safe - Safe	Effective - Effective	No mention - No mention
Zaitoun, 2022[1212]	Intervent Neuroradiol	Coil	42	1	Switzerland	RO	2010 – 2015	32.3	None	None	Safe	Effective	No mention
Scerrati, 2021[1213]	J Integr Neurosci	Endo	250	6	Italy	RO	2015 – 2020		None	None	Safe	Effective	Durable
Shi, 2021[1214]	Neurol Res 2021	SAC	42	1	China	RO	2015 - 2019	28	Yes	None	Safe	Effective	Durable
Qi, 2021[1215]	Biomed Res Int	SAC	25	1	China	RO	2010 - 2020		NR	None	Safe	Effective	No mention
Hong, 2022[1216]	J Neurosurg	Clip	110	1	South Korea	RO	2013 - 2018		NR	NS	Safe	Effective	No mention
Hanalioglu, 2022[1217]	Acta Neurochir	Clip vs Coil	80 vs 61	1	USA	RO	2006 - 2019	93.9 ***Vs 73.3***	NR	NS	Safe - Safe	Effective - Effective	No mention - No mention
Harada, 2013[1218]	J Neurointervent Surg	Coil	92	1	Japan	RO	2008 - 2010		NR	NS	Safe	Effective	Durable
Onay, 2021[1219]	Interv Neuroradiol	SAC vs SAC	32 ***vs 16***	1	Turkey	RO	2016 - 2020	17.41	None	None	Safe - Safe	Effective - Effective	No mention – No mention
Pérez, 2022[1220]	J Clin Med	SAC	115	10	Multi-country	PO	2015 - **2018**	12.8	Yes	Yes	Safe	Effective	No mention
Pierot, 2021[1221]	J Neurointervent Surg	WEB	140		Multi-country	PO		36.8	Yes	Yes	Safe	Effective	Durable

© 2023 Volovici V et al. *JAMA Network Open.*

Profeta, 2004[1222]	Childs Nerv Syst	Clip	52	1	Italy	RO	2000 - 2003		NR	NS	Safe	Effective	No mention
Ho, 2015[1223]	Journal of neurological surgery	Clip	62	2	Multi-country	RO	2003 - 2012	6	None	None	Safe	No mention	No mention
Murayama, 2006[1224]	J. neurosurgery	Coil	112	2	Multi-country	PO	2001 - 2004		None	None	Safe	No mention	No mention
Pumar, 2021[1225]	Front Neurol	FD	25	1	Spain	RO	2020 - 2020	3.45	None	Yes	Safe	Effective	No mention
Link, 2021[1226]	J Clin Neurosci	PED vs PED	46 vs 94	1	USA	RO	2012- ***2017***	12	None	None	Safe - Safe	Effective - Effective	No mention - No mention
Onay, 2022[1227]	Acad Radiol	Endo vs Endo	20 ***vs 89***	1	Turkey	RO	2016 - 2020	6	None	None	Safe - Safe	Effective - Effective	No mention - No mention
Winters, 2021[1228]	Front Neurol	FD	32	3	Germany	RO	2020 - 2021		None	Yes	Safe	Effective	No mention
Nouri, 2021[1229]	Oper Neurosurg (Hagerstown)	Bypass	36	1	USA	RO	2009 - 2020	12	None	None	Safe	Effective	Safe
Ni, 2021[1230]	World Neurosurg	Coil **vs SAC**	52 vs 29	1	China	RO	2015 - 2020	6	None	None	Safe - Safe	Effective - Effective	No mention - No mention
Ozaki, 2022[1231]	Neurosurgery	Coil	49	1	Japan	RO	2017 - 2021		Yes	None	Safe	Effective	No mention
Misra, 2021[1232]	Neurol India	Clip	134	1	India	RO	1996 - 2019		None	None	Doubt	No mention	No mention
You, 2021[1233]	BioMedical eng	SAC vs SAC	71 vs 73	1	China	PO	2016 - 2019	12	None	None	No mention - Safe	No mention - Effective	No mention - No mention
Yoon, 2017[1234]	Intervent neuroradiol	Coil	56	1	South Korea	RO	2008 - 2016		Yes	None	Safe	Effective	Doubt
Zhang, 2022[1235]	Frontiers neurol	PED	652	14	China	RO	2014 - 2019		Yes	None	Safe	Effective	No mention
Yeon, 2021[1236]	Clin neuroradiol	Coil	54	1	South Korea	RO	2008 - 2010		NR	None	No mention	No mention	Durable
Zheng, 2015[1237]	Medicine	Clip	59	1	China	RO	2005 - 2014		Yes	NS	Safe	Effective	No mention
Xue, 2021[1238]	Chin Neurosurg J	SAC **vs SAC**	142 vs 93	1	China	RO	2014 - 2017	12	Yes	None	Safe – No mention	Effective - No mention	No mention - No mention
Zhao, 2018[1239]	J of craniofacial surg	Clip	31	1	China	RO	2015- 2017		NR	NS	Safe	Effective	No mention
Kis, 2007[1240]	Clin neuroradiol	SAC	71	2	Germany	RO	2003- 2006	10	Yes	Yes	Safe	Effective	No mention

© 2023 Volovici V et al. *JAMA Network Open.*

Kim, 2021[1241]	AJNR	Coil	64	1	South Korea	RO	2015 - 2020		NR	NS	Safe	Effective	No mention
Yamahata, 2014[1242]	Neurosurg Rev	Clip	103	1	Japan	RO	2002 - 2009		NR	NS	Safe	Effective	No mention
Kiran, 2014[1243]	Operative neurosurg	Clip	39	1	Finland	RO	1997 - 2009	3	None	None	Safe	Effective	No mention
Yang, 2008[1244]	Operative neurosurg	Clip	25	1	USA	RO	2005 - 2007		NR	NS	Safe	Effective	No mention
Sweid, 2020[1245]	Neurosurgery	FD	598	1	USA	RO	2010 - 2019	22.8	NR	NS	Safe	Effective	No mention
Sweid, 2020[1246]	Neurosurgery	SAC	69	4	USA	RO	2018 - 2019	4	NR	NS	Safe	Effective	No mention
Murphy, 2001[1247]	Radiology	Coil	60	7	Multi	PO			None	None	Safe	Effective	No mention
Nussbaum, 2022[1248]	Acta neurosurgery	Clip	1750	1	Austria	RO	1997 - 2019	105.6	Yes	None	Safe	Effective	No mention
Won, 2015[1249]	Neurol Res	SAC	26	1	South Korea	RO	2006 - 2013	33	None	None	No mention	Effective	No mention
Won, 2021[1250]	Sci Rep	Clip	287	1	Germany	RO	2007 - 2017	6	Yes	None	Safe	Effective	No mention
Wong, 2022[1251]	Can J Neurol Sci	Clip **vs Coil**	95 vs 287	1	Canada	PO	2002 - 2017	12	Yes	None	No mention - No mention	Effective – Effective	No mention - No mention
Peng, 2022[1252]	Front Neurol	FD vs SAC	**34 vs 43**	1	China	RO	2016 - 2020	28.5 **vs 27.4**	Yes	None	Doubt – Doubt	Effective – Effective	No mention - No mention
Pflaeging, 2021[1253]	World neurosurgery	Coil **vs Clip**		1	Germany	RO		6	None	None	Safe - Safe	Effective – Effective	No mention - No mention
Yao, 2021[1254]	World Neurosurg	SAC	**101**	1	China	RO	2013 - 2018	41.6	None	None	Safe	Effective	No mention
Yeon, 2021[1255]	Neurosurgery	LVIS	**124 vs 124**	1	South Korea	RO	2015 - 2019		None	None	Doubt – Doubt	Doubt – Doubt	No mention - No mention
Thanabalasundaram, 2021[1256]	Br J Neurosurg	Atlas	100			RO		6	NR	None	Safe	Effective	No mention
Xue, 2020[1257]	Front Neurol	Clip	31	1	China	RO	2014 - 2018	25.3	Yes	None	Safe	Effective	No mention
Xue, 2021[1258]	Front Neurol	SAC	40	1	China	RO	2014 - 2019	29.6	Yes	None	Safe	Effective	Durable
Wu, 2022[1259]	Front Neurol	***Endo***	62	1	China	RO	2014 - 2020	27.5	Yes	None	Safe	Effective	Durable

© 2023 Volovici V et al. *JAMA Network Open.*

Liu, 2021[1260]	Chinese Neurosurgical Journal	PED **vs SAC**	**51 vs 51**	1	China	RO	2014 - 2019		Yes	None	Safe - Safe	Effective – Effective	No mention - No mention
Luo, 2022[1261]	J investig Med BMJ	Clip vs BAC	**65 vs 65**	1	China	PO	2017 - 2020		None	None	Safe - Safe	Effective – Effective	No mention - No mention
Ni, 2020[1262]	Interventional Neuroradiology	SAC	110	1	China	RO	2015 - 2019	18.5	None	None	Safe	Effective	No mention
Oishi, 2018[1263]	Neurol Med Chir	PED	94	1	Japan	RO	2012 - 2017		NR	Yes	Safe	Effective	No mention
Nickele, 2022[1264]	Operative Neurosurg	Coil	145	1	USA	PO	2003 - 2012		Yes	Yes	Safe	Effective	No mention
Luzzi, 2022[1265]	Interdisciplinary Neurosurgery	Clip + bypass	58	3	Italy	RO	1993 - 2021		NR	None	No mention	No mention	Durable
Ma, 2022[1266]	Frontiers neurology	PED vs Endo	37 vs 37	3	China	RO	2016 - 2020	12 **vs 8**	Yes	None	Safe - Safe	Effective – Effective	No mention - No mention
Piano, 2021[1267]	Journal of neurosurgical science	FD	108	34	Italy	RO	***2016 - 2018***	3	None	None	Safe	Effective	No mention
Porto, 2021[1268]	World neurosurgery	FD	84	5	USA	RO	***2007 - 2020***	27.5	None	Yes	Safe	Effective	No mention
Zhang, 2021[1269]	Front Neurol	PED vs Endo	99 vs 41	1	China	RO	2018 - 2020		Yes	None	Safe - Safe	Effective – Effective	No mention - No mention
Zhang, 2021[1270]	J Clin Neurosci	Endo	27	1	China	RO	2016 - 2020	3.5	Yes	None	Safe	Effective	No mention
Zhao, 2022[1271]	World Neurosurg	Endo vs Endo	**168 vs 166**	10	China	RCT	2017 - 2019	12	Yes	Yes	Safe - Safe	Effective – Effective	No mention - No mention
Zarco, 2021[1272]	Clin Radiol	FD	**122**	1	Spain	RO	2013 - 2014	12	NR	None	Safe	Effective	No mention
You, 2022[1273]	Front Neurol	SAC	**114**	1	China	RO	2016 - 2018		Yes	None	Doubt	Doubt	No mention
Szmygin, 2021[1274]	Polish Journal of Neurology and Neurosurger	Coil	**44**	1	Poland	RO	2015 - 2020		None	None	Doubt	Effective	No mention
Take, 2021[1275]	Surgical Neurology Internationa	Coil vs Clip	9 vs 28	1	Japan	RO	2012 - 2018		None	None	No mention - No mention	Effective – Effective	No mention - No mention
Tang, 2022[1276]	Interventional neuroradiologu	Coil	28	1	China	RO	2016 - 2020	22.9	Yes	None	Safe	Effective	No mention

© 2023 Volovici V et al. *JAMA Network Open.*

Taqi. 2021[1277]	Cerebrovascular diseases	PED	26	1	USA	RO	2019 - 2020		None	None	Safe	Effective	No mention
Suzuki, 2022[1278]	Neurol Med Chir	Coil vs FD	**24 vs 21**	1	Japan	RO	2009 - 2019	55 **vs 23.1**	NR	None	Safe - Safe	Effective – Effective	No mention - Durable
Suyama, 2022[1279]	Neuroradiology	FD	**36**	1	Japan	RO	2020 - 2021		None	None	Safe	Effective	No mention
Hellstern, 2021[1280]	Front Neurol	FD	**54**	1	Germany	RO	2012 - 2019		NR	Yes	Safe	Effective	No mention
Sturiale, 2017[1281]	Acta neurochirurgica	Clip vs Clip	**37 vs 31**	1	Italy	RO	2011 - 2014	37.3 vs 20.9	NR	None	Safe - Safe	Effective – Effective	No mention - No mention
Spiotta, 2021[1282]	Front Neurol	Endo	905	68	Multi-country	PO	2016 - 2018	12	NR	None	Safe	Effective	Durable
Seo, 2022[1283]	World Neurosurg	Clip vs Clip	82 vs 43	1	South Korea	RO	2003 - 2020	28.5 **vs 28.9**	NR	None	Safe - Safe	Effective – Effective	No mention - No mention
Sato, 2021[1284]	Turk Neurosurg	SAC vs SAC	17 vs 32	1	Japan	RO	2010 - 2019		NR	NS	Safe - Safe	Effective – Effective	No mention - No mention
Sano, 2010[1285]	Acta Neurochir Suppl	Clip	258	1	Japan	RO	1975 - 2008		NR	NS	Safe	Effective	No mention
Salem, 2022[1286]	Neurosurgery	FD	61	4	USA	PO	2020 - 2021	6.1	NR	Yes	Safe	Effective	No mention
Rautio, 2021[1287]	J Neuroradiol	FD	29	2	Finland	RO	2019 - 2020	6	None	None	Safe	Effective	No mention
Qureshi, 2001[1288]	J Neurosurg	Coil	80	1	USA	RO	1990 - 1999		NR	NS	Safe	Effective	No mention
Qin, 2021[1289]	Front Neurol	Endo	152	1	China	RO	2014 - 2020	6.8	Yes	None	Safe	Effective	Durable
Pumar, 2021[1290]	Front Neurol	LE	101	1	Spain	RO	2004 - 2016		NR	NS	Safe	Effective	Durable
Ohshima, 2017[1291]	Nagoya J Med Sci	BAC	152	1	Japan	RO	2013 - 2016		NR	None	Safe	Effective	No mention
Heye, 2015[1292]	J Neurointervent Surg	BAC	29	1	Germany	RO	2007 - 2013		NR	None	Safe	Effective	No mention
Harris, 2021[1293]	Br J Neurosurg	Coil vs Clip	113 vs 19	1	UK	RO	2012 - 2018	12	NR	None	No mention - No mention	No mention - No mention	Not durable - Durable
Hanel, 2021[1294]	Neurosurgery	SAC	35	25	USA	PO	2015 - 2016	12	None	Yes	Safe	Effective	No mention
Goertz, 2021[1295]	Neurosurgery	WEB vs Clip	63 vs 103	3	Germany	RO	2011 - 2019	6 vs 7.4	None	Yes	Safe - Safe	Effective – Effective	Durable - Durable

© 2023 Volovici V et al. *JAMA Network Open.*

Burrows, 2015[1296]	J Neurointervent Surg	FD	93	1	USA	RO	2009 - 2013	17	Yes	Yes	Safe	Effective	No mention
Teixeira, 2021[1297]	arq bras neurosurgery	FD	81	1	Brazil	RO	2018 - 2019		None	None	Safe	Effective	No mention
Tessitore, 2022[1298]	Interventional neuroradiology	Coil		1	Italy	RO	2015 - 2018	12	None	None	Safe - Safe	Effective – Effective	No mention - No mention
Wang, 2018[1299]	Biomes res	Clip	118	1	China	PO	2011 - 2015	17	None	None	Safe	No mention	No mention
Wang, 2016[1300]	nt J Clin Exp Med	Clip	26	1	China	RO	2012 - 2013	12	None	None	Safe	Doubt	No mention
Patel, 2016[1301]	Interventional neuroradiology	PED	25	1	USA	RO	2015- 2015		None	None	Safe	Effective	No mention
Yin, 2020[1302]	Neurol india	Endo vs Clip	26 vs 29	1	China	PO	2011 - 2015	29.6	Yes	None	Safe - Safe	Effective – Effective	No mention - No mention
Zhitao, 2010[1303]	Neurol india	Clip	42	1	China	RO	1993 - 2008	69.6	NR	NS	Safe	Effective	No mention
Bhatoe, 2009[1304]	Neurol india	Clip	52	1	India	PO	2003 - 2009	21	NR	NR	Safe	Effective	No mention
Tang, 2018[1305]	World neurosurgery	Clip vs Clip	356 vs 301	2	Multi	RO	2009 - 2016	6	None	None	Safe - No mention	No mention - No mention	No mention - No mention
Wang, 2018[1306]	Clinical neuroscience	Clip vs Clip	80 vs 96	**1**	China	RO	2010 - 2015	12	None	None	Safe - Safe	No mention - No mention	No mention - No mention
Vignesh, 2022[1307]		BAC	188	1	India	RO	2014 - 2019		None	None	Safe	No mention	No mention
Molyneux, 2015[1308]	Lancet	Clip vs Coil	1070 vs 1073	43	UK	RCT	1994 - 2002	120	Yes	None	Safe - Safe	Effective – Effective	No mention - Durable
Molyneux, 2002[1309]	Lancet	Clip vs Coil	1070 vs 1073	43	UK	RCT	1994 - 2002	12	Yes	Yes	No mention - Safe	Effective – Effective	No mention - Durable
White, 2011[1310]	Lancet	Coil vs Coil	234 vs 247	24	Multi-country	RCT	2004 - 2007	***18***	Yes	Yes	No mention - No mention	No mention - No mention	Durable - No mention
White, 2008[1311]	Am J Neuroradiol	Coil vs Coil	249 vs 250	24	Multi-country	RCT	2004 - 2007	3	Yes	NR	Safe - Safe	Effective – Effective	No mention - No mention
Qin, 2017[1312]	J Neurosurg	Endo	55	1	China	RO	2007 - 2014	6	NR	None	No mention	Doubt	No mention
Pumar, 2018[1313]	J Neurointervent Surg	FD	104	4	Spain	RO	2008 - 2013	6	NR	None	Safe	Effective	No mention
Pumar, 2017[1314]	Neurosurgery	FD	157	4	Spain	RO	2008 - 2013	6	NR	None	No mention	Effective	No mention

© 2023 Volovici V et al. *JAMA Network Open.*

Puffer, 2014[1315]	Am J Neuroradiol	FD	44	1	USA	PO	2009 - 2013	6	NR	Yes	Safe	Effective	No mention
Poncyljusz, 2015[1316]	J Neurointervent Surg	SAC	78	5	Poland	RO		6	NR	Yes	Safe	Effective	Durable
Piske, 2009[1317]	Neurosurgery	PED	69	1	Brazil	RO	2002 - 2006		NR	NR	Safe	Effective	No mention
Qu, 2009[1318]	Neuroradiol J	Endo	43	1	China	RO	2000 - 2007	21.1	NR	NR	No mention	Effective	No mention
Quadros, 2007[1319]	Am J Neuroradiol	Endo	55	1	France	RO	2001 - 2006	12	NR	NR	Safe	Effective	No mention
Quintana, 2019[1320]	Interv Neuroradiol	Atlas	30	1	Spain	RO	2015 - 2017		None	None	Safe	Doubt	No mention
Raftopoulos, 2000[1321]	J Neurosurg	Coil vs Clip	60 vs 49	1	Belgium	PO	1996 - 1999	***6***	NR	NR	No mention - No mention	Effective – Effective	No mention - No mention
Piotin, 2010[1322]	Stroke	**SAC vs Coil**		1	France	RO	2002 - 2009		NR	None	Safe - Safe	No mention - No mention	Durable - Durable
Raftopoulos, 2003[1323]	Neurosurgery	Coil vs Clip	38 vs 39	1	Belgium	PO	1996 - 2001	3	NR	NR	Doubt – Doubt	Effective – Effective	No mention - No mention
Piotin, 2018[1324]	J Neurointervent Surg	Coil	63	9	Multi-country	PO	2011 - 2013	36	Yes	Yes	Safe	Effective	No mention
Pierot, 2016[1325]	Am J Neuroradiol	WEB	62	10	France	PO	2012 - 2014	12	Yes	Yes	Safe	No mention	No mention
Pierot, 2015[1326]	Neurosurgery	WEB	113	10	Multi-country	PO	2011 - 2014	12	NR	Yes	Safe	Effective	No mention
Iskandar, 2011[1327]	Intervent Neuroradiol	Coil	934	1	Denmark	RO	1999 - 2009	9	NR	NR	Doubt	No mention	Durable
Chiu, 2018[1328]	J Neurosurg	WEB	33	13	Multi-country	RO	2013 - 2015	8	NR	Yes	No mention	No mention	No mention
Raymond, 2014[1329]	AJNR Am J Neuroradiol	Coil vs Coil	222 vs 222	25	Multi-country	RCT	2007 - 2014	1	Yes	NR	Safe - Safe	Effective – Effective	No mention – No mention
Raz, 2015[1330]	AJNR Am J Neuroradiol	PED	28	1	USA	RO	2008 - 2013	15.1	NR	NR	Safe	Effective	No mention
Rho, 2013[1331]	Acta Neurochir	SAC	28	1	South Korea	RO	2006 - 2012	32	NR	None	Doubt	Effective	No mention
Rivet, 2007[1332]	AJNR Am J Neuroradiol	Coil vs Coil	70 vs 70	1	USA	RO	2002 - 2004		NR	NR	No mention – No mention	Effective – Effective	No mention – No mention
Rossitti, 2007[1333]	Acta Radiol	Coil	104	1	Sweden	RO	2004 - 2006	5.9	None	None	Safe	Effective	No mention

Roy, 2001[1334]	Stroke	Coil	116	1	Canada	PO	1992 - 1999	32.1	NR	NR	Safe	No mention	No mention
Saatci, 2012[1335]	AJNR Am J Neuroradiol	PED	191	1	Turkey	RO	2008 - 2011	**12**	Yes	NR	Safe	Effective	Durable
Safavi-Abbasi, 2016[1336]	J Neurosurg	Clip	57	1	USA	RO	2004 - 2014	**60.9**	NR	None	Safe	Effective	Durable
Safavi-Abbasi, 2016[1337]	World Neurosurg	Clip	30	1	USA	RO	2004 - 2014	**42.3**	NR	Yes	Safe	No mention	No mention
Schaller, 2008[1338]	J Stroke Cerebrovasc Dis	Bypass	408	20	Multi-Country	RO	1985 - 2002	**39**	NR	NR	Doubt	Doubt	No mention
Seifert, 2008[1339]	Dtsch Arztebl Int	Clip vs Coil	126 vs 74	1	Germany	RO	1999 - 2007	**6**	NR	None	Doubt – Doubt	No mention - No mention	No mention - No mention
Sekhar, 2013[1340]	Neurosurgery	Clip vs Coil	37 vs 63	1	USA	RO	2005 - 2012	**12**	NR	Yes	Doubt – Doubt	Doubt – Doubt	Durable - Durable
Sejkorová, 2017[1341]	Acta Neurochir (Wien)	Coil vs Clip	43 vs 38	3	Multi-Country	RO	2000 - 2015	**36**	Yes	None	No mention – No mention	Effective – Effective	No mention – No mention
Zweifel, 2015[1342]	Acta Neurochir	Endo vs Clip	107 vs 102	1	Canada	PO	2007 - 2012	**6**	Yes	None	Safe - Safe	Effective – Effective	No mention – No mention
Yahia, 2007[1343]	Neurocrit Care	SAC	29	1	USA	PO	2004 - 2004		NR	NR	Safe	No mention	No mention
Yadla, 2011[1344]	Neurosurgery	Coil vs Clip	147 vs 23	1	USA	RO	1997 - 2009		NR	Yes	Safe - Safe	Effective – Effective	No mention – No mention
Van Rooij, 2012[1345]	Am J Neuroradiol 2012	Endo	85	1	The Netherlands	RO	1995 - 2010	**42**	NR	NR	Safe	Effective	No mention
Taqi, 2018[1346]	Interv Neurol	BAC	81	8	Multi-Country	PO	2013 - 2015		Yes	Yes	Safe	Effective	No mention
Taschner, 2016[1347]	Neuroradiology	Coil vs Coil	243 vs 241	22	Multi-Country	RCT	2009 - 2015		Yes	Yes	Safe - Safe	Effective – Effective	No mention – No mention
Tjahjadi, 2017[1348]	Interdiscip Neurosurg	Endo	109	1	South Korea	RO	2003 - 2014	**43.5**	NR	NR	Doubt	Effective	No mention
Song, 2015[1349]	Acta neurosurgery	**Endo vs Clip**	**566 vs 557**	1	South Korea	RO	2008 - 2014	**6**	None	None	Safe - Safe	No mention – No mention	No mention – No mention
Szeleny, 2011[1350]	Clinical neurology and neurosurgery	Clip	87	1	Germany	RO	2001 - 2006	**6**	None	None	Safe	No mention	No mention
Taha, 2006[1351]	Surgical neurology	Coil vs Clip	71 **vs 49**	1	Japan	RO	2001 - 2005	**3**	None	None	Safe – No mention	No mention – No mention	No mention – No mention
Chalouhi, 2012[1352]	Neurosurg	**Coil vs SAC**	147 vs 88	**1**	USA	**RO**	**2004 vs 2011**		None	Yes	Safe - Safe	Effective – Effective	No mention – No mention

© 2023 Volovici V et al. *JAMA Network Open.*

Kulcsár, 2016[1353]	J Neurointervent Surg	**Coil**	**92**	**3**	**Multi-country**	**RO**	**2011-2014**	**7**	**NR**	**Yes**	**Safe**	**Effective**	**Doubt**
Sluzewski, 2003[1354]	AJNR Am J Neuroradiol	**Coil**	**29**	**1**	**The Netherlands**	**RO**	**1994 – 2000**	**50**	**NR**	**NS**	**No mention**	**Not effective**	**No mention**
Iskander, 2011[1355]	Intervent Neuroradiol	**Endovascular**	**107**	**1**	**Denmark**	**RO**	**1999 – 2009**	**9**	**NR**	**NS**	**No mention**	**Doubt**	**Doubt**
Van Rooij, 2006[1356]	Neurosurgery	**Clip + bypass**	**33**	**1**	**The Netherlands**	**RO**	**1994 – 2008**		**NR**	**Yes**	**Safe**	**No mention**	**No mention**

© 2023 Volovici V et al. *JAMA Network Open.*

eReferences.

1. Aguilar-Salinas P, Brasiliense LB, Santos R, Cortez G, Gonsales D, Aghaebrahim A, et al. Safety and Efficacy of Stent-assisted Coiling in the Treatment of Unruptured Wide-necked Intracranial Aneurysms: A Single-center Experience. Cureus. 2019;11(6):e4847.
2. Pierot L, Gubucz I, Buhk JH, Holtmannspötter M, Herbreteau D, Stockx L, et al. Safety and efficacy of aneurysm treatment with the WEB: Results of the WEBCAST 2 study. Am J Neuroradiol. 2017;38(6):1151-555.
3. Pierot L, Costalat V, Moret J, Szikora I, Klisch J, Herbreteau D, et al. Safety and efficacy of aneurysm treatment with WEB: Results of the WEBCAST study. J Neurosurg. 2016;124(5):1250-6.
4. Chalouhi N, Zanaty M, Whiting A, Yang S, Tjoumakaris S, Hasan D, et al. Safety and efficacy of the Pipeline Embolization Device in 100 small intracranial aneurysms. J Neurosurg. 2015;122(6):1498-502.
5. Briganti F, Leone G, Ugga L, Marseglia M, Solari D, Caranci F, et al. Safety and efficacy of flow re-direction endoluminal device (FRED) in the treatment of cerebral aneurysms: a single center experience. Acta Neurochir. 2016;158(9):1745-55.
6. Kraus B, Goertz L, Turowski B, Borggrefe J, Schlamann M, Dorn F, et al. Safety and efficacy of the Derivo Embolization Device for the treatment of unruptured intracranial aneurysms: A multicentric study. J Neurointervent Surg. 2019;11(1):68-73.
7. Xue G, Tang H, Liu P, Zuo Q, Yang P, Zhou Y, et al. Safety and Long-term Efficacy of Stent-assisted Coiling for the Treatment of Complex Posterior Cerebral Artery Aneurysms. Clin Neurorad. 2019.
8. Dellaretti M, Ronconi D, Batista DM, Ferreira de Souza R, Almeida CERD, Fontoura RR, et al. Safety and Efficacy of Surgical Treatment of Intracranial Aneurysms: The Experience of a Single Brazilian Center. World Neurosurg. 2018;117:e580-e7.
9. Lawson A, Molyneux A, Sellar R, Lamin S, Thomas A, Gholkar A, et al. Safety results from the treatment of 109 cerebral aneurysms using the Woven EndoBridge technique: Preliminary results in the United Kingdom. J Neurosurg. 2018;128(1):144-53.
10. Griessenauer CJ, Ogilvy CS, Foreman PM, Chua MH, Harrigan MR, He L, et al. Pipeline embolization device for small intracranial aneurysms: Evaluation of safety and efficacy in a multicenter cohort. Clin Neurosurgery. 2017;80(4):579-87.
11. Pierot L, Spelle L, Berge J, Januel AC, Herbreteau D, Aggour M, et al. SAFE study (Safety and efficacy Analysis of FRED Embolic device in aneurysm treatment): 1-year clinical and anatomical results. J Neurointervent Surg. 2019;11(2):184-9.
12. Phillips TJ, Wenderoth JD, Phatouros CC, Rice H, Singh TP, Devilliers L, et al. Safety of the pipeline embolization device in treatment of posterior circulation aneurysms. Am J Neuroradiol. 2012;33(7):1225-31.
13. Martínez-Galdámez M, Biondi A, Kalousek V, Pereira VM, Ianucci G, Gentric JC, et al. Periprocedural safety and technical outcomes of the new Silk Vista Baby flow diverter for the treatment of intracranial aneurysms: Results from a multicenter experience. J Neurointervent Surg. 2019;11(7):723-7.
14. Deshaies EM, Adamo MA, Boulos AS. A prospective single-center analysis of the safety and efficacy of the HydroCoil embolization system for the treatment of intracranial aneurysms. J Neurosurg. 2007;106(2):226-33.
15. Taschner CA, Leclerc X, Gauvrit JY, Kerkeni A, El-Mahdy M, Lejeune JP, et al. Safety of endovascular treatment of intracranial aneurysms with a new, complex shaped Guglielmi detachable coil. Neuroradiology. 2007;49(9):761-6.
16. Möhlenbruch MA, Herweh C, Jestaedt L, Stampfl S, Schönenberger S, Ringleb PA, et al. The FRED flow-diverter stent for intracranial aneurysms: Clinical study to assess safety and efficacy. Am J Neuroradiol. 2015;36(6):1155-61.

17. Clarençon F, Di Maria F, Gabrieli J, Shotar E, Zeghal C, Nouet A, et al. Flow Diverter Stents for the Treatment of Anterior Cerebral Artery Aneurysms: Safety and Effectiveness. Clin Neurorad. 2017;27(1):51-6.
18. Guerreiro-Simoes R, Soize S, Gawlitza M, Manceau PF, Pierot L. Intracranial aneurysms treatment with Barricade coils: Safety and 1-year efficacy in a prospective, single-center series. J Neuroradiol. 2019;46(5):331-5.
19. Fiorella D, Molyneux A, Coon A, Szikora I, Saatci I, Baltacioglu F, et al. Demographic, procedural and 30-day safety results from the WEB Intra-saccular Therapy Study (WEB-IT). J Neurointervent Surg. 2017;9(12):1191-6.
20. Luecking H, Engelhorn T, Lang S, Goelitz P, Kloska S, Roessler K, et al. FRED Flow diverter: A study on safety and efficacy in a consecutive group of 50 patients. Am J Neuroradiol. 2017;38(3):596-602.
21. Fargen KM, Blackburn S, Deshaies EM, Carpenter JS, Jabbour P, Mack WJ, et al. Final results of the multicenter, prospective Axium MicroFX for Endovascular Repair of IntraCranial Aneurysm Study (AMERICA). J Neurointervent Surg. 2015;7(1):40-3.
22. Ng P, Khangure MS, Phatouros CC, Bynevelt M, ApSimon H, McAuliffe W. Endovascular treatment of intracranial aneurysms with Guglielmi detachable coils: Analysis of midterm angiographic and clinical outcomes. Stroke. 2002;33(1):210-7.
23. Gizewski ER, Göricke S, Wolf A, Schoch B, Stolke D, Forsting M, et al. Endovascular treatment of intracranial aneurysms in patients 65 years or older: Clinical outcomes. Am J Neuroradiol. 2008;29(8):1575-80.
24. Mukonoweshuro W, Laitt RD, Hughes DG. Endovascular treatment of PICA aneurysms. Neuroradiology. 2003;45(3):188-92.
25. Kim CH, Cho YD, Jung SC, Ahn JH, Kang HS, Kim JE, et al. Endovascular treatment for superior cerebellar artery aneurysms: Morphological features, technique, and outcome. Neuroradiology. 2014;56(8):647-54.
26. Gonzalez NR, Dusick JR, Duckwiler G, Tateshima S, Jahan R, Martin NA, et al. Endovascular coiling of intracranial aneurysms in elderly patients: Report of 205 treated aneurysms. Neurosurgery. 2010;66(4):714-20.
27. Oishi H, Yamamoto M, Nonaka S, Arai H. Endovascular therapy of internal carotid artery bifurcation aneurysms. J Neurointerv Surg. 2013;5(5):400-4.
28. Zhou Y, Yang PF, Fang YB, Xu Y, Hong B, Zhao WY, et al. A novel flow-diverting device (tubridge) for the treatment of 28 large or giant intracranial aneurysms: A single-center experience. Am J Neuroradiol. 2014;35(12):2326-33.
29. Guzzardi G, Stanca C, Cerini P, Del Sette B, Divenuto I, Malatesta E, et al. Long-term follow-up in the endovascular treatment of intracranial aneurysms with flow-diverter stents: update of a single-centre experience. Radiol Med. 2018;123(6):449-55.
30. Gobble RM, Hoang H, Jafar J, Adelman M. Extracranial-intracranial bypass: Resurrection of a nearly extinct operation. J Vasc Surg. 2012;56(5):1303-7.
31. Van Rooij SBT, Peluso JP, Sluzewski M, Kortman HG, Van Rooij WJ. The new low-profile WEB 17 system for treatment of intracranial aneurysms: First clinical experiences. Am J Neuroradiol. 2018;39(5):859-63.
32. Sun Y, Li Y, Li AM. Endovascular treatment of paraclinoid aneurysms. Intervent Neuroradiol. 2011;17(4):425-30.
33. Gentric JC, Biondi A, Piotin M, Mounayer C, Lobotesis K, Bonafé A, et al. Safety and efficacy of neuroform for treatment of intracranial aneurysms: A prospective, consecutive, french multicentric study. Am J Neuroradiol. 2013;34(6):1203-8.

© 2023 Volovici V et al. *JAMA Network Open.*

34. Ozpeynirci Y, Braun M, Pala A, Schick M, Schmitz B. WEB-only treatment of ruptured and unruptured intracranial aneurysms: a retrospective analysis of 47 aneurysms. Acta Neurochir. 2019;161(8):1507-13.
35. Pujari A, Howard BM, Skukalek SL, Cherian J, Al-Bayati A, Tong F, et al. Hemodynamic Fate of the Precommunicating Anterior Cerebral Artery Is Predicted by Vessel Dominance After Pipeline Embolization Device Deployment Across the Internal Carotid Artery Terminus. World Neurosurg. 2019;128:e688-e93.
36. Hanel RA, Kallmes DF, Lopes DK, Nelson PK, Siddiqui A, Jabbour P, et al. Prospective study on embolization of intracranial aneurysms with the pipeline device: the PREMIER study 1 year results. J Neurointerv Surg. 2019.
37. Teramoto S, Oishi H, Arai H. Comparative Analysis of Long-Term Effect of Stent-Assisted Coiling in Unruptured Sidewall-Type and Terminal-Type Aneurysms. World Neurosurg. 2019;126:e753-e7.
38. Enriquez-Marulanda A, Ravindran K, Salem MM, Ascanio LC, Kan P, Srinivasan VM, et al. Evaluation of Radiological Features of the Posterior Communicating Artery and Their Impact on Efficacy of Saccular Aneurysm Treatment with the Pipeline Embolization Device: A Case Series Study. World Neurosurg. 2019;125:e998-e1007.
39. Pujari A, Howard BM, Madaelil TP, Skukalek SL, Roy AK, Dion JE, et al. Pipeline embolization device treatment of internal carotid artery terminus aneurysms. J Neurointervent Surg. 2019;11(5):485-8.
40. Haffaf I, Clarençon F, Shotar E, Rolla-Bigliani C, Vande Perre S, Mathon B, et al. Medina embolization device for the treatment of intracranial aneurysms: 18 months' angiographic results. J Neurointervent Surg. 2019;11(5):516-22.
41. Wallace AN, Madaelil TP, Kamran M, Miller TR, Delgado Almandoz JE, Grossberg JA, et al. Pipeline Embolization of Vertebrobasilar Aneurysms—A Multicenter Case Series. World Neurosurg. 2019;124:e460-e9.
42. Balaji A, Rajagopal N, Yamada Y, Teranishi T, Kawase T, Kato Y. A Retrospective Study in Microsurgical Procedures of Large and Giant Intracranial Aneurysms: An Outcome Analysis. World Neurosurg X. 2019;2.
43. Mooney MA, Simon ED, Brigeman S, Nakaji P, Zabramski JM, Lawton MT, et al. Long-term results of middle cerebral artery aneurysm clipping in the Barrow Ruptured Aneurysm Trial. J Neurosurg. 2019;130(3):895-901.
44. Roy AK, Philipp LR, Howard BM, Cawley CM, Grossberg JA, Barrow DL. Microsurgical Treatment of Cerebral Aneurysms After Previous Endovascular Therapy: Single-Center Series and Systematic Review. World Neurosurg. 2019;123:e103-e15.
45. Ni W, Yang H, Xu B, Xu F, Jiang H, Lei Y, et al. Proximal Middle Cerebral Artery Aneurysms: Microsurgical Management and Therapeutic Results. World Neurosurg. 2019;122:e907-e16.
46. Zidan M, Gawlitza M, Metaxas G, Foussier C, Soize S, Pierot L. Endovascular treatment of intracranial aneurysms with Barricade coils: Feasibility, procedural safety, and immediate postoperative anatomical results. J Neuroradiol. 2016;43(5):353-7.
47. Zhu YQ, Li MH, Lin F, Song DL, Tan HQ, Gu BX, et al. Frequency and predictors of endoleaks and long-term patency after covered stent placement for the treatment of intracranial aneurysms: A prospective, non-randomised multicentre experience. Eur Radiol. 2013;23(1):287-97.
48. Zang Y, Wang C, Zhang Y, Ding X, Wang Y, Wang X, et al. Long-term follow-up study of 35 cases after endovascular treatment for vertebrobasilar dissecting aneurysms. Clin Neurol Neurosurg. 2015;137:121-31.
49. Wallace AN, Kayan Y, Delgado Almandoz JE, Fease JL, Milner AA, Scholz JM. Endovascular Treatment of Wide-Necked Intracranial Aneurysms with the Scepter XC Balloon Catheter, with Low-Profile Visualized Intraluminal Support (LVIS) Jr. Deployment as a "Bailout" Technique. World Neurosurg. 2019;121:e798-e807.

© 2023 Volovici V et al. *JAMA Network Open.*

50. Aguilar-Pérez M, Kurre W, Fischer S, Bäzner H, Henkes H. Coil occlusion of wide-neck bifurcation aneurysms assisted by a novel intra- to extra-aneurysmatic neck-bridging device (pCONus): Initial experience. Am J Neuroradiol. 2014;35(5):965-71.
51. Zanaty M, Chalouhi N, Barros G, Schwartz EW, Saigh MP, Starke RM, et al. Flow-diversion for ophthalmic segment aneurysms. Neurosurgery. 2015;76(3):286-9; discussion 9-90.
52. Zammar SG, Buell TJ, Chen CJ, Crowley RW, Ding D, Griessenauer CJ, et al. Outcomes After Off-Label Use of the Pipeline Embolization Device for Intracranial Aneurysms: A Multicenter Cohort Study. World Neurosurg. 2018;115:e200-e5.
53. Yue W. Endovascular treatment of unruptured intracranial aneurysms. Intervent Neuroradiol. 2011;17(4):420-4.
54. Ten Brinck MFM, De Vries J, Bartels RHMA, André Grotenhuis J, Boogaarts HD. Neuroform atlas stent-assisted coiling: Preliminary results. Neurosurgery. 2019;84(1):179-89.
55. Yan B, du Mesnil de Rochement R, Raabe A, Zanella F, Berkefeld J. Single-center experience with TruFill platinum coils for the embolization of cerebral aneurysms. Neuroradiology. 2006;48(4):264-8.
56. Xu F, Xu B, Huang L, Xiong J, Gu Y, Lawton MT. Surgical Treatment of Large or Giant Fusiform Middle Cerebral Artery Aneurysms: A Case Series. World Neurosurg. 2018;115:e252-e62.
57. Sheen JJ, Park W, Kwun BD, Park JC, Ahn JS. Microsurgical treatment strategy for large and giant aneurysms of the internal carotid artery. Clin Neurol Neurosurg. 2019;177:54-62.
58. Brasiliense LBC, Aguilar-Salinas P, Lopes DK, Nogueira D, Desousa K, Nelson PK, et al. Multicenter Study of Pipeline Flex for Intracranial Aneurysms. Clin Neurosurgery. 2019;84(6):E402-E9.
59. Xiaoxi Z, Jing C, Qinghai H, Jianmin L, Bo H, Dongwei D. Microcatheter Looping Technique Facilitates the Embolization of Complex Intracranial Aneurysms with an Acute Angle Branch Incorporated into the Sac. World Neurosurg. 2017;100:56-61.
60. Williams A, Millar J, Ditchfield A, Vundavalli S, Barker S. Use of Hydrocoil in small aneurysms: procedural safety, treatment efficacy and factors predicting complete occlusion. Interv neuroradiol. 2014;20(1):37-44.
61. Weber W, Siekmann R, Kis B, Kuehne D. Treatment and follow-up of 22 unruptured wide-necked intracranial aneurysms of the internal carotid artery with Onyx HD 500. Am J Neuroradiol. 2005;26(8):1909-15.
62. Wanke I, Doerfler A, Goericke S, Gizewski ER, Sandalcioglu E, Moemken S, et al. Treatment of wide-necked intracranial aneurysms with a self-expanding stent: Mid-term results. Zentralbl Neurochir. 2005;66(4):163-9.
63. Wang CC, Fang YB, Zhang P, Zhu X, Hong B, Xu Y, et al. Reconstructive endovascular treatment of vertebral artery dissecting aneurysms with the Low-profile Visualized Intraluminal Support (LVIS) device. PLoS ONE. 2017;12(6).
64. Wang WX, Xu BN, Wang FY, Wu C, Sun ZH. Microsurgical management of posterior cerebral artery aneurysms: A report of thirty cases in modern era. Br J Neurosurg. 2015;29(3):406-12.
65. Wang H, Luo L, Ye Z, Li W, Chen C, Ba Y, et al. Clipping of anterior communicating artery aneurysms in the early post-rupture stage via transorbital keyhole approach - Chinese neurosurgical experience. Br J Neurosurg. 2015;29(5):644-9.
66. Wakhloo AK, Lylyk P, De Vries J, Taschner C, Lundquist J, Biondi A, et al. Surpass flow diverter in the treatment of intracranial aneurysms: A prospective multicenter study. Am J Neuroradiol. 2015;36(1):98-107.
67. Wakhloo AK, Mandell J, Gounis MJ, Brooks C, Linfante I, Winer J, et al. Stent-assisted reconstructive endovascular repair of cranial fusiform atherosclerotic and dissecting aneurysms: long-term clinical and angiographic follow-up. Stroke. 2008;39(12):3288-96.

© 2023 Volovici V et al. *JAMA Network Open.*

68. Wakhloo AK, Gounis MJ, Sandhu JS, Akkawi N, Schenck AE, Linfante I. Complex-shaped platinum coils for brain aneurysms: Higher packing density, improved biomechanical stability, and midterm angiographic outcome. Am J Neuroradiol. 2007;28(7):1395-400.
69. Wajnberg E, de Souza JM, Marchiori E, Gasparetto EL. Single-center experience with the Neuroform stent for endovascular treatment of wide-necked intracranial aneurysms. Surg Neurol. 2009;72(6):612-9.
70. Volker M, Anastasios M, Jan B, Nuran A, Thomas L, Franziska D, et al. Treatment of Intracranial Aneurysms with the Pipeline Embolization Device Only: a Single Center Experience. Neurointervention. 2018;13(1):32-40.
71. Velasco González A, Stracke P, Nordmeyer H, Heddier M, Saleme S, Sauerland C, et al. Low rates of recanalization for wide-necked aneurysms treated with stenting after balloon-assisted coiling: combination of techniques delivers stable and improved results during follow-up. Neuroradiology. 2018;60(11):1223-30.
72. Van Rooij WJ, Sluzewski M. Coiling of very large and giant basilar tip aneurysms: Midterm clinical and angiographic results. Am J Neuroradiol. 2007;28(7):1405-8.
73. Van Rooij WJ, Sluzewski M. Procedural morbidity and mortality of elective coil treatment of unruptured intracranial aneurysms. Am J Neuroradiol. 2006;27(8):1678-80.
74. Van Rooij WJ, De Gast A, Sluzewski M, Nijssen PC, Beute GN. Coiling of truly incidental intracranial aneurysms. Am J Neuroradiol. 2006;27(2):293-6.
75. Tureli D, Sabet S, Senol S, Andac N, Donmez H, Geyik S, et al. Stent-assisted coil embolization of challenging intracranial aneurysms: initial and mid-term results with low-profile ACCLINO devices. Acta Neurochir. 2016;158(8):1545-53.
76. Thornton J, Aletich VA, Debrun GM, Alazzaz A, Misra M, Charbel F, et al. Endovascular treatment of paraclinoid aneurysms. Surg Neurol. 2000;54(4):288-99.
77. Hagen F, Maurer CJ, Berlis A. Endovascular treatment of unruptured MCA bifurcation aneurysms regardless of aneurysm morphology: Short- and long-term follow-up. Am J Neuroradiol. 2019;40(3):503-9.
78. Goertz L, Dorn F, Siebert E, Herzberg M, Borggrefe J, Schlamann M, et al. Safety and efficacy of the Neuroform Atlas for stent-assisted coiling of intracranial aneurysms: A multicenter experience. J Clin Neurosci. 2019.
79. Wang L, Lu S, Qian H, Shi X. Internal Maxillary Artery Bypass with Radial Artery Graft Treatment of Giant Intracranial Aneurysms. World Neurosurg. 2017;105:568-84.
80. Van Dijk JMC, Groen RJM, Ter Laan M, Jeltema JR, Mooij JJA, Metzemaekers JDM. Surgical clipping as the preferred treatment for aneurysms of the middle cerebral artery. Acta Neurochir. 2011;153(11):2111-5.
81. Thines L, Bourgeois P, Lejeune JP. Surgery for unruptured intracranial aneurysms in the ISAT and ISUIA Era. Can J Neurol Sci. 2012;39(2):174-9.
82. Taschner CA, Vedantham S, de Vries J, Biondi A, Boogaarts J, Sakai N, et al. Surpass Flow Diverter for Treatment of Posterior Circulation Aneurysms. AJNR Am J Neuroradiol. 2017;38(3):582-9.
83. Tähtinen OI, Manninen HI, Vanninen RL, Rautio R, Haapanen A, Seppänen J, et al. Stent-assisted embolization of recurrent or residual intracranial aneurysms. Neuroradiology. 2013;55(10):1221-31.
84. De Leacy RA, Fargen KM, Mascitelli JR, Fifi J, Turkheimer L, Zhang X, et al. Wide-neck bifurcation aneurysms of the middle cerebral artery and basilar apex treated by endovascular techniques: A multicentre, core lab adjudicated study evaluating safety and durability of occlusion (BRANCH). J Neurointervent Surg. 2019;11(1):31-6.
85. Bhogal P, Chudyk J, Bleise C, Lylyk I, Perez N, Henkes H, et al. The Use of Flow Diversion in Vessels ≤2.5 mm in Diameter–A Single-Center Experience. World Neurosurg. 2018;118:e575-e83.

© 2023 Volovici V et al. *JAMA Network Open.*

86. Schob S, Hoffmann KT, Richter C, Bhogal P, Köhlert K, Planitzer U, et al. Flow diversion beyond the circle of Willis: Endovascular aneurysm treatment in peripheral cerebral arteries employing a novel low-profile flow diverting stent. J Neurointervent Surg. 2019.
87. Tahtinen OI, Manninen HI, Vanninen RL, Seppanen J, Niskakangas T, Rinne J, et al. The silk flow-diverting stent in the endovascular treatment of complex intracranial aneurysms: technical aspects and midterm results in 24 consecutive patients. Neurosurgery. 2012;70(3):617-23; discussion 23-4.
88. Symon L. Surgical experiences with giant intracranial aneurysms. ACTA NEUROCHIR. 1992;118(1-2):53-8.
89. Suzuki K, Suzuki R, Takigawa T, Shimizu N, Matsumoto Y, Fujii Y, et al. A single center experience with coil embolization for cerebral aneurysms greater than 10 mm in the internal carotid artery. Neurol Med -Chir. 2017;57(5):231-7.
90. Suh SJ, Kim SC, Kang DG, Ryu KY, Lee HG, Cho JH. Clinical and angiographic results after treatment with combined clipping and wrapping technique for intracranial aneurysm. J Korean Neurosurg Soc. 2008;44(4):190-5.
91. Sturiale CL, Brinjikji W, Murad MH, Cloft HJ, Kallmes DF, Lanzino G. Endovascular treatment of distal anterior cerebral artery aneurysms: Single-center experience and a systematic review. Am J Neuroradiol. 2013;34(12):2317-20.
92. Starke RM, Chalouhi N, Ali MS, Penn DL, Tjoumakaris SI, Jabbour PM, et al. Endovascular treatment of very small ruptured intracranial aneurysms: Complications, occlusion rates and prediction of outcome. J Neurointervent Surg. 2013;5(SUPPL.3):iii66-iii71.
93. Souza JRF, Otoch M, Ribeiro SP, Ramos Jr F, De Almeida JPC, De Albuquerque LAF, et al. Endovascular treatment of cerebral aneurysms: A retrospective study of 163 embolized aneurysms. Arq Neuro-Psiquiatr. 2007;65(2 B):411-5.
94. Sorteberg W, Slettebø H, Eide PK, Stubhaug A, Sorteberg A. Surgical treatment of aneurysmal subarachnoid haemorrhage in the presence of 24-h endovascular availability: Management and results. Br J Neurosurg. 2008;22(1):53-62.
95. Mine B, Goutte A, Brisbois D, Lubicz B. Endovascular treatment of intracranial aneurysms with the Woven EndoBridge device: Mid term and long term results. J Neurointervent Surg. 2018;10(2):127-32.
96. Sluzewski M, Menovsky T, Van Rooij WJ, Wijnalda D. Coiling of very large or giant cerebral aneurysms: Long-term clinical and serial angiographic results. Am J Neuroradiol. 2003;24(2):257-62.
97. Simon S, Archer K, Mericle R. Multicenter registry of liquid embolic treatment of cerebral aneurysms. World Neurosurg. 2014;82(6):E731-E8.
98. Shimizu K, Imamura H, Mineharu Y, Adachi H, Sakai C, Tani S, et al. Endovascular parent-artery occlusion of large or giant unruptured internal carotid artery aneurysms. A long-term single-center experience. J Clin Neurosci. 2017;37:73-8.
99. Shimizu K, Imamura H, Mineharu Y, Adachi H, Sakai C, Sakai N. Endovascular treatment of unruptured Paraclinoid aneurysms: Single-center experience with 400 cases and literature review. Am J Neuroradiol. 2016;37(4):679-85.
100. Sato K, Endo H, Fujimura M, Endo T, Matsumoto Y, Shimizu H, et al. Endovascular Treatments in Combination with Extracranial-Intracranial Bypass for Complex Intracranial Aneurysms. World Neurosurg. 2018;113:e747-e60.
101. Santillan A, Greenberg E, Patsalides A, Salvaggio K, Riina HA, Pierre Gobin Y. Long-term clinical and angiographic results of neuroform stent-assisted coil embolization in wide-necked intracranial aneurysms. Neurosurgery. 2012;70(5):1232-7.
102. Rodríguez-Hernández A, Walcott BP, Birk H, Lawton MT. The superior cerebellar artery aneurysm: A posterior circulation aneurysm with favorable microsurgical outcomes. Clin Neurosurgery. 2017;80(6):908-16.

© 2023 Volovici V et al. *JAMA Network Open.*

103. Raymond J, Roy D. Safety and efficacy of endovascular treatment of acutely ruptured aneurysms. NEUROSURGERY. 1997;41(6):1235-46.
104. Ravindran K, Enriquez-Marulanda A, Kan PTM, Renieri L, Limbucci N, Mangiafico S, et al. Use of Flow Diversion for the Treatment of Distal Circulation Aneurysms: A Multicohort Study. World Neurosurg. 2018;118:e825-e33.
105. Bender MT, Jiang B, Campos JK, Lin LM, Beaty N, Vo CD, et al. Single-stage flow diversion with adjunctive coiling for cerebral aneurysm: Outcomes and technical considerations in 72 cases. J Neurointervent Surg. 2018;10(9):843-50.
106. Cohen JE, Gomori JM, Leker RR, Spektor S, Abu El Hassan H, Itshayek E. Stent and flow diverter assisted treatment of acutely ruptured brain aneurysms. J Neurointervent Surg. 2018;10(9):851-8.
107. Raslan AM, Oztaskin M, Thompson EM, Dogan A, Petersen B, Nesbit G, et al. Neuroform stent-assisted embolization of incidental anterior communicating artery aneurysms: Long-term clinical and angiographic follow-up. Neurosurgery. 2011;69(1):27-37.
108. Raco A, Frati A, Santoro A, Vangelista T, Salvati M, Delfini R, et al. Long-term surgical results with aneurysms involving the ophthalmic segment of the carotid artery. J Neurosurg. 2008;108(6):1200-10.
109. Pumar JM, Arias-Rivas S, Rodríguez-Yáñez M, Blanco M, Ageitos M, Vazquez-Herrero F, et al. Using Leo Plus stent as flow diverter and endoluminal remodeling in endovascular treatment of intracranial fusiform aneurysms. J Neurointervent Surg. 2013;5(SUPPL.3):iii22-iii7.
110. Pierot L, Klisch J, Liebig T, Gauvrit JY, Leonardi M, Nuzzi NP, et al. WEB-DL endovascular treatment of wide-neck bifurcation aneurysms: Long-term results in a European series. Am J Neuroradiol. 2015;36(12):2314-9.
111. Pierot L, Klisch J, Cognard C, Szikora I, Mine B, Kadziolka K, et al. Endovascular WEB flow disruption in middle cerebral artery aneurysms: Preliminary feasibility, clinical, and anatomical results in a multicenter study. Neurosurgery. 2013;73(1):27-34.
112. Pierot L, Liebig T, Sychra V, Kadziolka K, Dorn F, Strasilla C, et al. Intrasaccular flow-disruption treatment of intracranial aneurysms: Preliminary results of a multicenter clinical study. Am J Neuroradiol. 2012;33(7):1232-8.
113. Pierot L, Spelle L, Vitry F, Pasco A, Bonneville JF, Barreau X, et al. Immediate anatomic results after the endovascular treatment of unruptured intracranial aneurysms: Analysis of the ATENA series. Am J Neuroradiol. 2010;31(1):140-4.
114. Pierot L, Cognard C, Ricolfi F, Anxionnat R. Immediate anatomic results after the endovascular treatment of ruptured intracranial aneurysms: Analysis in the CLARITY series. Am J Neuroradiol. 2010;31(5):907-11.
115. Pierot L, Bonafé A, Bracard S, Leclerc X. Endovascular treatment of intracranial aneurysms with matrix detachable coils: Immediate posttreatment results from a prospective multicenter registry. Am J Neuroradiol. 2006;27(8):1693-9.
116. Cay F, Peker A, Arat A. Stent-assisted coiling of cerebral aneurysms with the Neuroform Atlas stent. Intervent Neuroradiol. 2018;24(3):263-9.
117. Dodier P, Frischer JM, Wang WT, Auzinger T, Mallouhi A, Serles W, et al. Immediate Flow Disruption as a Prognostic Factor After Flow Diverter Treatment: Long-Term Experience with the Pipeline Embolization Device. World Neurosurg. 2018;113:e568-e78.
118. Liang F, Zhang Y, Guo F, Zhang Y, Yan P, Liang S, et al. Use of Pipeline Embolization Device for Posterior Circulation Aneurysms: Single-Center Experiences with Comparison with Anterior Circulation Aneurysms. World Neurosurg. 2018;112:e683-e90.
119. Ho MJ, Göricke SL, Mummel P, Mönninghoff C, Wrede K, Wanke I. Stent-assisted treatment of ruptured intracranial aneurysms in the acute phase: A single center experience. eNeurologicalSci. 2018;10:31-6.

© 2023 Volovici V et al. *JAMA Network Open.*

120. Zhou J, Wang Y, Wang D, Chen Q, Wang H, Gao L. Endovascular Treatment for Ruptured Aneurysms at Distal Cerebral Arteries. World Neurosurg. 2019;123:e387-e92.
121. Zhou Y, Yang PF, Li Q, Zhao R, Fang YB, Xu Y, et al. Stent placement for complex middle cerebral artery aneurysms. J Stroke Cerebrovasc Dis. 2014;23(6):1447-56.
122. Zhou Y, Yang PF, Fang YB, Xu Y, Hong B, Zhao WY, et al. Endovascular treatment for saccular aneurysms of the proximal (M1) segment of the middle cerebral artery. Acta Neurochir (Wien). 2012;154(10):1835-43.
123. Zheng Y, Song Y, Liu D, Liu Y, Xu Q, Tian Y, et al. Stent-assisted coiling embolization of tiny, wide-necked intracranial aneurysms. Acta Neurochir. 2017;159(1):93-100.
124. Zheng Y, Song Y, Liu Y, Xu Q, Tian Y, Leng B. Stent-Assisted Coiling of 501 Wide-Necked Intracranial Aneurysms: A Single-Center 8-Year Experience. World Neurosurg. 2016;94:285-95.
125. Zhao X, Li Z, Fang X, Liu J, Wu D, Lai N. Treatment of ruptured middle cerebral artery aneurysms by endovascular approach: a single-center experience. Int J Neurosci. 2017;127(5):433-8.
126. Zhang Y, Zhang Y, Guo F, Liang F, Yan P, Liang S, et al. Treatment of Small and Tiny Aneurysms Before and After Flow Diversion Era: A Single Center Experience of 409 Aneurysms. World Neurosurg. 2018;116:e386-e93.
127. Zhang Y, Yang M, Zhang H, Zhang X, Li Y, Jiang C, et al. Stent-Assisted Coiling May Prevent the Recurrence of Very Small Ruptured Intracranial Aneurysms: A Multicenter Study. World Neurosurg. 2017;100:22-9.
128. Zhang Y, Gao G, Chao Y, Chen Y, Yu J, Gu D, et al. Endovascular treatment of irregular and complicated intracranial aneurysms with coils using double microcatheter technique. Exp Ther Med. 2017;13(1):75-8.
129. Zhang YY, Fang YB, Wu YN, Zhang Q, Li Q, Xu Y, et al. Angiographic Characteristics and Endovascular Treatment of Anterior Cerebral Artery A1 Segment Aneurysms. World Neurosurg. 2017;97:551-6.
130. Zhang JZ, Yang PF, Huang QH, Xu Y, Hong B, Zhao WY, et al. Stent-assisted coiling strategies for the treatment of wide-necked basilar artery bifurcation aneurysms. J Clin Neurosci. 2014;21(6):962-7.
131. Zenteno M, Modenesi Freitas JM, Aburto-Murrieta Y, Koppe G, Machado E, Lee Á. Balloon-expandable stenting with and without coiling for wide-neck and complex aneurysms. Surg Neurol. 2006;66(6):603-10.
132. Yu M, Liu F, Jiang S, Nie B. Stent-assisted coiling for the treatment of ruptured micro-intracranial wide-necked aneurysms. Intervent Neuroradiol. 2015;21(1):40-3.
133. Yu SCH, Chan MSY, Boet R, Wong JKT, Lam JMK, Poon WS. Intracranial Aneurysms Treated with Guglielmi Detachable Coils: Midterm Clinical and Radiological Outcome in 97 Consecutive Chinese Patients in Hong Kong. Am J Neuroradiol. 2004;25(2):307-13.
134. Youn SO, Lee JI, Ko JK, Lee TH, Choi CH. Endovascular treatment of wide-necked intracranial aneurysms using balloon-assisted technique with hyperform balloon. J Korean Neurosurg Soc. 2010;48(3):207-12.
135. Yavuz K, Geyik S, Saatci I, Cekirge HS. Endovascular treatment of middle cerebral artery aneurysms with flow modification with the use of the pipeline embolization device. Am J Neuroradiol. 2014;35(3):529-35.
136. Yavuz K, Geyik S, Saatci I, Cekirge HS. WingSpan Stent System in the endovascular treatment of intracranial aneurysms: Clinical experience with midterm follow-up results. J Neurosurg. 2008;109(3):445-53.
137. Yang PF, Liu JM, Huang QH, Zhao WY, Hong B, Xu Y, et al. Preliminary experience and short-term follow-up results of treatment of wide-necked or fusiform cerebral aneurysms with a self-expanding, closed-cell, retractable stent. J Clin Neurosci. 2010;17(7):837-41.

© 2023 Volovici V et al. *JAMA Network Open.*

138. Yakovlev SB, Arustamyan SR, Dorokhov PS, Bocharov AV, Bukharin EY, Arkhangel'skaya YN, et al. [Endovascular treatment of large and giant intracranial aneurysms using flow-diverting stents]. Zh Vopr Neirokhir Im N N Burdenko. 2015;79(4):19-27.
139. Ya P, Xuan JG, Yang YL, Wang SN. Acute ruptured intracranial aneurysm packing with HydroCoil Embolic System: Initial clinical experience. Neuroradiol J. 2007;20(3):327-30.
140. Xu D, Zhang C, Wang T, Wang C, Kallmes DF, Lanzino G, et al. Evaluation of Enterprise Stent-Assisted Coiling and Telescoping Stent Technique as Treatment of Supraclinoid Blister Aneurysms of the Internal Carotid Artery. World Neurosurg. 2018;110:e890-e6.
141. Xu L, Deng X, Wang S, Cao Y, Zhao Y, Zhang D, et al. Giant Intracranial Aneurysms: Surgical Treatment and Analysis of Risk Factors. World Neurosurg. 2017;102:293-300.
142. Xu F, Hong Y, Zheng Y, Xu Q, Leng B. Endovascular treatment of posterior inferior cerebellar artery aneurysms: A 7-year single-center experience. J Neurointervent Surg. 2017;9(1):45-51.
143. Xu X, Zheng Y, Wang D, Cui J, Shang X. Improved Endovascular Coiling of Wide-Neck Intracranial Aneurysms in Elderly Patients by Double-Microcatheter Technique. Cell Biochem Biophys. 2015;71(3):1281-6.
144. Xu N, Wang H, Luo Q. Endovascular treatment of intracranial wide-necked aneurysms with GDCs combined with balloon or stent. Neuroradiol J. 2009;22(1):86-91.
145. Wu P, Ocak PE, Wang D, Ocak U, Xu S, Li Y, et al. Endovascular Treatment of Ruptured Tiny Intracranial Aneurysms with Low-Profile Visualized Intraluminal Support Device. J Stroke Cerebrovasc Dis. 2019;28(2):330-7.
146. Wong GKC, Yu SCH, Poon WS. Clinical and angiographic outcome of intracranial aneurysms treated with Matrix detachable coils in Chinese patients. Surg Neurol. 2007;67(2):122-6.
147. Wolynski J, Mordasini P, Schroth G, Barth A, Seiler RW, Remonda L. Endovascular treatment of anterior circulation cerebral aneurysms by using guglielmi detachable coils: A 10-year single-center experience with special emphasis on the use of three-dimensional GDC. Clin Neurorad. 2007;17(2):98-107.
148. Willinsky RA, Peltz J, Da Costa L, Agid R, Farb RI, TerBrugge KG. Clinical and angiographic follow-up of ruptured intracranial aneurysms treated with endovascular embolization. Am J Neuroradiol. 2009;30(5):1035-40.
149. Weber W, Bendszus M, Kis B, Boulanger T, Solymosi L, Kühne D. A new self-expanding nitinol stent (Enterprise) for the treatment of wide-necked intracranial aneurysms: Initial clinical and angiographic results in 31 aneurysms. Neuroradiology. 2007;49(7):555-61.
150. Wanke I, Doerfler A, Dietrich U, Egelhof T, Schoch B, Stolke D, et al. Endovascular treatment of unruptured intracranial aneurysms. Am J Neuroradiol. 2002;23(5):756-61.
151. Wang C, Wu Y, Feng Z, Wang J, Li Q, Zhao R, et al. Preliminary experience with the use of low profile visualized intraluminal support device in basilar artery for aneurysm treatment. J Neurointervent Surg. 2019;11(4):405-10.
152. Wang J, Vargas J, Spiotta A, Chaudry I, Turner RD, Lena J, et al. Stent-assisted coiling of cerebral aneurysms: A single-center clinical and angiographic analysis. J Neurointervent Surg. 2018;10(7):691-6.
153. Wang CC, Li W, Feng ZZ, Hong B, Xu Y, Liu JM, et al. Preliminary experience with stent-assisted coiling of aneurysms arising from small (<2.5 mm) cerebral vessels using the low-profile visualized intraluminal support device. Am J Neuroradiol. 2017;38(6):1163-8.
154. Wang CC, Wen WL, Feng ZZ, Xu Y, Hong B, Liu JM, et al. Endovascular Treatment of 48 Early Branch Aneurysms of the Middle Cerebral Artery. World Neurosurg. 2016;94:131-6.
155. Wang Y, Li Y, Jiang C, Jiang F, Meng H, Siddiqui AH, et al. Endovascular treatment of paraclinoid aneurysms: 142 aneurysms in one centre. J Neurointervent Surg. 2013;5(6):552-6.
156. Wang Y, Li Y, Jiang C, Wu Z, Jiang F, Meng H, et al. Could the types of paraclinoid aneurysm be used as a criterion in choosing endovascular treatment? Neuro-radiologists' view. Acta Neurochir. 2013;155(11):2019-27.

157. Wan J, Gu W, Zhang X, Geng D, Lu G, Huang L, et al. Endovascular coil embolization of aneurysm neck for the treatment of ruptured intracranial aneurysm with bleb formation. Med Sci Monit. 2014;20:1121-8.
158. Wallace AN, Delgado Almandoz JE, Kayan Y, Fease JL, Scholz JM, Milner AM, et al. Pipeline Treatment of Intracranial Aneurysms Is Safe and Effective in Patients with Cutaneous Metal Allergy. World Neurosurg. 2019;123:e180-e5.
159. Waldenberger P, Petersen J, Chemelli A, Schenk C, Gruber I, Strasak A, et al. Endovascular therapy of distal anterior cerebral artery aneurysms-an effective treatment option. Surg Neurol. 2008;70(4):368-77.
160. Waldau B, Turk IAS, Yashar P, Khaldi A, Turner IRD, Chaudry MI, et al. Perioperative safety of Hydrosoft coils. J Neurointervent Surg. 2012;4(5):375-8.
161. Wakhloo AK, Linfante I, Silva CF, Samaniego A, Dabus G, Etezadi V, et al. Closed-cell stent for coil embolization of intracranial aneurysms: Clinical and angiographic results. Am J Neuroradiol. 2012;33(9):1651-6.
162. Voigt P, Schob S, Jantschke R, Nestler U, Krause M, Weise D, et al. Stent-assisted coiling of ruptured and incidental aneurysms of the intracranial circulation using moderately flow-redirecting, braided leo stents-Initial experience in 39 patients. Front Neurol. 2017;8(NOV).
163. Veznedaroglu E, Koebbe CJ, Siddiqui A, Rosenwasser RH. Initial experience with bioactive cerecyte detachable coils: Impact on reducing recurrence rates. Neurosurgery. 2008;62(4):799-805.
164. Vendrell JF, Costalat V, Brunel H, Riquelme C, Bonafe A. Stent-assisted coiling of complex middle cerebral artery aneurysms: Initial and midterm results. Am J Neuroradiol. 2011;32(2):259-63.
165. Velioglu M, Selcuk H, Kizilkilic O, Basekim C, Kocer N, Islak C. Endovascular Management of Superior Cerebellar Artery Aneurysms: Mid and Long-Term Results. Turk Neurosurg. 2015;25(4):526-31.
166. Velioglu M, Kizilkilic O, Selcuk H, Kocak B, Tureci E, Islak C, et al. Early and midterm results of complex cerebral aneurysms treated with Silk stent. Neuroradiology. 2012;54(12):1355-65.
167. van Rooij SBT, van Rooij WJ, Peluso JP, Sluzewski M. The Woven EndoBridge (WEB) as primary treatment for unruptured intracranial aneurysms. Intervent Neuroradiol. 2018;24(5):475-81.
168. Van Rooij SBT, Van Rooij WJ, Peluso JP, Sluzewski M, Bechan RS, Kortman HG, et al. WEB treatment of ruptured intracranial aneurysms: A single-center cohort of 100 patients. Am J Neuroradiol. 2017;38(12):2282-7.
169. Van Rooij WJ, Peluso JP, Bechan RS, Sluzewski M. WEB treatment of ruptured intracranial aneurysms. Am J Neuroradiol. 2016;37(9):1679-83.
170. Van Rooij WJ, Sluzewski M, Beute GN. Endovascular treatment of posterior cerebral artery aneurysms. Am J Neuroradiol. 2006;27(2):300-5.
171. Van Doormaal TPC, Van Der Zwan A, Verweij BH, Regli L, Tulleken CAF. Giant aneurysm clipping under protection of an excimer laser-assisted non-occlusive anastomosis bypass. Neurosurgery. 2010;66(3):439-47.
172. Ulfert C, Pfaff J, Schönenberger S, Bösel J, Herweh C, Pham M, et al. The pCONus Device in Treatment of Wide-necked Aneurysms: Technical and Midterm Clinical and Angiographic Results. Clin Neurorad. 2018;28(1):47-54.
173. Ulfert C, Pham M, Sonnberger M, Amaya F, Trenkler J, Bendszus M, et al. The Neuroform Atlas stent to assist coil embolization of intracranial aneurysms: A multicentre experience. J Neurointervent Surg. 2018;10(12):1192-6.
174. Tsai JP, Hardman J, Moore NZ, Hussain MS, Bain MD, Rasmussen PA, et al. Early post-Humanitarian Device Exemption experience with the Neuroform Atlas stent. J Neurointervent Surg. 2019.
175. Topcuoglu OM, Akgul E, Daglioglu E, Topcuoglu ED, Peker A, Akmangit I, et al. Flow Diversion in Middle Cerebral Artery Aneurysms: Is It Really an All-Purpose Treatment? World Neurosurg. 2016;87:317-27.
176. Tomatis A, Trevisi G, Boido B, Perez R, Benech CA. Surgical Outcomes and Their Correlation with

Increasing Surgical Experience in a Series of 250 Ruptured or Unruptured Aneurysms Undergoing Microsurgical Clipping. World Neurosurg. 2019.
177. Tjahjadi M, Kivelev J, Serrone JC, Maekawa H, Kerro O, Jahromi BR, et al. Factors determining surgical approaches to basilar bifurcation aneurysms and its surgical outcomes. Neurosurgery. 2016;78(2):181-90.
178. Tevah J, Senf R, Cruz J, Fava M. Endovascular treatment of complex cerebral aneurysms with onyx hd-500® in 38 patients. J Neuroradiol. 2011;38(5):283-90.
179. Teping F, Fischer G, Huelser M, Sippl C, Linsler S, Knosp E, et al. A New Clip Generation for Microsurgical Treatment of Intracranial Aneurysms-The First Case Series. 2019.
180. Teleb MS, Pandya DJ, Castonguay AC, Eckardt G, Sweis R, Lazzaro MA, et al. Safety and predictors of aneurysm retreatment for remnant intracranial aneurysm after initial endovascular embolization. J Neurointervent Surg. 2014;6(7):490-4.
181. Taqi MA, Quadri SA, Puri AS, Fitzsimmons BF, Jin JN, Rai AT, et al. A Prospective Multicenter Trial of the TransForm Occlusion Balloon Catheter: Trial Design and Results. Intervent Neurol. 2018;7(1-2):53-64.
182. Tanweer O, Raz E, Brunswick A, Zumofen D, Shapiro M, Riina HA, et al. Cavernous carotid aneurysms in the era of flow diversion: A need to revisit treatment paradigms. Am J Neuroradiol. 2014;35(12):2334-40.
183. Tan HQ, Li MH, Zhang PL, Li YD, Wang JB, Zhu YQ, et al. Reconstructive endovascular treatment of intracranial aneurysms with the Willis covered stent: Medium-term clinical and angiographic follow-up - Clinical article. J Neurosurg. 2011;114(4):1014-20.
184. Takayasu M, Nagatani T, Noda A, Shibuya M, Yoshida J. Clinical safety and performance of Sugita titanium aneurysm clips. Acta Neurochir. 2000;142(2):159-63.
185. Szikora I, Marosfoi M, Salomváry B, Berentei Z, Gubucz I. Resolution of mass effect and compression symptoms following endoluminal flow diversion for the treatment of intracranial aneurysms. Am J Neuroradiol. 2013;34(5):935-9.
186. Suh SH, Kim BM, Chung TS, Kim DI, Kim DJ, Hong CK, et al. Reconstructive endovascular treatment of intracranial fusiform aneurysms: A 1-stage procedure with stent and balloon. Am J Neuroradiol. 2010;31(1):155-60.
187. Stiefel MF, Park MS, McDougall CG, Albuquerque FC. Endovascular treatment of unruptured intracranial aneurysms in the elderly: Analysis of procedure related complications. J Neurointervent Surg. 2010;2(1):11-5.
188. Stetler WR, Wilson TJ, Al-Holou WN, Chaudhary N, Gemmete JJ, Thompson BG, et al. Conventional endovascular treatment of small intracranial aneurysms is not associated with additional risks compared with treatment of larger aneurysms. J Neurointervent Surg. 2015;7(4):262-5.
189. Stapleton CJ, Walcott BP, Fusco MR, Butler WE, Thomas AJ, Ogilvy CS. Surgical management of ruptured middle cerebral artery aneurysms with large intraparenchymal or sylvian fissure hematomas. Neurosurgery. 2015;76(3):258-64.
190. Standhardt H, Boecher-Schwarz H, Gruber A, Benesch T, Knosp E, Bavinzski G. Endovascular treatment of unruptured intracranial aneurysms with Guglielmi detachable coils: Short- and long-term results of a single-centre series. Stroke. 2008;39(3):899-904.
191. Spiotta AM, Chaudry MI, Turner RD, Turk AS, Derdeyn CP, Mocco J, et al. An update on the adjunctive neurovascular support of wide-neck aneurysm embolization and reconstruction trial: 1-year safety and angiographic results. Am J Neuroradiol. 2018;39(5):848-51.
192. Spiotta AM, Derdeyn CP, Tateshima S, Mocco J, Crowley RW, Liu KC, et al. Results of the ANSWER Trial Using the PulseRider for the Treatment of Broad-Necked, Bifurcation Aneurysms. Neurosurgery. 2017;81(1):56-65.

© 2023 Volovici V et al. *JAMA Network Open.*

193. Spiotta AM, Fargen KM, Lena J, Chaudry I, Turner RD, Turk AS, et al. Initial Technical Experience with the SMART Coil for the Embolization of Intracranial Aneurysms. World Neurosurg. 2017;97:80-5.
194. Sonobe M, Nakai Y, Sugita K, Kato N, Okamoto S. Embolization of ruptured aneurysms in the acute stage: experience at a single institute. Surg Neurol. 2008;69(5):478-82.
195. Solander S, Ulhoa A, Viñuela F, Duckwiler GR, Gobin YP, Martin NA, et al. Endovascular treatment of multiple intracranial aneurysms by using Guglielmi detachable coils. J Neurosurg. 1999;90(5):857-64.
196. Sokolowski JD, Ilyas A, Buell TJ, Taylor DG, Chen CJ, Ding D, et al. SMART coils for intracranial aneurysm embolization: Follow-up outcomes. J Clin Neurosci. 2019;59:93-7.
197. Soeda A, Sakai N, Sakai H, Iihara K, Nagata I. Endovascular treatment of asymptomatic cerebral aneurysms: Anatomic and technical factors related to ischemic events and coil stabilization. Neurol Med - Chir. 2004;44(9):456-65.
198. Sirakov S, Sirakov A, Bhogal P, Penkov M, Minkin K, Ninov K, et al. The p64 Flow Diverter—Mid-term and Long-term Results from a Single Center. Clin Neurorad. 2019.
199. Sirakov S, Sirakov A, Hristov H, Minkin K, Penkov M, Karakostov V. Early experience with a temporary bridging device (Comaneci) in the endovascular treatment of ruptured wide neck aneurysms. J Neurointervent Surg. 2018;10(10):978-82.
200. Sheehan MJ, Dunne R, Thornton J, Brennan P, Looby S, O'Hare A. Endovascular repair of posterior communicating artery aneurysms, associated with oculomotor nerve palsy: A review of nerve recovery. Intervent Neuroradiol. 2015;21(3):312-6.
201. Shankar JJS, Quateen A, Weill A, Tampieri D, Del Pilar Cortes M, Fahed R, et al. Canadian Registry of LVIS Jr for Treatment of Intracranial Aneurysms (CaRLA). J Neurointervent Surg. 2017;9(9):849-53.
202. Sedat J, Chau Y, Gaudart J, Sachet M, Beuil S, Lonjon M. Stent-assisted coiling of intracranial aneurysms using LEO stents: long-term follow-up in 153 patients. Neuroradiology. 2018;60(2):211-9.
203. Sedat J, Chau Y, Mondot L, Vargas J, Szapiro J, Lonjon M. Endovascular occlusion of intracranial wide-necked aneurysms with stenting (Neuroform) and coiling: Mid-term and long-term results. Neuroradiology. 2009;51(6):401-9.
204. Sedat J, Dib M, Lonjon M, Litrico S, Von Langsdorf D, Fontaine D, et al. Endovascular treatment of ruptured intracranial aneurysms in patients aged 65 years and older: Follow-up of 52 patients after 1 year. Stroke. 2002;33(11):2620-5.
205. Sattur MG, Li Y, Almallouhi E, Lena J, Spiotta AM. Lessons Learned from Endovascular Coil Embolization of Pericallosal Artery Aneurysms and Adoption of Flow Diversion: A Retrospective Cohort Assessment of the Efficacy of Coiling and Flow Diversion. World Neurosurg. 2019;129:e444-e51.
206. Saraf R, Shrivastava M, Siddhartha W, Limaye U. Intracranial pediatric aneurysms: Endovascular treatment and its outcome. J Neursurg Pediatr. 2012;10(3):230-40.
207. Santillan A, Schwarz J, Boddu S, Gobin YP, Knopman J, Patsalides A. Stent-assisted coil embolization of anterior communicating artery aneurysms using the LVIS Jr stent. Interv neuroradiol. 2019;25(1):12-20.
208. Santillan A, Boddu S, Schwarz J, Lin N, Gobin YP, Knopman J, et al. LVIS Jr. stent for treatment of intracranial aneurysms with parent vessel diameter of 2.5 mm or less. Intervent Neuroradiol. 2018;24(3):246-53.
209. Sandalcioglu IE, Wanke I, Schoch B, Gasser T, Regel JP, Doerfler A, et al. Endovascularly or surgically treated vertebral artery and posterior inferior cerebellar artery aneurysms: Clinical analysis and results. Zentralbl Neurochir. 2005;66(1):9-16.
210. Samaniego EA, Mendez AA, Nguyen TN, Kalousek V, Guerrero WR, Dandapat S, et al. LVIS Jr Device for Y-Stent-Assisted Coil Embolization of Wide-Neck Intracranial Aneurysms: A Multicenter Experience. Intervent Neurol. 2018;7(5):271-83.

© 2023 Volovici V et al. *JAMA Network Open.*

211. Adeeb N, Griessenauer CJ, Shallwani H, Shakir H, Foreman PM, Moore JM, et al. Pipeline Embolization Device in Treatment of 50 Unruptured Large and Giant Aneurysms. World Neurosurg. 2017;105:232-7.
212. Ailani AG, Saiful Azli MN, Regunath K, Azmin Kass R, Abdul Rahman Izani G. Characteristics and outcomes of patients with anterior circulation intracranial aneurysm managed with clipping in Hospital Sungai Buloh. Malays J Med Sci. 2016;23(6):113-7.
213. Abla AA, McDougall CM, Breshears JD, Lawton MT. Intracranial-to-intracranial bypass for posterior inferior cerebellar artery aneurysms: Options, technical challenges, and results in 35 patients. J Neurosurg. 2016;124(5):1275-86.
214. Aydin Y, Çavusoglu H, Kahyaoglu O, Müslüman AM, Yilmaz A, Türkmenoglu ON, et al. Clip ligation of unruptured intracranial aneurysms: A prospective midterm outcome study. Acta Neurochir. 2012;154(7):1135-44.
215. Gory B, Turjman F. Endovascular treatment of 404 intracranial aneurysms treated with nexus detachable coils: Short-term and mid-term results from a prospective, consecutive, European multicenter study. Acta Neurochir. 2014;156(5):831-7.
216. Choi SW, Ahn JS, Park JC, Kwon DH, Kwun BD, Kim CJ. Surgical treatment of unruptured intracranial middle cerebral artery aneurysms: angiographic and clinical outcomes in 143 aneurysms. J cerebrovasc endovasc neurosurg. 2012;14(4):289-94.
217. Jung YJ, Ahn JS, Park ES, Kwon DH, Kwun BD, Kim CJ. Surgical results of unruptured intracranial aneurysms in the elderly: Single center experience in the past ten years. J Korean Neurosurg Soc. 2011;49(6):329-33.
218. Adeeb N, Moore JM, Griessenauer CJ, Foreman PM, Shallwani H, Dmytriw AA, et al. Treatment of Tandem Internal Carotid Artery Aneurysms Using a Single Pipeline Embolization Device: Evaluation of Safety and Efficacy. AJNR Am J Neuroradiol. 2017;38(8):1605-9.
219. Cai Y, Spelle L, Wang H, Piotin M, Mounayer C, Vanzin JR, et al. Endovascular treatment of intracranial aneurysms in the elderly: Single-center experience in 63 consecutive patients. Neurosurgery. 2005;57(6):1096-102.
220. Andic C, Aydemir F, Kardes O, Gedikoglu M, Akin S. Single-stage endovascular treatment of multiple intracranial aneurysms with combined endovascular techniques: Is it safe to treat all at once? J Neurointervent Surg. 2017;9(11):1069-74.
221. Akpek S, Arat A, Morsi H, Klucznick RP, Strother CM, Mawad ME. Self-expandable stent-assisted coiling of wide-necked intracranial aneurysms: A single-center experience. Am J Neuroradiol. 2005;26(5):1223-31.
222. Aletich VA, Debrun GM, Misra M, Charbel F, Ausman JI. The remodeling technique of balloon-assisted Guglielmi detachable coil placement in wide-necked aneurysms: Experience at the University of Illinois at Chicago. J Neurosurg. 2000;93(3):388-96.
223. Beneš V, Mitchell P, Molyneux AJ, Renowden SA. Endovascular coiling in 131 patients with low complication rate justifies treating most unruptured intracranial aneurysms. Zentralbl Neurochir. 2010;71(1):1-7.
224. Elewa MK. Endovascular coiling for cerebral aneurysm: single-center experience in Egypt. Egypt J Neurol Psychiat Neurosurg. 2018;54(1):33.
225. Chalouhi N, Jabbour P, Tjoumakaris S, Dumont AS, Chitale R, Rosenwasser RH, et al. Single-center experience with balloon-assisted coil embolization of intracranial aneurysms: Safety, efficacy and indications. Clin Neurol Neurosurg. 2013;115(5):607-13.
226. Liu J, Li X, Sun S, Wang Y, Zang P. Clinical and Angiographic Outcomes of Endovascular Treatment for Ruptured Posterior Circulation Cerebral Aneurysms. Turk Neurosurg. 2016;26(4):513-7.

227. Arustamyan SR, Yakovlev SB, Bocharov AV, Bukharin EY, Dorokhov PS, Mikeladze KG, et al. [Endovascular treatment of large and giant intracranial aneurysms using stent assistance]. Zh Vopr Neirokhir Im N N Burdenko. 2015;79(4):28-37.
228. Bracard S, Abdel-Kerim A, Thuillier L, Klein O, Anxionnat R, Finitsis S, et al. Endovascular coil occlusion of 152 middle cerebral artery aneurysms: Initial and midterm angiographic and clinical results. J Neurosurg. 2010;112(4):703-8.
229. Bavinzski G, Killer M, Gruber A, Reinprecht A, Gross CE, Richling B. Treatment of basilar artery bifurcation aneurysms by using Guglielmi detachable coils: a 6-year experience. J Neurosurg. 1999;90(5):843-52.
230. Bergui M, Bradac GB. Acute endovascular treatment of ruptured aneurysms in poor-grade patients. Neuroradiology. 2004;46(2):161-4.
231. Bhogal P, AlMatter M, Hellstern V, Ganslandt O, Bazner H, Henkes H, et al. The Combined Use of Intraluminal and Intrasaccular Flow Diversion for the Treatment of Intracranial Aneurysms: Report of 25 Cases. Neurointervention. 2018;13(1):20-31.
232. Gory B, Aguilar-Pérez M, Pomero E, Turjman F, Weber W, Fischer S, et al. PCONus device for the endovascular treatment of wide-neck middle cerebral artery aneurysms. Am J Neuroradiol. 2015;36(9):1735-40.
233. Bhogal P, Martinez Moreno R, Ganslandt O, Bäzner H, Henkes H, Perez MA. Use of flow diverters in the treatment of unruptured saccular aneurysms of the anterior cerebral artery. J Neurointervent Surg. 2017;9(3):283-9.
234. Bhogal P, Perez MA, Ganslandt O, Bazner H, Henkes H, Fischer S. Treatment of posterior circulation non-saccular aneurysms with flow diverters: a single-center experience and review of 56 patients. J Neurointerv Surg. 2017;9(5):471-81.
235. Cho YD, Lee WJ, Kim KM, Kang HS, Kim JE, Han MH. Stent-assisted coil embolization of posterior communicating artery aneurysms. Am J Neuroradiol. 2013;34(11):2171-6.
236. Gory B, Klisch J, Bonafé A, Mounayer C, Beaujeux R, Moret J, et al. Solitaire AB stent-assisted coiling of wide-necked intracranial aneurysms: Short-term results from a prospective, consecutive, European multicentric study. Neuroradiology. 2013;55(11):1373-8.
237. Chiu AHY, Cheung AK, Wenderoth JD, De Villiers L, Rice H, Phatouros CC, et al. Long-term follow-up results following elective treatment of unruptured intracranial aneurysms with the pipeline embolization device. Am J Neuroradiol. 2015;36(9):1728-34.
238. Chae KS, Jeon P, Kim KH, Kim ST, Kim HJ, Byun HS. Endovascular coil embolization of very small intracranial aneurysms. Kor J Radiol. 2010;11(5):536-41.
239. Lin N, Lanzino G, Lopes DK, Arthur AS, Ogilvy CS, Ecker RD, et al. Treatment of distal anterior circulation aneurysms with the pipeline embolization device: A US multicenter experience. Clin Neurosurgery. 2016;79(1):14-22.
240. Caroff XJ, Mihalea C, Klisch J, Strasilla C, Berlis A, Patankar T, et al. Single-layer webs: Intrasaccular flow disrupters for aneurysm treatment-feasibility results from a european study. Am J Neuroradiol. 2015;36(10):1942-6.
241. Johnson AK, Heiferman DM, Lopes DK. Stent-assisted embolization of 100 middle cerebral artery aneurysms: Clinical article. J Neurosurg. 2013;118(5):950-5.
242. Kühn AL, Hou SY, Puri AS, Silva CF, Gounis MJ, Wakhloo AK. Stent-assisted coil embolization of aneurysms with small parent vessels: Safety and efficacy analysis. J Neurointervent Surg. 2016;8(6):581-5.
243. Geyik S, Yavuz K, Yurttutan N, Saatci I, Cekirge HS. Stent-assisted coiling in endovascular treatment of 500 consecutive cerebral aneurysms with long-term follow-up. Am J Neuroradiol. 2013;34(11):2157-62.
244. Byrne JV. Acute endovascular treatment by coil embolisation of ruptured intracranial aneurysms. Ann R Coll Surg Engl. 2001;83(4):253-6.

© 2023 Volovici V et al. *JAMA Network Open.*

245. Kang HS, Han MH, Lee TH, Shin YS, Roh HG, Kwon OK, et al. Embolization of intracranial aneurysms with hydrogel-coated coils: Result of a Korean multicenter trial. Neurosurgery. 2007;61(1):51-8.
246. Bonadio LE, Mello LRG, Boer VHT, Haas LJ, Bernardes CIC, De Lara D, et al. Pericallosal aneurysms: Effectiveness of endovascular management. Arq Bras Neurocir. 2017;36(1):7-13.
247. Das KK, Singh S, Sharma P, Mehrotra A, Bhaisora K, Sardhara J, et al. Results of Proactive Surgical Clipping in Poor-Grade Aneurysmal Subarachnoid Hemorrhage: Pattern of Recovery and Predictors of Outcome. World Neurosurg. 2017;102:561-70.
248. Giacomini L, Piske RL, Baccin CE, Barroso M, Joaquim AF, Tedeschi H. Neurovascular reconstruction with flow diverter stents for the treatment of 87 intracranial aneurysms: Clinical results. Intervent Neuroradiol. 2015;21(3):292-9.
249. Pahl FH, de Oliveira MF, Brock RS, Lucio JE, Rotta JM. Surgical clipping is still a good choice for the treatment of paraclinoid aneurysms. Arq Neuropsiquiatr. 2016;74(4):314-9.
250. Oishi H, Yoshida K, Shimizu T, Yamamoto M, Horinaka N, Arai H. Endovascular treatment with bare platinum coils for middle cerebral artery aneurysms. Neurol Med -Chir. 2009;49(7):287-93.
251. Chung J, Lim YC, Suh SH, Shim YS, Kim YB, Joo JY, et al. Stent-assisted coil embolization of ruptured wide-necked aneurysms in the acute period: incidence of and risk factors for periprocedural complications. J Neurosurg. 2014;121(1):4-11.
252. Cho YD, Sohn CH, Kang HS, Kim JE, Cho WS, Hwang G, et al. Coil embolization of intracranial saccular aneurysms using the Low-profile Visualized Intraluminal Support (LVIS™) device. Neuroradiology. 2014;56(7):543-51.
253. Fargen KM, Blackburn S, Carpenter JS, Jabbour P, Mack WJ, Rai AT, et al. Early results of the Axium MicroFX for Endovascular Repair of IntraCranial Aneurysm (AMERICA) study: A multicenter prospective observational registry. J Neurointervent Surg. 2014;6(7):495-9.
254. Choulakian A, Drazin D, Alexander MJ. Endosaccular treatment of 113 cavernous carotid artery aneurysms. J Neurointerv Surg. 2010;2(4):359-62.
255. Geyik S, Yavuz K, Ergun O, Koc O, Cekirge S, Saatci I. Endovascular treatment of intracranial aneurysms with bioactive Cerecyte coils: Effects on treatment stability. Neuroradiology. 2008;50(9):787-93.
256. Lylyk P, Ferrario A, Pabón B, Miranda C, Doroszuk G. Buenos Aires experience with the Neuroform self-expanding stent for the treatment of intracranial aneurysms. J Neurosurg. 2005;102(2):235-41.
257. Durst C, Starke RM, Gaughen J, Nguyen Q, Patrie J, Jensen ME, et al. Vision outcomes and major complications after endovascular coil embolization of ophthalmic segment aneurysms. Am J Neuroradiol. 2014;35(11):2140-5.
258. Niemann D, Aviv R, Cowsill C, Sneade M, Molyneux AJ. Anatomically conformable, three-dimensional, detachable platinum microcoil system for the treatment of intracranial aneurysms. Am J Neuroradiol. 2004;25(5):813-8.
259. Klisch J, Clajus C, Sychra V, Eger C, Strasilla C, Rosahl S, et al. Coil embolization of anterior circulation aneurysms supported by the solitaire™ AB neurovascular remodeling device. Neuroradiology. 2010;52(5):349-59.
260. Gu DQ, Zhang X, Luo B, Long XA, Duan CZ. The effect of Neuroform stent-assisted coil embolization of wide-necked intracranial aneurysms and clinical factors on progressive aneurysm occlusion on angiographic follow-up. J Clin Neurosci. 2013;20(2):244-7.
261. Guglielmi G, Viñuela F, Duckwiler G, Jahan R, Cotroneo E, Gigli R. Endovascular treatment of 306 anterior communicating artery aneurysms: Overall, perioperative results: Clinical article. J Neurosurg. 2009;110(5):874-9.
262. Ilyas A, Buell TJ, Chen CJ, Ding D, Raper DMS, Taylor DG, et al. SMART coils for intracranial aneurysm embolization: Initial outcomes. Clin Neurol Neurosurg. 2018;164:87-91.
263. Calvacante T, Derrey S, Curey S, Langlois O, Fréger P, Gérardin E, et al. Distal middle cerebral artery aneurysm: A proposition of microsurgical management. Neurochirurgie. 2013;59(3):121-7.

© 2023 Volovici V et al. *JAMA Network Open.*

264. Alghamdi F, Mine B, Morais R, Scillia P, Lubicz B. Stent-assisted coiling of intracranial aneurysms located on small vessels: midterm results with the LVIS Junior stent in 40 patients with 43 aneurysms. Neuroradiology. 2016;58(7):665-71.
265. O'Kelly CJ, Spears J, Chow M, Wong J, Boulton M, Weill A, et al. Canadian experience with the pipeline embolization device for repair of unruptured intracranial aneurysms. Am J Neuroradiol. 2013;34(2):381-7.
266. Mocco J, Snyder KV, Albuquerque FC, Bendok BR, Boulos AS, Carpenter JS, et al. Treatment of intracranial aneurysms with the Enterprise stent: A multicenter registry - Clinical article. J Neurosurg. 2009;110(1):35-9.
267. Colby GP, Bender MT, Lin LM, Beaty N, Caplan JM, Jiang B, et al. Declining complication rates with flow diversion of anterior circulation aneurysms after introduction of the Pipeline Flex: Analysis of a single-institution series of 568 cases. J Neurosurg. 2018;129(6):1475-81.
268. Invergo D, Gordhan A. Endovascular treatment of cerebral aneurysms at a low-volume community hospital practice: Management strategies, complications, and outcomes. J Neuroimaging. 2012;22(3):233-42.
269. Galal A, Bahrassa F, Dalfino JC, Boulos AS. Stent-assisted treatment of unruptured and ruptured intracranial aneurysms: Clinical and angiographic outcome. Br J Neurosurg. 2013;27(5):607-16.
270. Cho YD, Ahn JH, Jung SC, Kim CH, Cho WS, Kang HS, et al. Single-Stage Coil Embolization of Multiple Intracranial Aneurysms: Technical Feasibility and Clinical Outcomes. Clin Neurorad. 2016;26(3):285-90.
271. Lan Q, Gong Z, Kang D, Zhang H, Qian Z, Chen J, et al. Microsurgical experience with keyhole operations on intracranial aneurysms. Surg Neurol. 2006;66(SUPPL. 1):S2-S9.
272. Lubicz B, Leclerc X, Gauvrit JY, Lejeune JP, Pruvo JP. Endovascular Treatment of Ruptured Intracranial Aneurysms in Elderly People. Am J Neuroradiol. 2004;25(4):592-5.
273. Grunwald IQ, Papanagiotou P, Struffert T, Politi M, Krick C, Gül G, et al. Recanalization after endovascular treatment of intracerebral aneurysms. Neuroradiology. 2007;49(1):41-7.
274. Muto M, Giurazza F, Ambrosanio G, Vassallo P, Briganti F, Tecame M, et al. Stent-assisted coiling in ruptured cerebral aneurysms: multi-center experience in acute phase. Radiol Med. 2017;122(1):43-52.
275. Kaya T, Daglioglu E, Gurkas E, Akmangit I, Peker A, Belen D, et al. Silk Device for the Treatment of Intracranial Aneurysms, Part 2: Factors Related to Clinical and Angiographic Outcome. Turk Neurosurg. 2016;26(4):533-7.
276. Kocer N, Islak C, Kizilkilic O, Kocak B, Saglam M, Tureci E. Flow re-direction endoluminal device in treatment of cerebral aneurysms: Initial experience with short-term follow-up results: Clinical article. J Neurosurg. 2014;120(5):1158-71.
277. Moritz JLW, Vendrell JF, Hoa D, Menjot N, Costalat V, Brunel H, et al. Mid-term clinical and angiographic results of cerebral aneurisms treated with matrix2® coils. J Neuroradiol. 2012;39(5):326-31.
278. Leonardi M, Cirillo L, Toni F, Dall'Olio M, Princiotta C, Stafa A, et al. Treatment of intracranial aneurysms using flow-diverting silk stents (BALT): A single centre experience. Intervent Neuroradiol. 2011;17(3):306-15.
279. de Sousa AA, Filho MA, Faglioni W, Jr., Carvalho GT. Unilateral pterional approach to bilateral aneurysms of the middle cerebral artery. Surg Neurol. 2005;63 Suppl 1:S1-7.
280. Alurkar A, Karanam LS, Nayak S, Oak S. Stent-assisted coiling in ruptured wide-necked aneurysms: A single-center analysis. Surg Neurol Int. 2012;3:131.
281. Ferrell AS, Lessne ML, Alexander MJ, Shah P, Golshani K, Zomorodi A, et al. Visual complications after stent-assisted endovascular embolization of paraophthalmic and suprasellar variant superior hypophyseal aneurysms: The duke cerebrovascular center experience in 57 patients. World Neurosurg. 2012;78(3-4):289-94.

282. Fiorella D, Albuquerque FC, Deshmukh VR, McDougall CG. Usefulness of the Neuroform stent for the treatment of cerebral aneurysms: Results at initial (3-6-mo) follow-up. Neurosurgery. 2005;56(6):1191-201.
283. Maimon S, Gonen L, Nossek E, Strauss I, Levite R, Ram Z. Treatment of intra-cranial aneurysms with the SILK flow diverter: 2 years' experience with 28 patients at a single center. Acta Neurochir. 2012;154(6):979-87.
284. McDougall CG, Halbach VV, Dowd CF, Higashida RT, Larsen DW, Hieshima GB. Endovascular treatment of basilar tip aneurysms using electrolytically detachable coils. J NEUROSURG. 1996;84(3):393-9.
285. Lubicz B, Collignon L, Raphaeli G, Pruvo JP, Bruneau M, De Witte O, et al. Flow-diverter stent for the endovascular treatment of intracranial aneurysms: A prospective study in 29 patients with 34 aneurysms. Stroke. 2010;41(10):2247-53.
286. Bae IS, Yi HJ, Ko Y, Kim YS, Chun HJ, Choi KS. Practical Incidence of Complications and Degree of Patient Satisfaction After Endovascular Coil Embolization for Unruptured Intracranial Saccular Aneurysm Based on Patients' Surveys. World Neurosurg. 2019;127:e76-e85.
287. Bartolini B, Blanc R, Pistocchi S, Redjem H, Ciccio G, Piotin M. TransForm occlusion balloon catheter for the treatment of intracranial aneurysms, initial experience. Intervent Neuroradiol. 2015;21(2):155-60.
288. Atallah E, Saad H, Mouchtouris N, Bekelis K, Walker J, Chalouhi N, et al. Pipeline for Distal Cerebral Circulation Aneurysms. Neurosurgery. 2019.
289. Aydin K, Arat A, Sencer S, Barburoglu M, Men S. Stent-assisted coiling of wide-neck intracranial aneurysms using low-profile leo baby stents: Initial and midterm results. Am J Neuroradiol. 2015;36(10):1934-41.
290. Aydin K, Men S, Barburoglu M, Sencer S, Akpek S. Initial and long-term outcomes of complex bifurcation aneurysms treated by Y-stent-assisted coiling with low-profile braided stents. Am J Neuroradiol. 2018;39(12):2284-90.
291. Ban SP, Hwang G, Kim CH, Byoun HS, Lee SU, Kim T, et al. Risk factor analysis of recanalization and retreatment for patients with endovascular treatment of internal carotid artery bifurcation aneurysms. Neuroradiology. 2018;60(5):535-44.
292. Bartolini B, Blanc R, Pistocchi S, Redjem H, Piotin M. "Y" and "X" stent-assisted coiling of complex and wide-neck intracranial bifurcation aneurysms. Am J Neuroradiol. 2014;35(11):2153-8.
293. Bechan RS, Majoie CB, Sprengers ME, Peluso JP, Sluzewski M, van Rooij WJ. Therapeutic Internal Carotid Artery Occlusion for Large and Giant Aneurysms: A Single Center Cohort of 146 Patients. AJNR Am J Neuroradiol. 2016;37(1):125-9.
294. Geyik S, Yavuz K, Cekirge S, Saatci I. Endovascular treatment of basilar and ICA termination aneurysms: Effects of the use of HydroCoils on treatment stability in a subgroup of patients prone to a higher recurrence rate. Neuroradiology. 2007;49(12):1015-21.
295. Deng D, Sun H, Jin D, Qu K, Zhou J, Piao X, et al. Stent-assisted coiling of complex intracranial aneurysms. Neurosurg Q. 2012;22(3):153-8.
296. Çinar C, Bozkaya H, Oran I. Endovascular treatment of cranial aneurysms with the pipeline flow-diverting stent: Preliminary mid-term results. Diagn Intervention Radiol. 2013;19(2):154-64.
297. Martínez-Galdámez M, Romance A, Vega P, Vega A, Caniego JL, Paul L, et al. Pipeline endovascular device for the treatment of intracranial aneurysms at the level of the circle of Willis and beyond: Multicenter experience. J Neurointervent Surg. 2015;7(11):816-23.
298. Lopes DK, Johnson AK, Kellogg RG, Heiferman DM, Keigher KM. Long-term radiographic results of Stent-assisted Embolization of cerebral aneurysms. Neurosurgery. 2014;74(3):286-91.
299. Colby GP, Lin LM, Caplan JM, Jiang B, Huang J, Tamargo RJ, et al. Immediate procedural outcomes in 44 consecutive Pipeline Flex cases: The first North American single-center series. J Neurointervent Surg. 2016;8(7):702-9.

300. Iijima A, Piotin M, Mounayer C, Spelle L, Weill A, Moret J. Endovascular treatment with coils of 149 middle cerebral artery berry aneurysms. Radiology. 2005;237(2):611-9.
301. Linzey JR, Griauzde J, Guan Z, Bentley N, Gemmete JJ, Chaudhary N, et al. Stent-assisted coiling of cerebrovascular aneurysms: Experience at a large tertiary care center with a focus on predictors of recurrence. J Neurointervent Surg. 2017;9(11):1081-5.
302. Molyneux AJ, Cekirge S, Saatci I, Gál G. Cerebral Aneurysm Multicenter European Onyx (CAMEO) Trial: Results of a Prospective Observational Study in 20 European Centers. Am J Neuroradiol. 2004;25(1):39-51.
303. Hokari M, Kuroda S, Nakayama N, Houkin K, Ishikawa T, Kamiyama H. Long-term prognosis in patients with clipped unruptured cerebral aneurysms - Increased cerebrovascular events in patients with surgically treated unruptured aneurysms. Neurosurg Rev. 2013;36(4):567-71.
304. Vora N, Thomas AJ, Gupta R, Gologorsky Y, Panapitiya N, Jovin T, et al. Endovascular treatment of distal anterior cerebral artery aneurysms: Technical results and review of the literature. J Neuroimaging. 2010;20(1):70-3.
305. Colby GP, Lin LM, Gomez JF, Paul AR, Huang J, Tamargo RJ, et al. Immediate procedural outcomes in 35 consecutive pipeline embolization cases: A single-center, single-user experience. J Neurointervent Surg. 2013;5(3):247-52.
306. Lubicz B, Klisch J, Gauvrit JY, Szikora I, Leonardi M, Liebig T, et al. WEB-DL endovascular treatment of wide-neck bifurcation aneurysms: Short- and midterm results in a European study. Am J Neuroradiol. 2014;35(3):432-8.
307. Abdulrauf SI, Vuong P, Patel R, Sampath R, Ashour AM, Germany LM, et al. "Awake" clipping of cerebral aneurysms: Report of initial series. J Neurosurg. 2017;127(2):311-8.
308. Lee S, Gong TS, Lee YW, Kim HJ, Kweon CY. Results of Endovascular Coil Embolization Treatment for Small (<= 5 mm) Unruptured Intracranial Aneurysms. J cerebrovasc endovasc neurosurg. 2016;18(3):229-33.
309. Yang X, Wu Z, Mu S, Li Y, Lv M. Endovascular treatment of giant and large intracranial aneurysms using the neuroform stent-assisted coil placement. Neurol Res. 2008;30(6):598-602.
310. Fischer S, Vajda Z, Perez MA, Schmid E, Hopf N, Bäzner H, et al. Pipeline embolization device (PED) for neurovascular reconstruction: Initial experience in the treatment of 101 intracranial aneurysms and dissections. Neuroradiology. 2012;54(4):369-82.
311. Xu X, Shang XM, Cui JZ, Wang DY. Endovascular treatment of intracranial aneurysms using coil embolization plus an Enterprise stent. Chin Med J. 2011;124(4):611-4.
312. Feng X, Wang L, Guo E, Zhang B, Qian Z, Liu P, et al. Progressive Occlusion and Recanalization After Endovascular Treatment for 287 Unruptured Small Aneurysms (<5mm): A Single-Center 6-Year Experience. World Neurosurg. 2017;103:576-83.
313. Becske T, Potts MB, Shapiro M, Kallmes DF, Brinjikji W, Saatci I, et al. Pipeline for uncoilable or failed aneurysms: 3-year follow-up results. J Neurosurg. 2017;127(1):81-8.
314. Debrun GM, Aletich VA, Thornton J, Alazzaz A, Charbel FT, Ausman JI, et al. Techniques of coiling cerebral aneurysms. Surg Neurol. 2000;53(2):150-6.
315. Behme D, Berlis A, Weber W. Woven endo bridge intrasaccular flow disrupter for the treatment of ruptured and unruptured wide-neck cerebral aneurysms: Report of 55 Cases. Am J Neuroradiol. 2015;36(8):1501-6.
316. Machi P, Costalat V, Lobotesis K, Ruiz C, Cheikh YB, Eker O, et al. LEO baby stent use following balloon-assisted coiling: Single- and dual-stent technique-immediate and midterm results of 29 consecutive patients. Am J Neuroradiol. 2015;36(11):2096-103.
317. Lozier AP, Kim GH, Sciacca RR, Connolly ES, Solomon RA, Batjer HH, et al. Microsurgical Treatment of Basilar Apex Aneurysms: Perioperative and Long-term Clinical Outcome. Neurosurgery. 2004;54(2):286-99.

© 2023 Volovici V et al. *JAMA Network Open.*

318. Oishi H, Yamamoto M, Shimizu T, Yoshida K, Arai H. Endovascular therapy of 500 small asymptomatic unruptured intracranial aneurysms. Am J Neuroradiol. 2012;33(5):958-64.
319. Raymond J, Guilbert F, Roy D. Neck-bridge device for endovascular treatment of wide-neck bifurcation aneurysms: initial experience. Radiology. 2001;221(2):318-26.
320. Wang H, Xu D, Xiang Y, Li W, Chen Z, Chen S, et al. Endovascular treatment for wide-necked intracranial aneurysms with the Enterprise stent. Neurol India. 2011;59(4):548-52.
321. Behme D, Weber A, Kowoll A, Berlis A, Burke TH, Weber W. Low-profile visualized intraluminal support device (LVIS Jr) as a novel tool in the treatment of wide-necked intracranial aneurysms: Initial experience in 32 cases. J Neurointervent Surg. 2015;7(4):281-5.
322. Asiltürk M, Abdallah A. Clinical outcomes of multiple aneurysms microsurgical clipping: Evaluation of 90 patients. Neurol Neurochir Pol. 2018;52(1):15-24.
323. Lubicz B, Balériaux D, Lefranc F, Brotchi J, Bruneau M, Levivier M. Endovascular treatment of intracranial aneurysms as the first thérapeutic option. J Neuroradiol. 2007;34(4):250-9.
324. Gupta SK, Ghanta RK, Chhabra R, Mohindra S, Mathuriya SN, Mukherjee KK, et al. Poor-grade subarachnoid hemorrhage: is surgical clipping worthwhile? Neurol India. 2011;59(2):212-7.
325. Kulcsár Z, Göricke SL, Gizewski ER, Schlamann M, Sure U, Sandalcioglu IE, et al. Neuroform stent-assisted treatment of intracranial aneurysms: Long-term follow-up study of aneurysm recurrence and in-stent stenosis rates. Neuroradiology. 2013;55(4):459-65.
326. Bender MT, Colby GP, Jiang B, Lin LM, Campos JK, Xu R, et al. Flow diversion of posterior circulation cerebral aneurysms: A single-institution series of 59 cases. Neurosurgery. 2019;84(1):206-16.
327. Benitez RP, Silva MT, Klem J, Veznedaroglu E, Rosenwasser RH, Halbach VV, et al. Endovascular occlusion of wide-necked aneurysms with a new intracranial microstent (neuroform) and detachable coils. Neurosurgery. 2004;54(6):1359-68.
328. Bohnstedt BN, Nguyen HS, Kulwin CG, Shoja MM, Helbig GM, Leipzig TJ, et al. Outcomes for clip ligation and hematoma evacuation associated with 102 patients with ruptured middle cerebral artery aneurysms. World Neurosurg. 2013;80(3-4):335-41.
329. Pinsker MO, Gerstner W, Wolf S, Trost HA, Lumenta CB. Surgery and outcome for aneurysmal subarachnoid hemorrhage in elderly patients. Acta Neurochir Suppl. 2002;82:61-4.
330. Su W, Zhang Y, Chen J, Liu J, Rajah G, Yang X. 225 intracranial aneurysms treated with the Low-profile Visualized Intraluminal Support (LVIS) stent: a single-center retrospective study. Neurol Res. 2018;40(6):445-51.
331. Limbucci N, Renieri L, Nappini S, Consoli A, Rosi A, Mangiafico S. Y-stent assisted coiling of bifurcation aneurysms with Enterprise stent: Long-term follow-up. J Neurointervent Surg. 2016;8(2):158-62.
332. Sawada M, Kaku Y, Hayashi K, Ueda T, Yoshimura S, Sakai N. Endovascular treatment of ruptured intracranial aneurysms using platinum coils in patients over 70 years of age. Intervent Neuroradiol. 2000;6(SUPPL. 1):85-7.
333. Tomasello A, Romero N, Aixut S, Miquel MA, Macho JM, Castaño C, et al. Endovascular treatment of intracraneal aneurysm with pipeline embolization device: experience in four centres in Barcelona. Neurol Res. 2016;38(5):381-8.
334. Lv X, Ge H, Jin H, He H, Jiang C, Li Y. Endovascular treatment of unruptured posterior circulation intracranial aneurysms. Ann Indian Acad Neurol. 2016;19(3):302-6.
335. Mangiafico S, Cellerini M, Villa G, Nistri M, Pandolfo C, Ammannati F, et al. Utility of balloon-assisted Guglielmi detachable coiling in the treatment of cerebral aneurysms: A single center retrospective study. Intervent Neuroradiol. 2002;8(3):235-43.
336. Khan N, Yoshimura S, Roth P, Cesnulis E, Koenue-Leblebicioglu D, Curcic M, et al. Conventional microsurgical treatment of paraclinoid aneurysms: state of the art with the use of the selective extradural anterior clinoidectomy SEAC. Acta Neurochir Suppl. 2005;94:23-9.

© 2023 Volovici V et al. *JAMA Network Open.*

337. Mejdoubi M, Gigaud M, Trémoulet M, Albucher JF, Cognard C. Initial primary endovascular treatment in the management of ruptured intracranial aneurysms: A prospective consecutive series. Neuroradiology. 2006;48(12):899-905.
338. Panigrahi MK, Mani MK. An institutional experience with microsurgical clipping of 170 consecutive cases of intracranial aneurysms: A retrospective data analysis of personal cases. Arch Neurosci. 2016;3(3).
339. Terada T, Tsuura M, Matsumoto H, Masuo O, Tsumoto T, Yamaga H, et al. Endovascular treatment of unruptured cerebral aneurysms. Acta Neurochir Suppl. 2005;94:87-91.
340. Berenstein A, Song JK, Niimi Y, Namba K, Heran NS, Brisman JL, et al. Treatment of cerebral aneurysms with hydrogel-coated platinum coils (HydroCoil): Early single-center experience. Am J Neuroradiol. 2006;27(9):1834-40.
341. Berge J, Biondi A, Machi P, Brunel H, Pierot L, Gabrillargues J, et al. Flow-diverter silk stent for the treatment of intracranial aneurysms: 1-Year follow-up in a multicenter study. Am J Neuroradiol. 2012;33(6):1150-5.
342. Krisht AF, Gomez J, Partington S. Outcome of surgical clipping of unruptured aneurysms as it compares with a 10-year nonclipping survival period. Neurosurgery. 2006;58(2):207-14.
343. Tateshima S, Murayama Y, Gobin YP, Duckwiler GR, Guglielmi G, Vinuela F, et al. Endovascular treatment of basilar tip aneurysms using Guglielmi detachable coils: Anatomic and clinical outcomes in 73 patients from a single institution. Neurosurgery. 2000;47(6):1332-42.
344. Wang B, Gao BL, Xu GP, Xiang C, Liu XS. Endovascular embolization is applicable for large and giant intracranial aneurysms: Experience in one center with long-term angiographic follow-up. Acta Radiol. 2015;56(1):105-13.
345. Bulters DO, Santarius T, Chia HL, Parker RA, Trivedi R, Kirkpatrick PJ, et al. Causes of neurological deficits following clipping of 200 consecutive ruptured aneurysms in patients with good-grade aneurysmal subarachnoid haemorrhage. Acta Neurochir. 2011;153(2):295-303.
346. Holmin S, Krings T, Ozanne A, Alt JP, Claes A, Zhao W, et al. Intradural saccular aneurysms treated by Guglielmi detachable bare coils at a single institution between 1993 and 2005: clinical long-term follow-up for a total of 1810 patient-years in relation to morphological treatment results. Stroke. 2008;39(8):2288-97.
347. Tähtinen OI, Vanninen RL, Manninen HI, Rautio R, Haapanen A, Niskakangas T, et al. Wide-necked intracranial aneurysms: Treatment with stent-assisted coil embolization during acute (<72 hours) subarachnoid hemorrhage - Experience in 61 consecutive patients. Radiology. 2009;253(1):199-208.
348. Cekirge HS, Saatci I, Ozturk MH, Cil B, Arat A, Mawad M, et al. Late angiographic and clinical follow-up results of 100 consecutive aneurysms treated with Onyx reconstruction: Largest single-center experience. Neuroradiology. 2006;48(2):113-26.
349. Fiorella D, Albuquerque FC, Woo H, Rasmussen PA, Masaryk TJ, McDougall CG. Neuroform stent assisted aneurysm treatment: Evolving treatment strategies, complications and results of long term follow-up. J Neurointervent Surg. 2010;2(1):16-22.
350. Hara T, Arai S, Goto Y, Takizawa T, Uchida T. Bypass surgeries in the treatment of cerebral aneurysms. Acta Neurochir Suppl2016. p. 57-64.
351. Heller RS, Dandamudi V, Calnan D, Malek AM. Neuroform intracranial stenting for aneurysms using simple and multi-stent technique is associated with low risk of magnetic resonance diffusion-weighted imaging lesions. Neurosurgery. 2013;73(4):582-90.
352. Jödicke A, Bauer K, Hajdukova A. Rehabilitation after Clipping of Cerebral Aneurysms without Acute Subarachnoid Hemorrhage: Outcome Analysis of 100 Consecutive Cases. J Neurol Surg Part A Cent Eur Neurosurg. 2018;79(5):391-7.
353. Mocco J, Fargen KM, Albuquerque FC, Bendok BR, Boulos AS, Carpenter JS, et al. Delayed thrombosis or stenosis following enterprise-assisted stent-coiling: Is it safe? Midterm results of the interstate collaboration of enterprise stent coiling. Neurosurgery. 2011;69(4):908-13.

© 2023 Volovici V et al. *JAMA Network Open.*

354. Kremer C, Groden C, Lammers G, Weineck G, Zeumer H, Hansen HC. Outcome after endovascular therapy of ruptured intracranial aneurysms: Morbidity and impact of rebleeding. Neuroradiology. 2002;44(11):942-5.
355. Miyazawa N, Nukui H, Horikoshi T, Yagishita T, Sugita M, Kanemaru K. Surgical management of aneurysms of the bifurcation of the internal carotid artery. Clin Neurol Neurosurg. 2002;104(2):103-14.
356. Arustamyan SR, Yakovlev SB, Shakhnovich AR, Krasnoperov IV, Sazonova OB, Bocharov AV, et al. [Results of deconstructive endovascular surgery in treatment of large and giant intracranial aneurysms]. Zh Vopr Neirokhir Im N N Burdenko. 2016;80(5):22-31.
357. Biondi A, Janardhan V, Katz JM, Salvaggio K, Riina HA, Gobin YP. Neuroform stent-assisted coil embolization of wide-neck intracranial aneurysms: Strategies in stent deployment and midterm follow-up. Neurosurgery. 2007;61(3):460-8.
358. Birchall D, Khangure M, McAuliffe W, Apsimon H, Knuckey N. Endovascular treatment of posterior circulation aneurysms. Br J Neurosurg. 2001;15(1):39-43.
359. Brasiliense LB, Yoon JW, Orina JN, Miller DA, Tawk RG, Hanel RA. A Reappraisal of Anterior Communicating Artery Aneurysms: A Case for Stent-Assisted Embolization. Neurosurgery. 2016;78(2):200-7.
360. Horiuchi T, Hongo K. Clipping surgery for aneurysmal subarachnoid hemorrhage in patients aged 75 years or older. Neurol Res. 2011;33(8):853-7.
361. O'Hare AM, Fanning NF, Ti JP, Dunne R, Brennan PR, Thornton JM. Hydrocoils, occlusion rates, and outcomes: A large single-center study. Am J Neuroradiol. 2010;31(10):1917-22.
362. Miyachi S, Negoro M, Sahara Y, Suzuki O, Hattori K, Kobayashi N, et al. Treatment strategy for cerebral aneurysms based on the evidence of the efficacy of GDC embolization. Intervent Neuroradiol. 2003;9(SUPPL. 1):51-5.
363. Breu AK, Hauser TK, Ebner FH, Bischof F, Ernemann U, Seeger A. Morphologic and Clinical Outcome of Intracranial Aneurysms after Treatment Using Flow Diverter Devices: Mid-Term Follow-Up. Radiol Res Pract. 2016;2016.
364. Xu K, Luo Q, Chen X, Yu JL. A selective clipping microsurgical treatment for multiple intracranial anterior circulation aneurysms. Kuwait Med J. 2014;46(1):21-7.
365. Hirsch JA, Bendok BR, Paulsen RD, Cognard C, Campos J, Cronqvist M. Midterm Clinical Experience with a Complex-shaped Detachable Platinum Coil System for the Treatment of Cerebral Aneurysms: Trufill DCS Orbit Detachable Coil System Registry Interim Results. J Vasc Intervent Radiol. 2007;18(12):1487-94.
366. McLaughlin A, Rice H, de Viliers L, Withers T, Pearson D, Arnell M, et al. Three-year experience with interventional neuroradiology for management of cerebral aneurysms at a single Australian centre. J Med Imaging Radiat Oncol. 2018;62(1):51-6.
367. Mont'alverne F, Musacchio M, Tolentino V, Riquelme C, Tournade A. Endovascular management for intracranial ruptured aneurysms in elderly patients: Outcome and technical aspects. Neuroradiology. 2005;47(6):446-57.
368. Zeeshan Q, Ghodke BV, Juric-Sekhar G, Barber JK, Kim LJ, Sekhar LN. Surgery for very large and giant intracranial aneurysms: Results and complications. Neurol India. 2018;66(6):1741-57.
369. Alhothi AI, Qi T, Guo S, Shi Z, Liang F, Yang L, et al. Neuroform stent-assisted coil embolization: A new treatment strategy for complex intracranial aneurysms. Results of medium length follow-up. Neurol Neurochir Pol. 2010;44(4):366-74.
370. Dabus G, Hacein-Bey L, Varjavand B, Tomalty RD, Han PP, Yerokhin V, et al. Safety, immediate and mid-term results of the newer generation of hydrogel coils in the treatment of ruptured aneurysms: a multicenter study. J Neurointerv Surg. 2017;9(4):419-24.
371. Kim SJ, Choi IS. Midterm outcome of partially thrombosed intracranial aneurysms treated with Guglielmi detachable coils. Intervent Neuroradiol. 2000;6(1):13-22.

372. Castaño C, Terceño M, Remollo S, García-Sort MR, Domínguez C. Endovascular treatment of wide-neck intracranial bifurcation aneurysms with 'Y'-configuration, double Neuroform® stents-assisted coiling technique: Experience in a single center. Intervent Neuroradiol. 2017;23(4):362-70.
373. Mortimer A, Bradley MD, Mews P, Molyneux AJ, Renowden SA. Endovascular treatment of 300 consecutive middle cerebral artery aneurysms: Clinical and radiologic outcomes. Am J Neuroradiol. 2014;35(4):706-14.
374. Bruneau M, Amin-Hanjani S, Koroknay-Pal P, Bijlenga P, Jahromi BR, Lehto H, et al. Surgical clipping of very small unruptured intracranial aneurysms: A multicenter international study. Neurosurgery. 2015;78(1):47-52.
375. Clajus C, Strasilla C, Fiebig T, Sychra V, Fiorella D, Klisch J. Initial and mid-term results from 108 consecutive patients with cerebral aneurysms treated with the WEB device. J Neurointervent Surg. 2017;9(4):411-7.
376. Feng Z, Fang Y, Xu Y, Hong B, Zhao W, Liu J, et al. The safety and efficacy of low profile visualized intraluminal support (LVIS) stents in assisting coil embolization of intracranial saccular aneurysms: A single center experience. J Neurointervent Surg. 2016;8(11):1192-6.
377. Oishi H, Nonaka S, Yamamoto M, Arai H. Feasibility and efficacy of endovascular therapy for ruptured distal anterior cerebral artery aneurysms. Neurol Med -Chir. 2013;53(5):304-9.
378. Iosif C, Piotin M, Saleme S, Barreau X, Sedat J, Chau Y, et al. Safety and effectiveness of the Low Profile Visualized Intraluminal Support (LVIS and LVIS Jr) devices in the endovascular treatment of intracranial aneurysms: Results of the TRAIL multicenter observational study. J Neurointervent Surg. 2018;10(7):679-85.
379. Martínez-Galdámez M, Orlov K, Kadziolka K, Puthuran M, Kalousek V, Pabón B, et al. Safety and efficacy of intracranial aneurysm embolization using the “combined remodeling technique”: low-profile stents delivered through double lumen balloons: a multicenter experience. Neuroradiology. 2019;61(9):1067-72.
380. Kim YS, Joo SP, Kim TS. Microsurgical Management of Ruptured Blood Blister Aneurysms of the Internal Carotid Artery without Bypass: A Retrospective Single-Center Study of 36 Patients over 20 Years. World Neurosurg. 2019;128:e956-e65.
381. Da Ros V, Bozzi A, Comelli C, Semeraro V, Comelli S, Lucarelli N, et al. Ruptured Intracranial Aneurysms Treated with Woven Endobridge Intrasaccular Flow Disruptor: A Multicenter Experience. World Neurosurg. 2019;122:e498-e505.
382. Briganti F, Leone G, Ugga L, Marseglia M, MacEra A, Manto A, et al. Mid-term and long-term follow-up of intracranial aneurysms treated by the p64 Flow Modulation Device: A multicenter experience. J Neurointervent Surg. 2017;9(1):70-6.
383. Li TF, Shui SF, Han XW, Yan L, Ma J, Guo D. One-Stage Endovascular Embolization for Multiple Intracranial Aneurysms. Turk Neurosurg. 2018;28(1):43-7.
384. Jindal G, Miller T, Iyohe M, Shivashankar R, Prasad V, Gandhi D. Small Intracranial Aneurysm Treatment Using Target () Ultrasoft (TM) Coils. J vasc interv radiol neurol. 2016;9(1):46-51.
385. Malisch TW, Guglielmi G, Viñuela F, Duckwiler G, Gobin YP, Martin NA, et al. Intracranial aneurysms treated with the Guglielmi detachable coil: Midterm clinical results in a consecutive series of 100 patients. J NEUROSURG. 1997;87(2):176-83.
386. Jin SC, Kwon DH, Ahn JS, Kwun BD, Song Y, Choi CG. Clinical and radiological outcomes of endovascular detachable coil embolization in paraclinoid aneurysms: A 10-year experience. J Korean Neurosurg Soc. 2009;45(1):5-10.
387. Fiorella D, Boulos A, Turk AS, Siddiqui AH, Arthur AS, Diaz O, et al. The safety and effectiveness of the LVIS stent system for the treatment of wide-necked cerebral aneurysms: Final results of the pivotal US LVIS trial. J Neurointervent Surg. 2019;11(4):357-61.
388. Mihalea C, Caroff J, Pagiola I, Ikka L, Hashemi GB, Naderi S, et al. Safety and efficiency of the fifth

© 2023 Volovici V et al. *JAMA Network Open.*

generation Woven EndoBridge device: Technical note. J Neurointervent Surg. 2019;11(5):511-5.
389. Cheung NK, Chiu AHY, Cheung AK, Wenderoth JD. Long-term follow-up of aneurysms treated electively with woven stent-assisted coiling. J Neurointervent Surg. 2018;10(7):673-8.
390. Li H, Guan J, Liu J, Hou K, Zhao D, Wu G, et al. Treatment of acutely ruptured wide-necked intracranial aneurysms using self-expanding stent. Int J Clin Exp Med. 2015;8(1):1259-64.
391. Peluso JP, van Rooij WJ, Sluzewski M, Beute GN. Coiling of basilar tip aneurysms: results in 154 consecutive patients with emphasis on recurrent haemorrhage and re-treatment during mid- and long-term follow-up. J Neurol Neurosurg Psychiatry. 2008;79(6):706-11.
392. Fargen KM, Hoh BL, Welch BG, Pride GL, Lanzino G, Boulos AS, et al. Long-term results of enterprise stent-assisted coiling of cerebral aneurysms. Neurosurgery. 2012;71(2):239-44.
393. Chalouhi N, Tjoumakaris S, Gonzalez LF, Dumont AS, Starke RM, Hasan D, et al. Coiling of large and giant aneurysms: Complications and long-term results of 334 cases. Am J Neuroradiol. 2014;35(3):546-52.
394. Nussbaum ES, Madison MT, Goddard JK, Lassig JP, Kallmes KM, Nussbaum LA. Microsurgical treatment of unruptured middle cerebral artery aneurysms: A large, contemporary experience. J Neurosurg. 2019;130(5):1498-504.
395. Li TF, Shui SF, Han XW, Yan L, Ma J, Guo D, et al. The use of solitaire AB stents in coil embolization of wide-necked cerebral aneurysms. PLoS ONE. 2015;10(10).
396. De Vries J, Boogaarts J, Van Norden A, Wakhloo AK. New generation of flow diverter (surpass) for unruptured intracranial aneurysms: A prospective single-center study in 37 patients. Stroke. 2013;44(6):1567-77.
397. Dima S, Scheau C, Stefanescu F, Danaila L. Endovascular minimally invasive treatment of the intracranial aneurysms--first 124 cases. J Med Life. 2012;5(3):360-6.
398. Briganti F, Leone G, Cirillo L, de Divitiis O, Solari D, Cappabianca P. Postprocedural, midterm, and long-term results of cerebral aneurysms treated with flow-diverter devices: 7-year experience at a single center. Neurosurg Focus. 2017;42(6):E3.
399. Hai J, Deng DF, Chen ZQ, Pan QG. Endovascular embolization of small ruptured intracranial aneurysms using a biplane angiographic system with three-dimensional rotational digital subtraction angiography. J Clin Neurosci. 2009;16(8):1028-33.
400. Drescher F, Weber W, Berlis A, Rohde S, Carolus A, Fischer S. Treatment of intra- and extracranial aneurysms using the flow-redirection endoluminal device: Multicenter experience and follow-up results. Am J Neuroradiol. 2017;38(1):105-12.
401. Hwang SK, Hwang G, Oh CW, Jin SC, Park H, Bang JS, et al. Endovascular treatment for unruptured intracranial aneurysms in elderly patients: Single-center report. Am J Neuroradiol. 2011;32(6):1087-90.
402. Lu J, Liu JC, Wang LJ, Qi P, Wang DM. Tiny intracranial aneurysms: Endovascular treatment by coil embolisation or sole stent deployment. Eur J Radiol. 2012;81(6):1276-81.
403. Ma X, Yang Y, Zhou Y, Jia W. Endovascular treatment of ruptured intracranial aneurysms in elderly patients: clinical features and treatment outcome. Neurosurg Rev. 2019;42(3):745-51.
404. Briganti F, Leone G, Marseglia M, Mariniello G, Caranci F, Brunetti A, et al. Endovascular treatment of cerebral aneurysms using flow-diverter devices: A systematic review. Neuroradiol J. 2015;28(4):365-75.
405. Brown MA, Parish J, Guandique CF, Payner TD, Horner T, Leipzig T, et al. A long-term study of durability and risk factors for aneurysm recurrence after microsurgical clip ligation. J Neurosurg. 2017;126(3):819-24.
406. Jankowitz BT, Hanel R, Jadhav AP, Loy DN, Frei D, Siddiqui AH, et al. Neuroform Atlas Stent System for the treatment of intracranial aneurysm: Primary results of the Atlas Humanitarian Device Exemption cohort. J Neurointervent Surg. 2019;11(8):801-6.
407. Chen SH, Snelling BM, Shah SS, Sur S, Brunet MC, Starke RM, et al. Transradial approach for flow diversion treatment of cerebral aneurysms: A multicenter study. J Neurointervent Surg. 2019;11(8):796-

800.
408. Ciccio G, Robert T, Smajda S, Fahed R, Desilles JP, Redjem H, et al. Double stent assisted coiling of intracranial bifurcation aneurysms in Y and X configurations with the Neuroform ATLAS stent: immediate and mid term angiographic and clinical follow-up. J Neurointerv Surg. 2019.
409. Imamura H, Sakai N, Ito Y, Sakai C, Hyodo A, Miyachi S, et al. Prospective Registry of Embolization of Intracranial Aneurysms Using HydroSoft Coils: Results of the Japanese HydroSoft Registry. World Neurosurg. 2019;127:e631-e7.
410. D'Urso PI, Karadeli HH, Kallmes DF, Cloft HJ, Lanzino G. Coiling for paraclinoid aneurysms: Time to make way for flow diverters? Am J Neuroradiol. 2012;33(8):1470-4.
411. Park HK, Horowitz M, Jungreis C, Kassam A, Koebbe C, Genevro J, et al. Endovascular treatment of paraclinoid aneurysms: experience with 73 patients. Neurosurgery. 2003;53(1):14-23; discussion 4.
412. Lylyk P, Miranda C, Ceratto R, Ferrario A, Scrivano E, Luna HR, et al. Curative endovascular reconstruction of cerebral aneurysms with the pipeline embolization device: The Buenos Aires experience. Neurosurgery. 2009;64(4):632-42.
413. Liao L, Derelle AL, Merlot I, Civit T, Audibert G, Tonnelet R, et al. Endovascular treatment of distal anterior cerebral artery aneurysms: Long-term results. J Neuroradiol. 2018.
414. Chen Z, Yang Y, Miao H, Li F, Zhang J, Feng H, et al. Experiences and complications in endovascular treatment of paraclinoid aneurysms. J Clin Neurosci. 2013;20(9):1259-63.
415. Hwang SK, Kim SH. Endovascular treatment of intracranial aneurysms using polymer polyglycolic-lactic acid coated coils. EWHA Med J. 2012;35(1):38-43.
416. Hong B, Yang PF, Zhao R, Huang QH, Xu Y, Yang ZG, et al. Endovascular treatment of ruptured tiny intracranial aneurysms. J Clin Neurosci. 2011;18(5):655-60.
417. Friedman JA, Nichols DA, Meyer FB, Pichelmann MA, McIver JI, Toussaint ILG, et al. Guglielmi detachable coil treatment of ruptured saccular cerebral aneurysms: Retrospective review of a 10-year single-center experience. Am J Neuroradiol. 2003;24(3):526-33.
418. Djurdjevic T, Young V, Corkill R, Briley D, Küker W. Treatment of broad-based intracranial aneurysms with low profile braided stents: A single center analysis of 101 patients. J Neurointervent Surg. 2019;11(6):591-7.
419. Gao X, Liang G, Li Z, Wei X, Cao P. A single-centre experience and follow-up of patients with endovascular coiling of large and giant intracranial aneurysms with parent artery preservation. J Clin Neurosci. 2012;19(3):364-9.
420. Park JH, Kang HS, Han MH, Jeon P, Yoo DS, Lee TH. Embolization of intracranial aneurysms with hydrosoft coils: Results of the Korean multicenter study. Am J Neuroradiol. 2011;32(9):1756-61.
421. Piano M, Valvassori L, Quilici L, Pero G, Boccardi E. Midterm and long-term follow-up of cerebral aneurysms treated with flow diverter devices: A single-center experience ; Special topic. J Neurosurg. 2013;118(2):408-16.
422. Brinjikji W, Piano M, Fang S, Pero G, Kallmes DF, Quilici L, et al. Treatment of ruptured complex and large/giant ruptured cerebral aneurysms by acute coiling followed by staged flow diversion. J Neurosurg. 2016;125(1):120-7.
423. Buyukkaya R, Kocaeli H, Yildirim N, Cebeci H, Erdogan C, Hakyemez B. Treatment of complex intracranial aneurysms using flow-diverting silk® stents: An analysis of 32 consecutive patients. Intervent Neuroradiol. 2014;20(6):729-35.
424. Liu Y, Wang J, Lin L, Sang C, Lin Z, Pan Y, et al. Clinical study on complications of intracranial ruptured aneurysm embolization by stent-assisted coil. Med Sci Monit. 2018;24:8115-24.
425. Martínez-Galdámez M, Lamin SM, Lagios KG, Liebig T, Ciceri EF, Chapot R, et al. Periprocedural outcomes and early safety with the use of the Pipeline Flex Embolization Device with Shield Technology for unruptured intracranial aneurysms: Preliminary results from a prospective clinical study. J Neurointervent Surg. 2017;9(8):772-6.

© 2023 Volovici V et al. *JAMA Network Open.*

426. Jin SC, Kwon OK, Oh CW, Bang JS, Hwang G, Park NM, et al. Simple coiling using single or multiple catheters without balloons or stents in middle cerebral artery bifurcation aneurysms. Neuroradiology. 2013;55(3):321-6.
427. Colby GP, Bender MT, Lin LM, Beaty N, Huang J, Tamargo RJ, et al. Endovascular flow diversion for treatment of anterior communicating artery region cerebral aneurysms: a single-center cohort of 50 cases. J Neurointerv Surg. 2017;9(7):679-85.
428. Mericle RA, Reig AS, Burry MV, Eskioglu E, Firment CS, Santra S. Endovascular surgery for proximal posterior inferior cerebellar artery aneurysms: an analysis of Glasgow Outcome Score by Hunt-Hess grades. Neurosurgery. 2006;58(4):619-25; discussion -25.
429. Mine B, Aljishi A, D'Harcour JB, Brisbois D, Collignon L, Lubicz B. Stent-assisted coiling of unruptured intracranial aneurysms: Long-term follow-up in 164 patients with 183 aneurysms. J Neuroradiol. 2014;41(5):322-8.
430. Luo CB, Teng MMH, Chang FC, Lin CJ, Guo WY, Chang CY. Stent-assisted coil embolization of intracranial aneurysms: A single center experience. J Chin Med Assoc. 2012;75(7):322-8.
431. Ma L, Xu JC, Yan S, Feng H, Han HJ, Tan HQ, et al. A single-center experience in the endovascular treatment of carotid siphon aneurysms using the Willis covered stent: a retrospective analysis. J Neurointerv Surg. 2018;10(12):1197-202.
432. Lee SJ, Cho YD, Kang HS, Kim JE, Han MH. Coil embolization using the self-expandable closed-cell stent for intracranial saccular aneurysm: A single-center experience of 289 consecutive aneurysms. Clin Radiol. 2013;68(3):256-63.
433. Huang QH, Wu YF, Shen J, Hong B, Yang PF, Xu Y, et al. Endovascular treatment of acutely ruptured, wide-necked anterior communicating artery aneurysms using the Enterprise stent. J Clin Neurosci. 2013;20(2):267-71.
434. Delgado Almandoz JE, Kayan Y, Tenreiro A, Wallace AN, Scholz JM, Fease JL, et al. Clinical and angiographic outcomes in patients with intracranial aneurysms treated with the pipeline embolization device: intra-procedural technical difficulties, major morbidity, and neurological mortality decrease significantly with increased operator experience in device deployment and patient management. Neuroradiology. 2017;59(12):1291-9.
435. Katsaridis V, Papagiannaki C, Violaris C. Embolization of acutely ruptured and unruptured wide-necked cerebral aneurysms using the neuroform2 stent without pretreatment with antiplatelets: A single center experience. Am J Neuroradiol. 2006;27(5):1123-8.
436. Kawabata Y, Nakazawa T, Fukuda S, Kawarazaki S, Aoki T, Morita T, et al. Endovascular embolization of branch-incorporated cerebral aneurysms. Neuroradiol J. 2017;30(6):600-6.
437. Caragliano AA, Papa R, Pitrone A, Limbucci N, Nappini S, Ruggiero M, et al. The low-profile Neuroform Atlas stent in the treatment of wide-necked intracranial aneurysms - immediate and midterm results: An Italian multicenter registry. 2019.
438. Cekirge HS, Yavuz K, Geyik S, Saatci I. HyperForm balloon remodeling in the endovascular treatment of anterior cerebral, middle cerebral, and anterior communicating artery aneurysms: Clinical and angiographic follow-up results in 800 consecutive patients. Clinical article. J Neurosurg. 2011;114(4):944-53.
439. Choi HH, Cho YD, Yoo DH, Ahn SJ, Cho WS, Kang HS, et al. Stent-assisted coil embolization of anterior communicating artery aneurysms: Safety, effectiveness, and risk factors for procedural complications or recanalization. J Neurointervent Surg. 2019;11(1):49-56.
440. Choi HH, Cho YD, Yoo DH, Yeon EK, Lee J, Lee SH, et al. Selective compromise of hypoplastic posterior communicating artery variants with aneurysms treatable by coil embolization: Clinical and radiologic outcomes. J Neurointervent Surg. 2019;11(4):373-9.
441. Chung J, Suh SH, Hong CK, Joo JY, Lim YC, Shin YS, et al. Preliminary experience with self-expanding closed-cell stent placement in small arteries less than 2 mm in diameter for the treatment of

© 2023 Volovici V et al. *JAMA Network Open.*

intracranial aneurysms. J Neurosurg. 2015;122(6):1503-10.
442. Chung KHC, Herwadkar A, Laitt R, Patel HC. Rate and clinical impact of intra-procedural complications during coil embolisation of ruptured small (3 mm or less) cerebral aneurysms. Clin Neurol Neurosurg. 2013;115(8):1356-61.
443. Clajus C, Sychra V, Strasilla C, Klisch J. Stent-assisted coil embolization of intracranial aneurysms using the Solitaire™ AB Neurovascular Remodeling Device: Initial and midterm follow-up results. Neuroradiology. 2013;55(5):629-38.
444. Cottier JP, Pasco A, Gallas S, Gabrillargues J, Cognard C, Drouineau J, et al. Utility of balloon-assisted guglielmi detachable coiling in the treatment of 49 cerebral aneurysms: A retrospective, multicenter study. Am J Neuroradiol. 2001;22(2):345-51.
445. Daglioglu E, Akmangit İ, Acik V, Alagoz F, Sayin B, Uckun OM, et al. The Experience of the Derivo® Embolisation Device in Intracranial Aneurysms. Turk Neurosurg. 2019.
446. Daou B, Starke RM, Chalouhi N, Tjoumakaris S, Khoury J, Hasan D, et al. The use of the pipeline embolization device in the management of recurrent previously coiled cerebral aneurysms. Neurosurgery. 2015;77(5):692-7.
447. Fischer S, Aguilar-Pérez M, Henkes E, Kurre W, Ganslandt O, Bäzner H, et al. Initial Experience with p64: A novel mechanically detachable flow diverter for the treatment of intracranial saccular sidewall aneurysms. Am J Neuroradiol. 2015;36(11):2082-9.
448. Foa Torres G, Roca F, Noguera A, Godes J, Petrocelli S, Aznar I, et al. Silk flow-diverter stent for the treatment of complex intracranial aneurysms: A one-year follow-up multicenter study. Intervent Neuroradiol. 2018;24(4):357-62.
449. Elias T, Ogungbo B, Connolly D, Gregson B, Mendelow AD, Gholkar A. Endovascular treatment of anterior communicating artery aneurysms: Results of clinical and radiological outcome in Newcastle. Br J Neurosurg. 2003;17(3):278-86.
450. Luo CB, Teng MMH, Chang FC, Chang CY. Endovascular embolization of ruptured cerebral aneurysms in patients older than 70 years. J Clin Neurosci. 2007;14(2):127-32.
451. Malatesta E, Nuzzi NP, Divenuto I, Fossaceca R, Lombardi M, Cerini P, et al. Endovascular treatment of intracranial aneurysms with flow-diverter stents: preliminary single-centre experience. Radiol Med. 2013;118(6):971-83.
452. Maldonado IL, Machi P, Costalat V, Mura T, Bonafe A. Neuroform stent-assisted coiling of unruptured aneurysms: Short- and midterm results from a single-center experience with 68 patients. Am J Neuroradiol. 2011;32(1):131-6.
453. Goddard AJP, Annesley-Williams D, Gholkar A. Endovascular management of unruptured intracranial aneurysms: Does outcome justify treatment? J Neurol Neurosurg Psychiatry. 2002;72(4):485-90.
454. Johnson AK, Munich SA, Heiferman DM, Lopes DK. Stent assisted embolization of 64 anterior communicating artery aneurysms. J Neurointerv Surg. 2013;5 Suppl 3:iii62-5.
455. Jia J, Lv X, Liu A, Wu Z, Li Y. Enterprise stent-assisted coiling of wide-necked intracranial aneurysms: Clinical and angiographic follow-up. Intervent Neuroradiol. 2012;18(4):426-31.
456. Hur CW, Choi CH, Cha SH, Lee TH, Jeong HW, Lee JI. Eleven year's single center experience of endovascular treatment of anterior communicating artery aneurysms: Focused on digital subtraction angiography follow-up results. J Korean Neurosurg Soc. 2015;58(3):184-91.
457. Hwang SK, Hwang G, Bang JS, Oh CW, Kwon OK. Endovascular Enterprise stent-assisted coil embolization for wide-necked unruptured intracranial aneurysms. J Clin Neurosci. 2013;20(9):1276-9.
458. Knap D, Gruszka W, Sieroń D, Gruszczyńska K, Zawadzki M, Zbroszczyk M, et al. Evaluation of endovascular embolization of cerebral aneurysms by hydrogel coils. Pol J Radiol. 2017;82:203-8.
459. Morais R, Mine B, Bruyère PJ, Naeije G, Lubicz B. Endovascular treatment of intracranial aneurysms with the p64 flow diverter stent: mid-term results in 35 patients with 41 intracranial aneurysms.

© 2023 Volovici V et al. *JAMA Network Open.*

Neuroradiology. 2017;59(3):263-9.
460. Herbreteau D, Bibi R, Narata AP, Janot K, Papagiannaki C, Soize S, et al. Are anatomic results influenced by web shape modification? analysis in a prospective, single-center series of 39 patients with aneurysms Treated with the WEB. Am J Neuroradiol. 2016;37(12):2280-6.
461. Kosty JA, Andaluz NO, Gozal YM, Krueger BM, Scoville J, Zuccarello M. Microsurgical treatment for unruptured intracranial aneurysms: a modern single surgeon series. Br J Neurosurg. 2019;33(3):322-7.
462. Ocal O, Peker A, Balci S, Arat A. Placement of a stent within a flow diverter improves aneurysm occlusion rates. Am J Neuroradiol. 2019;40(11):1932-8.
463. Fiorella D, Arthur A, Boulos A, Diaz O, Jabbour P, Pride L, et al. Final results of the US humanitarian device exemption study of the low-profile visualized intraluminal support (LVIS) device. J Neurointervent Surg. 2016;8(9):894-7.
464. Deshmukh VR, Kakarla UK, Figueiredo EG, Zabramski JM, Spetzler RF. Long-term clinical and angiographic follow-up of unclippable wrapped intracranial aneurysms. Neurosurgery. 2006;58(3):434-42; discussion -42.
465. Doerfler A, Wanke I, Goericke SL, Wiedemayer H, Engelhorn T, Gizewski ER, et al. Endovascular treatment of middle cerebral artery aneurysms with electrolytically detachable coils. Am J Neuroradiol. 2006;27(3):513-20.
466. Kim BM, Park SI, Kim DJ, Kim DI, Suh SH, Kwon TH, et al. Endovascular coil embolization of aneurysms with a branch incorporated into the sac. AJNR Am J Neuroradiol. 2010;31(1):145-51.
467. Nossek E, Chalif DJ, Chakraborty S, Lombardo K, Black KS, Setton A. Concurrent use of the Pipeline Embolization Device and coils for intracranial aneurysms: Technique, safety, and efficacy. J Neurosurg. 2015;122(4):904-11.
468. Korkmazer B, Kocak B, Islak C, Kocer N, Kizilkilic O. Long-term results of flow diversion in the treatment of intracranial aneurysms: a retrospective data analysis of a single center. Acta Neurochir. 2019;161(6):1165-73.
469. Luo CB, Chang FC, Lin CJ, Guo WY. A coil placement technique to treat intracranial aneurysm with incorporated artery. J Chin Med Assoc. 2018;81(3):255-61.
470. Nanda A, Javalkar V. Microneurosurgical management of ophthalmic segment of the internal carotid artery aneurysms: Single-surgeon operative experience from Louisiana State University, Shreveport. Neurosurgery. 2011;68(2):355-70.
471. Nelson PK, Lylyk P, Szikora I, Wetzel SG, Wanke I, Fiorella D. The pipeline embolization device for the intracranial treatment of aneurysms trial. Am J Neuroradiol. 2011;32(1):34-40.
472. Gonzalez N, Murayama Y, Nien YL, Martin N, Frazee J, Duckwiler G, et al. Treatment of Unruptured Aneurysms with GDCs: Clinical Experience with 247 Aneurysms. Am J Neuroradiol. 2004;25(4):577-83.
473. Gonzalez N, Sedrak M, Martin N, Vinuela F. Impact of anatomic features in the endovascular embolization of 181 anterior communicating artery aneurysms. Stroke. 2008;39(10):2776-82.
474. Fanning NF, Berentei Z, Brennan PR, Thornton J. HydroCoil as an adjuvant to bare platinum coil treatment of 100 cerebral aneurysms. Neuroradiology. 2007;49(2):139-48.
475. David CA, Vishteh AG, Spetzler RF, Lemole M, Lawton MT, Partovi S. Late angiographic follow-up review of surgically treated aneurysms. J Neurosurg. 1999;91(3):396-401.
476. Lee JY, Seo JH, Cho YD, Kang HS, Han MH. Endovascular treatment of wide-neck intracranial aneurysms using a microcatheter protective technique: Results and outcomes in 75 aneurysms. Am J Neuroradiol. 2011;32(5):917-22.
477. Kwon SC, Kwon OK, Ahn JS, Kwon BD, Kwon DH, Baik MW, et al. Endovascular coil embolization of unruptured intracranial aneurysms: A Korean multicenter study. Acta Neurochir. 2014;156(5):847-54.
478. Link TW, Boddu SR, Hammad HT, Knopman J, Lin N, Gobin P, et al. Endovascular treatment of middle cerebral artery aneurysms: A single center experience with a focus on thromboembolic complications. Intervent Neuroradiol. 2018;24(1):14-21.

© 2023 Volovici V et al. *JAMA Network Open.*

479. Kwon OK, Kim SH, Kwon BJ, Kang HS, Kim JH, Oh CW, et al. Endovascular treatment of wide-necked aneurysms by using two microcatheters: Techniques and outcomes in 25 patients. Am J Neuroradiol. 2005;26(4):894-900.
480. Pandey AS, Koebbe C, Rosenwasser RH, Veznedaroglu E. Endovascular coil embolization of ruptured and unruptured posterior circulation aneurysms: review of a 10-year experience. Neurosurgery. 2007;60(4):626-36; discussion 36-7.
481. Vallé JN, Pierot L, Mont'Alverne F, Turjman F, Bonafé A, Bracard S, et al. Unruptured intracranial aneurysms treated by three-dimensional coil embolization: Evaluation of the postoperative aneurysm occlusion volume. Neuroradiology. 2005;47(6):438-45.
482. Peluso JPP, van Rooij WJ, Sluzewski M, Beute GN. Superior cerebellar artery aneurysms: Incidence, clinical presentation and midterm outcome of endovascular treatment. Neuroradiology. 2007;49(9):747-51.
483. Pardo MI, Pumar JM, Blanco M, Vazquez F, Guimaraens L, Casasco A. Medium-term results using the Leo self-expanding stent in the treatment of complex intracranial aneurysms. Neuroradiol J. 2008;21(5):704-11.
484. Pagiola I, Mihalea C, Caroff J, Ikka L, Chalumeau V, Yasuda T, et al. Flow diversion treatment of aneurysms of the complex region of the anterior communicating artery: Which stent placement strategy should 'I' use? A single center experience. J Neurointervent Surg. 2019.
485. Mohammadian R, Asgari M, Sattarnezhad N, Mansourizadeh R, Mohammadian F, Shimia M, et al. Endovascular treatment of very small and very large ruptured aneurysms of the anterior cerebral circulation: A single-center experience. Cerebrovasc Dis. 2013;35(3):235-40.
486. Darwish B, Rajak S, Wickremesekera A, Hunn M, Balakrishnan V, Braithwaite D, et al. Clinical and angiographic outcome after guglielmi detachable coil embolization of intracranial aneurysms. ANZ J Surg. 2003;73(9):717-21.
487. Gallas S, Drouineau J, Gabrillargues J, Pasco A, Cognard C, Pierot L, et al. Feasibility, procedural morbidity and mortality, and long-term follow-up of endovascular treatment of 321 unruptured aneurysms. Am J Neuroradiol. 2008;29(1):63-8.
488. Jeon P, Kim BM, Kim DJ, Kim DIK, Park KY. Y-configuration double-stent-assisted coiling using two closed-cell stents for wide-neck basilar tip aneurysms. Acta Neurochir. 2014;156(9):1677-86.
489. Kim B, Jeon P, Kim K, Yang N, Kim S, Kim H, et al. Endovascular treatment of unruptured ophthalmic artery aneurysms: Clinical usefulness of the balloon occlusion test in predicting vision outcomes after coil embolization. J Neurointervent Surg. 2016;8(7):696-701.
490. Kim BM, Kim DI, Park SI, Kim DJ, Suh SH, Won YS. Coil embolization of unruptured middle cerebral artery aneurysms. Neurosurgery. 2011;68(2):346-53.
491. Gunnarsson T, Klurfan P, Terbrugge KG, Willinsky RA. Treatment of intracranial aneurysms with hydrogel coated expandable coils. Can J Neurol Sci. 2007;34(1):38-46.
492. Mitra D, Herwadkar A, Soh C, Gholkar A. Follow-up of intracranial aneurysms treated with matrix detachable coils: A single-center experience. Am J Neuroradiol. 2007;28(2):362-7.
493. Melake MS, Yamamoto M, Yoshida K, Oishi H, Arai H, Elwan M, et al. A retrospective clinical and angiographic study of the coiling outcome of ruptured intracranial aneurysms. J Clin Neurosci. 2010;17(3):328-33.
494. Linfante I, Akkawi NM, Perlow A, Andreone V, Wakhloo AK. Polyglycolide/polylactide-coated platinum coils for patients with ruptured and unruptured cerebral aneurysms: A single-center experience. Stroke. 2005;36(9):1948-53.
495. Matouk CC, Kaderali Z, TerBrugge KG, Willinsky RA. Long-term clinical and imaging follow-up of complex intracranial aneurysms treated by endovascular parent vessel occlusion. Am J Neuroradiol. 2012;33(10):1991-7.
496. Cheung NK, Chiu AHY, Cheung A, Wenderoth JD. Long term follow-up of bifurcation aneurysms

© 2023 Volovici V et al. *JAMA Network Open.*

treated with braided stent assisted coiling and complex T-and Y-stent constructs. J Neurointervent Surg. 2018;10(6):563-8.
497. Drazin D, Fennell VS, Gifford E, Lagman C, Atchaneeyasakul K, Edgell RC, et al. Safety and outcomes of simultaneous vasospasm and endovascular aneurysm treatment (SVAT) in subarachnoid hemorrhage. J Neurointerv Surg. 2017;9(5):482-5.
498. Oh K, Lim YC. Single-session Coil Embolization of Multiple Intracranial Aneurysms. J cerebrovasc endovasc neurosurg. 2013;15(3):184-90.
499. Griessenauer CJ, Ogilvy CS, Foreman PM, Chua MH, Harrigan MR, Stapleton CJ, et al. Pipeline Embolization Device for small paraophthalmic artery aneurysms with an emphasis on the anatomical relationship of ophthalmic artery origin and aneurysm. J Neurosurg. 2016;125(6):1352-9.
500. Griessenauer CJ, Piske RL, Baccin CE, Pereira BJA, Reddy AS, Thomas AJ, et al. Flow Diverters for Treatment of 160 Ophthalmic Segment Aneurysms: Evaluation of Safety and Efficacy in a Multicenter Cohort. Neurosurgery. 2017;80(5):726-32.
501. Murayama Y, Viñuela F, Duckwiler GR, Gobin YP, Guglielmi G. Embolization of incidental cerebral aneurysms by using the guglielmi detachable coil system. J Neurosurg. 1999;90(2):207-14.
502. Müller M, Brockmann C, Afat S, Nikoubashman O, Schubert GA, Reich A, et al. Temporary stent-assisted coil embolization as a treatment option for wide-neck aneurysms. Am J Neuroradiol. 2017;38(7):1372-6.
503. Gupta AK, Sonwalkar HA, Purkayastha S, Krishnamoorthy T, Bodhey NK, Kapilamoorthy TR, et al. Endovascular treatment of intracranial aneurysms: Long-term follow-up. Neuroradiol J. 2006;19(3):339-47.
504. Lubicz B, Kadou A, Morais R, Mine B. Leo stent for endovascular treatment of intracranial aneurysms: very long-term results in 50 patients with 52 aneurysms and literature review. Neuroradiology. 2017;59(3):271-6.
505. Hallout S. Surgical Treatment of Middle Cerebral Artery Aneurysms Without Using Indocyanine Green Videoangiography Assistance: Retrospective Monocentric Study of 263 Clipped Aneurysms. World Neurosurg. 2015;84(4):972-7.
506. Chalouhi N, Jabbour P, Starke RM, Tjoumakaris SI, Gonzalez LF, Witte S, et al. Endovascular treatment of proximal and distal posterior inferior cerebellar artery aneurysms. J Neurosurg. 2013;118(5):991-9.
507. Chen Y, Zhang Y, Chao YJ, Gao G, Ni CS, Fu XM, et al. Stent-assisted coiling embolization of middle cerebral artery trifurcation wide-necked aneurysms. Eur Rev Med Pharmacol Sci. 2017;21(19):4346-9.
508. Lv X, Jiang C, Li Y, Yang X, Wu Z. Endovascular treatment for pediatric intracranial aneurysms. Neuroradiology. 2009;51(11):749-54.
509. Lubicz B, Bandeira A, Bruneau M, Dewindt A, Balériaux D, De Witte O. Stenting is improving and stabilizing anatomical results of coiled intracranial aneurysms. Neuroradiology. 2009;51(6):419-25.
510. Primiani CT, Ren Z, Kan P, Hanel R, Pereira VM, Lui WM, et al. A2, M2, P2 aneurysms and beyond: Results of treatment with pipeline embolization device in 65 patients. J Neurointervent Surg. 2019;11(9):903-7.
511. Aghakhani N, Vaz G, David P, Parker F, Goffette P, Ozan A, et al. Surgical management of unruptured intracranial aneurysms that are inappropriate for endovascular treatment: Experience based on two academic centers. Neurosurgery. 2008;62(6):1227-34.
512. Grossberg JA, Hanel RA, Dabus G, Keigher K, Haussen DC, Sauvageau E, et al. Treatment of wide-necked aneurysms with the Low-profile Visualized Intraluminal Support (LVIS Jr) device: A multicenter experience. J Neurointervent Surg. 2017;9(11):1098-102.
513. Kadkhodayan Y, Almandoz JED, Fease JL, Scholz JM, Blem AM, Tran K, et al. Endovascular treatment of 346 middle cerebral artery aneurysms: Results of a 16-year single-center experience. Neurosurgery. 2015;76(1):54-60.

© 2023 Volovici V et al. *JAMA Network Open.*

514. Ko JK, Cho WH, Cha SH, Choi CH, Lee SW, Lee TH. Semi-jailing technique using a Neuroform3 stent for coiling of wide-necked intracranial aneurysms. J Korean Neurosurg Soc. 2017;60(2):146-54.
515. Kocur D, Zbroszczyk M, Przybylko N, Hofman M, Jamroz T, Baron J, et al. Stand-alone coil embolization of anterior communicating artery aneurysms: Efficacy and technical issues. Neuroradiol j. 2016;29(5):361-7.
516. Lukić S, Mijailović M, Marković Ž, Janković SM, Nikolić R. Embolization of ruptured intracranial aneurysms with detachable coils: Case series. Jap J Rad. 2011;29(2):92-7.
517. Moscato G, Cirillo L, Dallolio M, Princiotta C, Simonetti L, Leonardi M. Management of unruptured brain aneurysms: Retrospective analysis of a single centre experience. Neuroradiol J. 2013;26(3):315-9.
518. Griffin A, Reese V, Hüseyinoglu Z, Niedzwiecki D, Yang L, Cutler A, et al. Predictors of Clinical Outcome After Treatment of Intracranial Aneurysms with the Pipeline Embolization Device. World Neurosurg. 2019.
519. Gory B, Aguilar-Pérez M, Pomero E, Turjman F, Weber W, Fischer S, et al. One-year angiographic results after pCONus stent-assisted coiling of 40Wide-neck middle cerebral artery aneurysms. Neurosurgery. 2017;80(6):925-33.
520. Ota N, Matsukawa H, Noda K, Sato H, Hatano Y, Hashimoto A, et al. Evaluation of Microsurgery for Managing Giant or Complex Cerebral Aneurysms: A Retrospective Study. World Neurosurg. 2018;115:e190-e9.
521. Meyers PM, Coon AL, Kan PT, Wakhloo AK, Hanel RA. SCENT Trial. Stroke. 2019;50(6):1473-9.
522. D'Agostino SJ, Harrigan MR, Chalela JA, Nicholas JS, Deveikis SI, Jones TM, et al. Clinical experience with Matrix2 360° coils in the treatment of 100 intracranial aneurysms. Surg Neurol. 2009;72(1):41-7.
523. Chalouhi N, Jabbour P, Singhal S, Drueding R, Starke RM, Dalyai RT, et al. Stent-assisted coiling of intracranial aneurysms: Predictors of complications, recanalization, and outcome in 508 cases. Stroke. 2013;44(5):1348-53.
524. Martínez-Galdámez M, Romance A, Vega P, Vega A, Caniego JL, Paul L, et al. Pipeline endovascular device for the treatment of intracranial aneurysms at the level of the circle of Willis and beyond: Multicenter experience. J Neurointervent Surg. 2015;7(11):816-23.
525. Lylyk P, Cohen JE, Ceratto R, Ferrario A, Miranda C. Endovascular reconstruction of intracranial arteries by stent placement and combined techniques. J Neurosurg. 2002;97(6):1306-13.
526. Castro ER, Villoria F, Castaño C, Romance A, Mendez JC, Barrena R, et al. Spanish Registry for Embolization of Small Intracranial Aneurysms with Cerecyte Coils (SPAREC) study: Early experience and mid-term follow-up results. Intervent Neuroradiol. 2008;14(4):375-84.
527. Gao BL, Li TX, Li L, Xu GQ, Yang BW. Tiny Cerebral Aneurysms Can Be Treated Safely and Effectively with Low-Profile Visualized Intraluminal Support Stent-Assisted Coiling or Coiling Alone. World Neurosurg. 2018;113:e426-e30.
528. Ghinda D, Dos Santos MP, Sabri A, Iancu D, Lum C, Lesiuk HJ. Clinical and angiographic outcomes of stent-assisted coiling of intracranial aneurysms. Intervent Neuroradiol. 2015;21(2):146-54.
529. Guan N, Mu S, Wang L, Huo X, Jiang Y, Lv X, et al. Endovascular Treatment of 147 Cases of Cavernous Carotid Aneurysms: A Single-Center Experience. J STROKE CEREBROVASC DIS. 2016;25(8):1929-35.
530. Henkes H, Fischer S, Mariushi W, Weber W, Liebig T, Miloslavski E, et al. Angiographic and clinical results in 316 coil-treated basilar artery bifurcation aneurysms. J Neurosurg. 2005;103(6):990-9.
531. Jeon HJ, Kim BM, Kim DJ, Park KY, Kim JW, Kim DI. Combination of Multicatheter Plus Stent or Balloon for Treatment of Complex Aneurysms. AJNR Am J Neuroradiol. 2016;37(2):311-6.
532. Henkes H, Fischer S, Weber W, Miloslavski E, Felber S, Brew S, et al. Endovascular Coil Occlusion of 1811 Intracranial Aneurysms: Early Angiographic and Clinical Results. Neurosurgery. 2004;54(2):268-85.
533. Liu Y, Shi X, Kc KIS, Sun Y, Liu F, Qian H, et al. Microsurgical Treatment for Complex Basilar Artery

© 2023 Volovici V et al. *JAMA Network Open.*

Aneurysms with Long-Term Follow-Up in a Series of 35 Cases. World Neurosurg. 2018;111:e710-e21.
534. Li XD, Qin J, Xiao ZY, Feng Y, Chen JK. Solitaire AB stent-assisted coiling of wide-neck micro aneurysms. J Korean Neurosurg Soc. 2016;59(4):341-5.
535. Jeong HW, Seung WB. Outcomes of Stent-assisted Coil Embolization of Wide-necked Intracranial Aneurysms Using the SolitaireTM AB Neurovascular Remodeling Device. J cerebrovasc endovasc neurosurg. 2015;17(4):301-12.
536. Petr O, Brinjikji W, Cloft H, Kallmes DF, Lanzino G. Current trends and results of endovascular treatment of unruptured intracranial aneurysms at a single institution in the flow-diverter era. Am J Neuroradiol. 2016;37(6):1106-13.
537. Iosif C, Di Maria F, Sourour N, Degos V, Bonneville F, Biondi A, et al. Is a high initial World Federation of Neurosurgery (WFNS) grade really associated with a poor clinical outcome in elderly patients with ruptured intracranial aneurysms treated with coiling? J Neurointervent Surg. 2014;6(4):286-90.
538. Kähärä VJ, Seppänen SK, Kuurne T, Laasonen EM. Patient outcome after endovascular treatment of intracranial aneurysms with reference to microsurgical clipping. Acta Neurol Scand. 1999;99(5):284-90.
539. Kühn AL, Kan P, Henninger N, Srinivasan V, de Macedo Rodrigues K, Wakhloo AK, et al. Impact of age on cerebral aneurysm occlusion after flow diversion. J Clin Neurosci. 2019;65:23-7.
540. Fu JP, Liu L, Zhao H, Liu GF. The efficacy of enterprise stent-assisted coil embolization in the treatment of intracranial wide necked aneurysms by magnetic resonance angiography. Int J Clin Exp Med. 2018;11(4):4343-51.
541. Chalouhi N, Tjoumakaris S, Dumont AS, Gonzalez LF, Randazzo C, Gordon D, et al. Superior hypophyseal artery aneurysms have the lowest recurrence rate with endovascular therapy. Am J Neuroradiol. 2012;33(8):1502-6.
542. Debrun GM, Aletich VA, Kehrli P, Misra M, Ausman JI, Charbel F. Selection of cerebral aneurysms for treatment using Guglielmi detachable coils: The preliminary university of Illinois at Chicago experience. Neurosurgery. 1998;43(6):1281-97.
543. Liu Y, Wang F, Fu X, Liu Y, Zhang G, Xu K. Clinical and angiographic outcomes following endovascular treatment of very small (3 mm or smaller) intracranial aneurysm: A single-center experience. Medicine (Baltimore). 2017;96(37):e7457.
544. Deng D, Jin D, Zhou J, Chang Q, Qu K. Characteristics and endovascular treatment of intracranial vertebral artery aneurysms. Neurol India. 2011;59(6):833-8.
545. Dinç H, Halil Öztürk M, Sari A, Çakir E, Gazioğlu G, Kuzeyli K. Coil embolization in 481 ruptured intracranial aneurysms: Angiographic and clinical results. Diagn Intervention Radiol. 2013;19(2):165-72.
546. Duvuru S, Sae-Ngow T, Kato Y, Kawase T, Yamada Y, Tanaka R. Does Age Affects the Surgical Outcome in Patients with Unruptured Cerebral Aneurysms? A 2-Year Retrospective Study from a Single Center in Japan. Asian J Neurosurg. 2018;13(4):1108-11.
547. Fang C, Li MH, Zhu YQ, Tan HQ, Zhang PL, Xu HW, et al. The effectiveness and feasibility of endovascular coil embolization for very small cerebral aneurysms: mid- and long-term follow-up. Ann Vasc Surg. 2010;24(3):400-7.
548. Finitsis S, Anxionnat R, Lebedinsky A, Albuquerque PC, Clayton MF, Picard L, et al. Endovascular treatment of ACom intracranial aneurysms: Report on series of 280 patients. Intervent Neuroradiol. 2010;16(1):7-16.
549. Koźba-Gosztyła M, Czapiga B, Jarmundowicz W, Tomiałowicz Ł. Unruptured intracranial aneurysms: Surgery still safe as a treatment option. Adv Clin Exp Med. 2016;25(5):911-6.
550. Grasso G, Perra G. Surgical management of ruptured small cerebral aneurysm: Outcome and surgical notes. Surg Neurol Intl. 2015;6(1).
551. Ji W, Kang H, Liu A, Li Y, Feng X, Qian Z, et al. Stent-assisted coiling of very small wide-necked intracranial aneurysms: Complications, anatomical results and clinical outcomes. Neurol Neurochir Pol.

© 2023 Volovici V et al. *JAMA Network Open.*

2016;50(6):410-7.
552. Gao BL, Zhang XJ, Fan QY, Hao WL, Li CH, Yang ST. Long-term outcome of the enterprise stent in the treatment of wide-necked cerebral aneurysms. Acta Med Mediterr. 2018;34(1).
553. Liu A, Peng T, Qian Z, Li Y, Jiang C, Wu Z, et al. Enterprise stent-assisted coiling for wide-necked intracranial aneurysms during ultra-early (48hours) subarachnoid hemorrhage: A single-center experience in 59 consecutive patients. J Neuroradiol. 2015;42(5):298-303.
554. Ishida A, Matsuo S, Asakuno K, Nemoto A, Niimura K, Yoshimoto H, et al. Utility of crankshaft clips for middle cerebral artery aneurysms: A single-center experience of 150 cases. Surg Neurol Intl. 2016;7(19):S518-S22.
555. Kim ST, Jeong HW, Jeong YG, Heo YJ, Seo JH, Paeng SH. A Self-expanding Nitinol Stent (Enterprise) for the Treatment of Wide-necked Intracranial Aneurysms: Angiographic and Clinical Results in 40 Aneurysms. J cerebrovasc endovasc neurosurg. 2013;15(4):299-306.
556. Mordasini P, Schroth G, Guzman R, Barth A, Seiler RW, Remonda L. Endovascular treatment of posterior circulation cerebral aneurysms by using Guglielmi detachable coils: A 10-year single-center experience with special regard to technical development. Am J Neuroradiol. 2005;26(7):1732-8.
557. Kocur D, Zbroszczyk M, Przybylko N, Hofman M, Jamroz T, Baron J, et al. Stent-assisted embolization of wide-neck anterior communicating artery aneurysms: Review of consecutive 34 cases. Neurol Neurochir Pol. 2016;50(6):425-31.
558. Lempert TE, Malek AM, Halbach VV, Phatouros CC, Meyers PM, Dowd CF, et al. Endovascular treatment of ruptured posterior circulation cerebral aneurysms: Clinical and angiographic outcomes. Stroke. 2000;31(1):100-10.
559. Gupta AK, Sonwalkar H, Purkayastha S, Bodhey N. Endovascular treatment of ruptured intracranial aneurysms: Immediate result and long term follow up. J Int Med Sci Acad. 2007;20(1):25-31.
560. Lee DH, Arat A, Morsi H, Jou LD, Mawad ME. Embolization of cerebral aneurysms with spherically shaped detachable microcoils (micrusphere microcoil system): A single centre experience. Intervent Neuroradiol. 2009;15(1):29-36.
561. Jeon P, Kim BM, Kim DJ, Kim DI, Suh SH. Treatment of multiple intracranial aneurysms with 1-stage coiling. Am J Neuroradiol. 2014;35(6):1170-3.
562. Juszkat R, Nowak S, Kociemba W, Smól S, Blok T, Paprzycki W. Endovascular treatment of cerebral aneurysms using hydraulically detachable coils. Pol J Radiol. 2006;71(3):57-63.
563. Higashida RT, Cognard C, Bracard S. Initial Clinical experience with a new complex-shaped detachable platinum coil system for the treatment of intracranial cerebral aneurysms. The cordis trufill DCS detachable coil system. Intervent Neuroradiol. 2006;12(2):123-30.
564. Killer-Oberpfalzer M, Kocer N, Griessenauer CJ, Janssen H, Engelhorn T, Holtmannspötter M, et al. European multicenter study for the evaluation of a dual-layer flow-diverting stent for treatment of wide-neck intracranial aneurysms: The european flow-redirection intraluminal device study. Am J Neuroradiol. 2018;39(5):841-7.
565. Matano F, Tanikawa R, Kamiyama H, Ota N, Tsuboi T, Noda K, et al. Surgical Treatment of 127 Paraclinoid Aneurysms with Multifarious Strategy: Factors Related with Outcome. World Neurosurg. 2016;85:169-76.
566. Kuether TA, Nesbit GM, Barnwell SL. Clinical and angiographic outcomes, with treatment data, for patients with cerebral aneurysms treated with Guglielmi detachable coils: A single- center experience. Neurosurgery. 1998;43(5):1016-23.
567. Lubicz B, Morais R, Bruyère PJ, Ligot N, Mine B. Stent-assisted coiling of wide-neck bifurcation aneurysms with a branch incorporated in the aneurysm base: long-term follow-up in 49 patients with 53 aneurysms. Neuroradiology. 2017;59(6):619-24.
568. Iosif C, Mounayer C, Yavuz K, Saleme S, Geyik S, Cekirge HS, et al. Middle cerebral artery bifurcation aneurysms treated by extrasaccular flow diverters: Midterm angiographic evolution and clinical

outcome. Am J Neuroradiol. 2017;38(2):310-6.
569. Khaldi A, Fargen KM, Waldau B, Siddiqui AH, Hoh BL, Mack W, et al. The Orbit Galaxy XTRASOFT Coils: A Multicenter Study of Coil Safety and Efficacy in Both Ruptured and Unruptured Cerebral Aneurysms. J vasc interv radiol neurol. 2012;5(1):17-21.
570. Gao X, Liang G, Li Z, Qu H, Wei X. Stent-assisted coil embolization of wide-necked intracranial aneurysms using a semi-deployment technique: Angiographic and clinical outcomes in 31 consecutive patients. Intervent Neuroradiol. 2010;16(4):385-93.
571. Karanam LSP, Joseph S. Endovascular management of intracranial giant aneurysms: Experience on 25 patients. J Clin Diagn Res. 2012;6(6):1022-5.
572. Kaku Y, Yoshimura S, Hayashi K, Ueda T, Sakai N. Follow-up study on intra-aneurysmal embolization for unruptured cerebral aneurysms. Intervent Neuroradiol. 1999;5(SUPPL. 1):89-92.
573. Liang G, Gao X, Li Z, Wei X, Xue H. Neuroform stent-assisted coiling of intracranial aneurysms: A 5 year single-center experience and follow-up. Neurol Res. 2010;32(7):721-7.
574. Negoro M, Okamoto T, Miyachi S, Takahashi I, Fukui K, Nakabayashi K, et al. Intravascular treatment for cerebral aneurysms. Intervent Neuroradiol. 1998;4(SUPPL. 1):145-8.
575. Mu SQ, Yang XJ, Li YX, Zhang YP, Lü M, Wu ZX. Endovascular treatment of wide-necked intracranial aneurysms using of "remodeling technique" with the HyperForm balloon. Chin Med J. 2008;121(8):725-9.
576. Gory B, Klisch J, Bonafé A, Mounayer C, Beaujeux R, Moret J, et al. Solitaire AB stent-assisted coiling of wide-necked intracranial aneurysms: Mid-term results from the SOLARE study. Neurosurgery. 2014;75(3):215-9.
577. Murayama Y, Viñuela F, Duckwiler GR, Gobin YP, Guglielmi G. Endovascular treatment of incidental cerebral aneurysms: Report on 115 cases treated with Guglielmi detachable coils. Intervent Neuroradiol. 1999;5(SUPPL. 1):79-81.
578. Moret J, Pierot L, Boulin A, Castaings L, Rey A. Endovascular treatment of anterior communicating artery aneurysms using Guglielmi detachable coils. Neuroradiology. 1996;38(8):800-5.
579. Nakai Y, Sonobe M, Kato N, Okamoto S, Nakamura K, Sugita K. Endovascular treatment of ruptured anterior communicating artery aneurysms. Results and technical considerations. Intervent Neuroradiol. 2006;12(SUPPL. 1):61-6.
580. Juszkat R, Nowak S, Smól S, Kociemba W, Blok T, Zarzecka A. Leo stent for endovascular treatment of broad-necked and fusiform intracranial aneurysms. Intervent Neuroradiol. 2007;13(3):255-69.
581. Kim BM, Kim DJ, Jeon P, Yoon PH, Lee BH, Lee MS, et al. Endovascular Embolization of Intracranial Aneurysms Using Bare Platinum AxiumTM Detachable Coils: Immediate and Short-Term Follow-up Results from a Multicenter Registry. Neurointervention. 2012;7(2):85-92.
582. Lubicz B, Van Der Elst O, Collignon L, Mine B, Alghamdi F. Silk flow-diverter stent for the treatment of intracranial aneurysms: A series of 58 patients with emphasis on long-term results. Am J Neuroradiol. 2015;36(3):542-6.
583. Heller RS, Lawlor CM, Hedges ITR, Bababekov YJ, Safain MG, Malek AM. Neuro-ophthalmic effects of stenting across the ophthalmic artery origin in the treatment of intracranial aneurysms. J Neurosurg. 2014;121(1):18-23.
584. Gory B, Huot L, Riva R, Labeyrie PE, Levrier O, Lebedinsky A, et al. One-year efficacy and safety of the Trufill DCS Orbit and Orbit Galaxy detachable coils in the endovascular treatment of intracranial aneurysms: Results from the TRULINE study. Intervent Neuroradiol. 2017;23(5):485-91.
585. Li MH, Li YD, Tan HQ, Luo QY, Cheng YS. Treatment of distal internal carotid artery aneurysm with the willis covered stent: a prospective pilot study. Radiology. 2009;253(2):470-7.
586. Nagatani T, Shibuya M, Ooka K, Suzuki Y, Takayasu M, Yoshida J. Titanium aneurysm clips: Mechanical characteristics and clinical trial. Neurol Med -Chir. 1998;38(SUPPL.):39-44.
587. Panagiotopoulos V, Ladd SC, Gizewski E, Asgari S, Sandalcioglu EI, Forsting M, et al. Recovery of

ophthalmoplegia after endovascular treatment of intracranial aneurysms. Am J Neuroradiol. 2011;32(2):276-82.
588. Piano M, Valvassori L, Lozupone E, Pero G, Quilici L, Boccardi E. FRED Italian Registry: a multicenter experience with the flow re-direction endoluminal device for intracranial aneurysms. J Neurosurg. 2019:1-8.
589. Moon K, Albuquerque FC, Ducruet AF, Webster Crowley R, McDougall CG. Treatment of ophthalmic segment carotid aneurysms using the pipeline embolization device: Clinical and angiographic follow-up. Neurol Res. 2014;36(4):344-50.
590. Matsumaru Y, Sonobe M, Masuda R, Yasuda M, Hori E, Sugita K, et al. The embolization of ruptured aneurysms in acute stage with Guglielmi Detachable Coils. Intervent Neuroradiol. 1999;5(SUPPL. 1):191-3.
591. Liang B, Lesley WS, Robinson TM, Chen W, Benardete EA, Huang JH. Off-Label Application of Pipeline Embolization Device for Intracranial Aneurysms. 2019.
592. Qi W, Wang S, Zhao YL, Yang HB, Zhao JZ. Clinical characteristics and surgical treatment of patients with giant intracranial aneurysms. Chin Med J. 2008;121(12):1085-8.
593. Qin F, Li Z, Fang X, Zhao X, Liu J, Wu D, et al. Therapeutic effect of enterprise stent-assisted embolization for very small ruptured intracranial aneurysms. Medicine. 2017;96(34).
594. Krisht AF, Krayenbühl N, Sercl D, Bikmaz K, Kadri PAS. Results of microsurgical clipping of 50 high complexity basilar apex aneurysms. Neurosurgery. 2007;60(2):242-50.
595. Sorimachi T, Ito Y, Morita K, Jimbo Y, Nishino K, Sasaki O, et al. Long-term follow-up of intra-aneurysmal coil embolization for unruptured paraclinoid aneurysms. Neurol Res. 2012;34(9):864-70.
596. Turek G, Lewszuk A, Kochanowicz J, Lyson T, Zielinska-Turek J, Gorbacz K, et al. Early outcomes and perioperative complications of endovascular embolization in patients with aneurysmal SAH. Neurol Neurochir Pol. 2016;50(5):342-8.
597. Tang W, Feng H, Chen Z, Miu H, Pan J, Lin J, et al. Microsurgical treatment of ruptured intracranial aneurysm: A 120-case analysis. Acta Neurochir Suppl2011. p. 141-3.
598. Sonobe M, Nakai Y, Matsumaru Y, Sugita K. The embolization of ruptured aneurysms in acute stage. Intervent Neuroradiol. 2001;7(SUPPL. 1):53-6.
599. Rathore YS, Chandra PS, Kumar R, Singh M, Sharma MS, Suri A, et al. Monitored gradual occlusion of the internal carotid artery followed by ligation for giant internal carotid artery aneurysms. Neurol India. 2012;60(2):174-9.
600. Sluzewski M, Van Rooij WJ, Rinkel GJE, Wijnalda D. Endovascular treatment of ruptured intracranial aneurysms with detachable coils: Long-term clinical and serial angiographic results. Radiology. 2003;227(3):720-4.
601. Jafar JJ, Russell SM, Woo HH, Han PP, Spetzler RF, Sekhar LN, et al. Treatment of giant intracranial aneurysms with saphenous vein extracranial-to-intracranial bypass grafting: Indications, operative technique, and results in 29 patients. Neurosurgery. 2002;51(1):138-46.
602. Kaku Y, Yoshimura S, Kokuzawa J, Sakai N. Clinical and angiographic results of intra-aneurysmal embolization for cerebral aneurysms and histopathological findings in an aneurysm treated with GDC. Intervent Neuroradiol. 2003;9(SUPPL. 1):35-40.
603. Kallmes DF, Brinjikji W, Boccardi E, Ciceri E, Diaz O, Tawk R, et al. Aneurysm study of pipeline in an observational registry (ASPIRe). Intervent Neurol. 2016;5(1-2):89-99.
604. Watanabe D, Hashimoto T, Koyama S, Ohashi HT, Okada H, Ichimasu N, et al. Endovascular treatment of ruptured intracranial aneurysms in patients 70 years of age and older. Surg Neurol Intl. 2014;5(Supplement).
605. Yaltirik Bilgin E, Onal B, Emmez H, Akkan K, Ilgit E, Bilgin E, et al. Endovascular Treatment of Intracranial Anterior Circulation Aneurysms with Flow Diverters: A Single Centre Experience with mid and long-term results. 2017.

© 2023 Volovici V et al. *JAMA Network Open.*

606. Yang PF, Liu JM, Huang QH, Zhao WY, Hong B, Xu Y, et al. Preliminary experience and short-term follow-up results of treatment of wide-necked or fusiform cerebral aneurysms with a self-expanding, closed-cell, retractable stent. J Clin Neurosci. 2010;17(7):837-41.
607. Hayakawa M, Murayama Y, Duckwiler GR, Gobin YP, Guglielmi G, Viñuela F. Natural history of the neck remnant of a cerebral aneurysm treated with the Guglielmi detachable coil system. J Neurosurg. 2000;93(4):561-8.
608. Larson JJ, Tew JM, Jr., Tomsick TA, van Loveren HR. Treatment of aneurysms of the internal carotid artery by intravascular balloon occlusion: long-term follow-up of 58 patients. Neurosurgery. 1995;36(1):26-30; discussion
609. Liu P, Lv X, Li Y, Lv M. Stent-assisted coiling of ruptured wide-necked intracranial aneurysms: A single-center experience of 218 consecutive patients. Neurol India. 2016;64:S70-S7.
610. Liu P, Lv X, Li Y, Lv M. Endovascular treatment of A1 aneurysms of the anterior cerebral artery. Neurol India. 2016;64(4):694-700.
611. Gunia D, Ingorokva G, Ekvtimishvili E, Basiladze G. SPECIFICATIONS OF INTRACRANIAL SACCULAR ANEURYSMS, TREATED BY ENDOVASCULAR APPROACH USING DETACHABLE COILS. Georgian Med News. 2017(265):83-8.
612. Lubicz B, Van Der Elst O, Collignon L, Mine B, Alghamdi F. Silk flow-diverter stent for the treatment of intracranial aneurysms: A series of 58 patients with emphasis on long-term results. Am J Neuroradiol. 2015;36(3):542-6.
613. Vendrell JF, Menjot N, Costalat V, Hoa D, Moritz J, Brunel H, et al. Endovascular treatment of 174 middle cerebral artery aneurysms: Clinical outcome and radiologic results at long-term follow-up. Radiology. 2009;253(1):191-8.
614. Pereira-Filho AA, Pereira AG, Pereira-Filho NA, Lima LC, da Costa JC, Kraemer JL, et al. Long-term behavioral and cognitive outcomes following clipping for incidental unruptured intracranial aneurysms. Neuropsychology. 2014;28(1):75-83.
615. Yu SCH, Kwok CK, Cheng PW, Chan KY, Lau SS, Lui WM, et al. Intracranial aneurysms: Midterm outcome of pipeline embolization device - A prospective study in 143 patients with 178 aneurysms. Radiology. 2012;265(3):893-901.
616. Lv X, Jiang C, Li Y, Yang X, Zhang J, Wu Z. Treatment of giant intracranial aneurysms. Intervent Neuroradiol. 2009;15(2):135-44.
617. Adeeb N, Griessenauer CJ, Patel AS, Foreman PM, Baccin CE, Moore JM, et al. The Use of Single Stent-Assisted Coiling in Treatment of Bifurcation Aneurysms: A Multicenter Cohort Study With Proposal of a Scoring System to Predict Complete Occlusion. Neurosurgery. 2018;82(5):710-8.
618. Iosif C, Camilleri Y, Saleme S, Caire F, Yardin C, Ponomarjova S, et al. Diffusion-weighted imaging-detected ischemic lesions associated with flow-diverting stents in intracranial aneurysms: Safety, potential mechanisms, clinical outcome, and concerns. J Neurosurg. 2015;122(3):627-36.
619. Wilson SJ, Drackford R, Holt M, Azim A. A retrospective study of survivors of endovascular coiling for posterior and anterior aneurysms: Medical and patient perspectives. Medicine. 2015;94(32).
620. Jabbour P, Chalouhi N, Tjoumakaris S, Gonzalez LF, Dumont AS, Randazzo C, et al. The pipeline embolization device: Learning curve and predictors of complications and aneurysm obliteration. Neurosurgery. 2013;73(1):113-20.
621. Wang Q, Zhang W, Wang M, Li Z, Quan Z. Application and outcomes of endovascular treatment of coil embolization on ruptured intracranial aneurysms with daughter sacs. Int J Clin Exp Med. 2017;10(12):16476-83.
622. Zhu CY, Liu CX, Liu JX, Zhou T, Li CH, Yang ST, et al. Long-term angiographic follow-up of intracranial aneurysms treated with the intracranial neuroform stent reconstruction. Biomed Res. 2017;28(15):6700-5.
623. Matsukawa H, Kamiyama H, Tsuboi T, Noda K, Ota N, Miyata S, et al. Subarachnoid hemorrhage

© 2023 Volovici V et al. *JAMA Network Open.*

after surgical treatment of unruptured intracranial aneurysms. J Neurosurg. 2018;129(2):490-7.
624. Vallee JN, Aymard A, Vicaut E, Reis M, Merland JJ. Endovascular treatment of basilar tip aneurysms with Guglielmi detachable coils: predictors of immediate and long-term results with multivariate analysis 6-year experience. Radiology. 2003;226(3):867-79.
625. Abud DG, Nakiri GS, Abud TG, Carlotti Jr CG, Colli BO, Santos AC. Endovascular therapy for selected (most non-surgical) intracranial aneurysms in a brazilian university hospital. Arq Neuro-Psiquiatr. 2010;68(5):764-9.
626. Murias Quintana E, Vega Valdés P, Morales Deza E, Gil Garcia A, Cuellar H, Costilla García S, et al. Analysis of endovascular treatment of ruptured aneurysms of the middle cerebral artery compared to other anatomical locations. Intervent Neuroradiol. 2016;22(6):649-53.
627. Mori K, Wada K, Otani N, Tomiyama A, Toyooka T, Fujii K, et al. Validation of effectiveness of keyhole clipping in nonfrail elderly patients with unruptured intracranial aneurysms. J Neurosurg. 2017;127(6):1307-14.
628. Adeeb N, Moore JM, Wirtz M, Griessenauer CJ, Foreman PM, Shallwani H, et al. Predictors of incomplete occlusion following pipeline embolization of intracranial aneurysms: Is It less effective in older patients? Am J Neuroradiol. 2017;38(12):2295-300.
629. Zaidat OO, Castonguay AC, Rai AT, Badruddin A, Mack WJ, Alshekhlee AK, et al. TARGET Intracranial Aneurysm Coiling Prospective Multicenter Registry: Final Analysis of Peri-Procedural and Long-Term Safety and Efficacy Results. Front Neurol. 2019;10:737.
630. Lowe SR, Bhalla T, Tillman H, Chaudry MI, Turk AS, Turner RD, et al. A comparison of diffusion-weighted imaging abnormalities following balloon remodeling for aneurysm coil embolization in the ruptured vs unruptured setting. Neurosurgery. 2018;82(4):515-24.
631. Labeyrie MA, Lenck S, Bresson D, Desilles JP, Bisdorff A, Saint-Maurice JP, et al. Parent artery occlusion in large, giant, or fusiform aneurysms of the carotid siphon: clinical and imaging results. AJNR Am J Neuroradiol. 2015;36(1):140-5.
632. Juszkat R, Kram P, Stanisławska K, Jankowski R, Stachowska-Tomczak B, Nowak S, et al. Ten years of experience in endovascular treatment of ruptured aneurysms of the posterior inferior cerebellar artery. Intervent Neuroradiol. 2016;22(2):129-37.
633. Iosif C, Lecomte JC, Pedrolo-Silveira E, Mendes G, Martel MPB, Saleme S, et al. Evaluation of ischemic lesion prevalence after endovascular treatment of intracranial aneurysms, as documented by 3-T diffusion-weighted imaging: A 2-year, single-center cohort study. J Neurosurg. 2018;128(4):982-91.
634. Debrun GM, Aletich VA, Kehrli P, Misra M, Ausman JI, Charbel F, et al. Aneurysm geometry: An important criterion in selecting patients for Guglielmi detachable coiling. Neurol Med -Chir. 1998;38(SUPPL.):1-20.
635. Jo KI, Kim HR, Yeon JY, Hong SC, Kim JS. Treatment outcomes of surgical clipping for unruptured anterior circulation aneurysm—single institute experiences in the era of neurophysiologic monitoring and endovascular treatment. Neurosurg Rev. 2015;38(4):677-82.
636. Akgul E, Onan HB, Bİlgİn SS, Tahta A, Khanmammadov E, Gungoren FZ, et al. Flow Diverter Stents in the Treatment of Cerebral Aneurysms Less than 5 mm. Turk Neurosurg. 2020.
637. Baek JW, Huh CW, Heo YJ, Yoo MW, Kwon SC, Kwon OK, et al. Endovascular coiling of proximal middle cerebral artery aneurysms: is it safe and durable? Acta Neurochir. 2018;160(12):2411-8.
638. Duckwiler G, Viñuela F, Gobin P, Guglielmi G. GDC in ruptured versus unruptured aneurysms. Intervent Neuroradiol. 1999;5(SUPPL. 1):199-202.
639. Miyazawa N, Nukui H, Mitsuka S, Hosaka T, Kakizawa T, Nishigaya K, et al. Treatment of intradural paraclinoidal aneurysms. Neurol Med -Chir. 1999;39(11):727-34.
640. Lubicz B, Graca J, Levivier M, Lefranc F, Dewitte O, Pirotte B, et al. Endovascular treatment of middle cerebral artery aneurysms. Neurocrit Care. 2006;5(2):93-101.
641. Bradac GB, Bergui M, Stura G, Fontanella M, Daniele D, Gozzoli L, et al. Periprocedural morbidity

© 2023 Volovici V et al. *JAMA Network Open.*

and mortality by endovascular treatment of cerebral aneurysms with GDC: A retrospective 12-year experience of a single center. Neurosurg Rev. 2007;30(2):117-25.
642. Wallace AN, Samaniego E, Kayan Y, Derdeyn CP, Delgado Almandoz JE, Dandapat S, et al. Balloon-assisted coiling of cerebral aneurysms with the dual-lumen Scepter XC balloon catheter: Experience at two high-volume centers. Intervent Neuroradiol. 2019;25(4):414-8.
643. Spiotta AM, Miranpuri A, Hawk H, Chaudry M, Turk AS, Turner RD. Balloon remodeling for aneurysm coil embolization with the coaxial lumen Scepter C balloon catheter: Initial experience at a high volume center. J Neurointervent Surg. 2013;5(6):582-5.
644. Nishi H, Ishii A, Satow T, Iihara K, Sakai N, Ezura M, et al. Parent artery occlusion for unruptured cerebral aneurysms: Results of the Japanese registry of neuroendovascular therapy 3. Neurol Med -Chir. 2019;59(1):1-9.
645. Takemoto K, Tateshima S, Golshan A, Gonzalez N, Jahan R, Duckwiler G, et al. Endovascular treatment of pediatric intracranial aneurysms: A retrospective study of 35 aneurysms. J Neurointervent Surg. 2014;6(6):432-8.
646. Gunnarsson T, Tong FC, Klurfan P, Cawley CM, Dion JE. Angiographic and clinical outcomes in 200 consecutive patients with cerebral aneurysm treated with hydrogel-coated coils. Am J Neuroradiol. 2009;30(9):1657-64.
647. Van Rooij WJ, Keeren GJ, Peluso JPP, Sluzewski M. Clinical and angiographic results of coiling of 196 very small (≤ 3 mm) intracranial aneurysms. Am J Neuroradiol. 2009;30(4):835-9.
648. Shi X, Qian H, Singh KCKI, Zhang Y, Zhou Z, Sun Y, et al. Surgical management of vertebral and basilar artery aneurysms: A single center experience in 41 patients. Acta Neurochir. 2013;155(6):1087-93.
649. Pierot L, Spelle L, Vitry F. Immediate clinical outcome of patients harboring unruptured intracranial aneurysms treated by endovascular approach: Results of the ATENA study. Stroke. 2008;39(9):2497-504.
650. Norbäck O, Gál G, Johansson M, Solander S, Tovi M, Persson L, et al. The establishment of endovascular aneurysm coiling at a neurovascular unit: Report of experience during early years. Neuroradiology. 2005;47(2):144-52.
651. Moon K, Albuquerque FC, Ducruet AF, Crowley RW, McDougall CG. Balloon remodeling of complex anterior communicating artery aneurysms: technical considerations and complications. J Neurointerv Surg. 2015;7(6):418-24.
652. Mehta P, Kalyanpur T, Narsinghpura KS, Krishnan R, Raja D, Yadav M, et al. Outcomes of endovascular coiling in patients with intracranial aneurysms presenting with poor clinical and SAH grade. Neuroradiol J. 2011;24(5):669-76.
653. Lum C, Narayanam SB, Silva L, Shankar J, Bussiere M, Dos Santos MP, et al. Outcome in small aneurysms (<4 mm) treated by endovascular coiling. J Neurointervent Surg. 2012;4(3):196-8.
654. Lim YC, Kim BM, Suh SH, Jeon P, Kim SH, Ihn YK, et al. Reconstructive treatment of ruptured blood blister-like aneurysms with stent and coil. Neurosurgery. 2013;73(3):480-8.
655. Kang JH, Huh SK, Kim J, Park KY, Chung J. Subdural Fluid Collection After the Clipping of Unruptured Intracranial Aneurysms: Its Clinical Course and Significance. World Neurosurg. 2018;116:e266-e72.
656. Johansson M, Norbáck O, Gál G, Cesarini KG, Tovi M, Solander S, et al. Clinical outcome after endovascular coil embolization in elderly patients with subarachnoid hemorrhage. Neuroradiology. 2004;46(5):385-91.
657. Jia ZY, Song YS, Sheen JJ, Kim JG, Lee CW, Suh DC. Loop microcatheter technique for coil embolization of paraclinoid aneurysms. Acta Neurochir (Wien). 2018;160(9):1755-60.
658. Jankowitz BT, Aleu A, Lin R, Kostov D, Thomas AJ, Gupta R, et al. Endovascular Treatment of Atypical Posterior Circulation Aneurysms: Technical Results and Review of the Literature. J Neuroimaging. 2011;21(1):56-61.
659. Arthur AS, Molyneux A, Coon AL, Saatci I, Szikora I, Baltacioglu F, et al. The safety and

effectiveness of the Woven EndoBridge (WEB) system for the treatment of wide-necked bifurcation aneurysms: final 12-month results of the pivotal WEB Intrasaccular Therapy (WEB-IT) Study. J Neurointerv Surg. 2019;11(9):924-30.
660. Atasoy D, Kandasamy N, Hart J, Lynch J, Yang SH, Walsh D, et al. Outcome study of the pipeline embolization device with shield technology in unruptured aneurysms (PEDSU). Am J Neuroradiol. 2019;40(12):2094-101.
661. Iskandar A, Nepper-Rasmussen J. Endovascular treatment of very small intracranial aneurysms. Intervent Neuroradiol. 2011;17(3):299-305.
662. Byrne JV, Adams CBT, Kerr RSC, Molyneux AJ. Endosaccular treatment of inoperable intracranial aneurysms with platinum coils. BR J NEUROSURG. 1995;9(5):585-92.
663. Daglioglu E, Akmangit I, Acik V, Alagoz F, Sayin B, Uckun OM, et al. The Experience of the DerivoÂ® Embolisation Device in Intracranial Aneurysms. Turk Neurosurg. 2020;30(1):30-7.
664. Im SH, Han MH, Kwon OK, Kwon BJ, Kim SH, Kim JE, et al. Endovascular coil embolization of 435 small asymptomatic unruptured intracranial aneurysms: Procedural morbidity and patient outcome. Am J Neuroradiol. 2009;30(1):79-84.
665. Hwang JH, Roh HG, Chun YI, Kang HS, Choi JW, Moon WJ, et al. Endovascular coil embolization of very small intracranial aneurysms. Neuroradiology. 2011;53(5):349-57.
666. Hirota N, Musacchio M, Cardoso M, Villarejo F, Requelme C, Tournade A. Angiographic and clinical results after endovascular treatment for middle cerebral artery berry aneurysms. Neuroradiol J. 2007;20(1):89-101.
667. Gallas S, Januel AC, Pasco A, Drouineau J, Gabrillargues J, Gaston A, et al. Long-term follow-up of 1036 cerebral aneurysms treated by bare coils: A multicentric cohort treated between 1998 and 2003. Am J Neuroradiol. 2009;30(10):1986-92.
668. Goertz L, Smyk MA, Mpotsaris A, Borggrefe J, Dorn F, Liebig T, et al. Long-term Angiographic Results of the Low-profile Acandis Acclino Stent for Treatment of Intracranial Aneurysms: A Multicenter Study. Clin Neurorad. 2019.
669. AlMatter M, Aguilar Pérez M, Hellstern V, Mitrovic G, Ganslandt O, Bäzner H, et al. Flow Diversion for Treatment of Acutely Ruptured Intracranial Aneurysms: A Single Center Experience from 45 Consecutive Cases. Clin Neurorad. 2020;30(4):835-42.
670. AlMatter M, Henkes E, Sirakov A, Aguilar Pérez M, Hellstern V, Serna Candel C, et al. The p48 MW flow modulation device for treatment of unruptured, saccular intracranial aneurysms: a single center experience from 77 consecutive aneurysms. CVIR Endovasc. 2020;3(1).
671. Hagen F, Berlis A, Skalej M, Maurer CJ. Endovascular Treatment of Ruptured Middle Cerebral Artery Bifurcation Aneurysms. A Retrospective Observational Study of Short- and Long-Term Follow-Up. Cardiovasc Intervent Radiol. 2021.
672. Guimaraens L, Vivas E, Saldaña J, Llibre JC, Gil A, Balaguer E, et al. Efficacy and safety of the dual-layer flow-diverting stent (FRED) for the treatment of intracranial aneurysms. J Neurointervent Surg. 2020;12(5):521-5.
673. Griffin A, Lerner E, Zuchowski A, Zomorodi A, Gonzalez LF, Hauck EF. Flow diversion of fusiform intracranial aneurysms. Neurosurg Rev. 2020.
674. Gallas S, Pasco A, Cottier JP, Gabrillargues J, Drouineau J, Cognard C, et al. A multicenter study of 705 ruptured intracranial aneurysms treated with Guglielmi detachable coils. Am J Neuroradiol. 2005;26(7):1723-31.
675. Andrade-Barazarte H, Kivelev J, Goehre F, Jahromi BR, Hijazy F, Moliz N, et al. Contralateral Approach to Internal Carotid Artery Ophthalmic Segment Aneurysms: Angiographic Analysis and Surgical Results for 30 Patients. Neurosurgery. 2015;77(1):104-12.
676. Arslan G, Maus V, Weber W, Berlis A, Maurer C, Fischer S. Two-center experience with Neuroform Atlas stent-assisted coil occlusion of broad-based intracranial aneurysms. Neuroradiology. 2021.

677. Aguilar Perez M, Henkes E, Hellstern V, Serna Candel C, Wendl C, Bazner H, et al. Endovascular Treatment of Anterior Circulation Aneurysms With the p64 Flow Modulation Device: Mid- and Long-Term Results in 617 Aneurysms From a Single Center. 2021.
678. Aydin K, Balci S, Sencer S, Barburoglu M, Umutlu MR, Arat A. Y-stent-assisted coiling with low-profile neuroform atlas stents for endovascular treatment of wide-necked complex intracranial bifurcation aneurysms. Neurosurgery. 2020;87(4):744-53.
679. Baek JW, Jin SC, Kim JH, Yoo MW, Jeong HW, Seo JH, et al. Initial multicentre experience using the neuroform atlas stent for the treatment of un-ruptured saccular cerebral aneurysms. Br J Neurosurg. 2020;34(3):333-8.
680. Bhogal P, Ganslandt O, Bazner H, Henkes H, Aguilar Perez M. Treatment of Unruptured, Saccular, Anterior Choroidal Artery Aneurysms with Flow Diversion : A Single Centre Experience. Clin Neuroradiol. 2019;29(3):459-65.
681. Kwon O, Chung J. Outcomes of Stent-Assisted Coiling Using the Neuroform Atlas Stent in Unruptured Wide-Necked Intracranial Aneurysms. J Korean Neurosurg Soc. 2021;64(1):23-9.
682. Kim BM, Kim DI, Chung EC, Kim SY, Shin YS, Park SI, et al. Endovascular coil embolization for anterior choroidal artery aneurysms. Neuroradiology. 2008;50(3):251-7.
683. Patzig M, Forbrig R, Ertl L, Brückmann H, Fesl G. Intracranial Aneurysms Treated by Flow-Diverting Stents: Long-Term Follow-Up with Contrast-Enhanced Magnetic Resonance Angiography. Cardiovasc Intervent Radiol. 2017;40(11):1713-22.
684. Lee SW, Kwon HJ, Jeong EO, Koh HS, Kim KH, Choi SW, et al. Endovascular coil embolization for unruptured intracranial aneurysms in patients over 80 years of age. J cerebrovasc endovasc neurosurg. 2020;22(4):237-44.
685. Bradač GB, Bergui M, Fontanella M. Endovascular treatment of cerebral aneurysms in elderly patients. Neuroradiology. 2005;47(12):938-41.
686. Daniel B, Henrik S, Ioannis T, Veit R, Marios-Nikos P. SMART coils for intracranial aneurysm repair - A single center experience. BMC Neurol. 2020;20(1).
687. Maurer C, König I, Berlis A, Weber W, Fischer S. Two-center experience in the endovascular treatment of intracranial aneurysms using the woven endobridge 17 device including midterm follow-up results: A retrospective analysis. Am J Neuroradiol. 2019;40(9):1517-22.
688. Binh NT, Luu VD, Thong PM, Cuong NN, Anh NQ, Tuan TA, et al. Flow diverter stent for treatment of cerebral aneurysms: A report of 130 patients with 134 aneurysms. Heliyon. 2020;6(2):e03356.
689. Wang C, Zhao R, Chang X, Li Q, Fang Y, Hong B, et al. Feasibility and midterm outcomes of endovascular embolization for true posterior communicating artery aneurysms. Neuroradiology. 2019;61(10):1191-8.
690. Weir RU, Marcellus ML, Do HM, Steinberg GK, Marks MP. Aneurysmal subarachnoid hemorrhage in patients with Hunt and Hess grade 4 or 5: treatment using the Guglielmi detachable coil system. AJNR Am J Neuroradiol. 2003;24(4):585-90.
691. Teo M, Martin S, Ponweera A, Macey A, Suttner N, Brown J, et al. Results of surgical clipping in a neurointerventional dominant department. Br J Neurosurg. 2015;29(6):792-8.
692. Strauss I, Maimon S. Silk flow diverter in the treatment of complex intracranial aneurysms: a single-center experience with 60 patients. Acta Neurochir. 2016;158(2):247-54.
693. Shankar JJS, Tampieri D, Iancu D, Cortes M, Agid R, Krings T, et al. SILK flow diverter for complex intracranial aneurysms: A Canadian registry. J Neurointervent Surg. 2016;8(3):273-8.
694. Rodríguez-Hernández A, Sughrue ME, Akhavan S, Habdank-Kolaczkowski J, Lawton MT. Current management of middle cerebral artery aneurysms: Surgical results with a clip first policy. Neurosurgery. 2013;72(3):415-27.
695. Kim SM, Lee HG, Choi BS, Kim JS, Lee SJ, Kim HY, et al. Recurrence of Small Cerebral Aneurysms (< 4 mm) Treated Endovascularly Using Target NanoTM Coils. J cerebrovasc endovasc neurosurg.

© 2023 Volovici V et al. *JAMA Network Open.*

2018;20(2):106-11.
696. Choi JY, Choi CH, Ko JK, Lee JI, Huh CW, Lee TH. Feasibility and efficacy of coil embolization for middle cerebral artery aneurysms. Yeungnam univ j med. 2019;36(3):208-18.
697. Ioannidis I, Lalloo S, Corkill R, Kuker W, Byrne JV. Endovascular treatment of very small intracranial aneurysms: Clinical article. J Neurosurg. 2010;112(3):551-6.
698. Zhou Y, Peng Q, Wu X, Zhang Y, Liu J, Yang X, et al. Endovascular Treatment of Tiny Aneurysms With Low-Profile Visualized Intraluminal Support Devices Using a "Compressed" Stent Technique. Front Neurol. 2020;11:610126.
699. Zhou Y, Duan G, Zhang X, Yang PF, Fang YB, Li Q, et al. Outcome and Prognostic Factors of Ruptured Middle Cerebral Artery Aneurysms Treated via Endovascular Approach: A Single-Center 11-Year Experience. World Neurosurg. 2020;133:e187-e96.
700. Jiang T, Yan L, Chen M, Cao J, Shen Y, Ren Z. Lateral supraorbital approach for the surgical clipping of ruptured tiny anterior circulation aneurysms. J Coll Phys Surg Pak. 2020;30(5):523-6.
701. Zheng J, Guo Z, Xu R, He Z, Sun X. Microsurgical Treatment for Patients with Fenestrated Anterior Communicating Artery Aneurysms. World Neurosurg. 2019;125:e807-e11.
702. Gudelj M, Bruyère PJ, Tebache M, Collignon L, Lubicz B. Endovascular treatment of intracranial aneurysms: Initial experience in a low-volume center. J Belg Soc Radiol. 2020;104(1).
703. Cui YF, Xu H, Liu HT, Wang Y. Clinical application of Solitaire AB stents in the embolization of intracranial aneurysms. Eur Rev Med Pharmacol Sci. 2015;19(7):1227-33.
704. Zhao J, Wang S, Zhao Y, Sui D, Zhang Y, Tang J, et al. Microneurosurgical management of carotid-ophthalmic aneurysms. J Clin Neurosci. 2006;13(3):330-3.
705. Linfante I, Andreone V, Ravelo N, Starosciak AK, Arif B, Shallwani H, et al. Endovascular Treatment of Giant Intracranial Aneurysms. Cureus. 2020;12(5):e8290.
706. Zhang Y, Tian Z, Zhu W, Liu J, Wang Y, Wang K, et al. Endovascular treatment of bilateral intracranial vertebral artery aneurysms: an algorithm based on a 10-year neurointerventional experience. Stroke Vasc Neurol. 2020;5(3):291-301.
707. Zhang G, Liu Y, Liu Y, Wang M, Li K, Wang F. Safety and efficacy of complete versus near-complete coiling in treatment of intracranial aneurysms. J Intervent Med. 2020;3(3):136-41.
708. Zaidat OO, Hanel RA, Sauvageau EA, Aghaebrahim A, Lin E, Jadhav AP, et al. Pivotal Trial of the Neuroform Atlas Stent for Treatment of Anterior Circulation Aneurysms: One-Year Outcomes. Stroke. 2020:2087-94.
709. Yue J, Xie Y, Zhang X, Jiang Y, Chen W, Ma Y, et al. The safety and outcomes of acutely ruptured intracranial aneurysms with incomplete occlusion after coiling: a case-control study. BMC Neurol. 2020;20(1).
710. Youssef PP, Dornbos Iii D, Peterson J, Sweid A, Zakeri A, Nimjee SM, et al. Woven EndoBridge (WEB) device in the treatment of ruptured aneurysms. 2020.
711. Yeomans J, Sandu L, Sastry A. Pipeline Flex embolisation device with Shield Technology for the treatment of patients with intracranial aneurysms: periprocedural and 6 month outcomes. Neuroradiol J. 2020.
712. Yan Y, Zeng Z, Wu Y, Xiong J, Zhao K, Hong B, et al. The use of single low-profile visualized intraluminal support stent-assisted coiling in the treatment of middle cerebral artery bifurcation unruptured wide-necked aneurysm. Intervent Neuroradiol. 2020.
713. Yan P, Zhang Y, Ma C, Liang F, Zhu H, Jiang C. Application of the Willis Covered Stent in the treatment of intracranial unruptured aneurysms in internal carotid artery: A retrospective single-center experience. J Clin Neurosci. 2020;78:222-7.
714. Caragliano AA, Papa R, Pitrone A, Limbucci N, Nappini S, Ruggiero M, et al. The low-profile Neuroform Atlas stent in the treatment of wide-necked intracranial aneurysms – immediate and midterm results: An Italian multicenter registry. J Neuroradiol. 2020;47(6):421-7.

© 2023 Volovici V et al. *JAMA Network Open.*

715. Xue G, Zuo Q, Duan G, Zhang X, Zhao R, Li Q, et al. Dual Stent-Assisted Coil Embolization for Intracranial Wide-Necked Bifurcation Aneurysms: A Single-Center Experience and a Systematic Review and Meta-Analysis. World Neurosurg. 2019;126:e295-e313.
716. Wongsuriyanan S, Sriamornrattanakul K. Interhemispheric Approach with Early A1 Exposure for Clipping Anterior Communicating Artery Aneurysms: Operative Techniques and Outcomes. World Neurosurg. 2020;138:e579-e90.
717. Winkler EA, Lu A, Burkhardt JK, Rutledge WC, Yue JK, Birk HS, et al. Microsurgical Clipping of Anterior Choroidal Artery Aneurysms: A Systematic Approach to Reducing Ischemic Complications in an Experience with 146 Patients. Oper Neurosurg. 2019;17(4):413-23.
718. Weinberg JH, Sweid A, Asada A, Abbas R, Joffe D, El Naamani K, et al. Coil Embolization of Wide-Neck Bifurcation Aneurysms via Shouldering and Framing: A Safe Alternative to Conventional Techniques. World Neurosurg. 2020;139:e800-e6.
719. Waqas M, Vakharia K, Dossani RH, Rajah GB, Tso MK, Gong AD, et al. Transradial access for flow diversion of intracranial aneurysms: Case series. Interv. 2020;neuroradiol..1591019920938961.
720. Wang X, Xing H, Cai J, Jin D, Chen Y, Cui Y, et al. The safety and efficacy of the LVIS stent for the treatment of ruptured intracranial aneurysms within 24hours: A multicenter retrospective study. Clin Neurol Neurosurg. 2020;197.
721. Wang JW, Li CH, Tian YY, Li XY, Liu JF, Li H, et al. Safety and efficacy of endovascular treatment of ruptured tiny cerebral aneurysms compared with ruptured larger aneurysms. Intervent Neuroradiol. 2020;26(3):283-90.
722. Van Rooij WJJ, Sluzewski M, Metz NH, Nijssen PCG, Wijnalda D, Rinkel GJE, et al. Carotid balloon occlusion for large and giant aneurysms: Evaluation of a new test occlusion protocol. Neurosurgery. 2000;47(1):116-22.
723. Van Rooij WJ, Sluzewski M, Beute GN. Internal carotid bifurcation aneurysms: Frequency, angiographic anatomy and results of coiling in 50 aneurysms. Neuroradiology. 2008;50(7):583-7.
724. Sato H, Haraguchi K, Takahashi Y, Ohtaki S, Shimizu T, Matsuura N, et al. Endovascular coil embolization of proximal middle cerebral artery aneurysms has better outcomes than other middle cerebral artery aneurysms: A retrospective study. Intervent Neuroradiol. 2020;26(3):268-74.
725. Vargas SA, Herrera DA, Cornejo JW. [Endovascular coil occlusion of 473 intracranial aneurysms: angiographic and clinical results]. Biomedica (Bogota). 2010;30(4):567-76.
726. Ulutas M, Çinar K, Dogan I, Secer M, Isik S, Aksoy K. Lateral transorbital approach: An alternative microsurgical route for supratentorial cerebral aneurysms. J Neurosurg. 2021;134(1):72-83.
727. Trivelato FP, Wajnberg E, Rezende MTS, Ulhôa AC, Piske RL, Abud TG, et al. Safety and Effectiveness of the Pipeline Flex Embolization Device with Shield Technology for the Treatment of Intracranial Aneurysms: Midterm Results from a Multicenter Study. Neurosurgery. 2020;87(1):104-11.
728. Tjahjadi M, Niemela M, Kivelev J, Serrone J, Maekawa H, Jahromi BR, et al. Presigmoid Approach to Vertebrobasilar Artery Aneurysms: A Series of 31 Patients and Review of the Literature. World Neurosurg. 2016;92:313-22.
729. Tian Z, Liu J, Zhang Y, Zhang Y, Zhang X, Zhang H, et al. Risk Factors of Angiographic Recurrence After Endovascular Coil Embolization of Intracranial Saccular Aneurysms: A Retrospective Study Using a Multicenter Database. Front Neurol. 2020;11.
730. Thavara BD, Yamada Y, Joshi G, Tanaka R, Miyatani K, Devareddy G, et al. Analysis of the Surgical Outcome of Unruptured Intracranial Saccular Aneurysms in Octogenarians (80-89 Years). Asian J Neurosurg. 2020;15(3):640-3.
731. Taschner CA, Stracke CP, Dorn F, Kadziolka KB, Kreiser K, Solymosi L, et al. Derivo embolization device in the treatment of unruptured intracranial aneurysms: a prospective multicenter study. J Neurointerv Surg. 2020.
732. Srinivasan VM, Srivatsan A, Spiotta AM, Hendricks BK, Ducruet AF, Albuquerque FC, et al. Early

© 2023 Volovici V et al. *JAMA Network Open.*

postmarket results with PulseRider for treatment of wide-necked intracranial aneurysms: A multicenter experience. J Neurosurg. 2020;133(6):1756-65.
733. Bhogal P, Chudyk J, Bleise C, Lylyk I, Perez N, Henkes H, et al. Treatment of Unruptured, Tandem Aneurysms of the ICA with a Single Flow Diverter. Clin Neuroradiol. 2019;29(4):725-31.
734. Song Y, Sheen JJ, Kim JG, Lee SH, Cho SH, Park JC, et al. Alpha Stent for Coiling of Unruptured, Wide-Necked, Distal Internal Carotid Artery Aneurysms: Safety and Effectiveness at 6 Months. Korean J Radiol. 2020;21(2):228-35.
735. Sirakov A, Minkin K, Penkov M, Ninov K, Karakostov V, Sirakov S. Comaneci-assisted coiling as a treatment option for acutely ruptured wide neck cerebral aneurysm: Case series of 118 patients. Neurosurgery. 2020;87(6):1148-56.
736. Sharma RK, Kumar A, Yamada Y, Tanaka R, Sharma S, Miyatani K, et al. Institutional Experience of Microsurgical Management in Posterior Circulation Aneurysm. Asian J Neurosurg. 2020;15(3):484-93.
737. Shanno GB, Armonda RA, Benitez RP, Rosenwasser RH. Assessment of acutely unsuccessful attempts at detachable coiling in intracranial aneurysms. Neurosurgery. 2001;48(5):1066-74.
738. Schob S, Kläver M, Richter C, Scherlach C, Maybaum J, Mucha S, et al. Single-Center Experience With the Bare p48MW Low-Profile Flow Diverter and Its Hydrophilically Covered Version for Treatment of Bifurcation Aneurysms in Distal Segments of the Anterior and Posterior Circulation. Front Neurol. 2020;11.
739. Satow T, Ikeda G, Takahashi JC, Iihara K, Sakai N. Coil embolization for unruptured intracranial aneurysms at the dawn of stent era: Results of the Japanese registry of neuroendovascular therapy (JR-NET) 3. Neurol Med -Chir. 2020;60(2):55-65.
740. Sato K, Matsumoto Y, Kanoke A, Ito A, Fujimura M, Tominaga T. Y-Configuration Stenting for Coil Embolization of Complex Intracranial Aneurysms: Distinguishing Between Use of Crossing-Y and Kissing-Y. World Neurosurg. 2020.
741. Sai Kiran NA, Raj V, Sivaraju L, Vidyasagar K, Mohan D, Hegde AS. Outcome of Microsurgical Clipping for Multiple Versus Single Intracranial Aneurysms: A Single-Institution Retrospective Comparative Cohort Study. World Neurosurg. 2020;143:e590-e603.
742. Russo R, Bradac GB, Castellan L, Gallesio I, Garbossa D, Iannucci G, et al. Neuroform Atlas stent-assisted coiling of ruptured intracranial aneurysms: A multicenter study. J Neuroradiol. 2020.
743. Ross IB, Weill A, Piotin M, Moret J. Endovascular treatment of distally located giant aneurysms. Neurosurgery. 2008;62(6 SUPPL.):SHC1354-SHC9.
744. Ross IB, Dhillon GS. Balloon assistance as a routine adjunct to the endovascular treatment of cerebral aneurysms. Surg Neurol. 2006;66(6):593-601.
745. Rodríguez-Hernández A, Zador Z, Rodríguez-Mena R, Lawton MT. Distal aneurysms of intracranial arteries: Application of numerical nomenclature, predilection for cerebellar arteries, and results of surgical management. World Neurosurg. 2013;80(1-2):103-12.
746. Rice H, Martínez Galdámez M, Holtmannspötter M, Spelle L, Lagios K, Ruggiero M, et al. Periprocedural to 1-year safety and efficacy outcomes with the Pipeline Embolization Device with Shield technology for intracranial aneurysms: A prospective, post-market, multi-center study. J Neurointervent Surg. 2020;12(11):1107-12.
747. Renowden SA, Beneš V, Bradley M, Molyneux AJ. Detachable coil embolisation of ruptured intracranial aneurysms: A single center study, a decade experience. Clin Neurol Neurosurg. 2009;111(2):179-88.
748. Pop R, Harsan O, Martin I, Mihoc D, Richter JS, Manisor M, et al. Balloon-assisted coiling of intracranial aneurysms using the Eclipse 2L double lumen balloon. Intervent Neuroradiol. 2020;26(3):291-9.
749. Poncyljusz W, Zwarzany Ł, Limanówka B, Zbroszczyk M, Banach M, Bereza S, et al. Stent-assisted coiling of unruptured MCA aneurysms using the LVIS Jr. Device: a multicenter registry. J Clin Med. 2020;9(10):1-10.

© 2023 Volovici V et al. *JAMA Network Open.*

750. Poncyljusz W, Kubiak K. Initial experience with LVIS EVO stents for the treatment of intracranial aneurysms. J Clin Med. 2020;9(12):1-9.
751. Pierot L, Soize S, Cappucci M, Manceau PF, Riva R, Eker OF. Surface-modified flow diverter p48-MW-HPC: Preliminary clinical experience in 28 patients treated in two centers. J Neuroradiol. 2020.
752. Pierot L, Barbe C, Herbreteau D, Gauvrit JY, Januel AC, Bala F, et al. Immediate post-operative aneurysm occlusion after endovascular treatment of intracranial aneurysms with coiling or balloon-assisted coiling in a prospective multicenter cohort of 1189 patients: Analysis of Recanalization after Endovascular Treatment of intracranial Aneurysm (ARETA) Study. J Neurointervent Surg. 2020.
753. Petrov A, Rentsenkhuu G, Nota B, Ganzorig E, Regzengombo B, Jagusch S, et al. Initial experience with the novel p64MW HPC flow diverter from a cohort study in unruptured anterior circulation aneurysms under dual antiplatelet medication. Intervent Neuroradiol. 2020.
754. Peng F, Feng X, Tong X, Zhang B, Wang L, Guo E, et al. Endovascular Treatment of Small Ruptured Intracranial Aneurysms (<5 mm): Long-term Clinical and Angiographic Outcomes and Related Predictors. Clin Neurorad. 2019.
755. Pang J, Zhao C, Zhang A, Pang W, Ning X. Impact of different surgical methods for endovascular embolization of intracranial wide-necked aneurysms on patient prognosis and cognitive function. Int J Clin Exp Med. 2020;13(5):3630-6.
756. Pasqualin A, Meneghelli P, Cozzi F, Chioffi F. Outcome After Surgical Treatment of Paraclinoid Carotid Aneurysms. Acta Neurochir Suppl. 2016;123:33-9.
757. Van Lanen RHGJ, Jacobi-Postma LAA, Veersema TJ, Teernstra OPM, Dings JTA. Clinical and Radiological Outcomes of Intracranial Aneurysm Clipping Aided by Transit Time Flowmetry. World Neurosurg. 2020;136:e660-e70.
758. Tamrakar K, Karki B, Duan CZ, Li XF, Zhang X. Efficacy of endovascular therapy for direct occlusion of intracranial aneurysms. J Nepal Med Assoc. 2011;51(3):109-15.
759. Otani N, Mori K, Wada K, Tomiyama A, Toyooka T, Takeuchi S, et al. Limited Indications for Clipping Surgery of Paraclinoid Aneurysm Based on Long-Term Visual Morbidity. World Neurosurg. 2020;134:e153-e61.
760. Orru E, Rice H, De Villiers L, Klostranec JM, Wakhloo AK, Coon AL, et al. First clinical experience with the new Surpass Evolve flow diverter: technical and clinical considerations. J Neurointerv Surg. 2020;12(10):974-80.
761. Oğuz Ş, Tabakci ÖN, Uysal E, Bulut E, Dinç H. Pipeline flex embolization device (Ped flex) for the treatment of intracranial aneurysms: Periprocedural outcomes and first-year angiographic results. Turk J Med Sci. 2019;49(6):1640-6.
762. Nussbaum ES, Touchette JC, Madison MT, Goddard JK, Lassig JP, Nussbaum LA. Microsurgical treatment of unruptured anterior communicating artery aneurysms: Approaches and outcomes in a large contemporary series and review of the literature. Oper Neurosurg. 2020;19(6):678-90.
763. Nurminen V, Raj R, Numminen J, Kivisaari R, Niemelä M, Lehecka M. Flow diversion for internal carotid artery aneurysms: Impact of complex aneurysm features and overview of outcome. Clin Neurol Neurosurg. 2020;193.
764. Nanda A, Sonig A, Banerjee AD, Javalkar VK. Microsurgical management of basilar artery apex aneurysms: a single surgeon's experience from Louisiana State University, Shreveport. World Neurosurg. 2014;82(1-2):118-29.
765. Nanda A, Ambekar S, Sharma M. Surgical management of middle cerebral artery aneurysms. J La State Med Soc. 2014;166(4):160-7.
766. Munich SA, Vakharia K, McPheeters MJ, Tso MK, Waqas M, Snyder KV, et al. Make Clipping Great Again: Microsurgery for Cerebral Aneurysms by Dual-Trained Neurosurgeons. World Neurosurg. 2020;137:e454-e61.
767. Aydin K, Stracke CP, Barburoglu M, Yamac E, Berdikhojayev M, Sencer S, et al. Long-term

outcomes of wide-necked intracranial bifurcation aneurysms treated with T-stent-assisted coiling. J Neurosurg. 2021;134(1):39-48.
768. Moubark M, Allah AEKA, Yosef H, Abdel-Tawab M, Panos P, Othman M. Flow diverter devices in the treatment of posterior communicating artery aneurysms: mid-term clinical and radiological outcomes. Egypt J Radiol Nucl Med. 2020;51(1).
769. Michelozzi C, Darcourt J, Guenego A, Januel AC, Tall P, Gawlitza M, et al. Flow diversion treatment of complex bifurcation aneurysms beyond the circle of Willis: Complications, aneurysm sac occlusion, reabsorption, recurrence, and jailed branch modification at follow-up. J Neurosurg. 2019;131(6):1751-62.
770. McAvoy MB, Cappuzzo JM, Stapleton CJ, Koch MJ, Raymond SB, Torok CM, et al. Long-term follow-up results of the SMART coil in the endovascular treatment of intracranial aneurysms. Intervent Neuroradiol. 2020.
771. Maus V, Weber W, Berlis A, Maurer C, Fischer S. Initial Experience with Surpass Evolve Flow Diverter in the Treatment of Intracranial Aneurysms. Clin Neurorad. 2020.
772. Mascitelli JR, Levitt MR, Griessenauer CJ, Kim LJ, Gross B, Abla A, et al. Transcirculation approach for stent-assisted coiling of intracranial aneurysms: a multicenter study. 2020.
773. Lv X, Lv M, Li Y, Yang X, Jiang C, Wu Z. Endovascular treatment of ruptured and unruptured vertebral artery aneurysms. Neuroradiol J. 2011;24(5):677-86.
774. Lv X, Li Y, Yang X, Jiang C, Wu Z. Results of endovascular treatment for intracranial wide-necked saccular and dissecting aneurysms using the Enterprise stent: A single center experience. Eur J Radiol. 2012;81(6):1179-83.
775. Lv X, Jiang C, Wu Z, Jiang W, Wang G. Complex cerebral aneurysms: intra-luminal reconstruction using Pipeline flow-diverting stent and the obliteration mechanism. Neuroradiol J. 2020;33(2):91-7.
776. Lv X, Jiang C, Liang S. Small ruptured and unruptured complex cerebral aneurysms: Single center experience of low-profile visualized intraluminal support stent. J Neurorestoratology. 2019;7(4):235-41.
777. Kim JH, Choi CH, Lee JI, Lee TH, Ko JK. Endovascular treatment of ruptured tiny aneurysms. J cerebrovasc endovasc neurosurg. 2019;21(2):67-76.
778. Ko JK, Kim HS, Choi HJ, Lee TH, Yun EY, Choi CH. Endovascular treatment of ruptured pericallosal artery aneurysms. J Korean Neurosurg Soc. 2015;58(3):197-204.
779. Baek JW, Jin SC, Kim ST, Jeong HW, Jeong YG, Heo YJ, et al. Radiological and clinical outcomes of endovascular coiling of proximal A1 aneurysms. J Clin Neurosci. 2020;73:67-73.
780. Luzzi S, Gragnaniello C, Giotta Lucifero A, Del Maestro M, Galzio R. Surgical Management of Giant Intracranial Aneurysms: Overall Results of a Large Series. World Neurosurg. 2020;144:e119-e37.
781. Luo B, Kang H, Zhang H, Li T, Liu J, Song D, et al. Pipeline Embolization device for intracranial aneurysms in a large Chinese cohort: factors related to aneurysm occlusion. Ther Adv Neurol Disord. 2020;13.
782. Lukic S, Mijailovic M, Kovacevic V, Opancina V. Evaluation of safety and successfulness of the coil embolization of intracranial aneurysms. Serb J Exp Clin Res. 2019;20(2):17-22.
783. Luecking H, Doerfler A, Goelitz P, Hoelter P, Engelhorn T, Lang S. Two- to five-year follow-up of 78 patients after treatment with the Flow Redirection Endoluminal Device. Intervent Neuroradiol. 2020;26(1):38-44.
784. Luecking H, Struffert T, Goelitz P, Engelhorn T, Brandner S, Kuramatsu JB, et al. Stent-Assisted Coiling Using Leo+ Baby Stent : Immediate and Mid-Term Results. 2020.
785. Lopes DK, Jang DK, Cekirge S, Fiorella D, Hanel RA, Kallmes DF, et al. Morbidity and Mortality in Patients With Posterior Circulation Aneurysms Treated With the Pipeline Embolization Device: A Subgroup Analysis of the International Retrospective Study of the Pipeline Embolization Device. Neurosurgery. 2018;83(3):488-500.
786. Leonardi M, Dall'Olio M, Ortiz Vasquez O, Quercetti C. Preliminary experience of cerecyte coils in the treatment of intracranial aneurysms. Intervent Neuroradiol. 2008;14(3):285-92.

787. Le Feuvre DE, Taylor AG. Endovascular cerebral aneurysm treatment: Long-term outcomes. S Afr Med J. 2008;98(12):954-7.
788. Labeyrie PE, Gory B, Aguilar-Perez M, Pomero E, Biondi A, Riva R, et al. The pCONus Device for Treatment of Complex Wide-Neck Anterior Communicating Artery Aneurysms. World Neurosurg. 2017;101:498-505.
789. Kühn AL, Hou SY, Puri AS, Silva CF, Gounis MJ, Wakhloo AK. Stent-assisted coil embolization of aneurysms with small parent vessels: Safety and efficacy analysis. J Neurointervent Surg. 2016;8(6):581-5.
790. Kim CH, Kim YH, Sung SK, Son DW, Song GS, Lee SW. Clinical safety and effectiveness of stent-assisted coil embolization with neuroform atlas stent in intracranial aneurysm. J Korean Neurosurg Soc. 2019;63(1):80-8.
791. Lafuente J, Maurice-Williams RS. Ruptured intracranial aneurysms: The outcome of surgical treatment in experienced hands in the period prior to the advent of endovascular coiling. J Neurol Neurosurg Psychiatry. 2003;74(12):1680-4.
792. Kaya HE, Bakdık S, Keskin F, Erdi MF, Koç O. Endovascular treatment of intracranial aneurysms using the Woven EndoBridge (WEB) device: retrospective analysis of a single center experience. Clin Imaging. 2020;59(1):25-9.
793. Kannan S, Yamada Y, Miyatani K, Teranishi T, Marathi AR, Mohan K, et al. Use of our Protocol of Multimodality Tools to Aid in the Safe Microsurgical Clipping of Unruptured Anterior Circulation Aneurysms. Asian J Neurosurg. 2019;14(3):773-9.
794. İnci S, Akbay A, Aslan T. The Longest Angiographic and Clinical Follow-Up of Microsurgically Treated Giant Intracranial Aneurysms: Experience with 70 Cases. World Neurosurg. 2020;134:e412-e21.
795. Igüs B, Selçuk H, Kara B, Fırat A, Salık AE. Endovascular treatment of intracranial aneursyms with flow diverter stents. Med J Bakirkoy. 2020;16(1):9-14.
796. Guglielmi G, Viñuela E, Duckwiler G, Jahan R, Cotroneo E, Gigli R. Endovascular treatment of middle cerebral artery aneurysms. Overall perioperative results. Apropos of 113 cases. Intervent Neuroradiol. 2008;14(3):241-5.
797. Griessenauer CJ, Möhlenbruch MA, Hendrix P, Ulfert C, Islak C, Sonnberger M, et al. The Fred for cerebral aneurysms of the posterior circulation: A subgroup analysis of the EUFRED registry. Am J Neuroradiol. 2020;41(4):658-62.
798. Gory B, Berge J, Bonafé A, Pierot L, Spelle L, Piotin M, et al. Flow diverters for intracranial aneurysms the DIVERSION national prospective cohort study. Stroke. 2019;50(12):3471-80.
799. Goel G, Gupta V, Chinchure S, Gupta A, Kaur G, Jha AN. A decade after International Subarachnoid Aneurysm Trial: Coiling as a first choice treatment in the management of intracranial aneurysms - Technical feasibility and early management outcomes. Asian J Neurosurg. 2014;9(3):137-43.
800. Gariel F, Marnat G, Barreau X, Menegon P, Bourcier R, Pierot L, et al. Safety and efficacy of the Silk flow diverter: Insight from the DIVERSION prospective cohort study. J Neuroradiol. 2020.
801. Fuse A, Rodesch G, Alvarez H, Lasjaunias P. Endovascular management of intradural berry aneurysms: Review of 203 consecutive patients managed between 1993 and 1998 morphological and clinical results at mid-term follow-up. Intervent Neuroradiol. 2000;6(1):27-36.
802. Fujimoto M, Lylyk I, Bleise C, Albina P, Chudyk J, Lylyk P. Long-Term Outcomes of the WEB Device for Treatment of Wide-Neck Bifurcation Aneurysms. AJNR Am J Neuroradiol. 2020;41(6):1031-6.
803. Foreman PM, Salem MM, Griessenauer CJ, Dmytriw AA, Parra-Farinas C, Nicholson P, et al. Flow Diversion for Treatment of Partially Thrombosed Aneurysms: A Multicenter Cohort. World Neurosurg. 2020;135:e164-e73.
804. Enomoto Y, Egashira Y, Matsubara H, Yoshimura S, Iwama T. Long-Term Outcome of Endovascular Therapy for Large or Giant Thrombosed Intracranial Aneurysms. World Neurosurg. 2020;144:e507-e12.
805. Dinc H, Saatci I, Oguz S, Baltacioglu F, Yildiz A, Donmez H, et al. Long-term clinical and angiographic follow-up results of the dual-layer flow diverter device (FRED) for the treatment of

© 2023 Volovici V et al. *JAMA Network Open.*

intracranial aneurysms in a multicenter study. Neuroradiology. 2021.
806. Decharin P, Churojana A, Aurboonyawat T, Chankaew E, Songsaeng D, Sangpetngam B, et al. Success Rate of Simple Coil Embolization in Wide-Neck Aneurysm with Aneurysmal Shoulder. Asian J Neurosurg. 2020;15(3):594-600.
807. Chiu AH, De Vries J, O'Kelly CJ, Riina H, McDougall I, Tippett J, et al. The second-generation eCLIPs Endovascular Clip System: Initial experience. J Neurosurg. 2018;128(2):482-9.
808. De Beule T, Boulanger T, Heye S, van Rooij WJ, van Zwam WH, Stockx L. p64 flow diverter: Results in 108 patients from a single center. Intervent Neuroradiol. 2020.
809. Chitale R, Gonzalez LF, Randazzo C, Dumont AS, Tjoumakaris S, Rosenwasser R, et al. Single center experience with pipeline stent: Feasibility, technique, and complications. Neurosurgery. 2012;71(3):679-91.
810. Chen L, Zheng C, Wu J, Gong J, Gao Y, Wan S. The Enterprise2 Stent for Endovascular Treatment of Intracranial Aneurysms: Short-Term Results From a Single Center Experience. Front Neurol. 2020;11.
811. Chalouhi N, Thakkar V, Tjoumakaris S, Fernando Gonzalez L, Hasan D, Rosenwasser R, et al. Microsurgical clipping of large and giant cerebral aneurysms: A single-center contemporary experience. J Clin Neurosci. 2014;21(8):1424-7.
812. Ban SP, Cho WS, Kim JE, Kim CH, Bang JS, Son YJ, et al. Bypass surgery for complex intracranial aneurysms: 15 years of experience at a single institution and review of pertinent literature. Oper Neurosurg. 2017;13(6):679-88.
813. Beuing O, Lenz A, Donitza A, Becker M, Serowy S, Skalej M. Stent-assisted coiling of broad-necked intracranial aneurysms with a new braided microstent (Accero): procedural results and long-term follow-up. Sci Rep. 2020;10(1):412.
814. Birknes JK, Hwang SK, Pandey AS, Cockroft K, Dyer AM, Benitez RP, et al. Feasibility and limitations of endovascular coil embolization of anterior communicating artery aneurysms: morphological considerations. Neurosurgery. 2006;59(1):43-52; discussion 43-52.
815. Borota L, Nyberg C, Lenell S, Semnic R, Mahmoud E. Endovascular treatment of type 1 and type 4 non-saccular aneurysms of cerebral arteries - a single-Centre experience. Interv. 2021;neuroradiol..1591019920988204.
816. Bracard S, Lebedinsky A, Anxionnat R, Neto JM, Audibert G, Long Y, et al. Endovascular treatment of Hunt and Hess grade IV and V aneuryms. AJNR Am J Neuroradiol. 2002;23(6):953-7.
817. Brilstra EH, Rinkel GJE, Klijn CJM, Van der Zwan A, Algra A, Lo RTH, et al. Excimer laser-assisted bypass in aneurysm treatment: Short-term outcomes. J Neurosurg. 2002;97(5):1029-35.
818. Brzegowy P, Kucybała I, Krupa K, Łasocha B, Wilk A, Latacz P, et al. Angiographic and clinical results of anterior communicating artery aneurysm endovascular treatment. Wideochir Inne Tech Maloinwazyjne. 2019;14(3):451-60.
819. Chen X, Li H, Wang MZ, Li MG, Cao Y, Zhang D, et al. Clinical features and outcomes of PComA aneurysms originating from fetal posterior communicating arteries in a single institution. Chin Neurosurg J. 2020;6(1).
820. Cherian J, Srinivasan V, Froehler MT, Grossberg JA, Cawley CM, Hanel RA, et al. Flow Diversion for Treatment of Intracranial Aneurysms in Pediatric Patients: Multicenter Case Series. Neurosurgery. 2020;87(1):53-62.
821. Trivelato FP, Abud DG, Ulhôa AC, Waihrich ES, Abud TG, Castro Afonso LH, et al. Derivo Embolization Device for the Treatment of Intracranial Aneurysms. Stroke. 2019:STROKEAHA119025407.
822. Kallmes DF, Hanel R, Lopes D, Boccardi E, Bonafé A, Cekirge S, et al. International retrospective study of the pipeline embolization device: A multicenter aneurysm treatment study. Am J Neuroradiol. 2015;36(1):108-15.
823. Kobayashi S, Satoh A, Koguchi Y, Yamauchi T, Itoh S, Ooishi H, et al. Endovascular treatment with GDC for severe acute SAH: Comparison with early direct surgery. Intervent Neuroradiol. 2000;6(SUPPL. 1):79-84.

© 2023 Volovici V et al. *JAMA Network Open.*

824. Fargen KM, Mocco J, Neal D, Dewan MC, Reavey-Cantwell J, Woo HH, et al. A multicenter study of stent-assisted coiling of cerebral aneurysms with a y configuration. Neurosurgery. 2013;73(3):466-72.
825. Garbossa D, Panciani PP, Fornaro R, Crobeddu E, Marengo N, Fronda C, et al. Subarachnoid hemorrhage in elderly: advantages of the endovascular treatment. Geriatr Gerontol Int. 2012;12(1):46-9.
826. Jung YJ, Chang CH, Kim JH. Advantages of Coil Embolization Performed Immediately After Diagnostic Cerebral Digital Subtraction Angiography in Unruptured Intracranial Aneurysms: Patients' Perspective. World Neurosurg. 2019.
827. Limbucci N, Cirelli C, Valente I, Nappini S, Renieri L, Laiso A, et al. Y-stenting versus pulserider-assisted coiling in the treatment of wide-neck bifurcation aneurysms: Role of anatomical features on midterm results. Neurosurgery. 2020;87(2):329-37.
828. Ibrahim Ali AM, Ashmawy GAHO, Eassa AYE, Mansour OY. Hyperacute versus subacute coiling of aneurysmal subarachnoid hemorrhage a short-term outcome and single-center experience, pilot study. Front Neurol. 2016;7(JUN).
829. Gu DQ, Zhang X, Luo B, Long XA, Duan CZ. Impact of Ultra-early Coiling on Clinical Outcome after Aneurysmal Subarachnoid Hemorrhage in Elderly Patients. Acad Radiol. 2012;19(1):3-7.
830. Hauck EF, Wohlfeld B, Welch BG, White JA, Samson D. Clipping of very large or giant unruptured intracranial aneurysms in the anterior circulation: An outcome study - Clinical article. J Neurosurg. 2008;109(6):1012-8.
831. Brinjikji W, Kallmes DF, Cloft HJ, Lanzino G. Age-related outcomes following intracranial aneurysm treatment with the Pipeline Embolization Device: A subgroup analysis of the IntrePED registry. J Neurosurg. 2016;124(6):1726-30.
832. Ohnishi H, Kosimae N. Surgical management of cerebral aneurysms in comparison with endovascular treatment. Interv neuroradiol. 1998;4 Suppl 1:149-52.
833. Oishi H, Fujii T, Yatomi K, Teranishi K, Suzuki K, Mishima Y, et al. Stent-assisted coil embolization of unruptured middle cerebral artery aneurysms using LVIS Jr. stents. J Clin Neurosci. 2020;80:87-91.
834. Burkhardt JK, Winkler EA, Weller J, Lawton MT. Early versus Delayed Microsurgical Clipping of Additional Unruptured Aneurysms in Patients with Aneurysmal Subarachnoid Hemorrhage. World Neurosurg. 2020;142:e233-e7.
835. Yuan B, Zhou XM, Fan JM, Chen SJ, You ZQ, Xu WD, et al. Safety and efficacy of different therapeutic strategies in the endovascular treatment of anterior cerebral artery aneurysms with different features: A single centre experience. Clin Neurol Neurosurg. 2020;193.
836. Klompenhouwer EG, Dings JTA, Van Oostenbrugge RJ, Oei S, Wilmink JT, Van Zwam WH. Single-center experience of surgical and endovascular treatment of ruptured intracranial aneurysms. Am J Neuroradiol. 2011;32(3):570-5.
837. Iwamuro Y, Nakahara I, Higashi T, Iwaasa M, Watanabe Y, Tsunetoshi K, et al. Result of neck clipping and coil embolization as a treatment for unruptured aneurysm. Intervent Neuroradiol. 2007;13(SUPPL. 1):151-6.
838. Jiang C, Yu Y, Hong B, Fu QL, Liu JM, Huang QH. Stent-assisted coil embolization for the treatment of ruptured aneurysms at the anterior circulation: Comparison between hydrosoft coils and bare platinum coils. Cardiovasc Intervent Radiol. 2014;37(4):935-41.
839. Jang EW, Jung JY, Hong CK, Joo JY. Benefits of surgical treatment for unruptured intracranial aneurysms in elderly patients. J Korean Neurosurg Soc. 2011;49(1):20-5.
840. Jamróz T, Jakutowicz I, Hofman M, Kołodkiewicz M, Ćmiel M, Łapaj A, et al. Safety and efficacy of treatment of very small intracranial aneurysms. Pol J Radiol. 2019;84:e360-e4.
841. Jahshan S, Abla AA, Natarajan SK, Drummond PS, Kan P, Karmon Y, et al. Results of stent-assisted vs non-stent-assisted endovascular therapies in 489 cerebral aneurysms: Single-center experience. Clin Neurosurgery. 2013;72(2):232-9.
842. Jin SC, Ahn JS, Kwun BD, Kwon DH. Analysis of clinical and radiological outcomes in microsurgical

© 2023 Volovici V et al. *JAMA Network Open.*

and endovascular treatment of basilar apex aneurysms. J Korean Neurosurg Soc. 2009;45(4):224-30.
843. Johnston SC, Wilson CB, Halbach VV, Higashida RT, Dowd CF, McDermott MW, et al. Endovascular and surgical treatment of unruptured cerebral aneurysms: Comparison of risks. Ann Neurol. 2000;48(1):11-9.
844. Ishii A, Murayama Y, Nien YL, Yuki I, Adapon PH, Kim R, et al. Immediate and midterm outcomes of patients with cerebral aneurysms treated with Matrix1 and Matrix2 coils: A comparative analysis based on a single-center experience in 250 consecutive cases. Neurosurgery. 2008;63(6):1071-7.
845. Kabbasch C, Goertz L, Siebert E, Herzberg M, Borggrefe J, Krischek B, et al. WEB embolization versus stent-assisted coiling: Comparison of complication rates and angiographic outcomes. J Neurointervent Surg. 2019;11(8):812-6.
846. Kadkhodayan Y, Somogyi CT, Cross IDT, Derdeyn CP, Zipfel GJ, Chicoine MR, et al. Technical, angiographic and clinical outcomes of Neuroform 1, 2, 2 Treo and 3 devices in stent-assisted coiling of intracranial aneurysms. J Neurointervent Surg. 2012;4(5):368-74.
847. Kaesmacher J, Müller-Leisse C, Huber T, Boeckh-Behrens T, Haller B, Shiban E, et al. Volume versus standard coils in the treatment of intracranial aneurysms. J Neurointerv Surg. 2016;8(10):1034-40.
848. Kato Y, Sano H, Dindorkar K, Abe M, Nagahisa S, Iwata S, et al. Treatment of unruptured intracranial aneurysms - A clinicopathological correlation. Acta Neurochir. 2001;143(7):681-7.
849. Katsaridis V, Papagiannaki C, Violaris C. Guglielmi detachable coils versus matrix coils: A comparison of the immediate posttreatment results of the embolization of 364 cerebral aneurysms in 307 patients: A single-center, single-surgeon experience. Am J Neuroradiol. 2006;27(9):1841-8.
850. Kawabata Y, Horikawa F, Ueno Y, Sawada M, Isaka F, Miyake H. Clinical predictors of delayed cerebral ischemia after subarachnoid hemorrhage: First experience with coil embolization in the management of ruptured cerebral aneurysms. J Neurointervent Surg. 2011;3(4):344-7.
851. Kawabe T, Tenjin H, Hayashi Y, Kakita K, Kubo S. Midterm prevention of rebleeding by Guglielmi detachable coils in ruptured intracranial aneurysms less than 10 mm. Clin Neurol Neurosurg. 2006;108(2):163-7.
852. Khandelwal P, Kato Y, Sano H, Yoneda M, Kanno T. Treatment of ruptured intracranial aneurysms: Our approach. Minimally Invasive Neurosurg. 2005;48(6):325-9.
853. Kim BM, Kim DI, Shin YS, Chung EC, Kim DJ, Suh SH, et al. Clinical outcome and ischemic complication after treatment of anterior choroidal artery aneurysm: Comparison between surgical clipping and endovascular coiling. Am J Neuroradiol. 2008;29(2):286-90.
854. Kim DJ, Heo Y, Byun J, Park JC, Ahn JS, Lee DH, et al. Role of microsurgery for treatment of posterior circulation aneurysms in the endovascular era. J cerebrovasc endovasc neurosurg. 2020;22(3):141-55.
855. Izar B, Rai A, Raghuram K, Rotruck J, Carpenter J. Comparison of devices used for stent-assisted coiling of intracranial aneurysms. PLoS ONE. 2011;6(9).
856. Kim SM, Lee HG, Choi BS, Kim JS, Lee SJ, Kim HY, et al. Recurrence of Small Cerebral Aneurysms (< 4 mm) Treated Endovascularly Using Target NanoTM Coils. J cerebrovasc endovasc neurosurg. 2018;20(2):106-11.
857. Kim SY, Park DS, Park HY, Chun YI, Moon CT, Roh HG. Simple coiling versus stent-assisted coiling of paraclinoid aneurysms: Radiological outcome in a single center study. J Korean Neurosurg Soc. 2017;60(6):644-53.
858. Kim DJ, Suh SH, Lee JW, Kim BM, Lee JW, Huh SK, et al. Influences of stents on the outcome of coil embolized intracranial aneurysms: Comparison between a stent-remodeled and non-remodeled treatment. Acta Neurochir. 2010;152(3):423-8.
859. Kim LJ, Tariq F, Levitt M, Barber J, Ghodke B, Hallam DK, et al. Multimodality treatment of complex unruptured cavernous and paraclinoid aneurysms. Neurosurgery. 2014;74(1):51-61.
860. Chalouhi N, Teufack S, Chandela S, Dalyai R, Tjoumakaris S, Hasan DM, et al. Aneurysmal

subarachnoid hemorrhage in patients under 35-years-old: A single-center experience. Clin Neurol Neurosurg. 2013;115(6):665-8.
861. Chalouhi N, Tjoumakaris S, Starke RM, Gonzalez LF, Randazzo C, Hasan D, et al. Comparison of flow diversion and coiling in large unruptured intracranial saccular aneurysms. Stroke. 2013;44(8):2150-4.
862. Jee TK, Nam TM, Yeon JY, Kim KH, Jeon P, Kim JS, et al. Intracranial Aneurysms in Young Adult Patients: Surgical and Endovascular Treatment Outcomes. World Neurosurg. 2020;136:e214-e22.
863. Kato N, Nishimura K, Sonoda S, Kakizaki S, Nagayama G, Aoki K, et al. Comparison of Clinical Outcomes After Stent-Assisted Coiling with 3 Types of Self-Expanding Laser-Cut Stents in Patients with Wide-Necked Intracranial Aneurysms. World Neurosurg. 2020.
864. Kiselev R, Orlov K, Dubovoy A, Berestov V, Gorbatykh A, Kislitsin D, et al. Flow diversion versus parent artery occlusion with bypass in the treatment of complex intracranial aneurysms: Immediate and short-term outcomes of the randomized trial. Clin Neurol Neurosurg. 2018;172:183-9.
865. Brinjikji W, Rabinstein AA, Lanzino G, Kallmes DF, Cloft HJ. Effect of age on outcomes of treatment of unruptured cerebral aneurysms: A study of the national inpatient sample 2001-2008. Stroke. 2011;42(5):1320-4.
866. Brinjikji W, White PM, Nahser H, Wardlaw J, Sellar R, Cloft HJ, et al. HydroCoils reduce recurrence rates in recently ruptured medium-sized intracranial aneurysms: A subgroup analysis of the HELPS trial. Am J Neuroradiol. 2015;36(6):1136-41.
867. Nagashima H, Kobayashi S, Tanaka Y, Hongo K. Endovascular therapy versus surgical clipping for basilar artery bifurcation aneurysm: Retrospective analysis of 117 cases. J Clin Neurosci. 2004;11(5):475-9.
868. Choi JH, Lee KS, Kim BS, Shin YS. Treatment outcomes of large and giant intracranial aneurysms according to various treatment modalities. Acta Neurochir. 2020;162(11):2745-52.
869. Brinjikji W, White PM, Nahser H, Wardlaw J, Sellar R, Gholkar A, et al. HydroCoils are associated with lower angiographic recurrence rates than are bare platinum coils in treatment of "difficult-to-treat" aneurysms: A post hoc subgroup analysis of the HELPS trial. Am J Neuroradiol. 2015;36(9):1689-94.
870. Chun YI, Roh HG, Choe WJ, Cho J, Moon CT, Koh YC. Tiny aneurysms treated with single coil: Morphological comparison between bare platinum coil and matrix coil. Clin Neurol Neurosurg. 2013;115(5):529-34.
871. Aoki T, Hirohata M, Noguchi K, Komaki S, Orito K, Morioka M. Comparative outcome analysis of anterior choroidal artery aneurysms treated with endovascular coiling or surgical clipping. Surg Neurol Intl. 2016;7(19):S504-S9.
872. Pennig L, Goertz L, Hoyer UCI, Dorn F, Siebert E, Herzberg M, et al. The Woven EndoBridge (WEB) Versus Conventional Coiling for Treatment of Patients with Aneurysmal Subarachnoid Hemorrhage: Propensity Score-Matched Analysis of Clinical and Angiographic Outcome Data. World Neurosurg. 2020.
873. Natarajan SK, Sekhar LN, Ghodke B, Britz GW, Bhagawati D, Temkin N. Outcomes of ruptured intracranial aneurysms treated by microsurgical clipping and endovascular coiling in a high-volume center. Am J Neuroradiol. 2008;29(4):753-9.
874. Navrátil O, Ďuriš K, Juráň V, Svoboda K, Hustý J, Hovorka E, et al. Current treatment of anterior communicating artery aneurysms: Single center study. Brain Sci. 2020;10(8):1-11.
875. Nishido H, Piotin M, Bartolini B, Pistocchi S, Redjem H, Blanc R. Analysis of complications and recurrences of aneurysm coiling with special emphasis on the stent-assisted technique. Am J Neuroradiol. 2014;35(2):339-44.
876. Ocal O, Peker A, Balci S, Arat A. Placement of a stent within a flow diverter improves aneurysm occlusion rates. Am J Neuroradiol. 2019;40(11):1932-8.
877. Oh SY, Lee KS, Kim BS, Shin YS. Management strategy of surgical and endovascular treatment of unruptured paraclinoid aneurysms based on the location of aneurysms. Clin Neurol Neurosurg. 2015;128:72-7.
878. Orlický M, Sameš M, Hejčl A, Vachata P. Carotid-ophthalmic aneurysms-Our results and treatment

© 2023 Volovici V et al. *JAMA Network Open.*

strategy. Br J Neurosurg. 2015;29(2):237-42.
879. Pan J, Xiao F, Szeder V, Yan M, Fan W, Gu J, et al. Stent, balloon-assisted coiling and double microcatheter for treating wide-neck aneurysms in anterior cerebral circulation. Neurol Res. 2013;35(10):1002-8.
880. Park J, Woo H, Kang DH, Kim Y, Baik SK. Ruptured intracranial aneurysms with small basal outpouching: Incidence of basal rupture and results of surgical and endovascular treatments. Neurosurgery. 2012;71(5):994-1001.
881. Peschillo S, Caporlingua A, Resta MC, Paul Peluso JP, Burdi N, Sourour N, et al. Endovascular treatment of large and giant carotid aneurysms with flow-diverter stents alone or in combination with coils: A multicenter experience and long-term follow-up. Oper Neurosurg. 2017;13(4):492-502.
882. Pierot L, Spelle L, Leclerc X, Cognard C, Bonafé A, Moret J. Endovascular treatment of unruptured intracranial aneurysms: Comparison of safety of remodeling technique and standard treatment with coils. Radiology. 2009;251(3):846-55.
883. Pierot L, Cognard C, Anxionnat R, Ricolfi F. Remodeling technique for endovascular treatment of ruptured intracranial aneurysms had a higher rate of adequate postoperative occlusion than did conventional coil embolization with comparable safety. Radiology. 2011;258(2):546-53.
884. Popiela TJ, Brzegowy P, Łasocha B, Urbanik A. The effectiveness of Penumbra 400 micro-coils in the embolization of large cerebral aneurysms. Neurol Neurochir Pol. 2017;51(4):304-10.
885. Proust F, Debono B, Hannequin D, Gerardin E, Clavier E, Langlois O, et al. Treatment of anterior communicating artery aneurysms: complementary aspects of microsurgical and endovascular procedures. J Neurosurg. 2003;99(1):3-14.
886. Raymond J, Klink R, Chagnon M, Barnwell SL, Evans AJ, Mocco J, et al. Hydrogel versus bare platinum coils in patients with large or recurrent aneurysms prone to recurrence after endovascular treatment: A randomized controlled trial. Am J Neuroradiol. 2017;38(3):432-41.
887. Ren Y, Liu L, Sun H, Liu Y, Li H, Ma L, et al. Microsurgical versus Endovascular Treatments for Blood-Blister Aneurysms of the Internal Carotid Artery: A Retrospective Study of 83 Patients in a Single Center. World Neurosurg. 2018;109:e615-e24.
888. Richling B, Gruber A, Killer M, Bavinzski G. Treatment of ruptured saccular intracranial aneurysms by microsurgery and electrolytically detachable coils: Evaluation of outcome and long-term follow-up. Oper Tech Neurosurg. 2000;3(4):282-99.
889. Lanzino G, Fraser K, Kanaan Y, Wagenbach A. Treatment of ruptured intracranial aneurysms since the International Subarachnoid Aneurysm Trial: Practice utilizing clip ligation and coil embolization as individual or complementary therapies. J Neurosurg. 2006;104(3):344-9.
890. Linfante I, DeLeo IMJ, Gounis MJ, Brooks CS, Wakhloo AK. Cerecyte versus platinum coils in the treatment of intracranial aneurysms: Packing attenuation and clinical and angiographic midterm results. Am J Neuroradiol. 2009;30(8):1496-501.
891. Koh RKM, Ng Z, Low SYY, Chua FHZ, Chou N, Low SW, et al. Management of ruptured intracranial aneurysms in the post-ISAT era: Outcome of surgical clipping versus endovascular coiling in a Singapore tertiary institution. Singapore Med J. 2013;54(6):332-8.
892. Kunz M, Bakhshai Y, Zausinger S, Fesl G, Janssen H, Brückmann H, et al. Interdisciplinary treatment of unruptured intracranial aneurysms: Impact of intraprocedural rupture and ischemia in 563 aneurysms. J Neurol. 2013;260(5):1304-13.
893. Lad SP, Babu R, Rhee MS, Franklin RL, Ugiliweneza B, Hodes J, et al. Long-term economic impact of coiling vs clipping for unruptured intracranial aneurysms. Neurosurgery. 2013;72(6):1000-11.
894. Lawson MF, Neal DW, Mocco J, Hoh BL. Rationale for treating unruptured intracranial aneurysms: Actuarial analysis of natural history risk versus treatment risk for coiling or clipping based on 14,050 patients in the nationwide inpatient sample database. World Neurosurg. 2013;79(3-4):472-8.
895. Lee JY, Seo JH, Lee SJ, Son YJ, Cho YD, Kang HS, et al. Mid-term outcome of intracranial

© 2023 Volovici V et al. *JAMA Network Open.*

aneurysms treated with HydroSoft coils compared to historical controls treated with bare platinum coils: A single-center experience. Acta Neurochir. 2014;156(9):1687-94.
896. Liu YQ, Wang QJ, Zheng T, Zhang X, Li XF, Cui XB, et al. Single-centre comparison of procedural complications, clinical outcome, and angiographic follow-up between coiling and stent-assisted coiling for posterior communicating artery aneurysms. J Clin Neurosci. 2014;21(12):2140-4.
897. Lehto H, Niemelä M, Kivisaari R, Laakso A, Jahromi BR, Hijazy F, et al. Intracranial Vertebral Artery Aneurysms: Clinical Features and Outcome of 190 Patients. World Neurosurg. 2015;84(2):380-9.
898. Lan Q, Zhang H, Zhu Q, Chen A, Chen Y, Xu L, et al. Keyhole Approach for Clipping Intracranial Aneurysm: Comparison of Supraorbital and Pterional Keyhole Approach. World Neurosurg. 2017;102:350-9.
899. Li J, Su L, Ma J, Kang P, Ma L, Ma L. Endovascular Coiling Versus Microsurgical Clipping for Patients With Ruptured Very Small Intracranial Aneurysms: Management Strategies and Clinical Outcomes of 162 Cases. World Neurosurg. 2017;99:763-9.
900. Koyanagi M, Fukuda H, Saiki M, Tsuji Y, Lo B, Kawasaki T, et al. Effect of choice of treatment modality on the incidence of shunt-dependent hydrocephalus after aneurysmal subarachnoid hemorrhage. J Neurosurg. 2019;130(3):949-55.
901. Kumar A, Kutty RK, Yamada Y, Tanaka R, Ravisankar V, Musara A, et al. A Retrospective Analysis of Treatment Outcomes of 40 Incidental Cavernous Carotid Aneurysms. World Neurosurg. 2019;130:e1034-e40.
902. Kwinta BM, Kliś KM, Krzyżewski RM, Wilk A, Dragan M, Grzywna E, et al. Elective Management of Unruptured Intracranial Aneurysms in Elderly Patients in a High-Volume Center. World Neurosurg. 2019;126:e1343-e51.
903. Li W, Wang Y, Zhang Y, Wang K, Zhang Y, Tian Z, et al. Efficacy of LVIs vs. Enterprise stent for endovascular treatment of medium-sized intracranial aneurysms: A hemodynamic comparison study. Front Neurol. 2019;10(MAY).
904. Chalouhi N, Penn DL, Tjoumakaris S, Jabbour P, Gonzalez LF, Starke RM, et al. Treatment of small ruptured intracranial aneurysms: Comparison of surgical and endovascular options. J Am Heart Assoc. 2012;1(4).
905. Chalouhi N, Starke RM, Koltz MT, Jabbour PM, Tjoumakaris SI, Dumont AS, et al. Stent-assisted coiling versus balloon remodeling of wide-neck aneurysms: Comparison of angiographic outcomes. Am J Neuroradiol. 2013;34(10):1987-92.
906. Ghorbani M, Griessenauer CJ, Wipplinger C, Nouri M, Asaadi S, Hejazian E, et al. Surgical clipping compared to endovascular coiling of ruptured coil able middle cerebral aneurysms: A single-center experience. Interdiscip Neurosurg Adv Tech Case Manage. 2020;21.
907. Krylov VV, Klimov AB, Polunina NA. Characteristics of the morphology, clinical features, diagnosis, and treatment of patients with giant aneurysms of the brain vessels. Neurosci Behav Physiol. 2012;42(9):980-7.
908. La Pira B, Brinjikji W, Burrows AM, Cloft HJ, Vine RL, Lanzino G. Unruptured internal carotid artery bifurcation aneurysms: general features and overall results after modern treatment. Acta Neurochir (Wien). 2016;158(11):2053-9.
909. Lim J, Cho YD, Hong N, Lee J, Yoo DH, Kang HS. Follow-up outcomes of intracranial aneurysms treated using braided or laser-cut stents with closed-cell design: a propensity score-matched case-controlled comparison. J Neurointerv Surg. 2020.
910. Liu Y, Wang F, Wang M, Zhang G. Comparison of stent-assisted coil placement and coiling-only for the treatment of ruptured intracranial aneurysms. Med Sci Monit. 2017;23:5697-704.
911. Lodi Y, Latorre J, El-Zammar Z, Swarnkar A, Gordon V, Whapham J, et al. Single Stage versus Multi-staged Stent-assisted Endovascular Repair of Intracranial Aneurysms. J vasc interv radiol neurol. 2011;4(2):24-8.

912. Lot G, Houdart E, Cophignon J, Casasco A, George B. Management of intracranial aneurysms by surgical and endovascular treatment - Modalities and results from a series of 395 cases. Neurol Med -Chir. 1998;38(SUPPL.):21-5.
913. Liu J, Peng C, Zhu G, Sheng C, Song S, Cheng Z, et al. Comparison of surgical clipping and endovascular coiling in the treatment of oculomotor nerve palsy caused by posterior communicating artery aneurysm. Medicine (Baltimore). 2020;99(47):e22969.
914. Aboukaïs R, Zairi F, Bourgeois P, Thines L, Kalsoum E, Leclerc X, et al. Clinical and imaging follow-up after surgical or endovascular treatment in patients with unruptured carotid-ophthalmic aneurysm. Clin Neurol Neurosurg. 2014;125:155-9.
915. Aboukais R, Zairi F, Thines L, Aguettaz P, Leclerc X, Lejeune JP. Multidisciplinary management of intracranial aneurysms: The experience of Lille university hospital center. Neurochirurgie. 2014;60(6):283-7.
916. Aboukaïs R, Zairi F, Bourgeois P, Boustia F, Leclerc X, Lejeune JP. Pericallosal aneurysm: A difficult challenge for microsurgery and endovascular treatment. Neurochirurgie. 2015;61(4):244-9.
917. Adeeb N, Griessenauer CJ, Foreman PM, Moore JM, Motiei-Langroudi R, Chua MH, et al. Comparison of Stent-Assisted Coil Embolization and the Pipeline Embolization Device for Endovascular Treatment of Ophthalmic Segment Aneurysms: A Multicenter Cohort Study. World Neurosurg. 2017;105:206-12.
918. Ahmed AZ, Zohdi AM, Zaghloul MS, Elsamman AK. Endovascular coiling versus surgical clipping in the treatment of ruptured anterior communicating artery aneurysm in Cairo University Hospitals. Egypt J Radiol Nucl Med. 2013;44(3):523-30.
919. Bendok BR, Abi-Aad KR, Ward JD, Kniss JF, Kwasny MJ, Rahme RJ, et al. The Hydrogel Endovascular Aneurysm Treatment Trial (HEAT): A Randomized Controlled Trial of the Second-Generation Hydrogel Coil. Neurosurgery. 2020;86(5):615-24.
920. Bendszus M, Bartsch AJ, Solymosi L. Endovascular occlusion of aneurysms using a new bioactive coil: A matched pair analysis with bare platinum coils. Stroke. 2007;38(10):2855-7.
921. Alawi AWS, Edgell RC, Elbabaa SK, Callison RC, Khalili YAL, Allam H, et al. Treatment of cerebral aneurysms in children: Analysis of the Kids' Inpatient Database. J Neurosurg Pediatr. 2014;14(1):23-30.
922. Allen BB, Forgacs PB, Fakhar MA, Wu X, Gerber LM, Boddu S, et al. Association of Seizure Occurrence with Aneurysm Treatment Modality in Aneurysmal Subarachnoid Hemorrhage Patients. Neurocrit Care. 2018;29(1):62-8.
923. AlMatter M, Bhogal P, Aguilar Pérez M, Hellstern V, Bäzner H, Ganslandt O, et al. Evaluation of safety, efficacy and clinical outcome after endovascular treatment of aneurysmal subarachnoid hemorrhage in coil-first setting. A 10-year series from a single center. J Neuroradiol. 2018;45(6):349-56.
924. Barker IFG, Amin-Hanjani S, Butler WE, Hoh BL, Rabinov JD, Pryor JC, et al. Age-dependent Differences in Short-term Outcome after Surgical or Endovascular Treatment of Unruptured Intracranial Aneurysms in the United States, 1996-2000. Neurosurgery. 2004;54(1):18-30.
925. Bekelis K, Missios S, Coy S, Singer RJ, MacKenzie TA. New York State: Comparison of Treatment Outcomes for Unruptured Cerebral Aneurysms Using an Instrumental Variable Analysis. J Am Heart Assoc. 2015;4(7).
926. Bhatia S, Sekula RF, Quigley MR, Williams R, Ku A. Role of calcification in the outcomes of treated, unruptured, intracerebral aneurysms. Acta Neurochir. 2011;153(4):905-11.
927. Bose RS, Dowling RJ, Yan B, Mitchell PJ. A single centre study of coil embolization of intracranial aneurysms comparing bare platinum and PGLA-coated coils. J Clin Neurosci. 2012;19(2):271-6.
928. Braun V, Rath S, Antoniadis G, Richter HP, Börm W. Treatment and outcome of aneurysmal subarachnoid haemorrhage in the elderly patient. Neuroradiology. 2005;47(3):215-21.
929. Brilstra EH, Rinkel GJE, Van Der Graaf Y, Sluzewski M, Groen RJ, Lo RTH, et al. Quality of life after treatment of unruptured intracranial aneurysms by neurosurgical clipping or by embolisation with coils: A prospective, observational study. Cerebrovasc Dis. 2004;17(1):44-52.

© 2023 Volovici V et al. *JAMA Network Open.*

930. Brinjikji W, Rabinstein AA, Nasr DM, Lanzino G, Kallmes DF, Cloft HJ. Better outcomes with treatment by coiling relative to clipping of unruptured intracranial aneurysms in the United States, 2001-2008. Am J Neuroradiol. 2011;32(6):1071-5.
931. Britz GW, Salem L, Newell DW, Eskridge J, Flum DR. Impact of surgical clipping on survival in unruptured and ruptured cerebral aneurysms: A population-based study. Stroke. 2004;35(6):1399-403.
932. Cantore G, Santoro A, Guidetti G, Delfinis CP, Colonnese C, Passacantilli E. Surgical treatment of giant intracranial aneurysms: Current viewpoint. Neurosurgery. 2008;63(4 SUPPL.):ONS279-ONS89.
933. Catapano JS, Nguyen CL, Frisoli FA, Sagar S, Baranoski JF, Cole TS, et al. Small intracranial aneurysms in the Barrow Ruptured Aneurysm Trial (BRAT). Acta Neurochir. 2021;163(1):123-9.
934. Bründl E, Böhm C, Lürding R, Schödel P, Bele S, Hochreiter A, et al. Treatment of Unruptured Intracranial Aneurysms and Cognitive Performance: Preliminary Results of a Prospective Clinical Trial. World Neurosurg. 2016;94:145-56.
935. Birski M, Wałęsa C, Gaca W, Paczkowski D, Birska J, Harat A. Clipping versus coiling for intracranial aneurysms. Neurol Neurochir Pol. 2014;48(2):122-9.
936. Cai K, Zhang Y, Shen L, Ni Y, Ji Q. Comparison of Stent-Assisted Coiling and Balloon-Assisted Coiling in the Treatment of Ruptured Wide-Necked Intracranial Aneurysms in the Acute Period. World Neurosurg. 2016;96:316-21.
937. Brunken M, Kehler U, Fiehler J, Leppien A, Eckert B. Coiling vs. clipping: Hospital stay and procedure time in intracranial aneurysm treatment. RoFo Fortschr Geb Rontgenstr Bildgebenden Verfahren. 2009;181(10):989-95.
938. Chalouhi N, Starke RM, Yang S, Bovenzi CD, Tjoumakaris S, Hasan D, et al. Extending the indications of flow diversion to small, unruptured, saccular aneurysms of the anterior circulation. Stroke. 2014;45(1):54-8.
939. Chung EJ, Shin YS, Lee CH, Song JH, Park JE. Comparison of clinical and radiologic outcomes among stent-assisted, double-catheter, and balloon-assisted coil embolization of wide neck aneurysms. Acta Neurochir. 2014;156(7):1289-95.
940. Claiborne Johnston S, Adams Dudley R, Gress DR, Ono L. Surgical and endovascular treatment of unruptured cerebral aneurysms at university hospitals. Neurology. 1999;52(9):1799-805.
941. Claiborne Johnston S, Zhao S, Adams Dudley R, Berman MF, Gress DR. Treatment of unruptured cerebral aneurysms in California. Stroke. 2001;32(3):[d]597-603.
942. Coley S, Sneade M, Clarke A, Mehta Z, Kallmes D, Cekirge S, et al. Cerecyte coil trial: Procedural safety and clinical outcomes in patients with ruptured and unruptured intracranial aneurysms. Am J Neuroradiol. 2012;33(3):474-80.
943. Consoli A, Vignoli C, Renieri L, Rosi A, Chiarotti I, Nappini S, et al. Assisted coiling of saccular wide-necked unruptured intracranial aneurysms: Stent versus balloon. J Neurointervent Surg. 2016;8(1):52-7.
944. Crobeddu E, Panciani PP, Garbossa D, Pilloni G, Fornaro R, Ronchetti G, et al. Cerebrovascular diseases in the elderly: The challenge of multiple aneurysms. Int J Neurosci. 2014;124(8):573-6.
945. Crocker M, Corns R, Hampton T, Deasy N, Tolias CM. Vascular neurosurgery following the International Subarachnoid Aneurysm Trial: Modern practice reflected by subspecialization. J Neurosurg. 2008;109(6):992-7.
946. Dabus G, Brinjikji W, Amar AP, Almandoz JED, Diaz OM, Jabbour P, et al. Angiographic and clinical outcomes of balloon remodeling versus unassisted coil embolization in the ruptured aneurysm cohort of the GEL the NEC study. J Neurointervent Surg. 2018;10(5):447-51.
947. Dengler J, Rüfenacht D, Meyer B, Rohde V, Endres M, Lenga P, et al. Giant intracranial aneurysms: Natural history and 1-year case fatality after endovascular or surgical treatment. J Neurosurg. 2021;134(1):49-57.
948. Derrey S, Penchet G, Thines L, Lonjon M, David P, Bataille B, et al. French collaborative group series on giant intracranial aneurysms: Current management. Neurochirurgie. 2015;61(6):371-7.

© 2023 Volovici V et al. *JAMA Network Open.*

949. Di RY, Ge L, Lu G, Huang L, Jiang YQ, Wan HL, et al. Clinical and angiographic outcomes of stent-assisted coiling of paraclinoid aneurysms: Comparison of LVIS and Neuroform stents. J Clin Neurosci. 2020.
950. Diaz OM, Rangel-Castilla L, Barber S, Mayo RC, Klucznik R, Zhang YJ. Middle cerebral artery aneurysms: A single-center series comparing endovascular and surgical treatment. World Neurosurg. 2014;81(2):322-9.
951. Dietrich P, Gravius A, Muhl-Benninghaus R, Yilmaz U, Kettner M, Bomberg H, et al. Single Center Experience in Stent-Assisted Coiling of Complex Intracranial Aneurysms Using Low-Profile Stents : The ACCLINO R Stent Versus the ACCLINO R Flex Stent. 2020.
952. Dong QL, Gao BL, Cheng ZR, He YY, Zhang XJ, Fan QY, et al. Comparison of surgical and endovascular approaches in the management of multiple intracranial aneurysms. Int J Surg. 2016;32:129-35.
953. Dumont AS, Crowley RW, Monteith SJ, Ilodigwe D, Kassell NF, Mayer S, et al. Endovascular treatment or neurosurgical clipping of ruptured intracranial aneurysms: Effect on angiographic vasospasm, delayed ischemic neurological deficit, cerebral infarction, and clinical outcome. Stroke. 2010;41(11):2519-24.
954. Durst CR, Khan P, Gaughen J, Patrie J, Starke RM, Conant P, et al. Direct comparison of Neuroform and Enterprise stents in the treatment of wide-necked intracranial aneurysms. Clin Radiol. 2014.
955. Enriquez-Marulanda A, Salem MM, Ascanio LC, Maragkos GA, Gupta R, Moore JM, et al. No differences in effectiveness and safety between pipeline embolization device and stent-assisted coiling for the treatment of communicating segment internal carotid artery aneurysms. Neuroradiol J. 2019.
956. Fan L, Tan X, Xiong Y, Zheng K, Li Z, Liu D, et al. Stent-assisted coiling versus coiling alone of ruptured anterior communicating artery aneurysms: A single-center experience. Clin Neurol Neurosurg. 2016;144:96-100.
957. Feng X, Tong X, Peng F, Wang K, Niu H, Qi P, et al. The Minimum Distance May Affect Perioperative Complications and Completed Occlusions of Endovascular Treatment for Tandem Intracranial Aneurysms: A Multi-Institutional Retrospective Study. Cerebrovasc Dis. 2020.
958. Frontera JA, Moatti J, De Los Reyes KM, McCullough S, Moyle H, Bederson JB, et al. Safety and cost of stent-assisted coiling of unruptured intracranial aneurysms compared with coiling or clipping. J Neurointervent Surg. 2014;6(1):65-71.
959. Gaba RC, Ansari SA, Roy SS, Marden FA, Viana MAG, Malisch TW. Embolization of intracranial aneurysms with hydrogel-coated coils versus inert platinum coils: Effects on packing density, coil length and quantity, procedure performance, cost, length of hospital stay, and durability of therapy. Stroke. 2006;37(6):1443-50.
960. Ge H, Lv X, Yang X, He H, Jin H, Li Y. LVIS Stent Versus Enterprise Stent for the Treatment of Unruptured Intracranial Aneurysms. World Neurosurg. 2016;91:365-70.
961. Gentric JC, Biondi A, Piotin M, Mounayer C, Lobotesis K, Bonafé A, et al. Balloon remodeling may improve angiographic results of stent-assisted coiling of unruptured intracranial aneurysms. Neurosurgery. 2015;76(4):441-5.
962. Gerlach R, Beck J, Setzer M, Vatter H, Berkefeld J, De Rochemont RDM, et al. Treatment related morbidity of unruptured intracranial aneurysms: Results of a prospective single centre series with an interdisciplinary approach over a 6 year period (1999-2005). J Neurol Neurosurg Psychiatry. 2007;78(8):864-71.
963. Sanai N, Quinones-Hinojosa A, Gupta NM, Perry V, Sun PP, Wilson CB, et al. Pediatric intracranial aneurysms: Durability of treatment following microsurgical and endovascular management. J Neurosurg. 2006;104 PEDIATRICS(SUPPL. 2):82-9.
964. Sauvigny T, Nawka MT, Schweingruber N, Mader MMD, Regelsberger J, Schmidt NO, et al. Early clinical course after aneurysmal subarachnoid hemorrhage: comparison of patients treated with Woven

EndoBridge, microsurgical clipping, or endovascular coiling. Acta Neurochir. 2019.
965. Schwyzer L, Soleman E, Ensner R, Mironov A, Landolt H, Fandino J. Quality of life and outcome after treatment of ruptured cerebral aneurysms: Results of a single center in switzerland. Acta Neurochir Suppl2015. p. 197-201.
966. Starke RM, Durst CR, Evans A, Ding D, Raper DMS, Jensen ME, et al. Endovascular treatment of unruptured wide-necked intracranial aneurysms: Comparison of dual microcatheter technique and stent-assisted coil embolization. J Neurointervent Surg. 2015;7(4):256-61.
967. Steklacova A, Bradac O, Charvat F, De Lacy P, Benes V. "Clip first" policy in management of intracranial MCA aneurysms: Single-centre experience with a systematic review of literature. Acta Neurochir. 2016;158(3):533-46.
968. Sweid A, Atallah E, Herial N, Saad H, Mouchtouris N, Barros G, et al. Pipeline-assisted coiling versus pipeline in flow diversion treatment of intracranial aneurysms. J Clin Neurosci. 2018;58:20-4.
969. Taheri Z, Harirchian MH, Ghanaati H, Khoshnevisan A, Salamati P, Miri M, et al. Comparison of endovascular coiling and surgical clipping for the treatment of intracranial aneurysms: A prospective study. Iran J Neurol. 2014;14(1):22-8.
970. Taschner CA, Thines L, El-Mahdy M, Rachdi H, Gauvrit JY, Lejeune JP, et al. GDC 360° for the endovascular treatment of intracranial aneurysms: A matched-pair study analysing angiographic outcomes with GDC 3D Coils in 38 patients. Neuroradiology. 2009;51(1):45-52.
971. Tsukahara T, Murakami N, Sakurai Y, Yonekura M, Takahashi T, Inoue T, et al. Treatment of unruptured cerebral aneurysms; a multi-center study at Japanese national hospitals. Acta Neurochir Suppl. 2005;94:77-85.
972. Vakharia K, Waqas M, Shakir HJ, Chin F, Hartke JN, Shallwani H, et al. Versatile use of catheter systems for deployment of the Pipeline embolization device: A comparison of biaxial and triaxial catheter systems. J Neurointervent Surg. 2020;12(6):585-90.
973. Van Rooij WJ, De Gast AN, Sluzewski M. Results of 101 aneurysms treated with polyglycolic/polylactic acid microfilament nexus coils compared with historical controls treated with standard coils. Am J Neuroradiol. 2008;29(5):991-6.
974. Vergouwen MDI, Fang J, Casaubon LK, Stamplecoski M, Robertson A, Kapral MK, et al. Higher incidence of in-hospital complications in patients with clipped versus coiled ruptured intracranial aneurysms. Stroke. 2011;42(11):3093-8.
975. Ye HW, Liu YQ, Wang QJ, Zheng T, Cui XB, Gao YY, et al. Comparison between solitaire™ AB and enterprise stent-assisted coiling for intracranial aneurysms. Exp Ther Med. 2015;10(1):145-53.
976. Zaidat OO, Ionita CC, Hussain SI, Alexander MJ, Friedman AH, Graffagnino C. Impact of ruptured cerebral aneurysm coiling and clipping on the incidence of cerebral vasospasm and clinical outcome. J Neuroimaging. 2009;19(2):144-9.
977. Zaidat OO, Castonguay AC, Rai AT, Badruddin A, Mack WJ, Alshekhlee AK, et al. TARGET Intracranial Aneurysm Coiling Prospective Multicenter Registry: Final Analysis of Peri-Procedural and Long-Term Safety and Efficacy Results. Front Neurol. 2019;10:737.
978. Zanaty M, Chalouhi N, Starke RM, Daou B, Todd M, Bayman E, et al. Short-Term Outcome of Clipping Versus Coiling of Ruptured Intracranial Aneurysms Treated by Dual-Trained Cerebrovascular Surgeon: Single-Institution Experience. World Neurosurg. 2016;95:262-9.
979. Zhai XD, Li CJ, Yu JX, He C, Ye M, Hu P, et al. Microsurgical and Endovascular Treatment Outcomes in Pericallosal Artery Aneurysms: A Single Center Retrospective Analysis. Turk Neurosurg. 2020;30(2):285-92.
980. Zhao B, Tan X, Yang H, Zheng K, Li Z, Xiong Y, et al. Stent-assisted coiling versus coiling alone of poor-grade ruptured intracranial aneurysms: A multicenter study. J Neurointervent Surg. 2017;9(2):165-8.
981. Zhao B, Xing H, Fan L, Tan X, Zhong M, Pan Y, et al. Endovascular Coiling versus Surgical Clipping of Very Small Ruptured Anterior Communicating Artery Aneurysms. World Neurosurg. 2019;126:e1246-

© 2023 Volovici V et al. *JAMA Network Open.*

e50.
982. Zuo Q, Yang P, Lv N, Huang Q, Zhou Y, Zhang X, et al. Safety of coiling with stent placement for the treatment of ruptured wide-necked intracranial aneurysms: A contemporary cohort study in a high-volume center after improvement of skills and strategy. J Neurosurg. 2019;131(2):435-41.
983. Yu SCH, Wong GKC, Wong JKT, Poon WS. Endovascular coiling versus neurosurgical clipping for ruptured intracranial aneurysms: Significant benefits in clinical outcome and reduced consumption of hospital resources in Hong Kong Chinese patients. Hong Kong Med J. 2007;13(4):271-8.
984. Sharma M, Brown B, Madhugiri V, Cuellar-Saenz H, Sonig A, Ambekar S, et al. Unruptured intracranial aneurysms: Comparison of perioperative complications, discharge disposition, outcome, and effect of calcification, between clipping and coiling: A single institution experience. Neurol India. 2013;61(3):270-6.
985. Silva MA, See AP, Khandelwal P, Mahapatra A, Frerichs KU, Du R, et al. Comparison of flow diversion with clipping and coiling for the treatment of paraclinoid aneurysms in 115 patients. J Neurosurg. 2019;130(5):1505-12.
986. Song J, Park J, Chung J, Lim Y, Shin Y. Treatment strategies of ruptured posterior inferior cerebellar artery aneurysm according to its segment. Surg Neurol Intl. 2017;8(1).
987. Spetzler RF, McDougall CG, Zabramski JM, Albuquerque FC, Hills NK, Nakaji P, et al. Ten-year analysis of saccular aneurysms in the Barrow Ruptured Aneurysm Trial. J Neurosurg. 2020;132(3):771-6.
988. Starke RM, Chalouhi N, Ali MS, Tjoumakaris SI, Jabbour PM, Fernando Gonzalez L, et al. Endovascular treatment of carotid cavernous aneurysms: complications, outcomes and comparison of interventional strategies. J Clin Neurosci. 2014;21(1):40-6.
989. Vrsajkov V, Kolak R, Uram-Benka A, Uvelin A, Kiselicki J. Anesthesia, complications, and clinical outcome for ruptured intracranial aneurysms: A retrospective comparison between endovascular coiling and neurosurgical clipping. Turk J Med Sci. 2012;42(3):477-83.
990. Vanzin JR, Mounayer C, Piotin M, Spelle L, Boissonnet H, Moret J. [Endovascular treatment of unruptured middle cerebral artery aneurysms]. J Neuroradiol. 2005;32(2):97-108.
991. Chen F, Fang X. Endovascular treatment of middle cerebral artery aneurysm with a (Lvis) device: comparison of lvis stent and non-lvis stent. Exp Ther Med. 2019;17(3).
992. Taweesomboonyat C, Tunthanathip T, Kaewborisutsakul A, Saeheng S, Oearsakul T, Riabroi K, et al. Outcome of Ruptured Posterior Communicating Artery Aneurysm Treatment Comparing Between Clipping and Coiling Techniques. World Neurosurg. 2019;125:e183-e8.
993. Xie Q, Gu Y, Song Y, Leng B, Zheng Y, Xu F. One-stage coiling versus clipping of multiple intracranial aneurysms in elderly patients. Clin Neurol Neurosurg. 2021;201.
994. Zaeske C, Goertz L, Dorn F, Turowski B, Abdullayev N, Schlamann M, et al. Comparative Analysis of the Pipeline and the Derivo Flow Diverters for the Treatment of Unruptured Intracranial Aneurysms—A Multicentric Study. World Neurosurg. 2021;145:e326-e31.
995. Wadd IH, Haroon A, Habibullah, Ansari S, Mukhtar S, Rashid U, et al. Aneurysmal subarachnoid hemorrhage: Outcome of aneurysm clipping versus coiling in anterior circulation aneurysm. J Coll Phys Surg Pak. 2015;25(11):798-801.
996. Lu P, Zhang Y, Niu H, Wang Y. Comparison of endovascular treatment for middle cerebral artery aneurysm with a low-profile visualized intraluminal support stent or pipeline embolization device. Exp Ther Med. 2019;18(3):2072-8.
997. Lubicz B, Lefranc F, Bruneau M, Balériaux D, Witte OD. Balloon-assisted coiling of intracranial aneurysms is not associated with a higher complication rate. Neuroradiology. 2008;50(9):769-76.
998. Lusseveld E, Brilstra EH, Nijssen PC, van Rooij WJ, Sluzewski M, Tulleken CA, et al. Endovascular coiling versus neurosurgical clipping in patients with a ruptured basilar tip aneurysm. J Neurol Neurosurg Psychiatry. 2002;73(5):591-3.
999. Lv Z, Zhu Y, Wang W, Wu Q, Li W, Li Q, et al. Comparison of Two Endovascular Interventions with

Low-Profile Visualized Intraluminal Support or Pipeline Embolization Device in Middle Cerebral Arterial Aneurysms Patients. J Invest Surg. 2019.
1000. Makhambetov Y, Kaliyev A, Kikuta KI, Smagulov F, Medetov Y, Kulmirzayev M, et al. Early and midterm results of treatment of giant internal carotid artery paraclinoid aneurysms with trapping and flow diverters. Acta Neurochir (Wien). 2019;161(9):1755-61.
1001. Manabe H, Hasegawa S, Takemura A. The role of GDC embolization as a second choice in the treatment of ruptured cerebral aneurysm: Retrospective analysis from mid-term outcome. Intervent Neuroradiol. 2003;9(SUPPL. 1):41-6.
1002. Manabe H, Takemura A, Hasegawa S, Nagahata M, Islam S. The choice of treatment of method for unruptured cerebral aneurysm. Investigation from clinical outcome, angiographical result, duration of hospital stay, and cost for treatment. Intervent Neuroradiol. 2004;10(SUPPL. 1):143-6.
1003. Maria FD, Pistocchi S, Clarencon F, Bartolini B, Blanc R, Biondi A, et al. Flow diversion versus standard endovascular techniques for the treatment of unruptured carotid-ophthalmic aneurysms. Am J Neuroradiol. 2015;36(12):2325-30.
1004. Mascitelli JR, Gandhi S, Meybodi AT, Lawton MT. The oculomotor-tentorial triangle. Part 2: A microsurgical workspace for vascular lesions in the crural and ambient cisterns. J Neurosurg. 2019;130(5):1435-45.
1005. McDonald JS, McDonald RJ, Fan J, Kallmes DF, Lanzino G, Cloft HJ. Comparative effectiveness of unruptured cerebral aneurysm therapies: Propensity score analysis of clipping versus coiling. Stroke. 2013;44(4):988-94.
1006. McDougall CG, Johnston SC, Gholkar A, Barnwell SL, Suarez JCV, Romero JM, et al. Bioactive versus bare platinum coils in the treatment of intracranial aneurysms: The MAPS (matrix and platinum science) trial. Am J Neuroradiol. 2014;35(5):935-42.
1007. McDougall CG, Johnston SC, Hetts SW, Gholkar A, Barnwell SL, Vazquez Suarez JC, et al. Five-year results of randomized bioactive versus bare metal coils in the treatment of intracranial aneurysms: The Matrix and Platinum Science (MAPS) Trial. J Neurointervent Surg. 2020.
1008. McDougall CG, Johnston SC, Gholkar A, Turk AS. Bioactive vs. Bare platinum coils: the MAPS 5 year results. Interventional neuroradiology. 2015;21:184-5.
1009. Milburn J, Pansara AL, Vidal G, Martinez RC. Initial experience using the Penumbra coil 400: Comparison of aneurysm packing, cost effectiveness, and coil efficiency. J Neurointervent Surg. 2014;6(2):121-4.
1010. Mohammad F, Horiguchi T, Mizutani K, Yoshida K. Clipping versus coiling in unruptured anterior cerebral circulation aneurysms. Surg Neurol Intl. 2020;11(50).
1011. Mokin M, Primiani CT, Ren Z, Piper K, Fiorella DJ, Rai AT, et al. Stent-assisted coiling of cerebral aneurysms: Multi-center analysis of radiographic and clinical outcomes in 659 patients. J Neurointervent Surg. 2020;12(3):289-97.
1012. Moon JS, Choi CH, Lee TH, Ko JK. Result of coiling versus clipping of unruptured anterior communicating artery aneurysms treated by a hybrid vascular neurosurgeon. J cerebrovasc endovasc neurosurg. 2020;22(4):225-36.
1013. Moret J, Cognard C, Weill A, Castaings L, Rey A. The 'remodelling technique' in the treatment of wide neck intracranial aneurysms: Angiographic results and clinical follow-up in 56 cases. INTERVENT NEURORADIOL. 1997;3(1):21-35.
1014. Mortimer AM, Bradford C, Steinfort B, Faulder K, Assaad N, Harrington T. Short term outcomes following clipping and coiling of ruptured intracranial aneurysms: Does some of the benefit of coiling stem from less procedural impact on deranged physiology at presentation? J Neurointervent Surg. 2016;8(2):145-51.
1015. Murphy M, Bell D, Worth RD, Jehle KS, Critchley GR, Norris JS. Angiography postclipping and coiling of cerebral aneurysms. Br J Neurosurg. 2005;19(3):225-8.

© 2023 Volovici V et al. *JAMA Network Open.*

1016. Cho WC, Shin YS, Kim BS, Choi JH. Treatment outcome after coiling or clipping for elderly patients with unruptured intracranial aneurysms. J cerebrovasc endovasc neurosurg. 2020;22(2):78-84.
1017. Miyachi S, Negoro M, Okamoto T, Suzuki O, Yoshida J. Endovascular treatment of unruptured vertebro-basilar aneurysms. Intervent Neuroradiol. 1999;5(SUPPL. 1):83-8.
1018. Monteiro A, Cortez GM, Aghaebrahim A, Sauvageau E, Hanel RA. Low-Profile Visualized Intraluminal Support Jr Braided Stent Versus Atlas Self-Expandable Stent for Treatment of Intracranial Aneurysms: A Single Center Experience. Neurosurgery. 2020.
1019. Moon K, Levitt MR, Almefty RO, Nakaji P, Albuquerque FC, Zabramski JM, et al. Treatment of Ruptured Anterior Communicating Artery Aneurysms: Equipoise in the Endovascular Era? Neurosurgery. 2015;77(4):566-71.
1020. McDonald JS, McDonald RJ, Fan J, Kallmes DF, Lanzino G, Cloft HJ. Comparative effectiveness of ruptured cerebral aneurysm therapies: Propensity score analysis of clipping versus coiling. Am J Neuroradiol. 2014;35(1):164-9.
1021. McDougall CG, Spetzler RF, Zabramski JM, Partovi S, Hills NK, Nakaji P, et al. The barrow ruptured aneurysm trial: Clinical article. J Neurosurg. 2012;116(1):135-44.
1022. Mascitelli JR, Patel AB, Polykarpou MF, Patel AA, Moyle H. Analysis of early angiographic outcome using unique large diameter coils in comparison with standard coils in the embolization of cerebral aneurysms: A retrospective review. J Neurointervent Surg. 2015;7(2):126-30.
1023. Mascitelli JR, Polykarpou MF, Patel AA, Kamath AA, Moyle H, Patel AB. Initial experience with Penumbra Coil 400 versus standard coils in embolization of cerebral aneurysms: A retrospective review. J Neurointervent Surg. 2013;5(6):573-6.
1024. Molyneux AJ, Birks J, Clarke A, Sneade M, Kerr RSC. The durability of endovascular coiling versus neurosurgical clipping of ruptured cerebral aneurysms: 18 year follow-up of the UK cohort of the International Subarachnoid Aneurysm Trial (ISAT). Lancet. 2015;385(9969):691-7.
1025. Molyneux A, Kerr R, Stratton I, Sandercock P, Clarke M, Shrimpton J, et al. International Subarachnoid Aneurysm Trial (ISAT) of neurosurgical clipping versus endovascular coiling in 2143 patients with ruptured intracranial aneurysms: A randomised trial. Lancet. 2002;360(9342):1267-74.
1026. Choi JH, Park JE, Kim MJ, Kim BS, Shin YS. Aneurysmal neck clipping as the primary treatment option for both ruptured and unruptured middle cerebral artery aneurysms. J Korean Neurosurg Soc. 2016;59(3):269-75.
1027. Cho WS, Hong HS, Kang HS, Kim JE, Cho YD, Kwon OK, et al. Stability of Cerebral Aneurysms after Stent-Assisted Coil Embolization: A Propensity Score-Matched Analysis. Neurosurgery. 2015;77(2):208-16.
1028. Goertz L, Brinker G, Hamisch C, Kabbasch C, Borggrefe J, Hof M, et al. Elective Treatment of Additional and Recurrent Aneurysms in Patients with a Previous Subarachnoid Hemorrhage: A Single-Center Analysis of Complications and Clinical Outcome. World Neurosurg. 2019;125:e1196-e202.
1029. Goertz L, Liebig T, Siebert E, Herzberg M, Pennig L, Schlamann M, et al. Low-profile intra-aneurysmal flow disruptor WEB 17 versus WEB predecessor systems for treatment of small intracranial aneurysms: Comparative analysis of procedural safety and feasibility. Am J Neuroradiol. 2019;40(10):1766-72.
1030. Gordhan A, Invergo D. Stent-assisted aneurysm coil embolization: Safety and efficacy at a low-volume center. Neurol Res. 2011;33(9):942-6.
1031. Griessenauer CJ, Enriquez-Marulanda A, Xiang S, Hong T, Zhang H, Taussky P, et al. Comparison of ped and fred flow diverters for posterior circulation aneurysms: a propensity score matched cohort study. J Neurointervent Surg. 2020.
1032. Guo XB, Fan YM, Zhang JN. HydroSoft coil versus HydroCoil for endovascular aneurysm occlusion study: A single center experience. Eur J Radiol. 2011;79(2):e42-e6.
1033. Hadjivassiliou M, Tooth CL, Romanowski CAJ, Byrne J, Battersby RDE, Oxbury S, et al. Aneurysmal SAH: Cognitive outcome and structural damage after clipping or coiling. Neurology. 2001;56(12):1672-7.

© 2023 Volovici V et al. *JAMA Network Open.*

1034. Hammer A, Steiner A, Kerry G, Ranaie G, Yakubov E, Lichtenstern D, et al. Efficacy and Safety of Treatment of Ruptured Intracranial Aneurysms. World Neurosurg. 2017;98:780-9.
1035. Hammer A, Steiner A, Kerry G, Ranaie G, Baer I, Hammer CM, et al. Treatment of ruptured intracranial aneurysms yesterday and now. PLoS ONE. 2017;12(3).
1036. Helland CA, Kråkenes J, Moen G, Wester K. A population-based study of neurosurgical and endovascular treatment of ruptured, intracranial aneurysms in a small neurosurgical unit. Neurosurgery. 2006;59(6):1168-75.
1037. Higashida RT, Lahue BJ, Torbey MT, Hopkins LN, Leip E, Hanley DF. Treatment of unruptured intracranial aneurysms: a nationwide assessment of effectiveness. AJNR Am J Neuroradiol. 2007;28(1):146-51.
1038. Hoh BL, Topcuoglu MA, Singhal AB, Pryor JC, Rabinov JD, Rordorf GA, et al. Effect of clipping, craniotomy, or intravascular coiling on cerebral vasospasm and patient outcome after aneurysmal subarachnoid hemorrhage. Neurosurgery. 2004;55(4):779-89.
1039. Hohlrieder M, Spiegel M, Hinterhoelzl J, Engelhardt K, Pfausler B, Kampfl A, et al. Cerebral vasospasm and ischaemic infarction in clipped and coiled intracranial aneurysm patients. Eur J Neurol. 2002;9(4):389-99.
1040. Horcajadas A, Ortiz I, Jorques AM, Katati MJ. Clinical results and costs of endovascular treatment in comparison with surgery in non ruptured aneurysms. Neurocirugia. 2018;29(6):267-74.
1041. Huang C, You C. Clipping Versus Coiling in the Management of Unruptured Aneurysms with Multiple Risk Factors. World Neurosurg. 2019;126:e545-e9.
1042. Hui FK, Schuette AJ, Moskowitz SI, Spiotta AM, Lieber ML, Rasmussen PA, et al. Microsurgical and endovascular management of pericallosal aneurysms. J Neurointervent Surg. 2011;3(4):319-23.
1043. Hwang US, Shin HS, Lee SH, Koh JS. Decompressive Surgery in Patients with Poor-grade Aneurysmal Subarachnoid Hemorrhage: Clipping with Simultaneous Decompression Versus Coil Embolization Followed by Decompression. J cerebrovasc endovasc neurosurg. 2014;16(3):254-61.
1044. Iihara K, Murao K, Sakai N, Shindo A, Sakai H, Higashi T, et al. Unruptured paraclinoid aneurysms: A management strategy. J Neurosurg. 2003;99(2):241-7.
1045. Ikawa F, Abiko M, Ishii D, Ohshita J, Matsushige T, Okazaki T, et al. Analysis of outcome at discharge after aneurysmal subarachnoid hemorrhage in Japan according to the Japanese stroke databank. Neurosurg Rev. 2018;41(2):567-74.
1046. Ikawa F, Michihata N, Akiyama Y, Iihara K, Matano F, Morita A, et al. Treatment Risk for Elderly Patients with Unruptured Cerebral Aneurysm from a Nationwide Database in Japan. World Neurosurg. 2019;132:e89-e98.
1047. Inamasu J, Tanaka T, Sadato A, Hayakawa M, Adachi K, Hayashi T, et al. Outcomes of surgical versus endovascular repair of unruptured brain aneurysms in individuals aged ≥75 years. Geriatr Gerontol Int. 2014;14(4):858-63.
1048. Park JS, Kwon MY, Lee CY. Minipterional craniotomy for surgical clipping of anterior circulation aneurysms: compatibility between the feasibility, safety and efficiency. J cerebrovasc endovasc neurosurg. 2020;22(2):65-77.
1049. Peng H, Tang X, Yang B, Sun H, Zhao L, Zhang T. Comparative research on clamping and interventional embolization on aneurysmal subarachnoid hemorrhage patients and effects on prognosis. Int J Clin Exp Med. 2019;12(3):2435-42.
1050. Pierot L, Moret J, Turjman F, Herbreteau D, Raoult H, Barreau X, et al. WEB Treatment of Intracranial Aneurysms: Feasibility, Complications, and 1-Month Safety Results with the WEB DL and WEB SL/SLS in the French Observatory. AJNR Am J Neuroradiol. 2015;36(5):922-7.
1051. Pilipenko Iu V, Eliava S, Iakovlev SB, Belousova OB, Buklina SB, Arustomian SR, et al. [The analysis of complications of surgical treatment of brain aneurysms in patients operated on in the late posthemorrhagic period]. Zh Vopr Neirokhir Im N N Burdenko. 2014;78(2):32-8; discussion 8-9.

© 2023 Volovici V et al. *JAMA Network Open.*

1052. Regli L, Dehdashti AR, Uske A, de Tribolet N. Endovascular coiling compared with surgical clipping for the treatment of unruptured middle cerebral artery aneurysms: an update. Acta Neurochir Suppl. 2002;82:41-6.
1053. Roh H, Kim J, Bae H, Chong K, Kim JH, Suh SI, et al. Comparison of stent-assisted and no-stent coil embolization for safety and effectiveness in the treatment of ruptured intracranial aneurysms. J Neurosurg. 2020;133(3):814-20.
1054. Salahuddin H, Siddiqui NS, Castonguay AC, Johnson M, Zaidi SF, Jumaa MA. Recent Trends in Electively Treated Unruptured Intracranial Aneurysms. J Stroke Cerebrovasc Dis. 2019;28(7):2011-7.
1055. Salem MM, Ravindran K, Enriquez-Marulanda A, Ascanio LC, Jordan N, Gomez-Paz S, et al. Pipeline embolization device versus stent-assisted coiling for intracranial aneurysm treatment: A retrospective propensity score-matched study. Neurosurgery. 2020;87(3):516-22.
1056. Binboga AB, Onay M, Altay CM. Strut remodeling with hypercompliant balloon: A new approach to Y stent-assisted coil embolization in the treatment of complex wide-neck bifurcation aneurysms. Interv neuroradiol. 2021;27(3):329-38.
1057. Bonafe A, Perez MA, Henkes H, Lylyk P, Bleise C, Gascou G, et al. Diversion-p64: results from an international, prospective, multicenter, single-arm post-market study to assess the safety and effectiveness of the p64 flow modulation device. J Neurointerv Surg. 2021.
1058. Boulouis G, Soize S, Maus V, Fischer S, Lobsien D, Klisch J, et al. Flow diversion for internal carotid artery aneurysms with compressive neuro-ophthalmologic symptoms: clinical and anatomical results in an international multicenter study. J Neurointerv Surg. 2021.
1059. Brzegowy P, Polak J, Wnuk J, Łasocha B, Kwinta B, Urbanik A, et al. Endovascular treatment of middle cerebral artery aneurysms – single-centre results. Pol J Radiol. 2020;85(1):e650-e6.
1060. Byun J, Park W, Park JC, Ahn JS. Clinical outcomes of large (>10 mm) unruptured posterior circulation aneurysms and their predictors. J Korean Neurosurg Soc. 2021;64(1):39-50.
1061. Chacón-Quesada T, Mielke D, Rohde V, Hernández-Durán S. Microsurgical clipping vs Woven EndoBridge (WEB) device for the management of unruptured wide-neck bifurcation aneurysms. Neurosurg Rev. 2022.
1062. Chagas Lourenco G, Dantas F, Silva ECT, Firmino RUR, Quesado Filgueiras Filho M, Tosello RT, et al. Endovascular treatment of intracranial aneurysms using the Derivo Embolization Device: a multicenter experience. 2022.
1063. Cherian J, Chen SR, Puri A, Vakharia K, Levy E, Eshraghi S, et al. Postmarket American Experience with Woven EndoBridge Device: Adjudicated Multicenter Case Series. Neurosurgery. 2021;89(2):275-82.
1064. Achey RL, Winkelman R, Sheikhi L, Davison M, Toth G, Moore NZ, et al. Use of Surpass Streamline Flow Diverter for the Endovascular Treatment of Craniocervical Aneurysms: A Single-Institution Experience. World Neurosurg. 2022;162:e281-e7.
1065. Choi JH, Sim SY, Shin YS, Chung J. A Single Flow Re-direction Endoluminal Device for the Treatment of Large and Giant Anterior Circulation Intracranial Aneurysms. Yonsei Med J. 2022;63(4):349-56.
1066. Cler SJ, Lauzier DC, Chatterjee AR, Osbun JW, Moran CJ, Kansagra AP. Comparative study of on-label versus off-label treatment of intracranial aneurysms with the Pipeline embolization device. J Neurosurg. 2022:1-6.
1067. Cohen JE, Gomori JM, Moscovici S, Kaye AH, Shoshan Y, Spektor S, et al. Flow-diverter stents in the early management of acutely ruptured brain aneurysms: effective rebleeding protection with low thromboembolic complications. J Neurosurg. 2021:1-8.
1068. Cortez GM, Akture E, Monteiro A, Arthur AS, Peterson J, Dornbos D, et al. Woven EndoBridge device for ruptured aneurysms: Perioperative results of a US multicenter experience. J Neurointervent Surg. 2021.
1069. Cui R, Yan L, Kang K, Yang M, Yu Y, Mo D, et al. Long-Term Outcome of Enterprise Stenting for Symptomatic ICAS in a High-Volume Stroke Center. Front Neurol. 2021;12.
1070. Dakay K, Cooper JB, Greisman JD, Kaur G, Al-Mufti F, Gandhi CD, et al. Flow diversion in anterior cerebral artery aneurysms. Brain circ. 2021;7(4):247-52.
1071. Daou BJ, Palmateer G, Linzey JR, Thompson BG, Chaudhary N, Gemmete JJ, et al. Stent-assisted coiling of cerebral aneurysms: Head to head comparison between the Neuroform Atlas and EZ

stents. Intervent Neuroradiol. 2021;27(3):353-61.
1072. De Beule T, Boulanger T, Heye S, van Rooij WJ, van Zwam W, Stockx L. The Woven EndoBridge for unruptured intracranial aneurysms: Results in 95 aneurysms from a single center. Intervent Neuroradiol. 2021;27(5):594-601.
1073. De Leacy R, Bageac DV, Siddiqui N, Bellon RJ, Park MS, Schirmer CM, et al. Safety and Long-Term Efficacy Outcomes for Endovascular Treatment of Wide-Neck Bifurcation Aneurysms of the Middle Cerebral Artery: Insights From the SMART Registry. Front Neurol. 2022;13.
1074. De Vries J, Boogaarts HD, Sørensen L, Holtmannspoetter M, Benndorf G, Turowski B, et al. ECLIPs bifurcation remodeling system for treatment of wide neck bifurcation aneurysms with extremely low dome-to-neck and aspect ratios: A multicenter experience. J Neurointervent Surg. 2021;13(5):438-42.
1075. Diestro JDB, Adeeb N, Dibas M, Boisseau W, Harker P, Brinjikji W, et al. Flow Diversion for Middle Cerebral Artery Aneurysms: An International Cohort Study. Neurosurgery. 2021;89(6):1112-21.
1076. Dellaretti M, do Nascimento LM, de Oliveira Lima AD, de Almeida JC, Quadros RS. Efficacy and safety of surgical treatment for middle cerebral artery aneurysms: A retrospective case series. Interdiscip Neurosurg Adv Tech Case Manage. 2021;23.
1077. Dietrich P, Gravius A, Mühl-Benninghaus R, Yilmaz U, Kettner M, Bomberg H, et al. Single Center Experience in Stent-Assisted Coiling of Complex Intracranial Aneurysms Using Low-Profile Stents: The ACCLINO® Stent Versus the ACCLINO® Flex Stent. Clin Neurorad. 2021;31(1):99-106.
1078. Dutta G, Singh D, Jagetia A, Srivastava AK, Singh H, Kumar A. Endovascular management of large and giant intracranial aneurysms: Experience from a tertiary care neurosurgery institute in India. J cerebrovasc endovasc neurosurg. 2021;23(2):99-107.
1079. Elian MMM, Issa ASI, Ibrahim MA, Khalil OAEW, Youssef FH. Endovascular management of middle cerebral artery aneurysms (single-center case series study). Egypt J Radiol Nucl Med. 2021;52(1).
1080. Elsheikh S, Möhlenbruch M, Seker F, Berlis A, Maurer C, Kocer N, et al. Flow Diverter Treatment of Ruptured Basilar Artery Perforator Aneurysms: A Multicenter Experience. Clin Neurorad. 2022.
1081. Enriquez-Marulanda A, Penumaka A, Ogilvy CS, Thomas AJ, Moore JM. Safety and Efficacy of the Off-Label Use of Pipeline Embolization Device Based on the 2018 Food and Drug Administration-Approved Indications for Intracranial Aneurysms: A Single-Center Retrospective Cohort Study. Neurosurgery. 2022;90(6):700-7.
1082. Feng SW, Luo CB, Lee CH, Chang FC, Lin CJ. Flow-diverter stent to manage intracranial aneurysms: A single center experience. J Chin Med Assoc. 2022;85(3):358-63.
1083. Fujii T, Teranishi K, Yatomi K, Suzuki K, Mitome-Mishima Y, Kondo A, et al. Long-term Follow-up Results after Flow Diverter Therapy Using the Pipeline Embolization Device for Large or Giant Unruptured Internal Carotid Artery Aneurysms: Single-center Retrospective Analysis in the Japanese Population. Neurol Med -Chir. 2022;62(1):19-27.
1084. Gajera J, Maingard J, Foo M, Ren Y, Lamanna A, Nour D, et al. The Woven EndoBridge Device for the Treatment of Intracranial Aneurysms: Initial Clinical Experience within an Australian Population. Neurointervention. 2022;17(1):28-36.
1085. Zimmer S, Maus V, Maurer C, Berlis A, Weber W, Fischer S. Widening the indications for intrasaccular flow disruption: WEB 17 in the treatment of aneurysm locations different from those in the good clinical practice trials. Am J Neuroradiol. 2021;42(3):524-9.
1086. Zhong W, Kuang H, Zhang P, Yang X, Luo B, Maimaitili A, et al. Pipeline Embolization Device for the Treatment of Ruptured Intracerebral Aneurysms: A Multicenter Retrospective Study. Front Neurol. 2021;12.
1087. Aihara M, Shimizu T, Naito I, Miyamoto N, Yamaguchi R, Aishima K, et al. Endovascular Treatment Strategy and Clinical Outcomes for Ruptured Blood Blister-Like Aneurysms of the Internal Carotid Artery Using Low-Profile Visualized Intraluminal Support Stent. World Neurosurg. 2021;149:e146-e53.
1088. Alpay K, Nania A, Parkkola R, Downer J, Lindgren A, Rautio R. The outcomes of recurrent wide-necked intracranial aneurysms treated with the Woven EndoBridge (WEB): A retrospective bicenter study. J Neuroradiol. 2022;49(3):298-304.
1089. Aydin K, Berdikhojayev M, Cay F, Barburoglu M, Nurzhan S, Aygun S, et al. Safety, Efficacy, and Durability of Stent-Assisted Coiling Treatment of M2 (Insular) Segment MCA Aneurysms. Am J Neuroradiol. 2022;43(4):560-7.
1090. De Beule T, Boulanger T, Heye S, van Rooij WJ, van Zwam WH, Stockx L. p64 flow diverter: Results in 108 patients from a single center. Intervent Neuroradiol. 2020.

© 2023 Volovici V et al. *JAMA Network Open.*

1091. Deuschl C, Oppong MD, Styczen H, Markhardt L, Wrede K, Jabbarli R, et al. Therapy results of pericallosal aneurysms: A retrospective unicenter study. Clin Pract. 2020;10(3):66-9.
1092. Lu J, Li M, Zhao Y, Zhao Y, Chen X, Zhao J. Paediatric Intracranial Aneurysms: Long-term Angiographic and Clinical Outcomes in a Contemporary Series. Front Neurol. 2022;13.
1093. Luzzi S, Del Maestro M, Galzio R. Posterior Circulation Aneurysms: A Critical Appraisal of a Surgical Series in Endovascular Era. Acta Neurochir Suppl. 2021;132:39-45.
1094. Lylyk I, Scrivano E, Lundquist J, Ferrario A, Bleise C, Perez N, et al. Pipeline Embolization Devices for the Treatment of Intracranial Aneurysms, Single-Center Registry: Long-Term Angiographic and Clinical Outcomes from 1000 Aneurysms. Neurosurgery. 2021;89(3):443-9.
1095. MacDonell J, Field NC, Entezami P, Yamamoto J, Boulos AS, Dalfino JC, et al. Comparison of hydrogel coils versus bare platinum coils for the treatment of anterior communicating artery aneurysms. Brain circ. 2022;8(1):6-9.
1096. Church EW, Bigder MG, Sussman ES, Gummidipundi SE, Han SS, Heit JJ, et al. Treatment of posterior circulation fusiform aneurysms. J Neurosurg. 2021;134(6):1894-900.
1097. Feigen CM, Vivanco-Suarez J, Javed K, Dardick JM, Holland R, Mendez-Ruiz A, et al. Pipeline Embolization Device and Pipeline Flex Versus Surpass Streamline Flow Diversion in Intracranial Aneurysms: A Retrospective Propensity Score–Matched Study. World Neurosurg. 2022;161:e384-e94.
1098. Ge H, Chen X, Liu K, Zhao Y, Zhang L, Liu P, et al. Endovascular Treatment of Large or Giant Basilar Artery Aneurysms Using the Pipeline Embolization Device: Complications and Outcomes. Front Neurol. 2022;13.
1099. Girot JB, Caroff J, Cortese J, Mihalea C, Rouchaud A, Da Ros V, et al. Endovascular treatment of small and very small intracranial aneurysms with the woven endobridge device. Am J Neuroradiol. 2021;42(7):1276-81.
1100. Adeeb N, Dibas M, Amireh A, Kandregula S, Cuellar H. Comparison of transradial and transfemoral access for the Woven EndoBridge embolization of intracranial aneurysms: A single-center experience. Intervent Neuroradiol. 2021.
1101. Adeeb N, Dibas M, Diestro JDB, Phan K, Cuellar-Saenz HH, Sweid A, et al. Comparing treatment outcomes of various intracranial bifurcation aneurysms locations using the Woven EndoBridge (WEB) device. J Neurointervent Surg. 2022.
1102. Akiyama T, Imamura H, Goto M, Fukumitsu R, Sunohara T, Matsumoto S, et al. Pipeline flow diversion with adjunctive coil embolization for internal carotid artery aneurysms following an intradural component: results in 46 consecutive aneurysms from a Japanese single-center experience. Neurosurg Rev. 2022;45(3):2221-30.
1103. Irie K, Murayama Y, Urashima M, Ikawa F, Sano H, Sato A. Japanese Subarachnoid Aneurysm Trial of Neurosurgical Clipping versus Endovascular Coiling in 1863 Patients with Ruptured Intracranial Aneurysms. Neurol Med -Chir. 2022;62(5):231-7.
1104. Jang D, Cho YD, Yoo DH, Kim SH, Cho WS, Kang HS, et al. Endovascular Treatment of Fenestration-related Aneurysms: Morphological Features, Operative Techniques and Therapeutic Outcomes. Clin Neurorad. 2022;32(1):99-106.
1105. Algin O, Corabay S, Ayberk G. Long-term efficacy and safety of WovenEndoBridge (WEB)-assisted cerebral aneurysm embolization. Intervent Neuroradiol. 2022.
1106. Jankowitz BT, Jadhav AP, Gross B, Jovin TG, Alhajeri AA, Fraser JF, et al. Pivotal trial of the Neuroform Atlas stent for treatment of posterior circulation aneurysms: One-year outcomes. J Neurointervent Surg. 2022;14(2):143-8.
1107. Jee TK, Yeon JY, Kim KH, Kim JS, Hong SC, Jeon P. Treatment Outcomes After Single-Device Flow Diversion for Large or Giant Aneurysms. World Neurosurg. 2021;153:e36-e45.
1108. Jee TK, Yeon JY, Kim KH, Kim JS, Hong SC, Jeon P. Early clinical experience of using the Surpass Evolve flow diverter in the treatment of intracranial aneurysms. Neuroradiology. 2022;64(2):343-51.
1109. Jesser J, Alberalar ND, Kizilkilic O, Saatci I, Baltacioglu F, Özlük E, et al. Safety and Efficacy of the FRED Jr Flow Re-Direction Endoluminal Device for Intracranial Aneurysms: Retrospective Multicenter Experience With Emphasis on Midterm Results. Front Neurol. 2021;12.
1110. Kan P, Mohanty A, Meyers PM, Coon AL, Wakhloo AK, Marosfoi M, et al. Treatment of large and giant posterior communicating artery aneurysms with the Surpass treamline flow diverter: results from the SCENT trial. J Neurointerv Surg. 2022.
1111. Kim HS, Cho YD, Yoo DH, Hong N, Pang CH, Kim KM, et al. Endovascular Treatment of

© 2023 Volovici V et al. *JAMA Network Open.*

Intracranial Kissing Aneurysms: Technical Feasibility and Clinical Outcomes. World Neurosurg. 2021;155:e529-e37.
1112. Kandemirli SG, Baltacioglu F, Jesser J, Kizilkilic O, Islak C, Möhlenbruch M, et al. Flow Redirection Endoluminal Device (FRED) with or without Adjunctive Coiling in Treatment of Very Large and Giant Cerebral Aneurysms. Clin Neurorad. 2021.
1113. Kachhara R, Nair S, Nigam P. Ophthalmic Segment Aneurysms: Surgical Treatment and Outcomes. J Neurosci Rural Pract. 2021;12(4):635-41.
1114. Kidani N, Sugiu K, Terasaka K, Nakashima H, Tokunaga K, Kobayashi K, et al. Mobile endovascular therapy for acute treatment of ruptured vertebral artery dissecting aneurysm in multiple hospitals. Acta Neurochir (Wien). 2022;164(2):517-23.
1115. Jiang W, Zuo Q, Xue G, Zhang X, Tang H, Duan G, et al. Low profile visualized intraluminal support stent-assisted Hydrocoil embolization for acutely ruptured wide-necked intracranial aneurysms: A propensity score-matched cohort study. Clin Neurol Neurosurg. 2022;218.
1116. Jin Y, Guo X, Quan T, Chen Z, Liu C, Guan S. Safety and efficacy of endovascular treatment for tiny ruptured intracranial aneurysms with low-profile visualized intraluminal support stents. Intervent Neuroradiol. 2022.
1117. Kang H, Luo B, Liu J, Zhang H, Li T, Song D, et al. Postoperative occlusion degree after flow-diverter placement with adjunctive coiling: Analysis of complications. J Neurointervent Surg. 2022;14(4):371-5.
1118. Becske T, Brinjikji W, Potts MB, Kallmes DF, Shapiro M, Moran CJ, et al. Long-Term clinical and angiographic outcomes following pipeline embolization device treatment of complex internal carotid artery aneurysms: Five-year results of the pipeline for uncoilable or failed aneurysms trial. Neurosurgery. 2017;80(1):40-8.
1119. Bender MT, Zarrin DA, Campos JK, Lin LM, Huang J, Caplan JM, et al. Tiny Pipes: 67 Cases of Flow Diversion for Aneurysms in Distal Vessels Measuring Less Than 2.0 mm. World Neurosurg. 2019;127:e193-e201.
1120. Briganti F, Napoli M, Leone G, Marseglia M, Mariniello G, Caranci F, et al. Treatment of intracranial aneurysms by flow diverter devices: Long-term results from a single center. Eur J Radiol. 2014;83(9):1683-90.
1121. Ahn JH, Cho YD, Kang HS, Kim JE, Cho WS, Jung SC, et al. Endovascular treatment of ophthalmic artery aneurysms: assessing balloon test occlusion and preservation of vision in coil embolization. AJNR Am J Neuroradiol. 2014;35(11):2146-52.
1122. Al Kasab S, Guerrero WR, Nakagawa D, Samaniego EA, Ortega-Gutierrez S, Hasan D. Safety and Efficacy of the Pipeline Embolization Device Use in the Outside Circle of Willis Located Intracranial Aneurysms: A Single-Center Experience. Intervent Neurol. 2019:83-91.
1123. Alanen M, Pyysalo L, Jalava I, Snicker O, Pienimäki JP, Öhman J, et al. Procedural complications of endovascular treatment in patients with aneurysmal subarachnoid haemorrhage treated at a single centre. Acta Neurochir. 2018;160(3):551-7.
1124. Andaluz N, Zuccarello M. Anterior communicating artery aneurysm surgery through the orbitopterional approach: Long-term follow-up in a series of 75 consecutive patients. Skull Base Surg. 2008;18(4):265-74.
1125. Chen R, Guo R, Wen D, You C, Ma L. Entire Orifice Blocking-Assisted Microsurgical Treatment: Clipping of Intracranial Giant Wide-Neck Paraclinoid Aneurysms. World Neurosurg. 2018;114:e861-e8.
1126. Cavalcanti DD, de Paula RC, Alvarenga PL, Pereira PJDM, Niemeyer Filho P. Engaging in a Keyhole Concept for the Management of Ruptured and Unruptured Aneurysms. World Neurosurg. 2017;102:466-76.
1127. Chalouhi N, Jabbour P, Ibrahim I, Starke RM, Younes P, El Hage G, et al. Surgical treatment of ruptured anterior circulation aneurysms: comparison of pterional and supraorbital keyhole approaches. Neurosurgery. 2013;72(3):437-41; discussion 41-2.
1128. Chalouhi N, Theofanis T, Jabbour P, Dumont AS, Gonzalez LF, Starke RM, et al. Endovascular treatment of posterior communicating artery aneurysms with oculomotor nerve palsy: clinical outcomes and predictors of nerve recovery. AJNR Am J Neuroradiol. 2013;34(4):828-32.
1129. Cheng WY, Lee HT, Sun MH, Shen CC. A pterion keyhole approach for the treatment of anterior circulation aneurysms. Minimally Invasive Neurosurg. 2006;49(5):257-62.
1130. Chhabra R, Gupta SK, Mohindra S, Mukherjee K, Bapuraj R, Khandelwal N, et al. Distal anterior cerebral artery aneurysms: Bifrontal basal anterior interhemispheric approach. Surg Neurol.

2005;64(4):315-9.
1131. Cho YD, Ahn JH, Jung SC, Kim CH, Kang HS, Kim JE, et al. Coil embolization in precommunicating (A1) segment aneurysms of anterior cerebral artery. Neuroradiology. 2014;56(3):219-25.
1132. Cho YD, Kang HS, Kim JE, Son YJ, Lee JY, Lee SJ, et al. Microcatheter looping technique for coil embolization of complex configuration middle cerebral artery aneurysms. Neurosurgery. 2012;71(6):1185-91.
1133. Hanel RA, Cortez GM, Lopes DK, Nelson PK, Siddiqui AH, Jabbour P, et al. Prospective study on embolization of intracranial aneurysms with the pipeline device (PREMIER study): 3-year results with the application of a flow diverter specific occlusion classification. J Neurointerv Surg. 2022.
1134. Kabbasch C, Mpotsaris A, Behme D, Dorn F, Stavrinou P, Liebig T. Pipeline Embolization Device for Treatment of Intracranial Aneurysms-The More, the Better? A Single-center Retrospective Observational Study. J vasc interv radiol neurol. 2016;9(2):14-20.
1135. Jin Y, Guo X, Quan T, Chen Z, Liu C, Guan S. Safety and efficacy of endovascular treatment for tiny ruptured intracranial aneurysms with low-profile visualized intraluminal support stents. Intervent Neuroradiol. 2022.
1136. Jeon BC, Chen SY, Zheng YR, Cho YW, Kwon KY. Superior Orbital Rim Approach for Anterior Communicating Artery Aneurysms: A Surgical Series of 27 Patients. J Korean Med Sci. 2003;18(4):566-72.
1137. Batista LL, Mahadevan J, Sachet M, Alvarez H, Rodesch G. 5-year angiographic and clinical follow-up of coil-embolised intradural saccular aneurysms. Intervent Neuroradiol. 2002;8(4):349-66.
1138. Bae HJ, Park YK, Cho DY, Choi JH, Kim BS, Shin YS. Predictors of the effects of flow diversion in very large and giant aneurysms. Am J Neuroradiol. 2021;42(6):1099-103.
1139. Ba Y, Zhang C, Huang J, Hua X, Cui T, Zhao S, et al. Microsurgical clipping vs. arterial embolization in the treatment of ruptured anterior circulation aneurysms. Am J Transl Res. 2021;13(7):8040-8.
1140. Garg A, Elmashala A, Roeder H, Ortega-Gutierrez S. Endovascular coiling versus neurosurgical clipping for treatment of ruptured and unruptured intracranial aneurysms during pregnancy and postpartum period. J Neurointervent Surg. 2022.
1141. Furtado SV, Jayakumar D, Perikal PJ, Mohan D. Contemporary Management of Distal Anterior Cerebral Artery Aneurysms: A Dual-Trained Neurosurgeon's Perspective. J Neurosci Rural Pract. 2021;12(4):711-7.
1142. Goertz L, Liebig T, Pennig L, Timmer M, Styczen H, Grunz JP, et al. Propensity score-adjusted analysis on stent-assisted coiling versus coiling alone for ruptured intracranial aneurysms. Sci Rep. 2021;11(1):21742.
1143. Gündoğmuş CA, Sabet S, Baltacıoğlu NA, Türeli D, Bayri Y, Baltacıoğlu F. Long-term results and comparison of flow re-direction endoluminal device and pipeline embolization device in endovascular treatment of intracranial carotid aneurysms*. Intervent Neuroradiol. 2022;28(3):302-10.
1144. Alpay K, Hinkka T, Lindgren AE, Isokangas JM, Raj R, Parkkola R, et al. Finnish flow diverter study: 8 years of experience in the treatment of acutely ruptured intracranial aneurysms. J Neurointervent Surg. 2021.
1145. Koltz MT, Chalouhi N, Tjoumakaris S, Fernando Gonzalez L, Dumont A, Hasan D, et al. Short-term outcome for saccular cerebral aneurysms treated with the Orbit Galaxy Detachable Coil System. J Clin Neurosci. 2014;21(1):148-52.
1146. Kang HS, Kwon BJ, Kwon OK, Jung C, Kim JE, Oh CW, et al. Endovascular coil embolization of anterior choroidal artery aneurysms. Clinical article. J Neurosurg. 2009;111(5):963-9.
1147. Lin LM, Colby GP, Kim JE, Huang J, Tamargo RJ, Coon AL. Immediate and follow-up results for 44 consecutive cases of small (<10 mm) internal carotid artery aneurysms treated with the pipeline embolization device. Surg Neurol Int. 2013;4:114.
1148. Li XY, Li CH, Wang JW, Liu JF, Li H, Gao BL. Safety and Efficacy of Endovascular Embolization of Ruptured Intracranial Aneurysms within 72 hours of Subarachnoid Hemorrhage. J Neurol Surg Part A Cent Eur Neurosurg. 2022;83(3):265-74.

© 2023 Volovici V et al. *JAMA Network Open.*

1149. Durst CR, Starke RM, Gaughen JR, Geraghty S, Kreitel KD, Medel R, et al. Single-center experience with a dual microcatheter technique for the endovascular treatment of wide-necked aneurysms. J Neurosurg. 2014;121(5):1093-101.
1150. Kunert P, Wójtowicz K, Żyłkowski J, Jaworski M, Rabczenko D, Wojciechowski J, et al. Flow-diverting devices in the treatment of unruptured ophthalmic segment aneurysms at a mean clinical follow-up of 5 years. Sci Rep. 2021;11(1):9206.
1151. Lebeaupin F, Comby PO, Lenfant M, Thouant P, Lemogne B, Guillen K, et al. Short-and long-term safety and efficacy of self-expandable leo stents used alone or with coiling for ruptured and unruptured intracranial aneurysms: A retrospective observational study. J Clin Med. 2021;10(19).
1152. Lee H, Marotta TR, Spears J, Sarma D, Montanera W, Bharatha A. Endovascular treatment of cavernous carotid artery aneurysms: A 10-year, single-center experience. Neuroradiol j. 2021;34(6):568-74.
1153. Saal-Zapata G, Ghodke B, Walker M, Preguntegui-Loayza I, Rodriguez-Varela R. Endovascular treatment of medium and large intracranial aneurysms with large volume coils: A single-center experience. Surg Neurol Int. 2022;13:9.
1154. Kim CH, Cho YD, Jung SC, Ahn JH, Kang HS, Kim JE, et al. Endovascular treatment for superior cerebellar artery aneurysms: Morphological features, technique, and outcome. Neuroradiology. 2014;56(8):647-54.
1155. Kim S, Park KY, Chung J, Kim YB, Lee JW, Huh SK. Comparative analysis of feasibility of the retrograde suction decompression technique for microsurgical treatment of large and giant internal carotid artery aneurysms. J Korean Neurosurg Soc. 2021;64(5):740-50.
1156. Turhon M, Kang H, Li M, Liu J, Zhang Y, Zhang Y, et al. Treatment of fusiform aneurysms with a pipeline embolization device: a multicenter cohort study. J Neurointerv Surg. 2022.
1157. Vollherbst DF, Cekirge HS, Saatci I, Baltacioglu F, Onal B, Koc O, et al. First clinical multicenter experience with the new Pipeline Vantage flow diverter. J Neurointerv Surg. 2022.
1158. Wang C, Luo B, Li T, Maimaitili A, Mao G, Song D, et al. Comparison of the Pipeline embolisation device alone or combined with coiling for treatment of different sizes of intracranial aneurysms. Stroke Vasc Neurol. 2022.
1159. Vieira E, Guimarães TC, Pontes ECA, Silva ACV, Carneiro MC, Netto AU, et al. Initial experience in the microsurgical treatment of ruptured brain aneurysms in the endovascular era: characteristics and safety of the learning curve in the first 300 consecutively treated patients. Acta Neurochir. 2022;164(4):973-84.
1160. Wan H, Lu G, Huang L, Ge L, Jiang Y, Zhang X. Comparison of Solitaire and Neuroform Stenting for Coiling of Intracranial Bifurcation Aneurysms. Intervent Neuroradiol. 2022.
1161. Uche EO, Matthew M, Meher S, Tripathy L, Mezue W, Jain H, et al. Predictors of Outcome Following Interventions for Ruptured Intracranial Aneurysms in an Emerging Health Institution in West Bengal: A 6-Year Experience. Asian J Neurosurg. 2021;16(2):264-70.
1162. Mori K, Esaki T, Yamamoto T, Nakao Y. Individualized pterional keyhole clipping surgery based on a preoperative three-dimensional virtual osteotomy technique for unruptured middle cerebral artery aneurysm. Minimally Invasive Neurosurg. 2011;54(5-6):207-13.
1163. McDougall CG, Diaz O, Boulos A, Siddiqui AH, Caplan J, Fifi JT, et al. Safety and efficacy results of the Flow Redirection Endoluminal Device (FRED) stent system in the treatment of intracranial aneurysms: US pivotal trial. J Neurointervent Surg. 2022;14(6):577-84.
1164. Martinez-Galdamez M, Lamin SM, Lagios KG, Liebig T, Ciceri EF, Chapot R, et al. Treatment of intracranial aneurysms using the pipeline flex embolization device with shield technology: Angiographic and safety outcomes at 1-year follow-up. J Neurointervent Surg. 2019;11(4):396-9.
1165. Martínez-Galdámez M, Onal Y, Cohen JE, Kalousek V, Rivera R, Sordo JG, et al. First multicenter experience using the Silk Vista flow diverter in 60 consecutive intracranial aneurysms: Technical aspects. J Neurointervent Surg. 2021;13(12):1145-51.

1166. Mascitelli JR, Lawton MT, Hendricks BK, Hardigan TA, Yoon JS, Yaeger KA, et al. Endovascular Therapy Versus Microsurgical Clipping of Ruptured Wide Neck Aneurysms (EVERRUN Registry): a multicenter, prospective propensity score analysis. J Neurosurg. 2021:1-8.
1167. McEachern J, Iancu D, van Adel B, Drake B, Kaderali Z, Spirou M, et al. Long term safety and effectiveness of LVIS Jr for treatment of intracranial aneurysms- a Canadian Multicenter registry. Intervent Neuroradiol. 2022.
1168. Mitchell P, Vindlacheruvu RR, Mahmood K, Ashpole RD, Grivas A, Mendelow AD. Supraorbital eyebrow minicraniotomy for anterior circulation aneurysms. Surg Neurol. 2005;63(1):47-51.
1169. Mizunari T, Murai Y, Kobayashi S, Hoshino S, Teramoto A. Utility of the orbitocranial approach for clipping of anterior communicating artery aneurysms: Significance of dissection of the interhemispheric fissure and the sylvian fissure. J Nippon Med Sch. 2011;78(2):77-83.
1170. Möhlenbruch MA, Kizilkilic O, Killer-Oberpfalzer M, Baltacioglu F, Islak C, Bendszus M, et al. Multicenter experience with FRED Jr flow re-direction endoluminal device for intracranial aneurysms in small arteries. Am J Neuroradiol. 2017;38(10):1959-65.
1171. Mokin M, Chinea A, Primiani CT, Ren Z, Kan P, Srinivasan VM, et al. Treatment of blood blister aneurysms of the internal carotid artery with flow diversion. J Neurointervent Surg. 2018;10(11):1074-8.
1172. Mori K, Wada K, Otani N, Tomiyama A, Toyooka T, Takeuchi S, et al. Keyhole strategy aiming at minimizing hospital stay for surgical clipping of unruptured middle cerebral artery aneurysms. J Neurosurg. 2019;130(4):1359-66.
1173. Mori K, Wada K, Otani N, Tomiyama A, Toyooka T, Tomura S, et al. Long-term neurological and radiological results of consecutive 63 unruptured anterior communicating artery aneurysms clipped via lateral supraorbital keyhole minicraniotomy. Oper Neurosurg. 2018;14(2):95-102.
1174. Mouchtouris N, Hasan D, Samaniego EA, Al Saiegh F, Sweid A, Abbas R, et al. The Woven EndoBridge (WEB) device: feasibility, techniques, and outcomes after FDA approval. J Neurosurg. 2022;136(5):1266-72.
1175. Naamani KE, Chen CJ, Abbas R, Sweid A, Sioutas GS, Badih K, et al. Woven EndoBridge versus stent-assisted coil embolization of cerebral bifurcation aneurysms. J Neurosurg. 2022:1-8.
1176. Strittmatter C, Meyer L, Broocks G, Alexandrou M, Politi M, Boutchakova M, et al. Procedural Outcome Following Stent-Assisted Coiling for Wide-Necked Aneurysms Using Three Different Stent Models: A Single-Center Experience. J Clin Med. 2022;11(12).
1177. Salem MM, Khorasanizadeh M, Lay SV, Renieri L, Kuhn AL, Sweid A, et al. Endoluminal flow diverting stents for middle cerebral artery bifurcation aneurysms: multicenter cohort. J Neurointerv Surg. 2021.
1178. Starke RM, Park MS, Bellon R, Bohnstedt B, Schirmer CM, De Leacy R, et al. Periprocedural safety of saccular aneurysm embolization with the Penumbra SMART Coil System: A SMART registry subset analysis. J Neurointervent Surg. 2021.
1179. Li XY, Li CH, Wang JW, Liu JF, Li H, Gao BL. Endovascular Management of Cerebral Aneurysms of the Posterior Cerebral Artery. Front Neurol. 2021;12.
1180. Li W, Ye M, Cimpoca A, Henkes H, Wang H, Xu X, et al. Avenir R vs. AxiumTM Coils for the Treatment of Intracranial Aneurysms: Results of a Multicenter Randomized Controlled Trial With Short-Term Follow-Up. Front Neurol. 2021;12:817989.
1181. Li L, Gao BL, Shao QJ, Zhang GL, Wang ZL, Li TX, et al. Small Unruptured Intracranial Aneurysms Can Be Effectively Treated With Flow-Diverting Devices. Front Neurol. 2022;13:913653.
1182. Xenofontos A, Raffalli-Ebezant H, Madhavan A, Khan H, Mastan A, Russell I, et al. Simple endovascular coiling: An effective long-term solution for wide-necked ruptured middle cerebral artery aneurysms? A 10-years retrospective study. Neuroradiol J. 2022.
1183. Wu YQ, Li LZ, Wang ZY, Zhang T, Xu M, Cheng MX. Endovascular Intervention with a Low-profile Visualized Intraluminal Support Stent Versus Surgical Clipping for Blood Blister-like Aneurysms:

A Retrospective Study. Clin Neurorad. 2021;31(2):417-24.
1184. Yatomi K, Mitome-Mishima Y, Fujii T, Teranishi K, Oishi H, Kondo A. Outcomes following aneurysmal coil embolization with intentionally shortened low-profile visible intraluminal support stent deployment. Neuroradiol j. 2022;35(1):77-85.
1185. Saatci I, Yavuz K, Ozer C, Geyik S, Cekirge HS. Treatment of intracranial aneurysms using the pipeline flow-diverter embolization device: A single-center experience with long-term follow-up results. Am J Neuroradiol. 2012;33(8):1436-46.
1186. Sturiale CL, Scerrati A, Ricciardi L, Rustemi O, Auricchio AM, Norri N, et al. Clipping versus coiling for treatment of middle cerebral artery aneurysms: a retrospective Italian multicenter experience. Neurosurg Rev. 2022.
1187. Styczen H, Fischer S, Gawlitza M, Meyer L, Goertz L, Maurer C, et al. Reconstructive endovascular treatment of basilar artery fenestration aneurysms: A multi-centre experience and literature review. Neuroradiol J. 2021.
1188. Sukun A, Cetin M, Alparslan A, Uyar R, Gediz T, Cekic B, et al. Mortality Outcomes of Endovascular Treatment and Surgical Clipping in Patients with Cerebral Aneurysms: A Single-Center Study. Turk Neurosurg. 2022;32(2):221-7.
1189. Li S, Lu Z, Tang H, Shang C, Zhao R, Dai D, et al. Flow diversion for aneurysms beyond the circle of Willis: A preliminary experience. J Clin Neurosci. 2022;95:63-9.
1190. Lefevre PH, Schramm P, Kemmling A, Barreau X, Marnat G, Piotin M, et al. Multi-centric European post-market follow-up study of the Neuroform Atlas Stent System: primary results. J Neurointerv Surg. 2022;14(7):694-8.
1191. Lee W, Han HJ, Kim J, Park KY, Kim YB, Jang CK, et al. Flow diverter for the treatment of large (> 10 mm) vertebral artery dissecting aneurysms. Acta Neurochir (Wien). 2022;164(5):1247-54.
1192. Lam J, Ravina K, Rennert RC, Russin JJ. Cerebrovascular bypass for ruptured aneurysms: A case series. J Clin Neurosci. 2021;85:106-14.
1193. Hong Q, Li W, Ma J, Jiang P, Zhang Y. Endovascular treatment of vertebral and basilar artery aneurysms with low-profile visualized intraluminal support device. BMC Neurol. 2021;21(1).
1194. Adeeb N, Dibas M, Diestro JDB, Phan K, Cuellar-Saenz HH, Sweid A, et al. Comparing treatment outcomes of various intracranial bifurcation aneurysms locations using the Woven EndoBridge (WEB) device. J Neurointervent Surg. 2022.
1195. Nakamura Y, Kohmura E. Outcome of surgical clipping for ruptured, low-grade, anterior circulation cerebral aneurysms: Should clipping be omitted after International Subarachnoid Aneurysm Trial? Surg Neurol. 2005;64(6):504-9.
1196. Nanda A, Konar S, Bir SC, Maiti TK, Ambekar S. Modified Far Lateral Approach for Posterior Circulation Aneurysms: An Institutional Experience. World Neurosurg. 2016;94:398-407.
1197. Ozpeynirci Y, Hutschenreuter B, Forbrig R, Bruckmann H, Liebig T, Dorn F. Endovascular treatment of basilar tip aneurysms in the era of endosaccular flow disruption: a comparative study. Neuroradiology. 2021;63(4):619-26.
1198. Pagano P, Paiusan L, Soize S, Pierot L. Intracranial aneurysm treatment with intrasaccular flow disruption: comparison of WEB-21 and WEB-17 systems. J Neurointerv Surg. 2021.
1199. Peng F, Feng X, Tong X, Zhang B, Wang L, Guo E, et al. Endovascular Treatment of Small Ruptured Intracranial Aneurysms (<5mm): Long-term Clinical and Angiographic Outcomes and Related Predictors. Clin Neurorad. 2020;30(4):817-26.
1200. Rahmanian A, Ghaffarpasand F, Derakhshan N. Surgical Outcome of Patients with Very Small Intracranial Aneurysms: A Single-Center Experience from Southern Iran. World Neurosurg. 2017;98:470-8.
1201. Rahmanian A, Ghaffarpasand F, Alibai E, Choque-Velasquez J, Jahromi BR, Hernesniemi J. Surgical Outcome of Very Small Intracranial Aneurysms Utilizing the Double Clip Technique. World Neurosurg. 2018;110:e605-e11.

© 2023 Volovici V et al. *JAMA Network Open.*

1202. Sharma BS, Gupta A, Ahmad FU, Suri A, Mehta VS. Surgical management of giant intracranial aneurysms. Clin Neurol Neurosurg. 2008;110(7):674-81.
1203. Shivhare P, Vaidya P, Garhwal G, Raj N, Gohil J, Sawant N, et al. ICA Bifurcation Aneurysms: Clinical Features and Surgical Outcome in a Tertiary Referral Center in South India. Turk Neurosurg. 2021;31(3):318-23.
1204. Sim SY, Chung J, Choi JH, Kim MJ, Shin YS, Lim YC. Basilar artery trunk aneurysm: Clinical and angiographic outcomes of endovascular treatment. J Neurointervent Surg. 2022;14(3):262-7.
1205. Simgen A, Roth C, Kulikovski J, Papanagiotou P, Roumia S, Dietrich P, et al. Endovascular treatment of unruptured intracranial aneurysms with flow diverters: A retrospective long-term single center analysis. Neuroradiol J. 2022.
1206. Song Y, Kwon B, Al-Abdulwahhab AH, Kurniawan RG, Suh DC. Microcatheter Stabilization Technique Using Partially Inflated Balloon for Coil Embolization of Paraclinoid Aneurysms. Neurointervention. 2021;16(2):132-40.
1207. Oishi H, Yamamoto M, Nonaka S, Shimizu T, Yoshida K, Mitsuhashi T, et al. Treatment results of endosaccular coil embolization of asymptomatic unruptured intracranial aneurysms in elderly patients. J Neurointervent Surg. 2015;7(9):660-5.
1208. Reisch R, Fischer G, Stadie A, Kockro R, Cesnulis E, Hopf N. The supraorbital endoscopic approach for aneurysms. World Neurosurg. 2014;82(6):S130-S7.
1209. Bhogal P, Makalanda HLD, Wong K, Keston P, Downer J, Du Plessis JC, et al. The Silk Vista Baby – The UK experience. Intervent Neuroradiol. 2022;28(2):201-12.
1210. Yıldırım İO, Kolu M, Durak MA, Tetik B, Paşahan R, Gürbüz Ş, et al. Y-stent assisted coiling of ruptured wide neck intracranial aneurysm in the acute phase. Intervent Neuroradiol. 2021;27(5):638-47.
1211. Winkler EA, Lee A, Yue JK, Raygor KP, Rutledge WC, Rubio RR, et al. Endovascular embolization versus surgical clipping in a single surgeon series of basilar artery aneurysms: a complementary approach in the endovascular era. Acta Neurochir. 2021;163(5):1527-40.
1212. Zaitoun MMA, Malky IE, Winklhofer S, Valavanis A, Baltsavias G. Unassisted and multiple microcatheter coiling of distal basilar aneurysms: Outcomes and literature review. Intervent Neuroradiol. 2022;28(2):169-76.
1213. Scerrati A, Trevisi G, Sturiale CL, Salomi F, de Bonis P, Saletti A, et al. Radiological outcomes for endovascular treatment of posterior communicating artery aneurysms: a retrospective multicenter study of the occlusion rate. J Integr Neurosci. 2021;20(4):919-31.
1214. Shi G, Xu S, Gareev I, Ji Z, Pei W, Zhang G, et al. Overlapping stent-assisted coil embolization for vertebrobasilar dissecting aneurysms: a single-center study. Neurol Res. 2021;43(9):701-7.
1215. Qi Y, Sun Y, Wang Y, Jia J, Zhong H, Yang H, et al. The Application of "Stilted Building" Technique in the Embolization of Aneurysms with Secondary Branches. Biomed Res Int. 2021;2021:9976541.
1216. Hong N, Cho WS, Pang CH, Choi YH, Bae JW, Ha EJ, et al. Treatment outcomes of 1-stage clipping of multiple unruptured intracranial aneurysms via keyhole approaches. J Neurosurg. 2022;136(2):475-84.
1217. Hanalioglu S, Sahin B, Sayyahmelli S, Ozaydin B, Erginoglu U, Aycan A, et al. The role of microsurgery for poor-grade aneurysmal subarachnoid hemorrhages in the endovascular era. Acta Neurochir (Wien). 2022;164(3):781-93.
1218. Harada K, Morioka J. Initial experience with an extremely soft bare platinum coil, ED coil-10 Extra Soft, for endovascular treatment of cerebral aneurysms. J Neurointervent Surg. 2013;5(6):577-81.
1219. Onay M, Binboga AB, Altay CM. Analysis of branch artery orifice angulation: Feasibility of the shelf technique for the treatment of wide-neck bifurcation aneurysms. Interv neuroradiol. 2021;27(3):362-71.
1220. Pérez MA, Henkes H, Kurre W, Bleise C, Lylyk PN, Lundquist J, et al. Results of the pToWin Study: Using the pCONUS Device for the Treatment of Wide-Neck Intracranial Aneurysms. J Clin Med. 2022;11(3).
1221. Pierot L, Szikora I, Barreau X, Holtmannspoetter M, Spelle L, Herbreteau D, et al. Aneurysm treatment with WEB in the cumulative population of two prospective, multicenter series: 3-year follow-up.

J Neurointervent Surg. 2021;13(4):363-8.
1222. Profeta G, De Falco R, Ambrosio G, Profeta L. Endoscope-assisted microneurosurgery for anterior circulation aneurysms using the angle-type rigid endoscope over a 3-year period. Child's Nerv Syst. 2004;20(11-12):811-5.
1223. Ho CL, Hwang PY. Endoscope-assisted Transorbital Keyhole Surgical Approach to Ruptured Supratentorial Aneurysms. J Neurol Surg Part A Cent Eur Neurosurg. 2015;76(5):376-83.
1224. Murayama Y, Viñuela F, Ishii A, Nien YL, Yuki I, Duckwiler G, et al. Initial clinical experience with Matrix detachable coils for the treatment of intracranial aneurysms. J Neurosurg. 2006;105(2):192-9.
1225. Pumar JM, Mosqueira A, Olier J, Rodriguez-Fernandez C, Vega P, Gonzalez-Diaz E. Treatment of Intracranial Aneurysms Using the New Silk Vista Flow Diverter: Safety Outcomes at Short-Term Follow-Up. Front Neurol. 2021;12.
1226. Link TW, Carnevale JA, Goldberg JL, Jones C, Kocharian G, Boddu SR, et al. Multiple pipeline embolization devices improves aneurysm occlusion without increasing morbidity: A single center experience of 140 cases. J Clin Neurosci. 2021;86:129-35.
1227. Onay M, Altay CM, Binboga AB. Targeted and Staged Treatment for Ruptured Wide-neck Intracranial Aneurysms: Bleb Coiling Strategy as a New Approach. Acad Radiol. 2022;29:S132-S40.
1228. Winters H, Schüngel MS, Scherlach C, Mucha D, Thalwitzer J, Härtig W, et al. First Experience of Three Neurovascular Centers With the p64MW-HPC, a Low-Profile Flow Diverter Designed for Proximal Cerebral Vessels With Antithrombotic Coating. Front Neurol. 2021;12.
1229. Nouri M, Schneider JR, Shah K, White TG, Katz JM, Dehdashti AR. Cerebral Bypass for Aneurysms in the Era of Flow Diversion: Single-Surgeon Case Series. Oper Neurosurg. 2021;21(5):303-11.
1230. Ni H, Zhao LB, Liu S, Jia ZY, Cao YZ, Shi HB. The Safety and Efficacy of Endovascular Treatment for Very Small Ruptured Anterior Communicating Artery Aneurysms: A Large Single-Center Experience With 81 Consecutive Cases. World Neurosurg. 2021;152:e576-e82.
1231. Ozaki T, Fujinaka T, Kidani T, Nishimoto K, Yamazaki H, Sawada H, et al. Coil Embolization of Unruptured Cerebral Aneurysms Using Stents in Small Arteries Less Than 2 mm in Diameter. Neurosurgery. 2022;90(5):538-46.
1232. Misra BK, Warade AG, Rohan R, Sarit S. Microsurgery of Giant Intracranial Aneurysm: A Single Institution Outcome Study. Neurol India. 2021;69(4):984-90.
1233. You L, Huang J, Zhang J, Jiang Z. Multiple overlapping stent-assisted coiling improves efficacy and safety of treatment for complex intracranial aneurysms: a randomized trial. Biomed Eng Online. 2021;20(1).
1234. Yoon PH, Lee JW, Lee YH, Kwon YS, Yang KH. Dual microcatheter coil embolization of acutely ruptured wide-necked intracranial aneurysms. Intervent Neuroradiol. 2017;23(5):477-84.
1235. Zhang H, Li L, Zhang H, Liu J, Song D, Zhao Y, et al. Small and Medium-Sized Aneurysm Outcomes Following Intracranial Aneurysm Treatment Using the Pipeline Embolization Device: A Subgroup Analysis of the PLUS Registry. Front Neurol. 2022;13:881353.
1236. Yeon EK, Cho YD, Yoo DH, Kim JE, Kim KM, Lee SH, et al. Midterm Outcomes after Low-Profile Visualization Endoluminal Support or Atlas Stent-Assisted Coiling of Intracranial Aneurysms: A Propensity Score Matching Analysis. Neurosurgery. 2021;89(5):862-6.
1237. Zheng SF, Yao PS, Yu LH, Kang DZ. Keyhole approach combined with external ventricular drainage for ruptured, Poor-Grade, anterior circulation cerebral Aneurysms. Medicine. 2015;94(51).
1238. Xue G, Zuo Q, Zhang X, Tang H, Zhao R, Li Q, et al. Safety and efficacy of stent-assisted coiling for acutely ruptured wide-necked intracranial aneurysms: comparison of LVIS stents with laser-cut stents. Chin Neurosurg J. 2021;7(1).
1239. Zhao C, Ma Z, Zhang Y, Mou S, Yang Y, Yang Y, et al. Application of Micromirror in Microsurgical Clipping to the Intracranial Aneurysms. J Craniofac Surg. 2018;29(3):e287-e90.
1240. Kis B, Weber W, Götz F, Becker H, Berlit P, Kühne D. Endovascular treatment of cerebral aneurysms using the Leo stent: Long-term follow-up and expansion of indications. Clin Neurorad. 2007;17(3):167-79.

© 2023 Volovici V et al. *JAMA Network Open.*

1241. Kim J, Han HJ, Lee W, Park SK, Chung J, Kim YB, et al. Safety and efficacy of stent-assisted coiling of unruptured intracranial aneurysms using low-profile stents in small parent arteries. Am J Neuroradiol. 2021;42(9):1621-6.
1242. Yamahata H, Tokimura H, Tajitsu K, Tsuchiya M, Taniguchi A, Hirabaru M, et al. Efficacy and safety of the pterional keyhole approach for the treatment of anterior circulation aneurysms. Neurosurg Rev. 2014;37(4):629-36.
1243. Kiran NA, Jahromi BR, Velasquez JC, Hijazy F, Goehre F, Kivisaari R, et al. Double-clip technique for the microneurosurgical management of very small (< 3 mm) intracranial aneurysms. Neurosurgery. 2015;11 Suppl 2:3-7.
1244. Yang I, Lawton MT. Clipping of complex aneurysms with fenestration tubes: application and assessment of three types of clip techniques. Neurosurgery. 2008;62(5 Suppl 2):ONS371-8; discussion 8-9.
1245. Sweid A, Robert MS, Herial N, Chalouhi N, Das S, Baldassari MP, et al. Predictors of complications, functional outcome, and morbidity in a large cohort treated with flow diversion. Neurosurgery. 2020;87(4):730-43.
1246. Sweid A, Herial N, Sajja K, Chalouhi N, Velagapudi L, Doermann A, et al. Early multicenter experience with the neuroform atlas stent: Feasibility, safety, and efficacy. Neurosurgery. 2020;87(3):E321-E35.
1247. Murphy KJ, Houdart E, Szopinski KT, Levrier O, Guimaraens L, Kühne D, et al. Mechanical detachable platinum coil: Report of the European phase II clinical trial in 60 patients. Radiology. 2001;219(2):541-4.
1248. Nussbaum ES, Touchette JC, Madison MT, Goddard JK, Lassig JP, Meyers ME, et al. Procedural complications in patients undergoing microsurgical treatment of unruptured intracranial aneurysms: a single-center experience with 1923 aneurysms. Acta Neurochir. 2022;164(2):525-35.
1249. Won YS, Rho MH, Chung EC, Hong HP, Kim SY, Park HJ, et al. Multiple overlapping stent-assisted coiling of complex aneurysms: a single-center experience. Neurol Res. 2015;37(3):189-96.
1250. Won SY, Seifert V, Dubinski D, Kashefiolasl S, Dinc N, Bruder M, et al. Short- and midterm outcome of ruptured and unruptured intracerebral wide-necked aneurysms with microsurgical treatment. Sci Rep. 2021;11(1):4982.
1251. Wong AD, Alubankudi R, Jarrett J, Huynh TJ, Dmytriw AA, Pickett GE. Management of Ruptured Intracranial Aneurysms in the Post-International Subarachnoid Aneurysm Trial Era: A Single-Centre Prospective Series. Can J Neurol Sci. 2022;49(1):62-9.
1252. Peng Q, Zhou Y, Li W, Wang C, Dong L, Mu S, et al. Reconstructive Endovascular Treatment of Basilar Trunk and Vertebrobasilar Junction Aneurysms: A Review of 77 Consecutive Cases. Front Neurol. 2022;13.
1253. Pflaeging M, Kabbasch C, Schlamann M, Pennig L, Juenger ST, Grunz JP, et al. Microsurgical Clipping versus Advanced Endovascular Treatment of Unruptured Middle Cerebral Artery Bifurcation Aneurysms After a "Coil-First" Policy. World Neurosurg. 2021;149:e336-e44.
1254. Yao L, Wu Q, Yuan B, Wen L, Yi R, Zhou X, et al. Correlation Between Vascular Geometry Changes and Long-Term Outcomes After Enterprise Stent Deployment for Intracranial Aneurysms Located on Small Arteries. World Neurosurg. 2021;153:e96-e104.

1255. Yeon EK, Cho YD, Yoo DH, Kim JE, Kim KM, Lee SH, et al. Midterm Outcomes after Low-Profile Visualization Endoluminal Support or Atlas Stent-Assisted Coiling of Intracranial Aneurysms: A Propensity Score Matching Analysis. Neurosurgery. 2021;89(5):862-6.
1256. Thanabalasundaram G, Soon WC, Ponnampalam A, Brydon HL. Case series of 100 supraorbital mini-craniotomies in patients with good grade aneurysmal subarachnoid haemorrhage at a single neurosurgical Centre. Br J Neurosurg. 2021.
1257. Xue G, Liu P, Xu F, Fang Y, Li Q, Hong B, et al. Endovascular Treatment of Ruptured Wide-Necked Anterior Communicating Artery Aneurysms Using a Low-Profile Visualized Intraluminal Support (LVIS)

© 2023 Volovici V et al. *JAMA Network Open.*

Device. Front Neurol. 2020;11.
1258. Xue G, Zhou Y, Liu P, Zuo Q, Yang P, Fang Y, et al. Endovascular Treatment of Ruptured Middle Cerebral Artery Aneurysms With a Low-Profile Visualized Intraluminal Support Device. Front Neurol. 2020;11.
1259. Wu Q, Xu S, Wang C, Ji Z, Li Y, Sun B, et al. Endovascular Management of Vertebrobasilar Trunk Artery Large Aneurysms: Complications and Long-Term Results. Front Neurol. 2022;13.
1260. Liu J, Li W, Zhang Y, Wang K, Yang X, Zhang Y. Hemodynamic analysis for endovascular treatment in small unruptured intracranial aneurysms: a matched comparison study of flow diverter versus LVIS. Chin Neurosurg J. 2021;7(1).
1261. Luo J, Wang C, Dai Y, Chen X, Tian X, Lin Y, et al. Efficacy and safety of endovascular therapy versus surgical clipping for patients with unruptured middle cerebral artery bifurcation aneurysms. J Investig Med. 2022.
1262. Ni H, Zhao LB, Liu S, Jia ZY, Cao YZ, Shi HB. Open-cell stent-assisted coiling for the treatment of paraclinoid aneurysms: traditional endovascular treatment is still not out of date. Neuroradiology. 2021;63(9):1521-30.
1263. Oishi H, Teranishi K, Yatomi K, Fujii T, Yamamoto M, Arai H. Flow diverter therapy using a pipeline embolization device for 100 unruptured large and giant internal carotid artery aneurysms in a single center in a Japanese population. Neurol Med -Chir. 2018;58(11):461-7.
1264. Nickele C, Oravec CS, Morris SD, Hoit D, Elijovich L, Arthur AS. Long-Term Follow-up of Aneurysms Treated With Hydrogel-Coated Coils Shows Progressive Thrombosis and Improvement in Raymond-Roy Classification. Oper Neurosurg (Hagerstown). 2022;22(4):239-43.
1265. Luzzi S, Giotta Lucifero A, Baldoncini M, Del Maestro M, Elbabaa SK, Galzio R. Paraclinoid aneurysms: Outcome analysis and technical remarks of a microsurgical series. Interdiscip Neurosurg Adv Tech Case Manage. 2022;27.
1266. Ma C, Zhu H, Liang S, Liang F, Sun J, Zhang Y, et al. Comparison of Pipeline Embolization Device and Traditional Endovascular Therapeutic Approaches in Distal Cerebral Circulation Aneurysms Using Propensity Score Matching Analysis. Front Neurol. 2022;13.
1267. Piano M, Lozupone E, Sgoifo A, Nuzzi NP, Asteggiano F, Pero G, et al. Long-term follow-up of the DERIVO® Embolization Device (DED®) for intracranial aneurysms: The Italian Multicentric Registry. J Neurosurg Sci. 2021;65(3):361-8.
1268. Porto GBF, Al Kasab S, Sattur MG, Almallouhi E, Lajthia O, Casey MA, et al. Endovascular Management of Distal Anterior Cerebral Artery Aneurysms: A Multicenter Retrospective Review. World Neurosurg. 2021;154:e421-e7.
1269. Zhang Q, Shao Q, Chang K, Zhang H, He Y, Andrade-Barazarte H, et al. Safety and Efficacy of Coils in Conjunction With the Pipeline Flex Embolization Device for the Treatment of Cerebral Aneurysms. Front Neurol. 2021;12.
1270. Zhang J, Yu M, Lv X. Endovascular treatment of blood blister-like aneurysms of internal carotid artery: Stent-assisted coiling and pipeline flow diversion. J Clin Neurosci. 2021;90:8-13.
1271. Zhao R, Duan G, Yang P, Li T, Guan S, Yang H, et al. Endovascular Aneurysm Treatment with the Numen Coil Embolization System: A Prospective Randomized Controlled Open-Label Multicenter Noninferiority Trial in China. World Neurosurg. 2022;160:e23-e32.
1272. Zarco F, Macias N, Delgado F, Rosati S, Gonzalez A, Jimenez E, et al. Multicenter Retrospective Registry of Anterior Communicating Artery Aneurysms with Endovascular Therapy (MACAARET): safety and efficacy study according to morphological considerations and spatial orientations. Clin Radiol. 2021;76(10):786.e1-.e8.
1273. You W, Feng J, Ge H, Jin H, Liu P, Li Y, et al. Bifurcated Aneurysm Location Predicts In-Stent Stenosis After Neuroform-EZ Stent-Assisted Coiling for Intracranial Aneurysm. Front Neurol. 2022;13.

© 2023 Volovici V et al. *JAMA Network Open.*

1274. Szmygin P, Szmygin M, Roman T, Jargiełło T. Endovascular treatment of vertebrobasilar system aneurysms — long term results. Neurol Neurochir Pol. 2021;55(6):567-73.

1275. Take Y, Kamide T, Kikkawa Y, Ikegami M, Teranishi A, Ehara T, et al. Current treatment options and prognostic factors for ruptured distal anterior cerebral artery aneurysms. Surg Neurol Intl. 2021;12.
1276. Tang H, Shang C, Zhang G, Zuo Q, Zhang X, Xu F, et al. Braided stents assisted coiling for endovascular management of posterior cerebral artery aneurysms: a preliminary mid-term experience. 2022.
1277. Taqi MA, Raz E, Vechera A, Shapiro M, Gupta R, Haynes J, et al. Early Experience with Comaneci, a Newly FDA-Approved Controllable Assist Device for Wide-Necked Intracranial Aneurysm Coiling. Cerebrovasc Dis. 2021;50(4):464-71.

1278. Suzuki R, Takigawa T, Nariai Y, Hyodo A, Suzuki K. Comparison of Pipeline Embolization and Coil Embolization for the Treatment of Large Unruptured Paraclinoid Aneurysms. Neurol Med Chir (Tokyo). 2022;62(2):97-104.

1279. Suyama K, Nakahara I, Matsumoto S, Suyama Y, Morioka J, Hasebe A, et al. Efficacy of the Flow Re-direction Endoluminal Device for cerebral aneurysms and causes of failed deployment. Neuroradiology. 2022;64(6):1213-9.

1280. Hellstern V, Aguilar-Pérez M, Henkes E, Serna-Candel C, Wendl C, Bäzner H, et al. Endovascular Treatment of Posterior Circulation Saccular Aneurysms With the p64 Flow Modulation Device: Mid-and Long-Term Results in 54 Aneurysms From a Single Center. Front Neurol. 2021;12.

1281. Sturiale CL, La Rocca G, Puca A, Fernandez E, Visocchi M, Marchese E, et al. Minipterional craniotomy for treatment of unruptured middle cerebral artery aneurysms. A single-center comparative analysis with standard pterional approach as regard to safety and efficacy of aneurysm clipping and the advantages of reconstruction. Acta neurochirurgica, supplementum. 2017;124:93-100.

1282. Spiotta AM, Park MS, Bellon RJ, Bohnstedt BN, Yoo AJ, Schirmer CM, et al. The SMART Registry: Long-Term Results on the Utility of the Penumbra SMART COIL System for Treatment of Intracranial Aneurysms and Other Malformations. Front Neurol. 2021;12.

1283. Seo D, Jo H, Jeong HG, Kim YD, Lee SU, Ban SP, et al. Simultaneous Craniotomies for Multiple Intracranial Aneurysm Clippings—One-Stage Surgery with Multiple Craniotomies. World Neurosurg. 2022;158:e689-e96.

1284. Sato H, Haraguchi K. Comparison of Stent-Assisted Coiling for Unruptured Internal Carotid Artery Aneurysms Between LVIS or LVIS Jr. and Enterprise VRD: A Retrospective and Single-Center Analysis. Turk Neurosurg. 2021;31(3):379-84.

1285. Sano H. Treatment of complex intracranial aneurysms of anterior circulation using multiple clips. Acta Neurochir Suppl2010. p. 27-31.

1286. Salem MM, Sweid A, Kuhn AL, Dmytriw AA, Gomez-Paz S, Maragkos GA, et al. Repeat Flow Diversion for Cerebral Aneurysms Failing Prior Flow Diversion: Safety and Feasibility From Multicenter Experience. Stroke. 2022;53(4):1178-89.

1287. Rautio R, Alpay K, Sinisalo M, Numminen J. Treatment of intracranial aneurysms using the new Surpass Evolve flow diverter: Safety outcomes and six-month imaging follow-up. J Neuroradiol. 2021.

1288. Qureshi AI, Suri MFK, Khan J, Kim SH, Fessler RD, Ringer AJ, et al. Endovascular treatment of intracranial aneurysms by using Guglielmi detachable coils in awake patients: Safety and feasibility. J Neurosurg. 2001;94(6):880-5.

1289. Qin F, Liu J, Zhao X, Wu D, Lai N, Zhang Z, et al. Endovascular Treatment of Ruptured Very Small Intracranial Aneurysms: Complications, Recurrence Rate, and Clinical Outcomes. Front Neurol. 2021;12.

1290. Pumar JM, Sucasas P, Mosqueira A, Vega P, Murias E. Five-Years Angiographic Follow-Up of Wide-Neck Intracranial Aneurysms Treated With LEO Plus Stent. Front Neurol. 2021;12.

1291. Ohshima T, Dash C, Belayev A, Yamamoto T, Goto S, Kato Y. 8-F balloon guide catheter for embolization of anterior circulation aneurysms: an institutional experience in 152 patients. Nagoya J Med Sci. 2017;79(4):435-41.

1292. Heye S, Stracke CP, Nordmeyer H, Heddier M, Stauder M, Chapot R. Retrograde access to the posterior inferior cerebellar artery in balloon-assisted coiling of posterior inferior cerebellar artery aneurysms. J Neurointervent Surg. 2015;7(11):824-8.

1293. Harris L, Hill CS, Elliot M, Fitzpatrick T, Ghosh A, Vindlacheruvu R. Comparison between outcomes of endovascular and surgical treatments of ruptured anterior communicating artery aneurysms. Br J Neurosurg. 2021;35(3):313-8.

1294. Hanel RA, Yoon N, Sauvageau E, Aghaebrahim A, Lin E, Jadhav AP, et al. Neuroform Atlas Stent for Treatment of Middle Cerebral Artery Aneurysms: 1-Year Outcomes from Neuroform Atlas Stent Pivotal Trial. Neurosurgery. 2021;89(1):102-8.

1295. Goertz L, Liebig T, Siebert E, Pennig L, Laukamp KR, Celik E, et al. Woven Endobridge Embolization Versus Microsurgical Clipping for Unruptured Anterior Circulation Aneurysms: A Propensity Score Analysis. Neurosurgery. 2021;88(4):779-84.

1296. Burrows AM, Cloft H, Kallmes DF, Lanzino G. Periprocedural and mid-term technical and clinical events after flow diversion for intracranial aneurysms. J Neurointervent Surg. 2015;7(9):646-51.

1297. Teixeira VF, Bastos AM, Santos RB. Endovascular Therapy of 103 Aneurysms in the Internal Carotid Artery with Flow Re-Direction Endoluminal Device. Arq Bras Neurocir. 2021.

1298. Tessitore A, Paolucci A, Hohenstatt S, Caragliano AA, Buonomo O, Mormina E, et al. Comparison between second generation HydroSoft coils and bare platinum coils for the treatment of large intracranial aneurysms. Intervent Neuroradiol. 2022.

1299. Wang J, Kan Z, Wang S. Surgical management of paraclinoid aneurysms via frontal lateral approach. Biomed Res. 2018;2018(Special Issue ArtificialIntelligentTechniquesforBioMedicalSignalProcessingEdition-II):S281-S7.

1300. Wang X, Luo J, Li P, Cheng B, Li Z, Cheng H. The role of pterional keyhole approach in the microsurgical clipping of anterior circulation artery aneurysms: Experiences with 26 cases and literature review. Int J Clin Exp Med. 2016;9(3):5922-31.

1301. Patel AS, Griessenauer CJ, Ogilvy CS, Thomas AJ. Biaxial system using the Benchmark intracranial guide catheter for placement of a Pipeline Embolization Device for intracranial aneurysms. Intervent Neuroradiol. 2016;22(4):402-6.

1302. Yin Q, Guo H, Wei J, Liu P, Lv M, Li Y. Posterior inferior cerebellar artery aneurysms: Comparison of results of surgical and endovascular managements at one single center. Neurol India. 2020;68(5):1115-24.

1303. Zhitao J, Yibao W, Anhua W, Shaowu O, Yunchao B, Renyi Z, et al. Microsurgical subtemporal approach to aneurysms on the P2 segment of the posterior cerebral artery. Neurol India. 2010;58(2):242-7.

1304. Bhatoe HS. Transciliary supraorbital keyhole approach in the management of aneurysms of anterior circulation: Operative nuances. Neurol India. 2009;57(5):599-606.

1305. Tang Y, Chen H, Ma F, Scharnweber R, Zhang J, Chen G, et al. Experience Using the Pterional Keyhole Approach for the Treatment of Ruptured Intracranial Aneurysms of the Anterior Circulation. World Neurosurg. 2018;118:e800-e5.

© 2023 Volovici V et al. *JAMA Network Open.*

1306. Wang J, Wu J, Cao Y, Kan Z, Wang S. Comparison between frontolateral approach and pterional approach in the surgical treatment of paraclinoid aneurysms. J Clin Neurosci. 2018;52:80-7.
1307. Vignesh S, Prasad SN, Singh V, Phadke RV, Balaguruswamy MM, Udiya A, et al. Balloon-Assisted Coiling of Intracranial Aneurysms: Technical Details and Evaluation of Local Complications. Neurol India. 2022;70(2):643-51.
1308. Molyneux AJ, Birks J, Clarke A, Sneade M, Kerr RSC. The durability of endovascular coiling versus neurosurgical clipping of ruptured cerebral aneurysms: 18 year follow-up of the UK cohort of the International Subarachnoid Aneurysm Trial (ISAT). Lancet. 2015;385(9969):691-7.
1309. Molyneux A, Kerr R, Stratton I, Sandercock P, Clarke M, Shrimpton J, et al. International Subarachnoid Aneurysm Trial (ISAT) of neurosurgical clipping versus endovascular coiling in 2143 patients with ruptured intracranial aneurysms: A randomised trial. Lancet. 2002;360(9342):1267-74.
1310. White PM, Lewis SC, Gholkar A, Sellar RJ, Nahser H, Cognard C, et al. Hydrogel-coated coils versus bare platinum coils for the endovascular treatment of intracranial aneurysms (HELPS): A randomised controlled trial. Lancet. 2011;377(9778):1655-62.
1311. White PM, Lewis SC, Nahser H, Sellar RJ, Goddard T, Gholkar A. HydroCoil Endovascular Aneurysm Occlusion and Packing Study (HELPS trial): Procedural safety and operator-assessed efficacy results. Am J Neuroradiol. 2008;29(2):217-23.
1312. Qin X, Xu F, Maimaiti Y, Zheng Y, Xu B, Leng B, et al. Endovascular treatment of posterior cerebral artery aneurysms: A single center's experience of 55 cases. J Neurosurg. 2017;126(4):1094-105.
1313. Pumar JM, Mosqueira A, Cuellar H, Dieguez B, Guimaraens L, Masso J, et al. Expanding the use of flow diverters beyond their initial indication: Treatment of small unruptured aneurysms. J Neurointervent Surg. 2018;10(3):245-8.
1314. Pumar JM, Banguero A, Cuellar H, Guimaraens L, Masso J, Miralbes S, et al. Treatment of intracranial aneurysms with the SILK embolization device in a multicenter study. A retrospective data analysis. Neurosurgery. 2017;81(4):595-601.
1315. Puffer RC, Piano M, Lanzino G, Valvassori L, Kallmes DF, Quilici L, et al. Treatment of cavernous sinus aneurysms with flow diversion: Results in 44 patients. Am J Neuroradiol. 2014;39(5):948-51.
1316. Poncyljusz W, Biliński P, Safranow K, Baron J, Zbroszczyk M, Jaworski M, et al. The LVIS/LVIS Jr. stents in the treatment of wideneck intracranial aneurysms: Multicentre registry. J Neurointervent Surg. 2015;7(7):524-9.
1317. Piske RL, Kanashiro LH, Paschoal E, Agner C, Lima SS, Aguiar PH. Evaluation of onyx hd-500 embolic system in the treatment of 84 wide-neck intracranial aneurysms. Neurosurgery. 2009;64(5):E865-E75.
1318. Qu S, Lv X, Wu Z. Clinical outcomes of basilar artery aneurysms. Neuroradiol J. 2009;22(2):228-38.
1319. Quadros RS, Gallas S, Noudel R, Rousseaux P, Pierot L. Endovascular treatment of middle cerebral artery aneurysms as first option: A single center experience of 92 aneurysms. Am J Neuroradiol. 2007;28(8):1567-72.

1320. Quintana EM, Valdes PV, Deza EM, García AG, Rodríguez MC, Pérez JMJ, et al. Initial experience and one-year follow-up with Neuroform Atlas Stent System for the treatment of brain aneurysms. Intervent Neuroradiol. 2019.
1321. Raftopoulos C, Mathurin P, Boscherini D, Billa RF, Van Boven M, Hantson P. Prospective analysis of aneurysm treatment in a series of 103 consecutive patients when endovascular embolization is considered the first option. J Neurosurg. 2000;93(2):175-82.
1322. Piotin M, Blanc R, Spelle L, Mounayer C, Piantino R, Schmidt PJ, et al. Stent-assisted coiling of intracranial aneurysms: Clinical and angiographic results in 216 consecutive aneurysms. Stroke. 2010;41(1):110-5.
1323. Raftopoulos C, Goffette P, Vaz G, Ramzi N, Scholtes JL, Wittebole X, et al. Surgical clipping may lead to better results than coil embolization: Results from a series of 101 consecutive unruptured

© 2023 Volovici V et al. *JAMA Network Open.*

intracranial aneurysms. Neurosurgery. 2003;52(6):1280-90.
1324. Piotin M, Biondi A, Sourour N, Mounayer C, Jaworski M, Mangiafico S, et al. The LUNA aneurysm embolization system for intracranial aneurysm treatment: Short-term, mid-term and long-term clinical and angiographic results. J Neurointervent Surg. 2018;10(12):E34.
1325. Pierot XL, Moret J, Turjman F, Herbreteau D, Raoult H, Barreau X, et al. WEB treatment of intracranial aneurysms: Clinical and anatomic results in the French Observatory. Am J Neuroradiol. 2016;37(4):655-9.
1326. Pierot L, Spelle L, Molyneux A, Byrne J. Clinical and anatomical follow-up in patients with aneurysms treated with the WEB device: 1-year follow-up report in the cumulated population of 2 prospective, multicenter series (WEBCAST and French Observatory). Neurosurgery. 2015;78(1):133-9.
1327. Iskandar A, Nepper-Rasmussen J. Endovascular treatment of very small intracranial aneurysms. Intervent Neuroradiol. 2011;17(3):299-305.
1328. Chiu AH, De Vries J, O'Kelly CJ, Riina H, McDougall I, Tippett J, et al. The second-generation eCLIPs Endovascular Clip System: Initial experience. J Neurosurg. 2018;128(2):482-9.
1329. Raymond J, Klink R, Chagnon M, Barnwell SL, Evans AJ, Mocco J, et al. Patients prone to recurrence after endovascular treatment: Periprocedural results of the PRET randomized trial on large and recurrent aneurysms. Am J Neuroradiol. 2014;35(9):1667-76.
1330. Raz E, Shapiro M, Becske T, Zumofen DW, Tanweer O, Potts MB, et al. Anterior choroidal artery patency and clinical follow-up after coverage with the pipeline embolization device. AJNR Am J Neuroradiol. 2015;36(5):937-42.
1331. Rho MH, Park HJ, Chung EC, Choi YJ, Lee SY, Won YS, et al. Various techniques of stent-assisted coil embolization of wide-necked or fusiform artherosclerotic and dissecting unruptured vertebrobasilar artery aneurysms for reducing recanalization: Mid-term results. Acta Neurochir. 2013;155(11):2009-17.
1332. Rivet DJ, Moran CJ, Mazumdar A, Pilgram TK, Derdeyn CP, Cross DT. Single-institution experience with matrix coils in the treatment of intracranial aneurysms: Comparison with same-center outcomes with the use of platinum coils. Am J Neuroradiol. 2007;28(9):1736-42.
1333. Rossitti S. Endovascular coiling of intracranial aneurysms using bioactive coils: a single-center study. Acta Radiol. 2007;48(5):565-76.
1334. Roy D, Milot G, Raymond J. Endovascular treatment of unruptured aneurysms. Stroke. 2001;32(9):1998-2004.
1335. Saatci I, Yavuz K, Ozer C, Geyik S, Cekirge HS. Treatment of intracranial aneurysms using the pipeline flow-diverter embolization device: A single-center experience with long-term follow-up results. Am J Neuroradiol. 2012;33(8):1436-46.
1336. Safavi-Abbasi S, Moron F, Sun H, Oppenlander ME, Kalani MYS, Mulholland CB, et al. Techniques and long-term outcomes of cotton-clipping and cotton-augmentation strategies for management of cerebral aneurysms. J Neurosurg. 2016;125(3):720-9.
1337. Safavi-Abbasi S, Moron F, Sun H, Wilson C, Frock B, Oppenlander ME, et al. Techniques and Outcomes of Gore-Tex Clip-Wrapping of Ruptured and Unruptured Cerebral Aneurysms. World Neurosurg. 2016;90:281-90.
1338. Schaller B. Extracranial-Intracranial Bypass to Reduce the Risk of Ischemic Stroke in Intracranial Aneurysms of the Anterior Cerebral Circulation: A Systematic Review. J Stroke Cerebrovasc Dis. 2008;17(5):287-98.
1339. Seifert V, Gerlach R, Raabe A, Guresir E, Beck J, Szelenyi A, et al. The interdisciplinary treatment of unruptured intracranial aneurysms. Dtsch Arztebl int. 2008;105(25):449-56.
1340. Sekhar LN, Tariq F, Morton RP, Ghodke B, Hallam DK, Barber J, et al. Basilar tip aneurysms: A microsurgical and endovascular contemporary series of 100 patients. Neurosurgery. 2013;72(2):284-98.
1341. Sejkorová A, Petr O, Mulino M, Cihlář J, Hejčl A, Thomé C, et al. Management of posterior inferior cerebellar artery aneurysms: What factors play the most important role in outcome? Acta Neurochir.

2017;159(3):549-58.
1342. Zweifel C, Sacho RH, Tymianski R, Radovanovic I, Tymianski M. Safety, efficacy, and cost of surgery for patients with unruptured aneurysms deemed unsuitable for endovascular therapy. Acta Neurochir. 2015;157(12):2061-70.
1343. Yahia AM, Gordon V, Whapham J, Malek A, Rehman M, Fessler RD. Sapphire® platinum detachable coil experience in a tertiary-care facility. Neurocrit Care. 2007;7(2):128-35.
1344. Yadla S, Campbell PG, Grobelny B, Jallo J, Gonzalez LF, Rosenwasser RH, et al. Open and endovascular treatment of unruptured carotid-ophthalmic aneurysms: clinical and radiographic outcomes. Neurosurgery. 2011;68(5):1434-43; discussion 43.
1345. Van Rooij WJ. Endovascular treatment of cavernous sinus aneurysms. Am J Neuroradiol. 2012;33(2):323-6.
1346. Taqi MA, Quadri SA, Puri AS, Fitzsimmons BF, Jin JN, Rai AT, et al. A Prospective Multicenter Trial of the TransForm Occlusion Balloon Catheter: Trial Design and Results. Intervent Neurol. 2018;7(1-2):53-64.
1347. Taschner CA, Chapot R, Costalat V, Machi P, Courthéoux P, Barreau X, et al. GREAT—a randomized controlled trial comparing HydroSoft/HydroFrame and bare platinum coils for endovascular aneurysm treatment: procedural safety and core-lab-assessedangiographic results. Neuroradiology. 2016;58(8):777-86.
1348. Tjahjadi M, Kim T, Ojar D, Byoun HS, Lee SU, Ban SP, et al. Long-term review of selected basilar-tip aneurysm endovascular techniques in a single institution. Interdiscip Neurosurg Adv Tech Case Manage. 2017;8:50-6.
1349. Song J, Kim BS, Shin YS. Treatment outcomes of unruptured intracranial aneurysm; experience of 1231 consecutive aneurysms. Acta Neurochir. 2015;157(8):1303-11.
1350. Szelényi A, Beck J, Strametz R, Blasel S, Oszvald A, Raabe A, et al. Is the surgical repair of unruptured atherosclerotic aneurysms at a higher risk of intraoperative ischemia? Clin Neurol Neurosurg. 2011;113(2):129-35.
1351. Taha MM, Nakahara I, Higashi T, Iwamuro Y, Iwaasa M, Watanabe Y, et al. Endovascular embolization vs surgical clipping in treatment of cerebral aneurysms: morbidity and mortality with short-term outcome. Surg Neurol. 2006;66(3):277-84.
1352. Chalouhi N, Jabbour P, Gonzalez LF, Dumont AS, Rosenwasser R, Starke RM, et al. Safety and efficacy of endovascular treatment of basilar tip aneurysms by coiling with and without stent assistance: A review of 235 cases. Neurosurgery. 2012;71(4):785-94.
1353. Kulcsár Z, Wanke I, Rüfenacht D, Wetzel SG, Göricke S, Kolia K, et al. Safety and effectiveness of large volume coils in the treatment of small aneurysms. J Neurointervent Surg. 2016;8(12):1260-3.
1354. Sluzewski M, Menovsky T, Van Rooij WJ, Wijnalda D. Coiling of very large or giant cerebral aneurysms: Long-term clinical and serial angiographic results. Am J Neuroradiol. 2003;24(2):257-62.
1355. Iskandar A, Nepper-Rasmussen J. Endovascular treatment of very small intracranial aneurysms. Intervent Neuroradiol. 2011;17(3):299-305.
1356. Van Rooij WJJ, Sluzewski M, Metz NH, Nijssen PCG, Wijnalda D, Rinkel GJE, et al. Carotid balloon occlusion for large and giant aneurysms: Evaluation of a new test occlusion protocol. Neurosurgery. 2000;47(1):116-22.

© 2023 Volovici V et al. *JAMA Network Open.*
