# Supplementary material for: Outcomes Associated With Intracranial Aneurysm Treatments Reported as Safe, Effective, or Durable: A Systematic Review and Meta-Analysis
Source: JAMA Netw Open. 2023 Sep 1;6(9):e2331798. doi: 10.1001/jamanetworkopen.2023.31798 (PMC10474558; doi:10.1001/jamanetworkopen.2023.31798)
Supplement: Supplement 3. — Data Sharing Statement [file jamanetwopen-e2331798-s003.pdf]

Data Sharing Statement

Volovici. Outcomes Associated With Intracranial Aneurysm Treatments Reported as Safe, Effective, or Durable. *JAMA Netw Open*. Published September 01, 2023. doi:10.1001/jamanetworkopen.2023.31798

Data
Data available: No

Additional Information
Explanation for why data not available: The data will be made available upon written reasonable request to the corresponding author. The data is still undergoing secondary analyses.
